# The Wnt Receptor Ryk Reduces Neuronal and Cell Survival Capacity by Repressing FOXO Activity During the Early Phases of Mutant Huntingtin Pathogenicity

**DOI:** 10.1371/journal.pbio.1001895

**Published:** 2014-06-24

**Authors:** Cendrine Tourette, Francesca Farina, Rafael P. Vazquez-Manrique, Anne-Marie Orfila, Jessica Voisin, Sonia Hernandez, Nicolas Offner, J. Alex Parker, Sophie Menet, Jinho Kim, Jungmok Lyu, Si Ho Choi, Kerry Cormier, Christina K. Edgerly, Olivia L. Bordiuk, Karen Smith, Anne Louise, Michael Halford, Steven Stacker, Jean-Philippe Vert, Robert J. Ferrante, Wange Lu, Christian Neri

**Affiliations:** 1 CNRS, UMR 8256, Laboratory of Neuronal Cell Biology and Pathology, Paris, France; 2 Sorbonnes Universités, University Pierre and Marie Curie (UPMC) Univ Paris 06, Paris, France; 3 INSERM, Unit 894, Paris, France; 4 Assistance Publique-Hopitaux de Paris (AP-HP), Charles Foix Hospital, Functional Exploration Unit, Ivry-sur-Seine, France; 5 Neurological Surgery Department, University of Pittsburgh, Pittsburgh, Pennsylvania, United States of America; 6 University of Southern California Keck School of Medicine, Eli and Edythe Broad Center for Regenerative Medicine and Stem Cell Research, Los Angeles, California, United States of America; 7 Department of Neurology, Boston University School of Medicine, Boston, Massachusetts, United States of America; 8 VA Bedford Geriatric Research Education and Clinical Center, Edith Nourse Rogers Memorial Veterans Hospital, Bedford, Massachusetts, United States of America; 9 Pasteur Institute, Cytometry Platform, Paris, France; 10 Peter MacCallum Cancer Center, East Melbourne, Victoria, Australia; 11 Mines ParisTech, Center for Computational Biology, Fontainebleau, France; 12 Curie Institute, Research Center, Paris, France; 13 INSERM, Unit 900, Paris, France; Baylor College of Medicine, United States of America

## Abstract

The Wnt receptor Ryk is an evolutionary-conserved protein important during neuronal differentiation through several mechanisms, including γ-secretase cleavage and nuclear translocation of its intracellular domain (Ryk-ICD). Although the Wnt pathway may be neuroprotective, the role of Ryk in neurodegenerative disease remains unknown. We found that Ryk is up-regulated in neurons expressing mutant huntingtin (HTT) in several models of Huntington's disease (HD). Further investigation in *Caenorhabditis elegans* and mouse striatal cell models of HD provided a model in which the early-stage increase of Ryk promotes neuronal dysfunction by repressing the neuroprotective activity of the longevity-promoting factor FOXO through a noncanonical mechanism that implicates the Ryk-ICD fragment and its binding to the FOXO co-factor β-catenin. The Ryk-ICD fragment suppressed neuroprotection by *lin-18*/Ryk loss-of-function in expanded-polyQ nematodes, repressed FOXO transcriptional activity, and abolished β-catenin protection of mutant htt striatal cells against cell death vulnerability. Additionally, Ryk-ICD was increased in the nucleus of mutant htt cells, and reducing γ-secretase PS1 levels compensated for the cytotoxicity of full-length Ryk in these cells. These findings reveal that the Ryk-ICD pathway may impair FOXO protective activity in mutant polyglutamine neurons, suggesting that neurons are unable to efficiently maintain function and resist disease from the earliest phases of the pathogenic process in HD.

## Abstract 2

A study of Huntington's disease reveals that neurons might fail to cope with maintaining their function during the pre-symptomatic, pathogenic phases of HD, possibly due to the early repression of key longevity-promoting transcription factors by abnormal developmental signaling.

## Author Summary

Neuronal cell decline in neurodegenerative disease can be caused by inherited mutations and involves neuronal dysfunction followed by neuronal death. The ability of neurons to cope with the chronic stress induced by mutant protein expression may determine the course of their decline and eventual demise. Although the pathophysiological importance of these stress responses has been previously shown, very little is known about the signaling networks that regulate neuronal homeostasis during the early presymptomatic—but pathogenic—phases of a neurodegenerative disorder such as Huntington's disease (HD). In particular, it remains unclear whether neuronal differentiation factors regulate stress response pathways during neurodegenerative disease and how this might impact the overall capacity of neurons to cope with stress and maintain their function. Here, we show that the Wnt receptor Ryk, a protein known to be important for neurogenesis, is increased in different animal models of HD, before or during the early phases of the disease process. Interestingly, increased levels of Ryk repress activity of the FOXO proteins—a family of transcription factors that play a role in cell survival/longevity and in neuronal homeostasis and protection. Ryk represses FOXO protective activity, possibly directly, through its intracellular domain, a product of γ-secretase–mediated cleavage previously implicated in the birth of new cortical neurons. This highlights the regulation of HD neuron survival by a Ryk-dependent pathway that is distinct from canonical Wnt/Ryk signaling. From our findings, we postulate that neurons are unable to develop an efficient FOXO-mediated survival response during the very early, pathogenic phases of HD.

## Introduction

Cell stress response factors are important for cells to maintain function in a large variety of normal and pathological contexts, including diseases linked to proteotoxicity [1]. Among these factors, the FOXO family of Forkhead transcription factors is central to longevity and cellular homeostasis [2],[3]. Additionally, FOXO factors may be important to the regulation of neuron survival in neurodegenerative diseases such as Huntington's disease (HD) [4],[5], a dominantly inherited CAG repeat disorder caused by expanded polyglutamines (polyQ) in the N-terminal portion of huntingtin (HTT) and characterized by striatal and cortical degeneration [6]. FOXO may indeed promote neuron survival in simple models of HD [7] and cellular proteostasis in simple models of Alzheimer's disease (AD) [8]. Interestingly, the canonical Wnt pathway component β-catenin may functionally interact with FOXO in oxidative stress signaling [9] and has a neuroprotective role in models of the early phases of the pathogenic process in HD [10]. This suggests that the canonical Wnt pathway may interact with the FOXO pathway to promote diseased-neuron function and survival, which is in line with the notion that neuronal differentiation factors such as Wnts may promote adult neuron survival [11]. However, Wnt signaling effectors may be impaired in HD and AD [12]–[14], raising the possibility that Wnt pathways may have a dual role in the regulation of neurodegeneration. Here, we hypothesized that alteration of Wnt pathways might antagonize the FOXO pathway to compromise cell stress response and neuronal resistance during the earliest phases of the pathogenic process in neurodegenerative disease such as HD. To test for this hypothesis, we used *Caenorhabditis elegans* transgenics that recapitulate an early phase of mutant HTT toxicity, namely neuronal dysfunction before cell death [15]. At the young adult stage, these animals show a dramatic loss of response to light touch produced by polyQ-expanded exon-1 like HTT fused to GFP in touch receptor neurons [15]. To assess the mechanisms that underlie the dysfunction of these neurons, we performed a microarray analysis of primary neurons upon Fluorescence Activated Cell Sorting (FACS) of embryonic cells. This analysis emphasized the deregulation of neuronal differentiation genes, notably genes that are up-regulated in expanded-polyQ nematodes and in the brain of HD patients such as Ryk. Ryk is an evolutionary-conserved Wnt receptor (*lin-18* in *C. elegans*) that is important to neurogenesis and axon guidance [16]–[18] and that is involved in the regulation of developmental/postdevelopmental processes such as planar cell polarity [19],[20], regeneration [21], and hematopoietic repopulation [22]. Further investigation revealed that loss-of-function (LOF) of *lin-18*/Ryk in polyQ nematodes and reduction of Ryk levels in mouse striatal cells derived from HdhQ111 knock-in mice [23] strongly protected from expanded polyQ/mutant HTT. Neuroprotection by *lin-18* LOF in expanded-polyQ nematodes, a cell-autonomous process, required the neuroprotective factor *daf-16*/FOXO [7], suggesting that *lin-18* represses the neuroprotective activity of *daf-16* in these animals. The intracellular domain of Ryk (Ryk-ICD), a γ-secretase cleavage product that translocates in the nucleus to control neurogenesis [16],[17], was found to bind to the FOXO partner β-catenin, suggesting that Ryk-ICD may trigger the repression of FOXO by increased levels of Ryk in mutant polyQ neurons. In support of this mechanism, Ryk-ICD overexpression was sufficient to repress the transcriptional activity of FOXO3a, a protein that promotes the survival of mutant htt striatal cells. Additionally, LIN-18 ICD expression was sufficient to suppress neuroprotection by *lin-18* LOF in expanded-polyQ nematodes. This mechanism was further supported by results in mutant htt cells showing that (*i*) Ryk-ICD overexpression abolished the protective activity of β-catenin, which is consistent with the possibility that an excess of Ryk-ICD may bind and block this survival protein; (*ii*) reducing presenilin PS1 levels (which is protective) compensated for the cytotoxicity of full-length Ryk but not that of Ryk-ICD, implicating this γ-secretase in the toxic effects of Ryk; and (*iii*) nuclear levels of endogenous Ryk-ICD were increased compared to normal htt cells, corroborating a role for the Ryk-ICD pathway in triggering abnormal Ryk signaling in mutant htt cells. Finally, Ryk was suggested to have a pathological role in HD as emphasized by the early stage (before or during the onset of pathology) increase of Ryk in expanded-polyQ nematodes [7] and the neostriatum of 140CAG knock-in mice [24]. Collectively, these results suggest that Ryk and its ICD fragment may reduce the ability of mutant polyQ neurons to handle cell stress and maintain function by repressing FOXO protective activity, which may occur during the earliest phases of the pathogenic process in HD.

## Results

### Microarray Data Analysis Highlights Axon Guidance Pathways

To explore the pathways that underlie the early phases of expanded-polyQ neurotoxicity, we performed a microarray analysis of mRNAs extracted from *C. elegans* touch receptor cells. To this end, we used transgenic nematodes expressing polyQ-expanded (128Q) and normal (19Q) N-terminal HTT fused to GFP under the control of the *mec-3* promoter [15], and transgenic nematodes expressing only GFP under the control of the same promoter as a control. In this model, expanded polyQ expression produces a strong level of neuronal dysfunction not found in normal polyQ animals, namely the loss of response to light touch [15]. GFP-positive cells were purified by cell sorting from primary cultures of embryonic cells prior to mRNA extraction and microarray analysis. Forty-one genes were deregulated in 19Q cells compared to cells expressing GFP only (Table S1). A total of 2,070 genes were deregulated in 128Q cells compared to 19Q cells (Table S2). Interestingly, only 18 of the 2,070 genes were also deregulated in 19Q nematode cells, suggesting that our microarray analysis has provided clean and specific information on the transcriptomic effects of expanded-polyQ expression. To analyze the biological content of these data, we used several methods including Gene Ontology analysis, Gene Set Enrichment Analysis (GSEA), and a powerful network-based method based on Fourier analysis (see Text S2). In contrast to the GO analysis (Figure S1), the GSEA and Fourier analyses highlighted several processes previously suspected to be altered in HD (see Text S1, Figure S2, Figure S3, and Tables S3–S5), suggesting that nematode data are relevant to HD pathogenesis. Additionally, cell differentiation pathways such as Wnt signaling were emphasized as components potentially involved in expanded-polyQ neuron dysfunction, a trend also emphasized by the network-based analysis of data resulting from a large-scale functional RNAi screen in our expanded-polyQ nematodes [25].

Among the up-regulated Fourier modules, module 40 (Wnt/TGF-β signaling) was of particular interest (Figure S4). This module suggested that *lin-18*/Ryk, a Wnt receptor important during neurogenesis and axon guidance [26],[27], is up-regulated in neurons expressing expanded polyQs, which was confirmed by qRT-PCR (Table S5). To enhance the prioritization of candidate genes, we focused on pathways and processes that were highlighted by GSEA and by the Fourier analysis and that contained evolutionary conserved druggable [28] genes (Table S6). Up-regulated module 40 was again pointed out, as it contained the largest number of genes in common with the GSEA and Fourier analyses (see Wnt, cell cycle, and TGF-β in Table S4) as well as 3 of the 25 genes deregulated in 128Q nematode cells and in the caudate nucleus of HD patients [29], among which was *lin-18*/Ryk.

### LOF of *lin-18*/Ryk Protects Nematode Neurons from Expanded PolyQs

**Figure 1:**
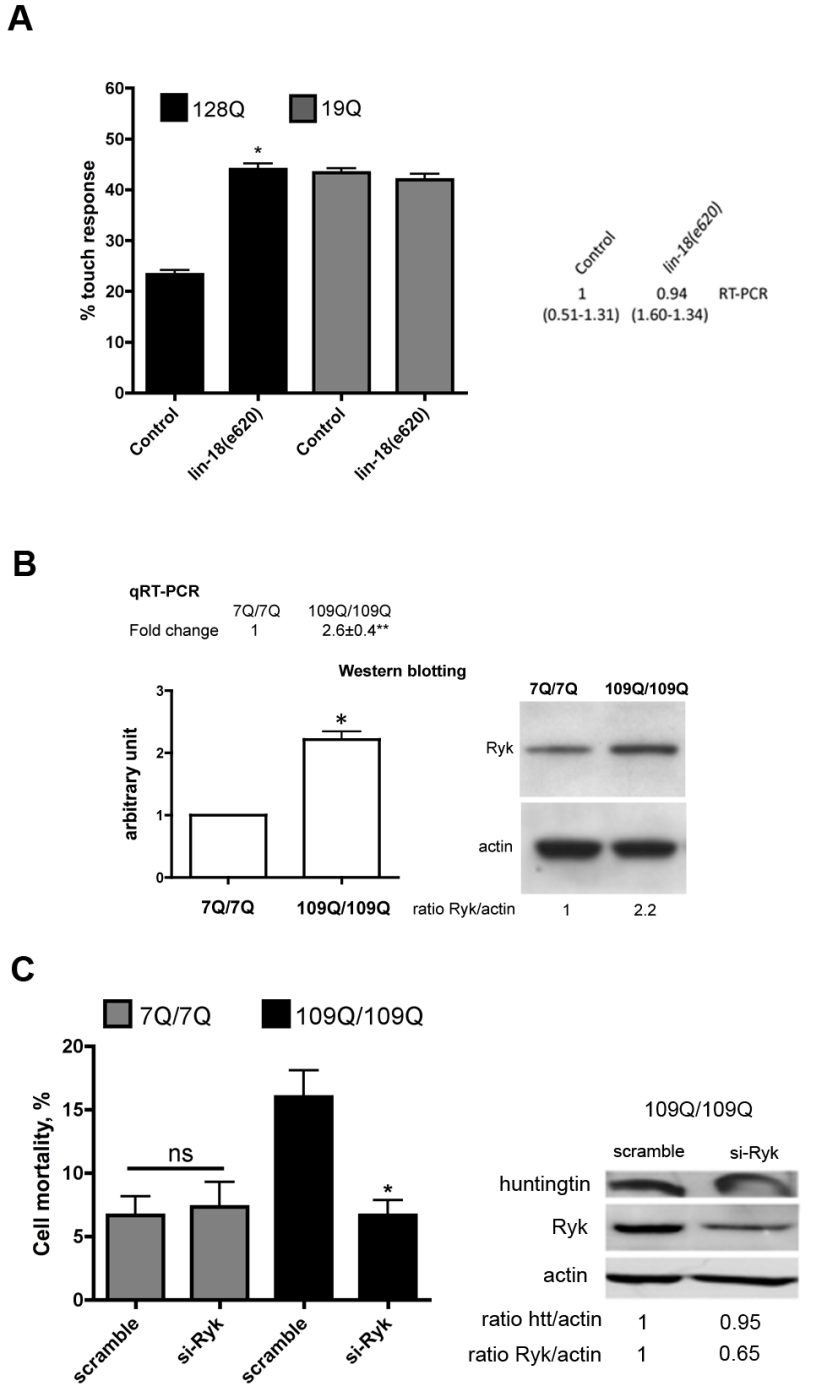
Analysis of Ryk in polyQ nematodes and striatal cells derived from HdhQ111 mice. (A) Modulation of touch response of polyQ nematodes by LOF of *lin-18*/RYK. Shown are the results in *C. elegans* transgenics expressing expanded (128Q) or normal (19Q) exon-1–like htt transgenes in touch receptor neurons. The 128Q-mediated loss of touch response is ameliorated by LOF of *lin-18*/RYK. No effects are detected in 19Q nematodes. Data are mean ± SEM with more than 200 animals tested per genotype. *p<0.001 versus 128Q controls. LOF of *lin-18*/RYK does not modify 128Q transgene expression levels as tested by qRT-PCR (right panel, data are mean ± SEM with *n* = 5). Significance was tested using one-way ANOVA, with correction for multiple testing by Tukey's Multiple Comparison Test. (B) Mutant htt (109Q/109Q) striatal cells have increased Ryk levels. Data for qRT-PCR are mean ± SEM (*n* = 7). **p<0.01 versus normal htt (7Q/7Q) cells. Data for Western blotting are mean ± SD (*n* = 3). **p<0.01 versus normal htt (7Q/7Q) cells. Significance was tested using paired *t* tests. (C) Reducing Ryk levels decreased the mortality of 109Q/109Q striatal cells induced by serum deprivation compared to scramble situation, with no effect detected in 7Q/7Q cells. Western blotting was used to test expression levels as 109Q/109Q cells do not display HTT aggregation. Mutant htt levels were unchanged by knockdown of Ryk. Data are mean ± SD (*n* = 4). *p<0.01 versus scramble. The RNA sequences shown are indicated in the Materials and Methods section. Significance was tested using paired *t* tests.

In the up-regulated Fourier module 40 (Figure S4), the conserved *lin-18* gene was of interest in the Wnt pathway as a druggable gene that may be deregulated in the touch receptor cells of expanded-polyQ nematodes and caudate nucleus of HD patients. Although target gene activation is an option for developing disease-modifying strategies, target inhibition is usually regarded as a more easily achievable approach. Interestingly in this respect, neuronal dysfunction was abolished by *lin-18*/Ryk LOF in 128Q nematodes with no effect in 19Q nematodes and no change in transgene expression (Figure 1A). These results suggested that *lin-18* up-regulation is toxic to 128Q neurons and that Ryk inhibition may provide protection from mutant polyQ cytotoxicity.

### Reducing Ryk Levels Decreases the Vulnerability of Mutant htt Striatal Cells

Having observed that neuronal dysfunction was suppressed by *lin-18*/Ryk LOF in 128Q nematodes, we tested if Ryk inhibition could decrease the cell death caused by full-length mutant HTT. To this end, we used striatal cells derived from the HdhQ111 knock-in mice [23]. Mutant htt (109Q/109Q) striatal cells are abnormally susceptible to cell death as induced by serum deprivation [23], a phenotype suitable for identifying modifiers of cell vulnerability [30]. As indicated by qRT-PCR and Western blotting, Ryk mRNA and protein levels are two times higher in 109/109Q cells compared to normal (7Q/7Q) cells (Figure 1B). To test whether reducing Ryk levels may promote mutant htt striatal cell survival, we subjected 109Q/109Q and 7Q/7Q cells to Ryk siRNA treatment. Ryk siRNA treatment enhanced the survival of 109Q/109Q cells with no effect on 7Q/7Q cells, an effect unrelated to a change in HTT expression (Figure 1C). This was consistent with neuroprotection by *lin-18*/Ryk LOF in 128Q nematodes, further suggesting that Ryk has a pathological role in HD neurons.

### Neuroprotection by *lin-18*/Ryk LOF Requires *daf-16*/FoxO Activity

**Figure 2:**
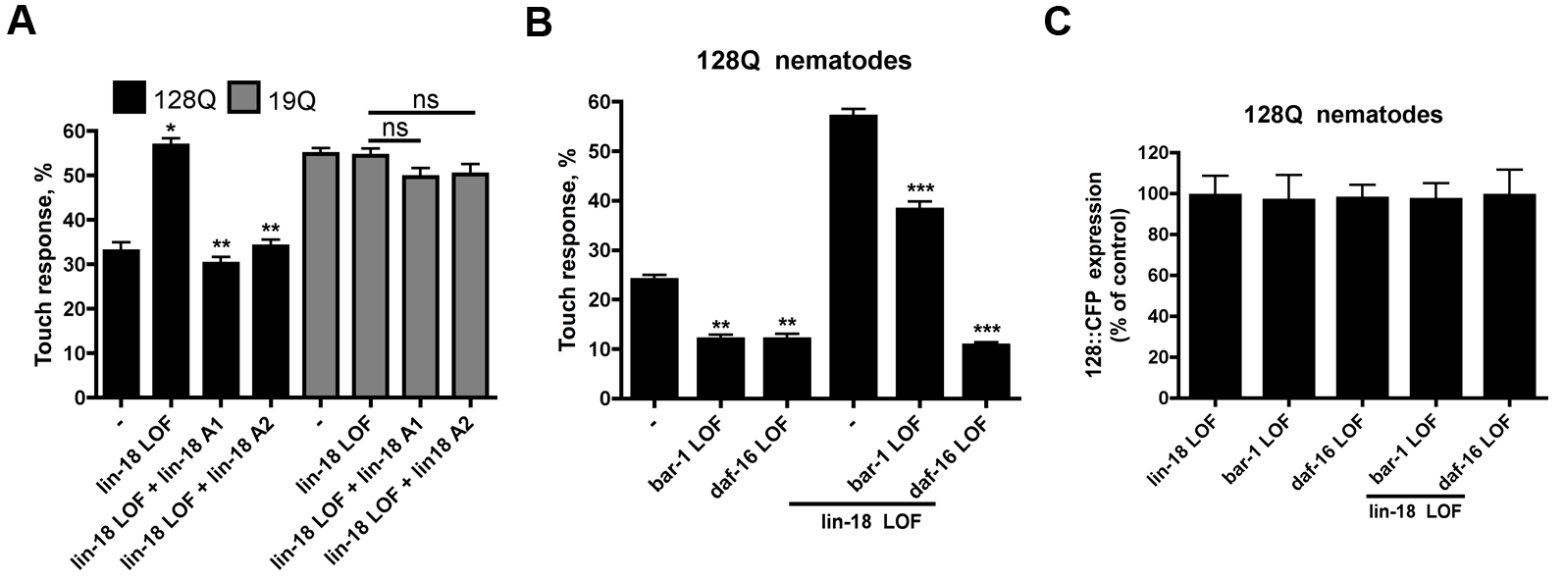
Neuroprotection by *lin-18*/Ryk LOF is a cell-autonomous process that requires *daf-16*/FoxO activity. (A) Expression of *lin-18* cDNA in touch receptor neurons using the *mec-3* promoter abolishes the neuroprotective activity of *lin-18* LOF in 128Q nematodes with no effect detected in 19Q nematodes as tested in two independent extrachromosomal arrays (A1, A2) per polyQ genotype. The expression of *lin-18* cDNA was confirmed by RT-PCR followed with enzymatic restriction for all of the arrays generated (unpublished data). Data are mean ± SEM (more than 200 animals tested). *p<0.001 compared to 128Q transgenics; **p<0.001 compared to 128Q;*lin-18* nematodes. (B) Neuron dysfunction is aggravated by *bar-1*/β-catenin or *daf-16*/FoxO LOF in 128Q nematodes, and protection from 128Q toxicity by *lin-18* LOF is reduced by LOF of *bar-1* and suppressed by LOF of *daf-16*. Data are mean ± SEM (more than 200 animals tested). **p<0.001 compared to 128Q transgenics; ***p<0.001 compared to 128Q;*lin-18* nematodes. (C) *lin-18*, *bar-1* and *daf-16* LOF alone or in combination do not change transgenic protein expression levels in 128Q nematodes. Data are mean ± SD (*n* = 3). Significance was tested using one-way ANOVA, with correction for multiple testing by Tukey's Multiple Comparison Test.

We next sought to examine the mechanisms underlying neuroprotection by Ryk inhibition. We first tested whether neuroprotection by *lin-18*/Ryk LOF in expanded-polyQ nematodes (see Figure 1A) occurred in a cell-autonomous manner. The expression of *lin-18* cDNA in touch receptor neurons (4 ng/µl) using the *mec-3* promoter abolished neuroprotection by *lin-18* LOF in 128Q nematodes with no effect detected in 19Q nematodes as observed in two independent arrays per polyQ genotype (Figure 2A), indicating that neuroprotection by *lin-18* LOF is cell autonomous. Ryk participates in canonical Wnt signaling [26] and there is a functional interaction between β-catenin, a downstream effector of canonical Wnt, and FOXO in oxidative stress signaling [9]. We thus tested whether *lin-18* required *bar-1*/β-catenin and *daf-16*/FoxO activity to modulate neuronal dysfunction in 128Q nematodes. In 19Q nematodes, LOF of *lin-18* (see Figure 1A), *daf-16* (see [1]), and *bar-1* (see [10]) had no effect on touch response. In 128Q nematodes, *bar-1* LOF exacerbated the loss of touch response (Figure 2B), with no change in transgenic protein expression (Figure 2C), suggesting that *bar-1* normally protects touch neurons from 128Q. The same applied to *daf-16* LOF (Figure 2B/C), as previously observed [7]. In 128Q;*lin-18(e620)* animals, neuroprotection by *lin-18* LOF was reduced by LOF of *bar-1* (Figure 2B) with no change in transgenic protein expression (Figure 2C), suggesting that *lin-18* activity requires *bar-1*. However, the effect of *bar-1* LOF in 128Q;*lin-18(e620)* nematodes was partial, suggesting a role for other genes. In contrast, neuroprotection by *lin-18* LOF was suppressed by *daf-16* LOF (Figure 2B), an effect unrelated to a change in transgenic protein expression (Figure 2C), indicating that neuroprotection by *lin-18* LOF fully requires *daf-16*. Given that *daf-16* is normally neuroprotective (Figure 2B), this indicated that *lin-18* may repress *daf-16* activity in 128Q nematodes, which led us to investigate the mechanisms by which Ryk may repress FOXO activity.

### The Ryk ICD Binds to β-Catenin

Ryk signals through multiple mechanisms such as the canonical Wnt pathway and the nuclear translocation of its cleaved ICD to regulate neuronal differentiation [16]. This suggests that one mechanism for Ryk to repress FOXO in mutant polyQ neurons might be the deregulation of canonical Wnt, a neuroprotective pathway [11]. However, LOF of *pop-1*/TCF does not appear to modulate 128Q neurotoxicity in *C. elegans* nematodes (unpublished data), suggesting that the outcome of the canonical Wnt pathway is not critical to mutant polyQ neuron survival. We thus hypothesized that another mechanism for Ryk to repress FOXO in mutant polyQ neurons could involve a noncanonical mechanism, namely the binding of the Ryk-ICD to FOXO or its partner β-catenin.

**Figure 3:**
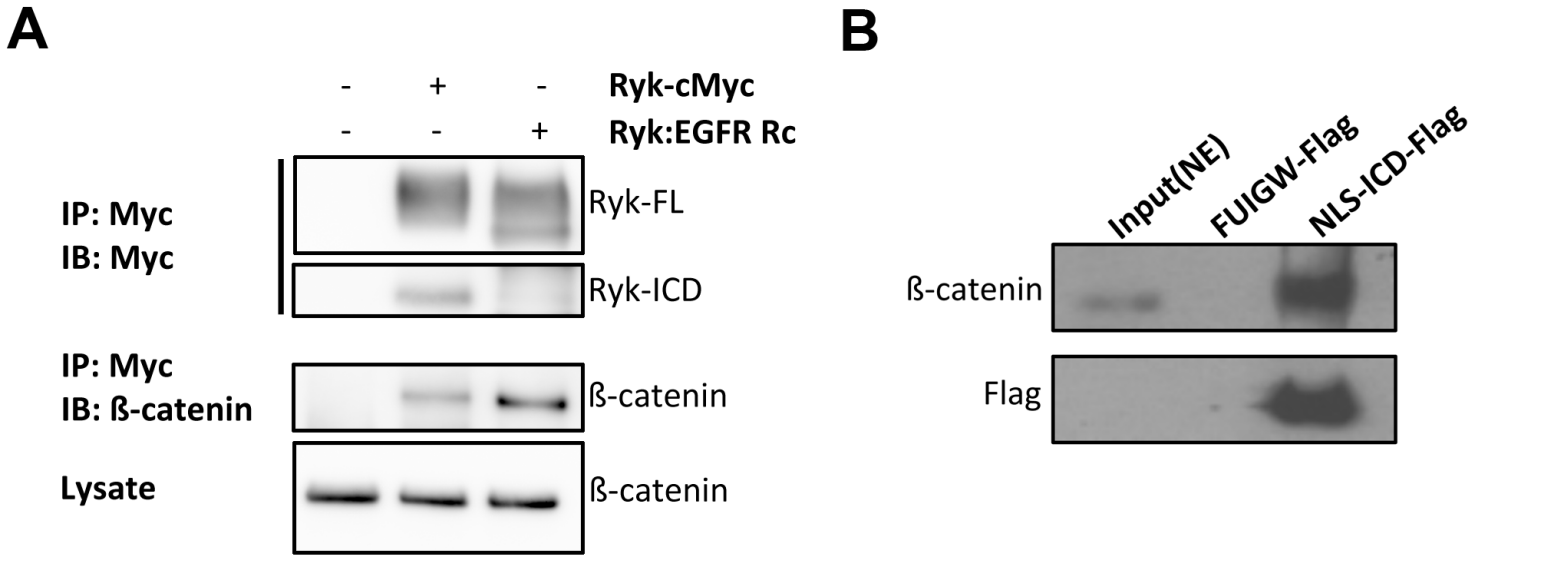
Ryk ICD binds to β-catenin. (A) β-catenin binds to Ryk. Constructs of Myc-tagged Ryk or uncleavable Ryk (Ryk:EGFR Rc) were transfected into 293T cells. Ryk proteins were immunoprecipitated with anti-Myc antibody, and beta-catenin associated with Ryk was determined by immunoblotting. Ryk ICD can be detected in the cells expressing wild-type Ryk. (B) The ICD of Ryk binds to β-catenin. Cells expressing Flag-tagged Ryk-ICD were used for anti-Flag immunoprecipitation. The ICD and associated β-catenin were determined by Western blot. NE, nuclear extract; FUIGW-FLAG, vector FUIGW plus a Flag sequence.

It was previously shown that β-catenin associates with Ryk [31]. Consistent with these findings, we also observed that β-catenin binds to Ryk by using constructs that code for Ryk with a Myc tag at the C-terminal end or an uncleavable Ryk mutant (Ryk: EGFRRc, a chimeric construct in which the transmembrane region was replaced with that of the EGF receptor) [16]. These constructs were transfected into 293T cells (derived from human kidney cells) and cell lysates subjected to immunoprecipitation using an anti-Myc antibody followed by immunoblotting. In the cells expressing Ryk or uncleavable Ryk, immunonoprecipitation of Myc-tagged Ryk pulled down endogenous β-catenin (Figure 3A), as expected.

To examine if Ryk-ICD binds to β-catenin, Flag-tagged Ryk-ICD constructs were transfected into 293T cells. β-catenin was detected in the anti-Flag immunoprecipitate, suggesting that Ryk-ICD is sufficient to associate with β-catenin (Figure 3B). Similar experiments aimed at testing for binding of Ryk-ICD to FOXO3a, a neuroprotective FOXO protein [32], did not detect any interaction (unpublished data). Together, these results suggested that Ryk might signal onto FOXO through binding of its ICD fragment to the FOXO co-factor β-catenin.

### Ryk-ICD Represses FOXO3a Activity in Luciferase Reporter Assays

**Figure 4:**
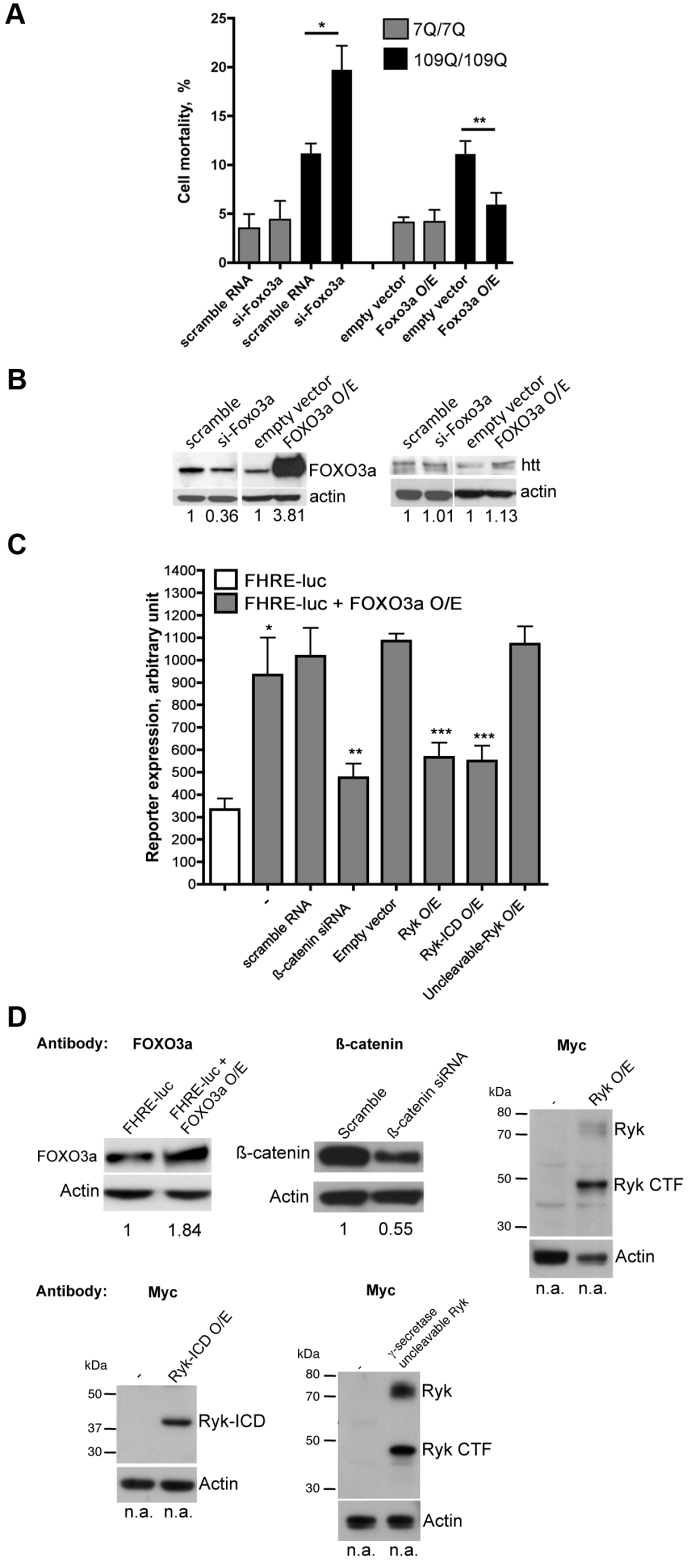
The Ryk ICD represses the transcriptional activity of FOXO3a, a protein that protects from mutant HTT. (A) Foxo3a siRNA treatment enhances the mortality of mutant htt striatal cells subjected to serum deprivation, whereas FOXO3a overexpression (O/E) has the opposite effect. Data are mean ± SD (*n* = 4). *p<0.001 compared to scramble; **p<0.001 compared to empty vector control. (B) Representative Western blots showing decreased (si-Foxo3a) or increased (FOXO3a O/E) FOXO3a levels and no change in HTT protein levels. (C) FOXO transcriptional activity was measured in normal htt mouse striatal cells. Cells were cultured in normal conditions and co-transfected with a construct encoding FOXO3a together with the reporter FHRE-luciferase, which contains three canonical FOXO binding sites, and an internal *Renilla* luciferase reporter construct. Luciferase and *Renilla* luciferase activities were measured and the ratio (luciferase/*Renilla* luciferase)10,000 calculated. Data are mean ± SD of four independent experiments performed in triplicate. Treatment with β-catenin siRNA, full-length Ryk cDNA, and Ryk-ICD cDNA reduces luciferase activity to similar levels, whereas treatment with uncleavable Ryk showed no effect. *p<0.001 compared to FHRE-luc, **p<0.001 compared to scramble RNA and ***p<0.001 compared to FOXO3a O/E. Significance was tested using one-way ANOVA, with correction for multiple testing by Tukey's Multiple Comparison Test. (D) Representative Western blots showing increased levels of FOXO3a and decreased levels of β-catenin, and expression of Myc-tagged Ryk, Myc-tagged Ryk-ICD, and Myc-tagged γ-secretase–uncleavable Ryk (all proteins with a Myc tag at the C-terminal end). The Myc-tagged Ryk and γ-secretase–uncleavable Ryk proteins were detected as two fragments, one corresponding to the full-length Ryk precursor (Ryk) and one corresponding to a Ryk CTF (Ryk CTF) resulting from proteolytic cleavage in the extracellular domain near the transmembrane domain. The full-length Ryk precursor is less abundant for wild-type Ryk expression compared to mutant Ryk expression (see Results for the discussion of Ryk expression profiles).

We then sought to test whether an excess of Ryk may repress FOXO transcriptional activity. We also tested whether an excess of the γ-secretase–cleaved ICD of Ryk may have a similar effect, as the Ryk-ICD fragment associates with β-catenin (see Figure 3B), a protein that promotes FOXO transcriptional activity [9]. We examined the activity of FOXO3a, a protein that is neuroprotective in models of motor neuron disease [32], also protecting against cell death associated with mutant HTT in striatal cells from HdhQ111 knock-in mice (Figure 4A/B). From here, we further examined the molecular relationships between FOXO3a, β-catenin, and Ryk in mutant polyQ toxicity. To this end, constructs encoding FOXO3a together with a Forkhead responsive element (FHRE)-luciferase reporter and internal control reporter were utilized to transfect mouse striatal cells that express normal HTT. Reducing β-catenin levels reduced luciferase activity compared to control cells (Figure 4C/D), which is consistent with the ability of β-catenin to promote FOXO transcriptional activity in mouse cells [9]. Overexpressing Ryk reduced luciferase activity to levels comparable to those observed for β-catenin siRNA treatment (Figure 4C), suggesting that Ryk up-regulation can repress FOXO3a transcriptional activity. Western blotting indicated that this effect might be attributable to full-length Ryk and to a Ryk C-terminal fragment (Ryk CTF) of ∼47 kDa (Figure 4D). However, the CTF fragment is too large to be the γ-secretase cleavage product of Ryk (Ryk-ICD, ∼40 kDa). The size of this fragment suggests that Ryk CTF contains the ICD plus the transmembrane domain and a portion of the extracellular domain, which is consistent with the possibility that other proteases might cleave the extracellular domain of Ryk near the transmembrane domain [33]. The Ryk-ICD fragment was not visible, which might be attributable to a very short half-life of this fragment similar to what has been observed for the γ-secretase–cleaved intracellular fragment of transmembrane proteins such as the amyloid precursor protein (APP) [34]. Interestingly, Ryk-ICD overexpression produced a similar reduction of luciferase activity compared to Ryk overexpression (Figure 4C/D), indicating that Ryk-ICD is sufficient to repress FOXO3a transcriptional activity. Furthermore, overexpressing a mutant form of Ryk that cannot undergo γ-secretase cleavage (Ryk: EGFRRc) showed no effect on luciferase activity (Figure 4C). Mutant Ryk was detected as one fragment corresponding to full-length Ryk and one fragment corresponding to Ryk CTF (Figure 4D). In contrast to cells transfected with wild-type Ryk, the full-length Ryk precursor appeared to be more abundant in cells transfected with mutant Ryk (Figure 4D). This suggests that blocking the γ-site of Ryk may also block Ryk CTF generation, an effect that was previously shown for APP [35] and that further points to putative similarities between Ryk and APP processing. Collectively, these results suggested that the generation of the Ryk-ICD fragment is important for the cytotoxic action of Ryk.

### LIN-18 ICD Suppresses Neuroprotection by *lin-18* LOF in Expanded-PolyQ Nematodes

**Figure 5:**
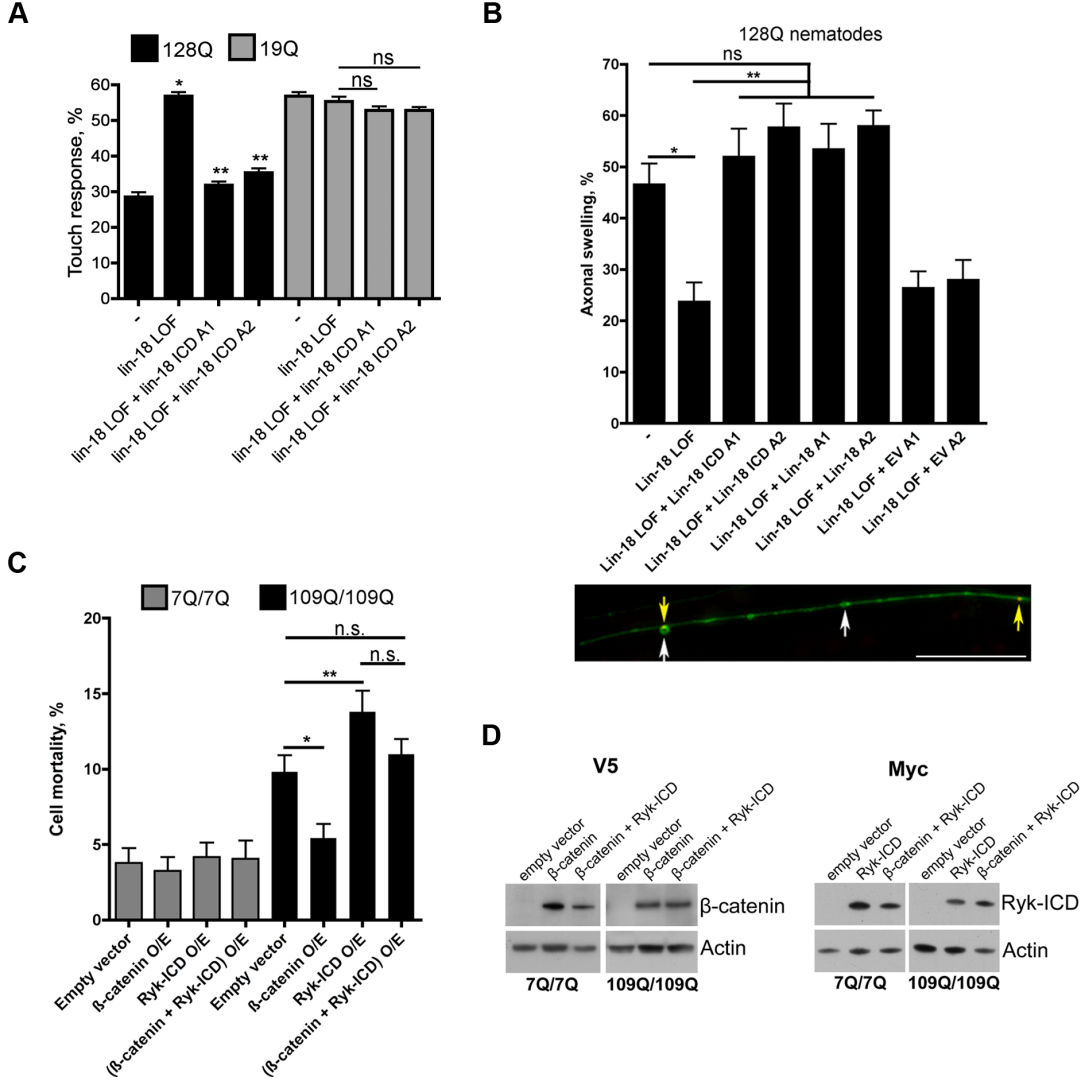
The Ryk ICD is cytotoxic in *C. elegans* neurons and mouse striatal cells expressing expanded polyQs. (A) Expression of LIN-18 ICD cDNA (4 ng/µl) in touch receptor neurons using the *mec-3* promoter is sufficient to abolish the neuroprotective activity of *lin-18* LOF in 128Q nematodes with no effect detected in 19Q nematodes as tested in two independent extrachromosomal arrays (128Q: A1 is ID1333, A2 is ID1334; 19Q: A1 is ID1331, A2 is ID1332; see also Table S8) per polyQ genotype. The expression of LIN-18 ICD cDNA was confirmed by RT-PCR for all of the arrays generated. Data are mean ± SEM (more than 200 animals tested). **p<0.001 compared to 128Q animals, **p<0.001 compared to 128Q;*lin-18* animals. ns, not significant. Significance was tested using one-way ANOVA, with correction for multiple testing by Tukey's Multiple Comparison Test. (B) Expression of LIN-18 ICD cDNA (4 ng/µl) in touch receptor neurons using the *mec-3* promoter is also sufficient to abolish the protective activity of *lin-18* LOF on axonal swelling in the PLM neurons of 128Q nematodes as tested in two independent extrachromosomal arrays (Lin-18 ICD: A1 is ID1333, A2 is ID1334; Lin-18: A1 is ID1325, A2 is ID1326). The expression of LIN-18 ICD cDNA was confirmed by RT-PCR for all of the arrays generated. Expression of empty vector (4 ng/µl) showed no effect as tested in two independent extrachromosomal arrays (A1 is ID1339, A2 is ID1340). Data are mean ± SEM (more than 200 animals tested). *p<0.001 compared to 128Q animals, **p<0.001 compared to 128Q;*lin-18* animals. ns, not significant. Significance was tested using one-way ANOVA, with correction for multiple testing by Tukey's Multiple Comparison Test. The lower panel shows a representative image of axonal swelling in the anterior process of posterior touch receptor neurons of 128Q nematodes co-expressing HTT1-57::CFP and YFP [7]. Swelling (white arrows, YFP signals are pseudocolored in green) and HTT::CFP aggregation (yellow arrows, CFP signals are pseudocolored in red) are shown. Magnification is 100× and scale bar is 5 µM. (C) Overexpressing either V5-tagged β-catenin or Myc-tagged Ryk-ICD or both has no effect on the mortality induced by serum deprivation in normal htt striatal cells. Overexpressing β-catenin reduces the mortality induced by serum deprivation in mutant htt striatal cells, whereas overexpressing the Ryk-ICD aggravates cell mortality. Co-expressing Ryk-ICD and β-catenin resulted in cell mortality levels that are similar to those induced by empty vector overexpression. Data are mean ± SD (*n* = 4). *p<0.01 and **p<0.05 compared to empty vector. ns, not significant. Significance was tested using paired *t* tests. (D) Representative Western blot showing increased V5-tagged β-catenin and Myc-tagged Ryk-ICD levels after transfection of 7Q/7Q and 109Q/109Q striatal cells.

We next tested whether the Ryk ICD has a role in mutant polyQ cytotoxicity as predicted by its ability to repress FOXO transcriptional and neuroprotective activity. In 128Q nematodes, lin-18*/*Ryk LOF is neuroprotective, and this effect is cell autonomous, as LIN-18 overexpression in touch receptor neurons suppresses the neuroprotective effect of *lin-18* LOF (see Figure 2A). Interestingly, overexpression (at a dose of 4 ng/µl) of the LIN-18 ICD was sufficient to suppress the protective effect of *lin-18* LOF on touch response in 128Q nematodes with no effect detected in 19Q nematodes as tested in two independent arrays per polyQ genotype (Figure 5A). Although overexpressing LIN-18 ICD at higher doses (40 ng/µl) showed a trend towards exacerbation of 128Q cytotoxicity but did not reach statistical significance relative to control (Figure S5), overexpressing LIN-18 ICD at 40 ng/µl produced cytotoxicity in 19Q nematodes (Figure S5), suggesting that LIN-18 ICD has dose-dependent effects in polyQ;*lin-18* nematodes. The overexpression (4 ng/µl) of LIN-18 ICD was also sufficient to suppress the protective effect of *lin-18* LOF on axonal swelling (Figure 5B), accompanying the loss of touch response in 128Q nematodes [15]. Given that overexpressing the Ryk-ICD fragment is sufficient to reduce FOXO transcriptional activity (Figure 4C/D), this further suggested that the Ryk-ICD fragment mediates Ryk cytotoxicity.

### Ryk-ICD Is Cytotoxic in Mouse Striatal Cells Expressing Mutant htt

Next, we tested whether overexpressing the Ryk-ICD fragment may be cytotoxic in striatal cells derived from HdhQ111 knock-in mice [23] and whether this involves β-catenin activity. To this end, striatal cells were transfected with either a construct coding for Myc-tagged Ryk-ICD or a construct coding for V5-tagged β-catenin, or both, and these cells were subjected to serum deprivation. The overexpression of Ryk-ICD showed no effect on normal htt cell survival (Figure 5C/D). These results are consistent with the absence of toxicity for LIN-18 and LIN-18 ICD overexpression in normal-polyQ nematodes (see Figure 2A, Figure 5A), supporting a model in which Ryk up-regulation represses a mechanism (FOXO activity) that specifically promotes mutant polyQ neuron survival. Although mutant htt cells have high endogenous Ryk levels (see Figure 1B), overexpressing Ryk-ICD potentiated cell mortality (Figure 5C/D). This effect was moderate, however, suggesting that Ryk toxicity is close to a maximum in these cells. Consistent with the pro-survival role of β-catenin, overexpressing β-catenin reduced the cell death of mutant htt cells with no effect detected in normal htt cells (Figure 5C/D). The protective effect of β-catenin overexpression in mutant htt cells was abolished by Ryk-ICD overexpression (Figure 5C/D), suggesting that Ryk-ICD may antagonize β-catenin activity. There was no significant difference between the mortality of mutant htt cells transfected with Ryk-ICD alone and that of cells co-transfected with Ryk-ICD and β-catenin (Figure 5C/D). Nonetheless, cells co-transfected with Ryk-ICD and β-catenin showed similar levels of mortality compared to cells transfected with the empty vector (Figure 5C/D), suggesting that β-catenin overexpression may compensate for Ryk-ICD cytotoxicity to that extent. As such, these results might just reflect the parallel activity of two proteins with antagonistic properties in the regulation of cell survival. However, given that Ryk-ICD binds to β-catenin (see Figure 3), these results supported a model in which a functional cross-talk between Ryk-ICD and β-catenin contributes to Ryk-ICD cytotoxicity.

### Reducing Presenilin 1 Levels Compensates for the Cytotoxicity of Full-Length Ryk in Mutant htt Mouse Striatal Cells

**Figure 6:**
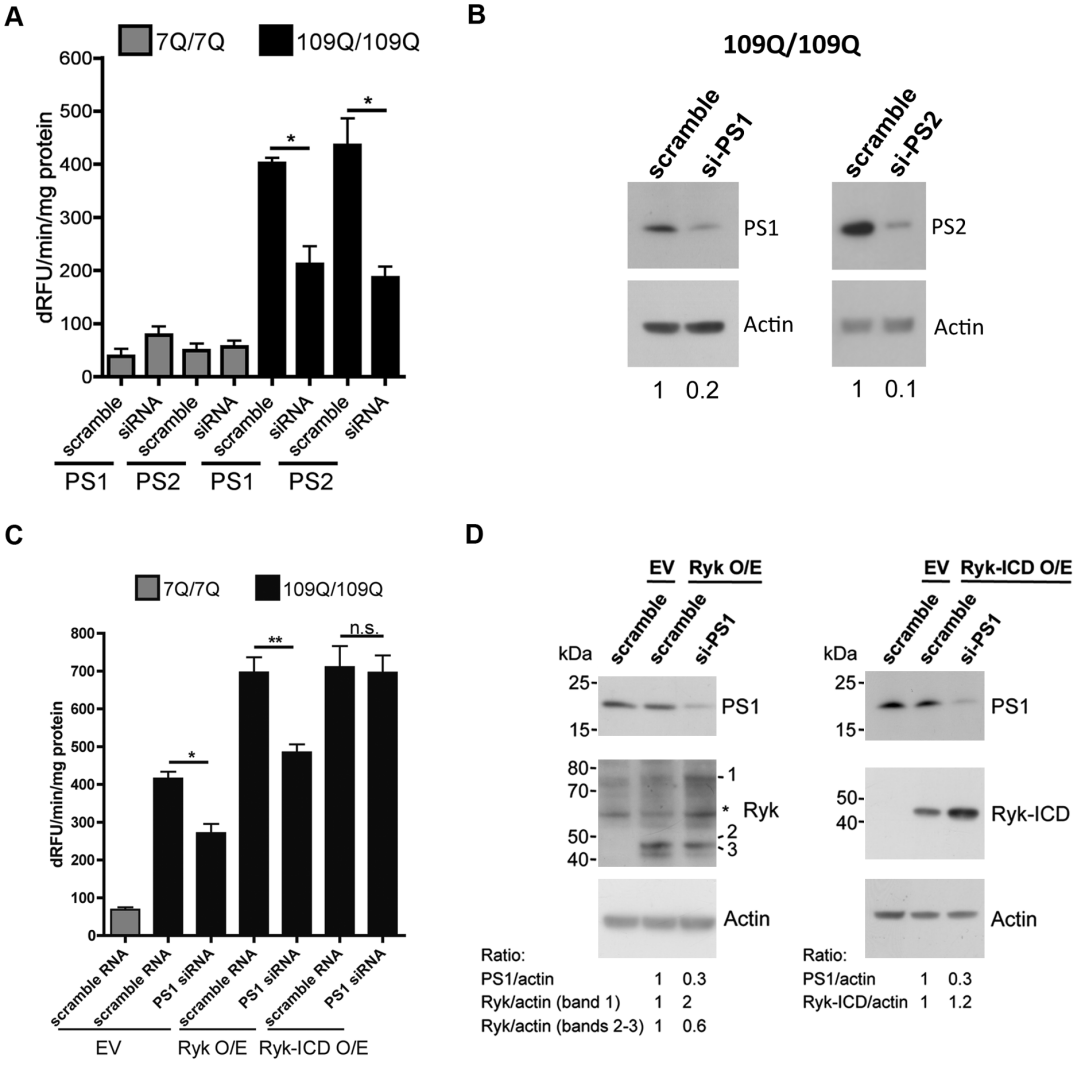
Reducing presenilin 1 expression counteracts the cytotoxicity of full-length Ryk overexpression in mutant htt striatal cells. Assays were performed using caspase 3/7 activity in cells subjected to serum deprivation. (A) PS1 and PS2 siRNA treatment enhances the viability of mutant htt striatal cells. Data are mean ± SD (*n* = 3), *p<0.01 compared to scramble RNA. Significance was tested using one-way ANOVA, with correction for multiple testing by Tukey's Multiple Comparison Test. (B) Representative Western blots showing decreased levels of PS1/PS2. (C) Knockdown of PS1 reduces the cytotoxic effects of overexpressing full-length Ryk in mutant htt striatal cells, with no effect detected on the cytotoxic effects of overexpressing Ryk-ICD. Data are mean ± SD (*n* = 4), *p<0.05 and **p<0.01 compared to scramble RNA. Significance was tested using one-way ANOVA, with correction for multiple testing by Tukey's Multiple Comparison Test. EV, empty vector; ns, not significant. (D) Representative Western blots showing decreased levels of PS1 and expression of Myc-tagged Ryk species and Myc-tagged Ryk-ICD. *Nonspecific signal.

Our results in *C. elegans* neurons and mouse striatal cells suggest a model in which the repression of FOXO3a activity by Ryk in mutant polyQ cells is mediated by the Ryk-ICD fragment, a γ-secretase cleavage product. The γ-secretase complex has been previously implicated in HD through its role in HTT cleavage and production of N-terminal fragments CpA and CpB [36]. Here, we sought to investigate the protective role of the γ-secretase complex relative to the pathological activity of Ryk in mutant polyQ cells. To this end, we tested whether decreasing the activity of the γ-secretase complex might promote the survival of mutant htt mouse striatal cells and whether this effect might compensate for Ryk toxicity in these cells. At this point in our study, we elected to measure caspase 3/7 activity instead of counting picnotic nuclei in order to perform faster and complementary measures of striatal cell mortality. We observed that reducing the expression of presenilins PS1 or PS2 enhanced the viability of mutant htt striatal cells, with no effect detected in normal htt cells (Figure 6A/B). Most importantly, reducing the levels of PS1 compensated for the cytotoxic effect of overexpressing full-length Ryk but not that of overexpressing Ryk-ICD in mutant htt cells (Figure 6C). The compensatory effects of reducing PS1 levels on Ryk cytotoxicity was accompanied by an apparent increase of full-length Ryk levels as inferred from Western blot analysis (Figure 6D). A decrease of the CTFs that may be generated by the sequential proteolysis of Ryk such as Ryk-CTF and Ryk-ICD [33] was also apparent, however to a lesser extent (Figure 6D). Given that Ryk-ICD may result from PS1 cleavage, as previously investigated in PS1-deficients cells [16], these results suggested that Ryk toxicity may be triggered by the production of Ryk-ICD in mutant htt cells, which led us to analyze endogenous Ryk-ICD levels in these cells.

### Ryk-ICD Is Increased in the Nucleus of Mutant htt Striatal Cells

**Figure 7:**
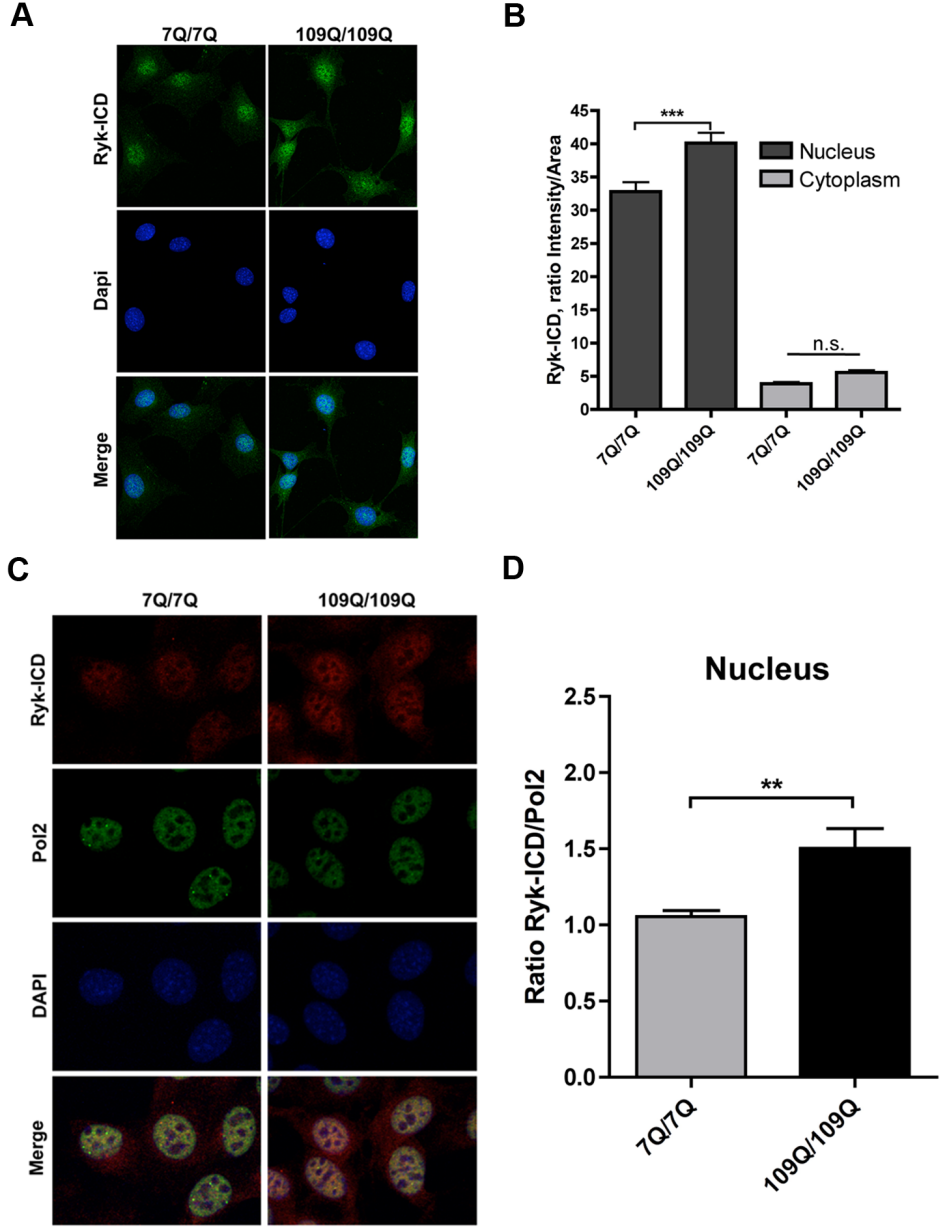
The ICD of Ryk is increased in the nucleus of mutant htt striatal cells. (A) Representative confocal microscopy images showing the pattern of Ryk-ICD immunoreactivity in normal htt (7Q/7Q) and mutant htt (109Q/109Q) striatal cells under normal culture conditions (no serum starvation) as detected using the rabbit polyclonal antibody anti-Ryk^ICD^. (B) Quantification of Ryk-ICD immunoreactivity in mouse striatal cells. 7Q/7Q and 109Q/109Q cells were grown on the same slides. Comparisons were performed for cells with a nucleus size in the range of 150–250 pixels. Ryk-ICD immunoreactivity was increased in the nucleus of 109Q/109Q cells compared to 7Q/7Q cells. Data are mean ± SEM for the ratio Intensity/Area as detected in either nucleus or cytoplasm (*n* = 3 for a total of at least 100 cells analyzed), ***p<0.0001 compared to normal htt cells; n.s., not significant. Significance was tested using one-way ANOVA, with correction for multiple testing by Tukey's Multiple Comparison Test. Ryk siRNA treatments were observed to reduce nuclear Ryk-ICD immunoreactivity in 109Q/109Q cells (see Figure S6). (C) Representative confocal microscopy images showing the pattern of Ryk-ICD immunoreactivity in normal htt (7Q/7Q) and mutant htt (109Q/109Q) striatal cells under normal culture conditions (no serum starvation) as detected using the rabbit polyclonal antibody anti-Ryk^ICD^ and mouse Pol2 antibody 7C2. (D) Quantification of Ryk-ICD and Pol2 immunoreactivity in mouse striatal cells. 7Q/7Q and 109Q/109Q cells were grown on the same slides. Comparisons were performed for cells with a nucleus size in the range of 150–250 pixels. The ratio for Ryk-ICD/Pol2 immunoreactivity was increased in the nucleus of 109Q/109Q cells compared to 7Q/7Q cells. Data are mean ± SEM for the ratio Intensity Ryk-ICD/Intensity Pol2 as detected in the nucleus (*n* = 4 for a total of at least 100 cells analyzed), **p<0.002 compared to normal htt cells. Significance was tested using Welch's *t* test.

The model supported by our data for Ryk to be toxic in HD suggests that Ryk-ICD levels might be increased in mutant polyQ cells. To explore this possibility, we used a newly obtained rabbit polyclonal Ryk-ICD antibody (anti-Ryk^ICD^) [33] to perform immunocytostaining of mouse striatal cells followed by confocal analysis. This antibody was raised against the Ryk-ICD fragment, and it recognizes Ryk species that contain the ICD including full-length Ryk and the Ryk-ICD fragment [33]. Depending on the type of cells and level of expression in these cells, this antibody may detect one or more of all possible Ryk species. The detection of Ryk species is also dependent on the methods used for expression analysis (see Western blot in Figure S6) and likely to be dependent on epitope accessibility. Immunocytostaining of mouse striatal cells indicated that Ryk-ICD immunoreactivity was primarily localized in the nucleus, with little signal detected in the cytoplasm and no signal detected at the membrane (Figure 7A/B). Additionally, the intensity of nuclear staining was decreased if Ryk expression is reduced (Figure S7). These results suggested that full-length Ryk might be rapidly processed to produce intracellular fragments that localize to the nucleus. Interestingly, nuclear Ryk-ICD levels appeared to be increased by about 25% in mutant htt cells compared to normal htt cells, with no change detected in the cytoplasm (Figure 7A/B). This effect was observed for normal and mutant htt cells cultured on the same slides and for cell nuclei having similar sizes between genotypes. We further investigated the possibility that nuclear Ryk-ICD levels may be increased by using an internal control for improved comparison of Ryk-ICD levels between normal and mutant htt cell nuclei. We elected to use Pol2 immunostaining as a second antigen, as we observed that Pol2 levels are similar between normal and mutant htt cells. Nuclear Ryk-ICD levels were increased by about 40% in mutant htt cells in these experiments (Figure 7C/D). Collectively, these observations suggested that nuclear Ryk-ICD levels may be significantly increased in mutant htt cells, corroborating the *C. elegans* and striatal cell data on the toxic effects of Ryk-ICD overexpression (see Figures 5 and 6) and further supporting a model in which the ICD of Ryk triggers the detrimental effects of Ryk increase in mutant polyQ cells.

### Striatal Ryk Expression Profiles in 140CAG Knock-in Mice and HD Patients

In 128Q nematodes, *lin-18*/Ryk is up-regulated at an early stage of touch receptor neuron genesis (see Figure S4, Table S5)—that is, before the onset of pathology (loss of touch response) as detected in late larvae/young adults [7]. This led us to explore whether Ryk might be increased before or at an early stage of pathology in HD mice. Although there are a number of fragment and full-length genetic mouse models of HD, each possessing useful experimental outcomes that can be used to provide a greater understanding of the human disease process, full-length knock-in HD mice such as 140CAG mice [24] may provide the best possible genetic comparability to human HD. Western blot analysis of protein extracts from the striatum of 140CAG mice using a N-terminal Ryk antibody showed that Ryk can be detected as two bands, one band corresponding to full-length Ryk and a weaker band of ∼28 kDa (Figure S8A). The weaker band likely corresponds to an extracellular fragment of Ryk that is generated by proteolytic cleavage near the transmembrane domain, illustrating again (see Figure 4D) the possibility that Ryk may be cleaved by several proteases [33]. Densitometric analysis of Ryk showed an increase of immunofluorescence activity in the neostriatum of 140CAG HD mice at 8 mo of age (Figure S8B). To explore the chronological features of Ryk increase in the striatum of 140CAG mice, we performed immunohistochemical analysis and observed a significant age-dependent increase of Ryk levels at 2, 4, 6, and 8 mo of age (Figure S8C). Because running wheel and sensorimotor deficits may begin at 4 mo in the 140CAG HD mice with climbing deficits detected at 1.5 mo [24], this observation suggested that Ryk might be increased prior to the onset or during the early phases of motor decline in these mice.

We next explored striatal Ryk expression in HD brains. Human HD brain tissue specimens were age-matched and postmortem interval-matched. Densitometric analysis of Ryk showed a significant increase of immunofluorescence in grade 1/2 HD patients (Figure S9A). In contrast to large cholinergic neurons and medium-sized NADPH-diaphorase aspiny neurons, medium-sized spiny GABAergic projection neurons of the HD striatum are affected early and most severely [37]. To further explore Ryk immunoreactivity within the striatum of HD patients, we performed double immunofluorescence for Ryk in combination with either calbindin or NOS immunoreactivities, labeling degenerating and relatively unaffected striatal neurons, respectively. Ryk co-localized with calbindin-positive striatal neurons, with no cross-reactivity with spared NOS-positive neurons (Figure S9B), suggesting that Ryk immunoreactivity might correlate with the selective neuronal loss observed in HD. Additionally, we tested whether Ryk immunoreactivity might correlate with HD severity. Little positive Ryk immunoreactivity was present in the control specimens, with marked expression of Ryk immunoreactivity in all HD tissue samples in neurons, neuropil, and other cell types with increased degrees of neuropathological severity (Figure S9C). Densitometric analysis showed grade-dependent increases in Ryk immunoreactivity (Figure S9C). Finally, we used combined Ryk GFAP immunofluorescence to test whether Ryk might be increased in cells other than neurons for the most severe HD grade (grade 4), in which few medium spiny neurons are left in the caudate nucleus. Compared with age-matched normal specimens, Ryk immunofluorescence was increased within astroglia from caudate nucleus grade 4 HD, but not on a one-to-one basis (Figure S10). Although Ryk immunofluorescence signals were observed in the GFAP-negative area, and although there were glial figures without co-localized Ryk, Ryk immunoreactivity appeared to be predominantly from astrocytes (Figure S10), a possibility also suggested by the densitometric analysis of Ryk immunoreactivity in mouse striata (Figure S8B).

Collectively, these results suggested that Ryk may have discrete patterns of increased expression in striata and caudate nuclei, an aspect that will the subject of future studies that include additional Ryk antibodies and brain samples.

## Discussion

Wnt signaling regulates several developmental processes, including synaptic differentiation, as well as adult neurogenesis [11]. The activation of canonical and noncanonical Wnt pathways may be neuroprotective in neurodegenerative disease as indicated by studies of Aβ oligomer toxicity [11]. Studies of polyglutamine expansion [7] and Aβ toxicity [8] indicates that the same applies to the activation of the FOXO pathway, a pathway that is central to cell survival/longevity [38] as well as neuronal cell homeostasis [39]. Here, we found that Ryk, a Wnt receptor important to axon guidance [40],[41], is increased in mutant polyQ neurons and represses FOXO neuroprotective activity, highlighting a pathological situation in which the Wnt and FOXO pathways interact to promote neuron dysfunction and degeneration. The Ryk protein is involved in canonical Wnt to regulate neurite outgrowth, being required for TCF-driven transcription through binding to Frizzled and Dishevelled [26]. This raised the possibility that Ryk might repress FOXO activity via the alteration of the canonical Wnt/Fzd–β-catenin pathway. However, Wnt ligands such as *cwn-1*/WNT4, *cwn-2/*WNT5, and *egl-20/*WNT16 and the transcription factor *pop-1*/TCF did not appear to modulate 128Q neurotoxicity in *C. elegans*, suggesting that canonical Ryk signaling may not contribute to the toxicity of Ryk in mutant polyQ neurons. Besides its ability to signal through the planar cell polarity pathway [19],[20], the Ryk protein can also signal through another noncanonical pathway, namely the nuclear translocation of its ICD (Ryk-ICD), a γ-secretase cleavage product that is required for cortical neurogenesis [16]. Here, we provide a model in which the repression of FOXO by Ryk in mutant polyQ neurons may primarily occur through the nuclear translocation of the Ryk-ICD fragment. Our data noticeably indicate that (*i*) the free Ryk-ICD fragment may be sufficient to repress FOXO activity in mutant polyQ cells, (*ii*) the *lin-18*/Ryk ICD fragment is sufficient to suppress the protective effects of *lin-18* knock-out on the function of polyQ-expanded nematode neurons, and (*iii*) the free Ryk-ICD fragment may bind to β-catenin, and there is a functional cross-talk between Ryk-ICD and β-catenin in the modulation of Ryk-ICD cytotoxicity. These data support the possibility that abnormally high amounts of Ryk-ICD may prevent the activity of β-catenin, a survival protein [9],[14],[42] that promotes FOXO transcriptional activity [9] and protects against the early phases of mutant polyQ cytotoxicity [10]. This model is further supported by our data linking Ryk and γ-secretase PS1 in the modulation of mutant htt striatal cell viability, and by our data on the increase of endogenous Ryk-ICD levels in the nucleus of these cells. Collectively, these observations point to the Ryk-ICD fragment as an important mediator of the detrimental effects of Ryk increase. The γ-secretase complex is involved in the role of Ryk in cortical neurogenesis [16] and has been previously implicated in HD through its effect on HTT proteolysis and production of N-terminal HTT species [36]. Our data suggest that γ-secretases may also have a role in the regulation of cell stress response in HD by mediating the detrimental effects of Ryk increase on FOXO3a activity. Here, it is noticeable that *aph-1*, the *C. elegans* homologue of APH-1B [43], may be up-regulated by expanded polyQs, as suggested by our microarray data from touch receptor cells, which might further contribute to the toxicity of the Ryk-ICD pathway in mutant polyQ cells. Future research will build on these observations to further investigate the mechanisms that may underlie the pathological role of the Ryk-ICD/FOXO3a pathway in HD. Besides testing whether Ryk cytotoxicity might be modulated by mammalian Wnt ligands or Ryk inhibitory antibodies [33] and studying whether an excess of the Ryk-ICD fragment might tether β-catenin away from FOXO, it will be interesting to identify the FOXO3a transcriptional targets that may be deregulated by Ryk in mutant polyQ neurons and understand how this may impact brain longevity mechanisms as the pathogenic process develops in HD. The type and activity of FOXO target genes may greatly depend on the cellular context in which FOXO operates [4],[44], which may also be true in HD, as suggested by the study of context dependence in FOXO interaction networks across mouse and human datasets [25]. Interestingly, the transcriptomic signature of 128Q nematode touch receptor cells contains 84 genes that were previously identified as putative *daf-16*/FOXO targets in *C. elegans*, including 31 genes that are highly conserved in mice (Table S7), suggesting that FOXO target genes may be altered by mutant polyQ expression. The deregulation status of most of these 31 genes appeared to be greatly context-dependent, as evaluated by using entropy-based feature selection across 14 HD-associated conditions including murine striatum and human (postmortem brains, blood samples, induced pluripotent stem cells) datasets (Table S7) [5], which emphasizes the importance of context (e.g., cell type, time requirement) in future analyses of the physiological impact of abnormal Ryk signaling on FOXO activity in HD.

*Lin-18*/Ryk is up-regulated in 128Q nematode touch cells before the loss of touch response—that is, prior to pathology in *C. elegans*. In the neostriatum of 140CAG mice, Ryk increase is first detected during the early phases of pathology in these mice, which further links increased expression of Ryk with the early phases of HD pathology. The alteration of Ryk may also be associated with the human disease, as suggested by the grade-dependent increase of Ryk immunoreactivity in the human HD caudate nucleus. However, although the increase of Ryk detected in the neostriatum of 140CAG mice is unlikely to result from abnormal Ryk degradation or age-dependent accumulation of Ryk as it was observed in young mice, Ryk immunoreactivity in postmortem HD brains might be influenced by several factors such as alteration of cyto-architectural structure and disease-unrelated factors. Similarly to other membrane proteins, Ryk might be processed by several proteases that cleave Ryk in the vicinity of its transmembrane domain [33], which might generate Ryk C-terminal and N-terminal fragments with distinct localization and half-life in brain tissues. Selective Ryk antibodies such as inhibitory anti-Ryk antibody RWD1 were recently described [33], and these antibodies will be useful in additional studies of Ryk expression in the HD mouse and human HD brain.

The Ryk protein is solely represented in several genomes, and reducing its pathological levels is anticipated to ameliorate neurological disease, which makes this receptor an attractive candidate for therapeutic intervention. Ryk is acutely induced in models of CNS injury and in concert with Wnt signaling proteins inhibits axon regeneration [45],[46], suggesting that Ryk inhibition may promote axon regeneration upon injury. Our findings identify the inhibition of Ryk-ICD signaling as a therapeutic strategy to restore cell stress response and neuronal function in HD and perhaps in other neurodegenerative diseases. The up-regulation of Ryk prior to pathology in 128Q nematodes, the up-regulation of Ryk at the mRNA and protein levels in mutant htt striatal cells derived from HdhQ111 mice, and the converging evidence for nuclear Ryk-ICD to mediate Ryk neurotoxicity by altering FOXO activity suggest that the inhibition of the Ryk-ICD pathway may have therapeutic potential in HD.

In summary, our study reveals that Ryk activity is altered in mutant polyQ neurons, with Ryk up-regulation promoting neuronal dysfunction via the early-stage repression of FOXO neuroprotective activity. Regardless of a possible contribution from other mechanisms, our findings support a model in which the ICD of Ryk, a γ-secretase cleavage product, may be important for the neurotoxic action of Ryk increase by altering the protective activity of the β-catenin/FOXO complex. These data highlight a pathological process in which neurodevelopmental pathways may be altered during the early stages of the pathogenic process in neurodegenerative disease to repress cell stress response and “neuronal longevity” mechanisms such as those controlled by FOXO and its co-factors. Importantly, our data reveal that neurons may be unable to develop an efficient FOXO-mediated survival response against the earliest stages of mutant HTT toxicity. This suggests that the early-stage restoration of neuronal resistance capacity through the stimulation of cell-stress response networks and mechanisms that are under FOXO control might efficiently delay the pathogenic process in HD, which may have significant implications for the prioritization of disease-modifying strategies and identification of disease modifiers.

## Materials and Methods

### Ethics Statement

All the animal experiments were performed in accordance with the Guide for the Care and Use of Laboratory Animals and were approved by the Institutional Animal Care and Use Committee at the University of Pittsburgh. Work involving human brain tissue samples was approved by the institutional review board and the Committee for Oversight of Research Involving the Dead at the University of Pittsburgh.

### *C. elegans* Assays

Nematode strains were handled using standard methods [47]. The integrated polyQ strains used in this study were previously described [15],[48] and all assays were performed blindly. Some nematode strains used in this work were provided by the Caenorhabditis Genetics Center, which is funded by the National Institutes of Health National Center for Research Resources. The complete list of strains used in this study is shown in Table S8. Constructs encoding LIN-18 were generated as follows. The *lin-18* cDNA was obtained from wild-type animals by RT-PCR, using lin-18_attB5 (5′-GGGGACAACTTTGTATACAAAAGTTGATGTTCATCAGCAAAGAGGA) and lin-18_attB2 (5′-GGGGACCACTTTGTACAAGAAAGCTGGGTATTAGATGTATTGACTGAGT) primers. These primers contain, respectively, attB5 and attB2 sequences for recombination in the pDONR221-P5-P2 vector, using the Gateway system (Invitrogen). In parallel, we produced a clone, in pDONR221-P1-P5r, containing the promoter of *mec-3*, *mec-3p*, using primers RV3 (5′-GGGGACAAGTTTGTACAAAAAAGCAGGCTCCTGCAGGTACCCGGAGTAGTTG) and RV4 (5′-GGGGACAACTTTTGTATACAAAGTTGTGGCGCGCCAATGCGCGAAATTGTG GCTACTC). Both clones were used to assemble *mec-3p* and *lin-18*, using Gateway technology, in the destination vector pDEST-AN [49], which is suitable for *C. elegans* transgenesis. To produce the construct coding for the LIN-18 ICD, we used the same strategy. Briefly, we used as a reverse primer lin-18_attB2, and a forward primer located after the sequence that encodes LIN-18 ICD, RV61 (5′-GGGGACAACTTTGTATACAAAAGTTGAAAATGTTCAAGCGCTCTAAAAAAGAAGA). Constructs were verified for sequence integrity and were then injected at 4–40 ng/µl in 19Q;*lin-18(e620)* and 128Q;*lin-18(e620)* nematodes, together with pPD118.33 (a plasmid containing *myo-2p::GFP*) at 10 ng/µl as a marker to follow transgenesis and pUC18 as a DNA carrier to a final total DNA concentration of 100–150 ng/µl, using standard methods. We isolated at least two independent strains from each construct based on GFP expression in the pharynx to perform touch assays and axonal swelling assays. Touch response assays were carried out as described [7]. Touch tests involved scoring for the response to light touch at the tail by using a fine hair. Touch test were performed by scoring 10 touches at the tail of the animal for a minimum of 200 animals per genotype. Ordinarily, wild-type animals will respond by backing away from the touch. The responses were recorded for every animal such that, for example, 3 responses out of 10 at the tail is given as 30% responsiveness, and the mean values for responsiveness were retained for comparison of nematode groups. Touch tests were performed by two groups of two experimenters, and maximum baseline variation was 9%. Axonal swelling was scored as previously described [7]. Briefly, 128Q nematodes were mounted on agar pads and immobilized using levamisole 20 mM, prior to examination on a 40× objective of a Leica Axioplan microscope, equipped with fluorescence. A total of more than 150 animals per strain were examined for axonal swelling in PLM neurons. Animals containing at least one swollen axon were scored as positive. For strains expressing extrachromosomal arrays, only animals expressing the transgenic marker (i.e., GFP expressed in the pharynx under the control of *myo-2p*) were assayed. Extraction of protein from whole worms and Western blotting was conducted using standard methods [50] and the following primary antibodies: GFP antiserum (Abcam, 1∶5,000) and actin antibody (Molecular Probes, 1∶5,000). Secondary antibodies used were as follows: goat–anti-rabbit IgG HRP-conjugated (Abcam, 1∶10,000) and goat–anti-mouse IgG HRP-conjugated (Biorad, 1∶10,000). Proteins were detected by using ECL+ (ECL for actin) and evaluated by densitometry. Unpaired *t* tests were used for statistics.

### Primary Cultures of Embryonic Nematode Cells

Embryonic cells were obtained as previously described [51]. Briefly, embryos were isolated from gravid adults following lysis in a hypochlorite solution. Eggshells were removed by incubation in 0.5 ml chitinase/chymotrypsine (1 U/ml and 3,000 U/ml, respectively, in egg buffer) for 20 min. Following resuspension in egg buffer, the embryos were dissociated by 0.25% trypsin treatment for 5 min and resuspended in L-15 supplemented with antibiotics and 20% FBS (L15-CM). Cells were plated on TESPA (4%, Sigma) coated glass plates at a density of ∼300,000 cells/cm^2^ and maintained in L15-CM media. Cells were incubated at 20°C overnight. Wild-type (N2) cells were isolated and treated similarly.

### Microarray Experiments and Data Analysis

The materials and methods used for FACS sorting, RNA extraction, microarray analysis, microarray data analysis, and RT-PCR are described in Text S1.

### Striatal Cell Mortality Assays

We used striatal cells homozygous for normal (7Q/7Q) or mutant (109Q/109Q) htt derived from HdhQ111 knock-in mice [23] and handled them as previously described [30]. Low-passage (P9–P11) cell lines were used in all experiments. We used jetPEI-FluoR for transfection with cDNA, jetSI-ENDO for siRNA assays, and JetPrime for co-transfection with siRNA and cDNA as indicated by the manufacturer (PolyPlus Transfection). The siRNAs (si-Ryk, si-Foxo3a) and scramble RNAs were obtained from QIAGEN and Operon, respectively. Mixes of 3–4 different siRNA sequences per gene (each sequence at 25 or 33 nM) were systematically tested for modulation of cell survival and target gene expression, followed by the evaluation of individual siRNA sequences at optimal concentration (100 nM). Effects on cell survival were considered to be reliable if two different siRNAs showed similar effects on target expression and cell survival and if the scramble RNAs (100 nM; unique sequence that does match with any sequence in the mouse genome) did not show any effect. The active siRNA sequences shown in the figures are as follows: si-Ryk, 5′-GCAAATTAGTAGAAGCCAA-3′ (100 nM); si-Foxo3a, 5′-GCTTCATGCGCGTTCAGAA-3′ (100 nM); siPS1, 5′-GGAGCATTCTAACGAGTGA-3′ (100 nM); and siPS2, 5′-CTATCAAGTCTGTGCGTTT-3′ (100 nM). The corresponding scramble RNAs shown in the figures are as follows: Ryk, 5′-GACGAAAGACCTATAATGA-3′ (100 nM); Foxo3a, 5′-GAGCTGTACCGATGACCTT-3′ (100 nM); PS1, 5′-ACGTAGTCAATTCGGAGAG-3′ (100 nM); and PS2, 5′-GGTATTCCGTATTCGTCTA-3′ (100 nM). For transfection with cDNA constructs, we used 1 µg pcDNA3.1-FOXO3a-HA (a gift from Lisa Ellerby), pcDNA3.1/nV5-DEST-β-catenin (Addgene), or pcDNA3.1-Myc-Ryk ICD. The Ryk-ICD fragment (amino acids 239 to 595) was amplified from mouse cell RNAs by RT-PCR using the Ryk-ICD att-B1 5′-GGGGACCACTTTGTACAAGAAAGCTGGGTATCAGACTGAGGCTCCCAGGGCAG-3′ and Ryk-ICD att-B2 5′-GGGGACAAGTTTGTACAAAAAAGCAGGCTGCCGCCACCATG*GAACAAAAACTTATTTCTGAAGAAGATCTG*AAAAGGATTGAACTGGATGACG-3′ primers. The reverse primer contained a sequence (italicized) coding for a Myc tag. PCR products were subcloned into the pDONR221 vector and then into the final destination vector pcDNA3.1 using Gateway technology and verified for sequence integrity.

Cell mortality assays were performed as described previously by counting picnotic nuclei [52] or measuring caspase 3/7 activity [53]. Briefly, low passage number (P9–P12) 7Q/7Q and 109Q/109Q cells were subjected to a 24-h serum deprivation 48 h after cell transfection. Cells were then fixed and subjected to DAPI staining, and cell mortality was scored by counting picnotic versus normal nuclei in DAPI- and jetPEI-FluoR–, or JetSI-ENDO–positive cells. Alternatively, caspase assays were used and transfected cells were plated on collagen-coated 96-well plates for 48 h. After 24-h serum starvation, the activation of caspase 3/7 was measured in cells using the Apo 3/7 HTS High Throughput Screen Assay kit (Cell Technology). The activity of caspase 3/7 was measured using a Tecan infinite F500 microplate reader, with excitation and emission wavelengths of 485 and 535 nm, respectively. Caspase assays were performed using six replicates per point and data expressed as dRFU/min/mg of protein.

For qRT-PCR analysis, we used the forward 5′-TGAGAGCTGACACACCCAA and reverse 5′-CACTTCGCAAGTCGTTCTTC primers for amplification of Ryk mRNAs and forward 5′-TTTGCCGCGAGCCG and reverse 5′-TAACCTGGTTCATCATCGCTAATC primers for amplification of HPRT mRNAs as a control. For Western blotting, proteins were extracted as previously described [30], separated by SDS-PAGE, and analyzed by Western blotting using the following primary antibodies: mouse anti-HTT (4C8, Chemicon,1∶5,000), rabbit anti-RYK (Abgent, 1∶100), mouse anti-FOXO3a (Cell Signaling, 1∶1,000), rabbit anti-PS1 and rabbit anti-PS2 (Cell Signaling, 1∶1,000), rabbit anti-Myc tag (Cell Signaling, 1∶1,000), mouse anti-V5 tag (InVitrogen, 1∶2,000), and mouse anti-actin (MP Biomedicals, 1∶5,000). Secondary antibodies used were as follows: goat–anti-mouse IgG HRP-conjugated (Biorad, 1∶10,000) and goat–anti-rabbit IgG HRP-conjugated (Biorad, 1∶10,000). Proteins were detected by using ECL+ (ECL for actin) and evaluated by densitometry. Statistical analyses were performed using unpaired *t* tests.

### Immunoprecipitation Assays

To test for interaction between Ryk and β-catenin, transfection of 293T cells with wild-type Myc-tagged Ryk, Flag-tagged RYK ICD, or an uncleavable Ryk mutant (Ryk, EGFRRc) [16] was performed using a calcium phosphate precipitation method [54]. Cells were lysed in a lysis buffer containing 25 mM Tris-HCl, pH 7.4, 150 mM NaCl, 5 mM EDTA, 1% Triton X-100, 10 mM sodium pyrophosphate, 10 mM β-glycerophosphate, 1 mM sodium orthovanadate, 10% glycerol, and protease inhibitors (Roche). For immunoprecipitation, cell lysates were incubated with a specific antibody for 2 h at 4°C and then with Protein A/G agarose beads (Pierce) overnight. Immunoprecipitates were eluted using SDS sample buffer and separated using 8% or 10% SDS-PAGE. After blocking, the blots were incubated with a primary antibody and then with a peroxidase-conjugated secondary antibody. The bound secondary antibody was then detected using enhanced chemiluminescence (ECL) reagent (Santa Cruz Biotechnology). To test for an interaction between Ryk ICD and FOXO3a, mouse striatal cells were transfected with Foxo3a-HA and Myc-tagged Ryk-ICD using Jet PEI (PolyPlus Transfection) as described by the manufacturer. Cells were lysed in PBS 0.1% tween-20 supplemented with PMSF and protease inhibitors (Roche). For immunoprecipitation, cell lysates were incubated with a specific antibody for 2 h at 4°C and then with fastflow Protein A agarose beads (Sigma) overnight at 4°C. Immunoprecipitates were eluted using SDS sample buffer and separated using 4%–12% SDS-PAGE (InVitrogen). The Ryk-ICD fragment (amino acids 239 to 595) construct used in these experiments was the same as above (see striatal cell vulnerability assays).

### Luciferase Reporter Assays

Normal htt mouse striatal cells derived from HdhQ111 mice and used at low passage numbers (P9–P12) were seeded in 96-well plates, at a density of 25,000 cells/well, and were co-transfected using Amaxa technology with 0.5 µg of the plasmid(s) pcDNA3.1-Foxo3a-HA (a gift from Lisa Ellerby) together with pcDNA3.1-Myc-Ryk (see below), pcDNA3.1-Myc-Ryk ICD (see above), pcDNA3.1-Myc-uncleavable-Ryk (see above), 100 nM of β-catenin siRNAs (QIAGEN) or scramble RNAs (Operon), 0.5 µg of the luciferase reporter (FHRE-luc, Addgene) [55], which contains three canonical FOXO binding sites, and 50 ng of the Renilla luciferase construct (Promega). The pcDNA3.1-Myc-Ryk construct was generated as follows: the Myc-Ryk coding sequence was amplified from pCMV6-Myc-Ryk (Origene) by using the primers forward 5′-GGGGACAAGTTTGTACAAAAAAGCAGGCTGCCGCCACCATGCGCGCGGGCCGGGGC and reverse 5′-GGGGACCACTTTGTACAAGAAAGCTGGGTATCAGACGTAGGCTCCCAGGGCAG and subcloned into pcDNA3.1, and the resulting plasmid was verified for sequence integrity. The pmaxGFP construct (Amaxa) was used as a control for cotransfection. Transfection efficiency was greater than 80%. One day after transfection, cells were lysed in 20 µl of lysis buffer and the luciferase and Renilla luciferase activities assayed using Stop & Glo reagents (Promega) according to the manufacturer's protocol. For the β-catenin siRNA experiments, effects were considered to be reliable if two different siRNAs showed similar effects on β-catenin expression and luciferase activity and if a scramble RNA did not show any effect. The active β-catenin siRNA and the scramble RNA that are shown in the figures are 5′-GATAGAAATGGTCCGATTA-3′ and 5′-GTGTGAATGCATGAAACTA-3′, respectively.

### Confocal Analysis of Ryk ICD Signals

Endogenous Ryk-ICD levels were analyzed in 7Q/7Q and 109/109Q mouse striatal cells [23] by using the rabbit Ryk-ICD antibody anti-Ryk^ICD^ [33]. Low-passage (P9–P11) cell lines were grown in normal conditions (no serum starvation) and treated with Ryk siRNAs or scramble RNAs as described above (see striatal cell mortality assays) using Lab-Tek eight-chamber glass slides (BD Biosciences). For comparison of Ryk-ICD levels between 7Q/7Q and 109/109Q cells, cells were grown on the same slides. Cells were fixed and permeabilized using the Cytofix/Cytoperm Kit (BD Biosciences) following the manufacturer's protocol. Cells were then incubated with anti-Ryk^ICD^ (1∶100) followed by incubation with the anti-rabbit secondary antibody Alexa Fluor 488 (Invitrogen, 1∶500) and subjected to diamidino-2-phenylindole (DAPI) staining. Alternatively, cells were co-incubated with anti-Ryk^ICD^ (1∶100) and the Pol2 antibody 7C2 (1∶500) followed by co-incubation with the anti-rabbit secondary antibody Alexa Fluor 555 (Invitrogen, 1∶500) and the anti-mouse secondary antibody Alexa Fluor 488 (1∶500), and cells were then subjected to DAPI staining. Fluorescent signals were quantified using a confocal microscope (Leica TCS SP5) and images analyzed using ImageJ. For each of the focal planes, anti-Ryk^ICD^ signals were quantified from the nucleus (DAPI staining). The comparison of 7Q/7Q and 109/109Q cells was based on cell nuclei that have a size of 150–250 pixels in each of the cell lines. To assess cytoplasmic expression, nuclear anti-Ryk^ICD^ signals were blackened, and the remaining signals were quantified. Cytoplasmic analysis was performed only if nuclear anti-Ryk^ICD^ signals were detected in the same confocal plane and cell.

### Brain Tissue Specimens

#### Mouse

Male full-length knock-in mice heterozygous for the 140CAG repeat mutation were obtained from an established colony at the Bedford VA Medical Center and were backcrossed with B6CBA females from Jackson Laboratory (Bar Harbor, ME). The offspring were genotyped using a PCR assay on tail DNA. Disease expression is present in these mice by 8 mo of age [56] Groups (*n* = 10) of 1-, 2-, 4-, or 8-mo-old mutant and littermate wild-type control mice were deeply anesthetized and transcardially perfused with 2% buffered paraformaldehyde (100 ml), with care to avoid the introduction of any perfusion artifact. Brains were removed, cryoprotected, and serially sectioned (50 µm). Serial cut mouse tissue sections were subsequently immunostained for RYK immunofluorescence.

#### Human

Postmortem striatal tissue specimens from 15 adult-onset HD patients (two Grade 1, four Grade 2, four Grade 3, and five Grade 4 cases; mean age of death, 65.3 y; range, 54–72 y) and six age-matched patients without any known neurological sequella (mean age, 67.2 y; range, 60–78 y) were dissected fresh and placed in cold (4°C) 2% paraformaldehyde–lysine–periodate solution for 24–36 h. Brain tissue specimens were received from the Bedford VA Medical Center Brain Tissue Archive. The postmortem intervals did not exceed 15 h (mean time, 10.7 h; range, 4–15 h) and were similar for controls and HD patients. CAG repeat length analysis was performed on the HD specimens (mean number of CAG repeats, 44.6). The range of CAG repeats in the adult-onset HD patients was 41–49. Each HD patient had been clinically diagnosed based on known family history and phenotypic symptoms of HD. The diagnosis of HD was confirmed by neuropathological examination and graded by severity [57] Striatal tissue blocks were rinsed in 0.1 M sodium phosphate buffer and placed in cold cryoprotectant in increasing concentrations of 10% and 20% glycerol, 2% DMSO solution for 24–36 h. Frozen serial sections of the striatal tissue blocks from the anterior commissure to the rostral extent of the globus pallidus were cut at 50-mm intervals in the coronal plane and placed within a six-well collection container. The cut sections were stored in 0.1 M sodium phosphate buffer and 0.08% sodium azide at 4°C for subsequent Nissl and immunohistochemical techniques. The cut sections were stored in 0.1 M sodium phosphate buffer with 0.08% sodium azide at 4°C for subsequent immunocytochemistry, immunofluorescence, and combined immunofluorescence methods for tyrosine-protein kinase RYK precursor (RYK), calbindin, and nitric oxide synthase (NOS) antibodies. Tissue sections were examined using a Nikon Eclipse E800 microscope with a Spot RT digital camera.

### Immunocytochemistry and Fluorescent Immunocytochemistry

The methods used for immunocytochemistry and fluorescent immunocytochemistry analyses of mouse tissue samples and human tissue specimens are described in Text S2.

### Western Blot Image Processing

Western blot membranes were exposed to films. After exposure, films were scanned at high resolution (1,200 dpi) using a regular scanner (Epson), and scans (tiff files) were opened into Photoshop and Western blot images processed using PowerPoint and Photoshop (Microsoft) for constructing final figures. Alternatively, Western blot membranes were scanned at high resolution (692 dpi) using a luminescent image analyzer (FUJIFILM LAS-4000), and scans (tiff files) were opened into ImageJ and Western blot images processed as previously mentioned for constructing final figures.

### Statistical Analysis

Statistical analysis of variance (ANOVA), *t* tests, and Welch's *t* tests were performed using Prism. One-way ANOVA was followed by correction for multiple testing by Tukey's Multiple Comparison Test. The statistical analysis of microarray data and statistical methods used for biological annotations are described in Text S2.

## Supporting Information

Figure S1 Gene Ontology classification of genes specifically deregulated by expanded polyQ expression in nematode neurons (P*mec-3* targets). Genes were classified based on their functional annotations in the GO categories “Biological Process,” “Molecular Function,” and “Cellular Component.” The number of genes is indicated.
(TIF)

Figure S2 Stable Fourier analysis modules containing genes up-regulated in 128Q nematode neurons (P*mec-3* targets). The genes up-regulated (FDR <0.01) in nematode neurons are indicated by green nodes. Node borders in purple and blue indicate down- and up-regulation (*p*<0.01) of the human best ortholog in the caudate nucleus of HD patients, respectively, as previously reported [29]. Square nodes indicate druggable genes [28]. The enrichment in GO annotations was generated using Gostat and enrichments considered significant for *p*<0.001. Four modules described the extracellular matrix (Nos. 1–3, No. 5), three modules membrane proteins (Nos. 4–6), two modules the cytoskeleton (Nos. 7–8), nine modules the mitochondria (Nos. 9–13, 22–23, 26, 37), one module the golgi apparatus (No. 14), and five modules the nucleus (Nos. 15–20), with other modules corresponding to carbohydrate metabolism (Nos. 21–24), lipid metabolism (Nos. 25–27), chaperones (Nos. 37–39), and general metabolism (Nos. 28–36). Of note, module 40 contained components involved in signaling pathways (Wnt and TGF-β), axonal guidance, and cell cycle.
(PDF)

Figure S3 Stable Fourier analysis modules containing genes down-regulated in 128Q nematode neurons (P*mec-3* targets). The genes down-regulated (FDR<0.01) in nematode neurons are indicated by red nodes. Node borders in purple and blue indicate down- and up-regulation (*p*<0.01) of the human best ortholog in the caudate nucleus of HD patients, respectively, as previously reported ([29]; see [3]). Square nodes indicate druggable genes [28]. The enrichment in GO annotations was generated using Gostat and enrichments considered significant for *p*<0.001. The modules obtained described extracellular components (Nos. 1–3), membrane (Nos. 4–10), transport (No. 11), nucleus (Nos. 13–15), calcium sensors (No. 21), metabolism (Nos. 16–19), and signal transducers such as neuropeptides and the Ras and hedgehog pathways (Nos. 23–28).
(PDF)

Figure S4 Fourier analysis module containing the Wnt, TGF-β, and nonsense-mediated mRNA decay pathways (up-regulated module 40). Green nodes indicate up-regulated nematode genes with FDR<0.01. Node borders in purple and blue indicate down- and up-regulation, respectively, of human homologs in HD caudate nucleus [29] with *p*<0.01. Square shapes indicate druggable genes [28].
(TIF)

Figure S5 Effects of overexpressing LIN-18 ICD at 40 ng/µl in polyQ;*lin-18* nematodes. Overexpressing LIN-18 ICD cDNA at 40 ng/µl in touch receptor neurons using the *mec-3* promoter abolishes the neuroprotective activity of *lin-18* LOF in 128Q nematodes. This effect showed a trend toward exacerbation of 128Q cytotoxicity but did not reach statistical significance relative to 128Q nematodes. Overexpressing LIN-18 ICD at 40 ng/µl produced cytotoxicity in 19Q nematodes. Two independent *lin-18 ICD* extrachromosomal arrays (A1, A2) were tested per polyQ genotype. The expression of LIN-18 ICD cDNA was confirmed by RT-PCR for all of the arrays generated. EV, empty vector overexpression. Data are means ± SEM (more than 200 animals tested). *p<0.001 compared to 19Q animals. ns, not significant. Significance was tested using one-way ANOVA, with correction for multiple testing by Tukey's Multiple Comparison Test.
(TIF)

Figure S6 Effects of Ryk siRNA treatment and Ryk-ICD overexpression on the detection of Ryk species by antibody anti-Ryk^ICD^ in protein extracts from 109/109Q mouse striatal cells. Representative Western blot showing that Ryk siRNA treatment reduces the detection of full-length Ryk (Ryk FL), an effect accompanied by decreased Ryk mRNA levels as tested by qRT-PCR (left panel). Although the antibody anti-Ryk^ICD^ does not detect endogeneous levels of Ryk-ICD in these experiments, it detects Ryk-ICD when this Ryk fragment is overexpressed (Ryk-ICD O/E), with no signal detected for empty vector (right panel).
(TIF)

Figure S7 Ryk siRNA treatment decreases nuclear Ryk-ICD immunoreactivity in 109/109Q mouse striatal cells. (A) Representative confocal images showing the pattern of Ryk-ICD immunoreactivity in 109Q/109Q striatal cells upon scramble RNA or Ryk siRNA treatment as detected using the rabbit polyclonal antibody anti-Ryk^ICD^. The Ryk-ICD signals were primarily detected in the nucleus, with weak signals detected in the cytoplasm. (B) Quantification of anti-Ryk^ICD^ nuclear signals in 109Q/109Q striatal cells. Nuclear Ryk-ICD immunoreactivity was decreased by about 25% by Ryk siRNA treatments. Data are mean ± SEM for the ratio Intensity/Area as detected in nuclei (*n* = 3 for a total of at least 90 cells analyzed), ***p<0.0001 compared to scramble RNA. Reduction of Ryk mRNA levels (mean reduction, 54%) was tested by qRT-PCR.
(TIF)

Figure S8 Ryk is increased in the striatum of 140CAG HD mice. (A) Western blot analysis of Ryk expression in striatal protein lysates from 4-wk-old 140CAG homozygous mutant mice (*n* = 3). Using the N-terminal Ryk antibody Ab7577a without or with preincubation with blocking peptide BP7577a, two specific bands were detected, including one band corresponding to the full-length protein and a weaker band of ∼28 kDa likely corresponding to a Ryk extracellular domain fragment (see comments in the Results section). (B) Densitometric analysis showed an increase of Ryk immunofluorescence from the striata of 8-mo-old 140CAG HD mice (lower panel) compared with controls (upper panel) (*n* = 10 per genotype), *p<0.04. Scale bar, 50 µm. Significance was tested using one-way ANOVA. (C) Chronological immunohistochemical analysis of Ryk expression in the striatum of 140CAG mice. Left panels show example images for 140CAG mice at 1, 2, 4, 6, and 8 mo of age (a–e). Scale bar, 100 µm. Right panels show example images for wild-type mice at 1, 2, 4, 6, and 8 mo (a*–e*). 140CAG mice show a significant age-dependent increase of Ryk levels at 2, 4, 6, and 8 mo of age. Data are means ± SD with *n* = 10 in each group. *p<0.001 compared to control. Significance was tested using one-way ANOVA, with correction for multiple testing by Tukey's Multiple Comparison Test.
(TIF)

Figure S9 Ryk is increased in human HD caudate nucleus. Ryk immunofluorescence was assessed using the same Ryk antibody as in Figure S7. (A) Compared with age-matched controls (upper panel), Ryk immunofluorescence was increased in human caudate nucleus of grade 1/2 patients (lower panel). Densitometric analysis (right panel) showed increased Ryk protein levels in HD caudate nucleus (right) compared with controls (left) (*n* = 6 per group), *p<0.02. Scale bar, 50 µm. Significance was tested using one-way ANOVA. (B) Selective Ryk expression in brain striata. Combined immunofluorescence showed that Ryk co-localized (white arrows) with calbindin (degenerating neurons) but not with NOS immunoreactivity (spared neurons) in caudate neurons in brains from HD patients. Some level of co-localization was detected in control brain striata. (C) Densitometric analysis showed a disease grade-dependent increase of RYK immunoreactivity in human HD caudate nucleus. Level of Ryk expression in control (*n* = 5), Grade 1 HD (*n* = 4), Grade 2 HD (*n* = 5), Grade 3 HD (*n* = 5), and Grade 4 HD (*n* = 4) samples. *p<0.05 compared to control; **p<0.02 compared to Grade 1 HD; ***p<0.01 compared to Grade 2 HD; ****p<0.01 compared to Grade 3 HD. Also shown are images of Ryk immunostaining. (a) Immunoexpression of Ryk was low in the brain tissues of age-matched controls. (b–e) Increased Ryk immunoreactivity correlated with the degree of neuropathological severity (b, Control; c, Grade 1; d, Grade 2; e, Grade 3; f, Grade 4). Scale bar, 100 µm. Significance was tested using one-way ANOVA, with correction for multiple testing by Tukey's Multiple Comparison Test.
(TIF)

Figure S10 Partial co-localization of Ryk and GFAP immunofluorescence in Grade 4 HD caudate nucleus. Combined Ryk GFAP immunofluorescence shows co-localization with Ryk and GFAP in both Grade 4 HD (HD G4) striatum and, to a much smaller degree, in normal age-matched (Control) specimens. Compared with age-matched control specimens (a–d), Ryk immunofluorescence was increased within astroglia from human caudate nucleus Grade 4 HD patients (e–f), but not on a one-to-one basis. Several Ryk immunofluorescence signals were observed in the GFAP-negative area (white arrows), and there are multiple glial figures without co-localized Ryk. Ryk immunofluorescence was assessed using the same Ryk antibody as in Figure S8. Scale bar, 20 µm.
(TIF)

Table S1 Genes deregulated in 19Q nematode cells versus control (GFP alone) cells. Forty-one worm genes were found to be deregulated. Most of them were down-regulated (34/41) and about a half of them encoded extracellular and catalytic proteins or were associated to membranes. Gene Ontology (GO) enrichment tests indicated that Pepsin A activity (GO:0004194) was enriched (*p*<0.01). Among these 41 genes, six genes conserved in humans. Also shown are the best homologous genes in humans with their expression levels in the HD brain (caudate and cortex) versus control brains [29]. Human genes deregulated in HD caudate nucleus and cortex are labeled in orange and green, respectively (*p*<0.01).
(PDF)

Table S2 Genes deregulated in 128Q nematode cells versus 19Q nematode cells. We found 2,070 worm genes to be deregulated. Only 18 of the 2,070 genes were also deregulated in 19Q nematode cells versus GFP cells, including *pqn-48*, a lysosomal thiol reductase, and four nematode-specific genes encoding membrane proteins: *srg-64*, *nspb-2*, *nspb-5*, and *str-180*. Also found were nematode genes with no human homologs as inferred from Inparanoid [58], including a lectine (*clec-219*), innexin (*inx-4*), and xylosyltransferase enzyme (*sqv-6*). Among the 2,070 dysregulated genes, 516 genes are conserved in humans. Also shown are the best homologous genes in humans with their expression levels in HD brains (caudate and cortex) versus control brains [29]. Human genes deregulated in HD caudate nucleus and cortex are labeled in orange and green, respectively (*p*<0.01).
(PDF)

Table S3 GSEA of deregulated genes in nematode cells. For each enrichment category shown, gene sets are shown by increasing normalized enrichment score (NES) values. No enrichment was detected for 19Q cells. Enrichments were considered to be significant for FDR q-Val <0.25 as previously described [59]. This analysis was performed using gene sets from wormbook*, KEGG annotations^$^, and Gene Ontology annotations with^#^ or without^¥^ Inferred from Electronic Annotation.
(DOCX)

Table S4 Pathways and processes enriched in genes specifically deregulated by 128Q expression as shown by GSEA. *n* is the count of genes found in the gene set enriched in deregulation versus the total Gene Set. *Underlined are the genes deregulated in 128Q cells (FDR<0.01) or human HD caudate nucleus as previously reported [29] (*p*<0.01) or both. **%H is the percent of core genes with human homolog(s) as indicated by Inparanoid clusters. Gene sets are from Wormbook***, KEGG annotations^$^, and Gene Ontology annotations with^¥^ or without^#^ Inferred from Electronic Annotation.
(DOCX)

Table S5 RT-PCR analysis for hits of interest as emphasized by microarray data analysis.
(DOCX)

Table S6 Pathways/processes highlighted by Fourier analysis and GSEA.
(DOCX)

Table S7 Putative *daf-16*/FOXO transcriptional targets that are also found in the transcriptomic signature of expanded-polyQ nematode touch cells. Data on putative *daf-16*/FOXO targets are from the studies of Murphy et al. [60], MacElwee et al. [61], and Oh et al. [62]. *Microarray data. **Chromatin immunoprecipitation data. ***Gene expression entropy was assessed using entropy-based feature selection as previously described [5] across 14 HD-associated conditions including the striatum of N-terminal htt transgenic mice R6/2 (at 6 wk and 12 wk) and D9-N171-98Q (a.k.a. DE5; at 14 mo) [63],[64], full-length htt transgenic mice YAC128 (at 12 mo and 24 mo), knock-in mice CHL2 (at 22 mo) and HdH(Q92/Q92) (at 18 mo), caudate nucleus and BA4/BA9 cortex from post-mortem HD brains [29], blood samples from presymptomatic and symptomatic HD carriers [65], and HD-induced pluripotent stem (iPS) cells that were differentiated into neural stem cell (NSC) lines and that expressed 60 or 180 CAG repeats [66]. Most putative *daf-16* targets that are highly conserved in the mouse have moderate to high entropy values, suggesting that their behavior is significantly dependent on the HD-associated context (cell type, time requirement) in which they operate. NA, not applicable.
(DOCX)

Table S8 Names and genotypes of the *C. elegans* strains used in this study.
(DOCX)

Text S1 Supplementary materials and methods.
(DOC)

Text S2 Supplementary results.
(DOC)

## Abbreviations

AD: Alzheimer's disease
APP: amyloid precursor protein
CTF: C-terminal fragment
DAPI: diamidino-2-phenylindole
ECL: enhanced chemiluminescence
FACS: Fluorescence Activated Cell Sorting
FHRE: Forkhead responsive element
GSEA: Gene Set Enrichment Analysis
HD: Huntington's disease
HTT: huntingtin
ICD: intracellular domain
LOF: loss-of-function
NOS: nitric oxide synthase
polyQ: polyglutamines
