# Correction: The Wnt Receptor Ryk Reduces Neuronal and Cell Survival Capacity by Repressing FOXO Activity During the Early Phases of Mutant Huntingtin Pathogenicity

**DOI:** 10.1371/journal.pbio.1001925

**Published:** 2014-07-15

**Authors:** The PLOS Biology Staff

The eleventh author's name is spelled incorrectly. The correct name is: Jung Mook Lyu.
