# Supplementary figures and images for: The Wnt Receptor Ryk Reduces Neuronal and Cell Survival Capacity by Repressing FOXO Activity During the Early Phases of Mutant Huntingtin Pathogenicity
Source: PLoS Biol. 2014 Jun 24;12(6):e1001895. doi: 10.1371/journal.pbio.1001895 (PMC4068980; doi:10.1371/journal.pbio.1001895)

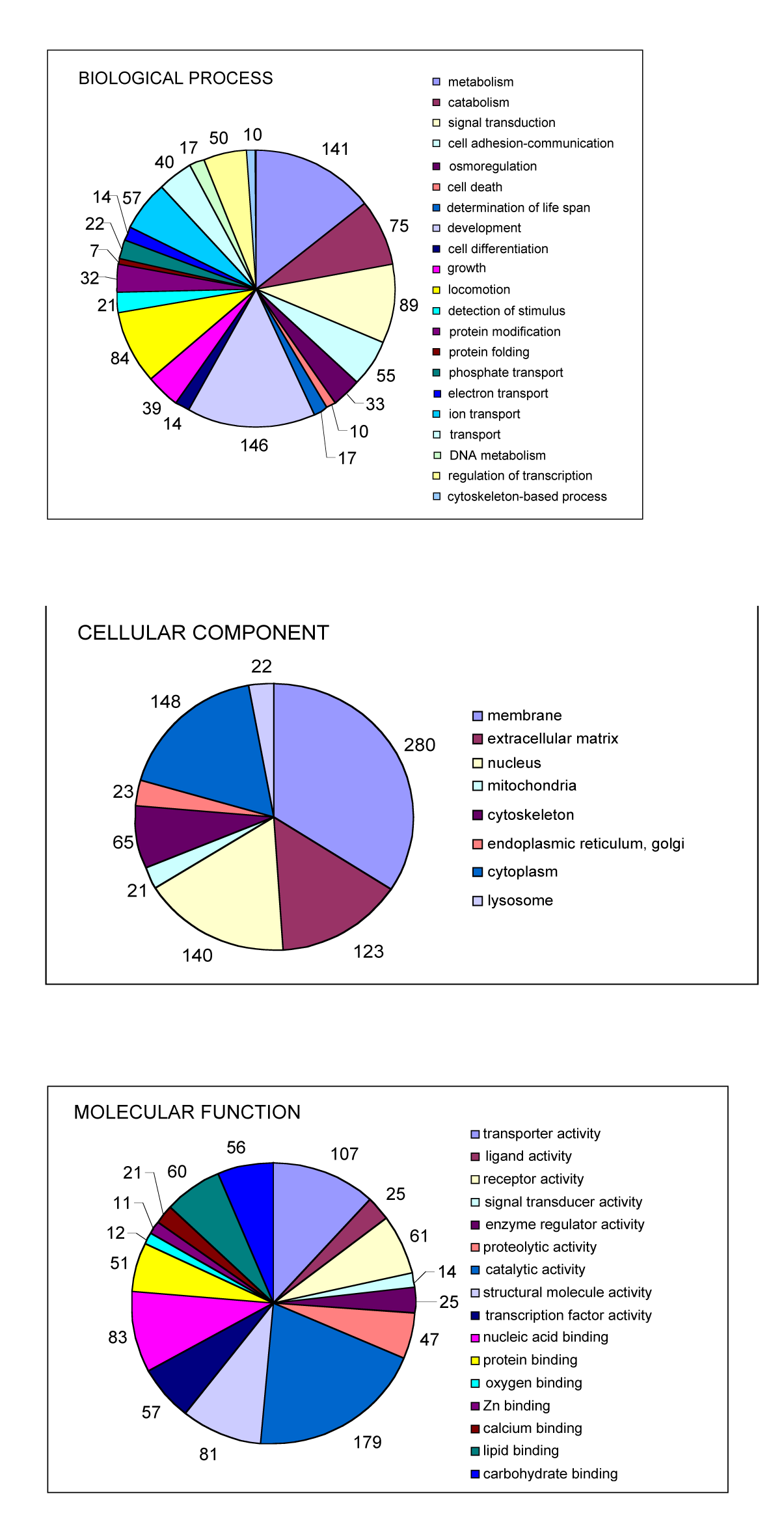

Supplement: Figure S1 — Gene Ontology classification of genes specifically deregulated by expanded polyQ expression in nematode neurons (Pmec-3 targets). Genes were classified based on their functional annotations in the GO categories “Biological Process,” “Molecular Function,” and “Cellular Component.” The number of genes is indicated. (TIF) [file pbio.1001895.s001.tif]

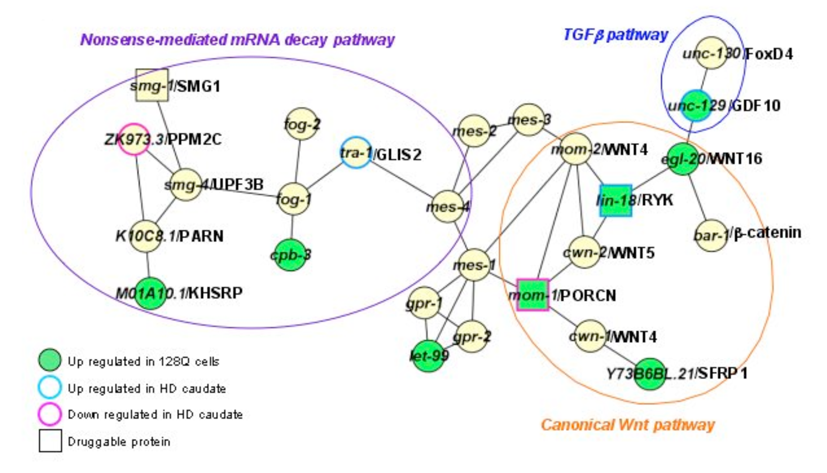

Supplement: Figure S4 — Fourier analysis module containing the Wnt, TGF-β, and nonsense-mediated mRNA decay pathways (up-regulated module 40). Green nodes indicate up-regulated nematode genes with FDR<0.01. Node borders in purple and blue indicate down- and up-regulation, respectively, of human homologs in HD caudate nucleus [29] with p<0.01. Square shapes indicate druggable genes [28]. (TIF) [file pbio.1001895.s004.tif]

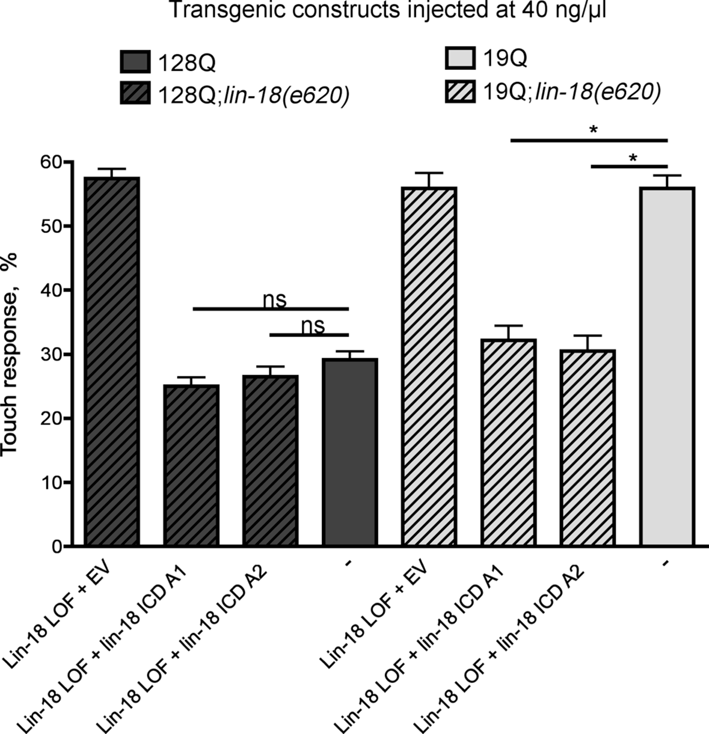

Supplement: Figure S5 — Effects of overexpressing LIN-18 ICD at 40 ng/µl in polyQ;lin-18 nematodes. Overexpressing LIN-18 ICD cDNA at 40 ng/µl in touch receptor neurons using the mec-3 promoter abolishes the neuroprotective activity of lin-18 LOF in 128Q nematodes. This effect showed a trend toward exacerbation of 128Q cytotoxicity but did not reach statistical significance relative to 128Q nematodes. Overexpressing LIN-18 ICD at 40 ng/µl produced cytotoxicity in 19Q nematodes. Two independent lin-18 ICD extrachromosomal arrays (A1, A2) were tested per polyQ genotype. The expression of LIN-18 ICD cDNA was confirmed by RT-PCR for all of the arrays generated. EV, empty vector overexpression. Data are means ± SEM (more than 200 animals tested). *p<0.001 compared to 19Q animals. ns, not significant. Significance was tested using one-way ANOVA, with correction for multiple testing by Tukey's Multiple Comparison Test. (TIF) [file pbio.1001895.s005.tif]

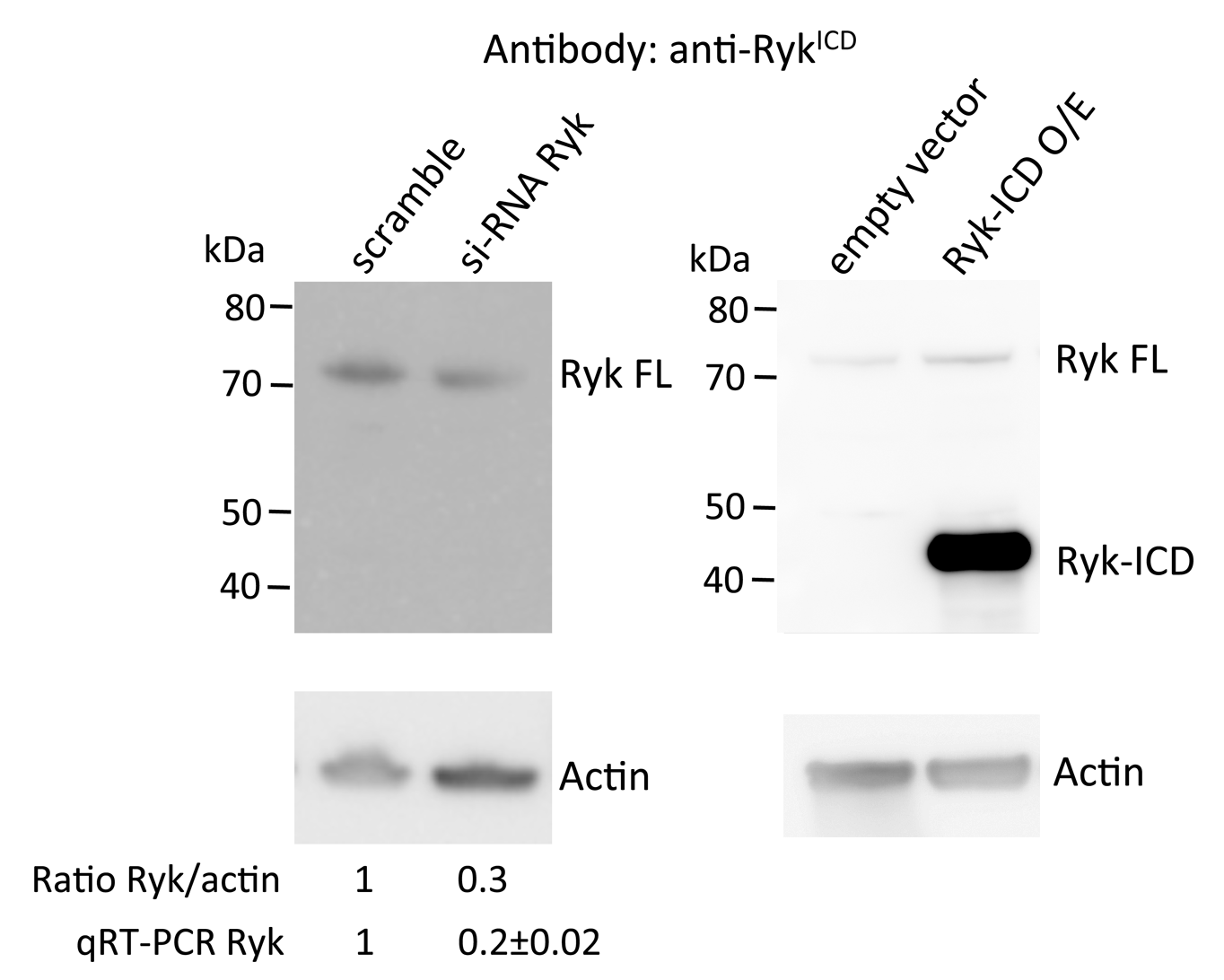

Supplement: Figure S6 — Effects of Ryk siRNA treatment and Ryk-ICD overexpression on the detection of Ryk species by antibody anti-RykICD in protein extracts from 109/109Q mouse striatal cells. Representative Western blot showing that Ryk siRNA treatment reduces the detection of full-length Ryk (Ryk FL), an effect accompanied by decreased Ryk mRNA levels as tested by qRT-PCR (left panel). Although the antibody anti-RykICD does not detect endogeneous levels of Ryk-ICD in these experiments, it detects Ryk-ICD when this Ryk fragment is overexpressed (Ryk-ICD O/E), with no signal detected for empty vector (right panel). (TIF) [file pbio.1001895.s006.tif]

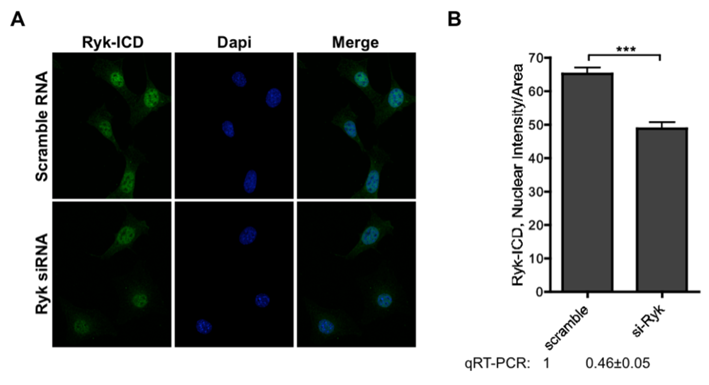

Supplement: Figure S7 — Ryk siRNA treatment decreases nuclear Ryk-ICD immunoreactivity in 109/109Q mouse striatal cells. (A) Representative confocal images showing the pattern of Ryk-ICD immunoreactivity in 109Q/109Q striatal cells upon scramble RNA or Ryk siRNA treatment as detected using the rabbit polyclonal antibody anti-RykICD. The Ryk-ICD signals were primarily detected in the nucleus, with weak signals detected in the cytoplasm. (B) Quantification of anti-RykICD nuclear signals in 109Q/109Q striatal cells. Nuclear Ryk-ICD immunoreactivity was decreased by about 25% by Ryk siRNA treatments. Data are mean ± SEM for the ratio Intensity/Area as detected in nuclei (n = 3 for a total of at least 90 cells analyzed), ***p<0.0001 compared to scramble RNA. Reduction of Ryk mRNA levels (mean reduction, 54%) was tested by qRT-PCR. (TIF) [file pbio.1001895.s007.tif]

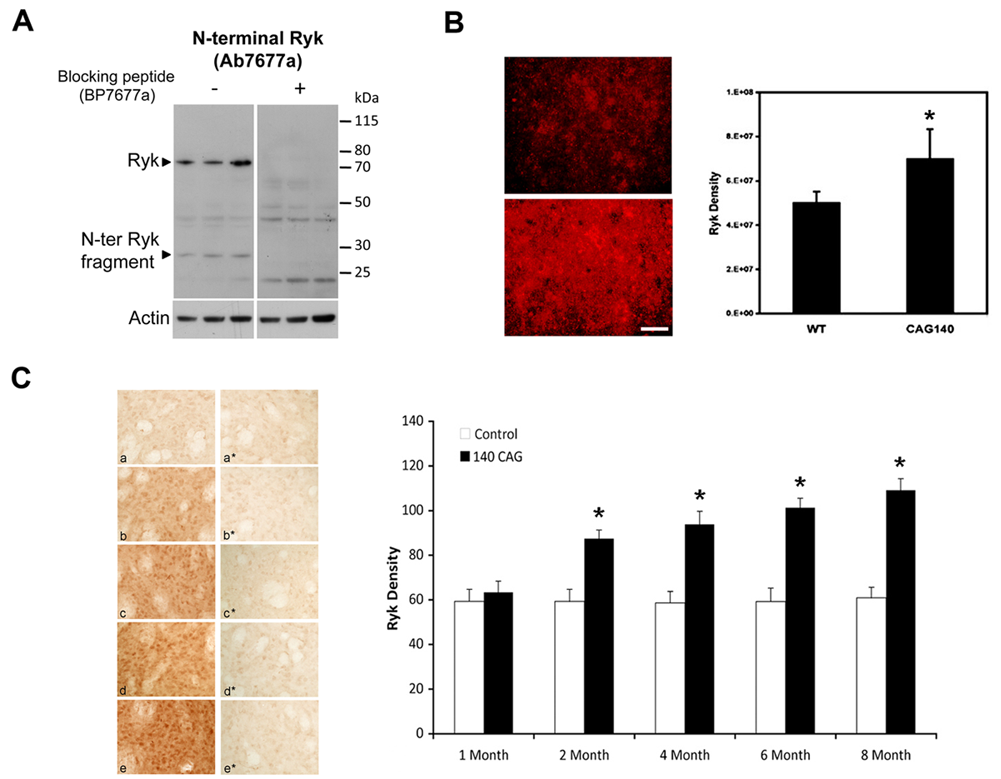

Supplement: Figure S8 — Ryk is increased in the striatum of 140CAG HD mice. (A) Western blot analysis of Ryk expression in striatal protein lysates from 4-wk-old 140CAG homozygous mutant mice (n = 3). Using the N-terminal Ryk antibody Ab7577a without or with preincubation with blocking peptide BP7577a, two specific bands were detected, including one band corresponding to the full-length protein and a weaker band of ∼28 kDa likely corresponding to a Ryk extracellular domain fragment (see comments in the Results section). (B) Densitometric analysis showed an increase of Ryk immunofluorescence from the striata of 8-mo-old 140CAG HD mice (lower panel) compared with controls (upper panel) (n = 10 per genotype), *p<0.04. Scale bar, 50 µm. Significance was tested using one-way ANOVA. (C) Chronological immunohistochemical analysis of Ryk expression in the striatum of 140CAG mice. Left panels show example images for 140CAG mice at 1, 2, 4, 6, and 8 mo of age (a–e). Scale bar, 100 µm. Right panels show example images for wild-type mice at 1, 2, 4, 6, and 8 mo (a*–e*). 140CAG mice show a significant age-dependent increase of Ryk levels at 2, 4, 6, and 8 mo of age. Data are means ± SD with n = 10 in each group. *p<0.001 compared to control. Significance was tested using one-way ANOVA, with correction for multiple testing by Tukey's Multiple Comparison Test. (TIF) [file pbio.1001895.s008.tif]

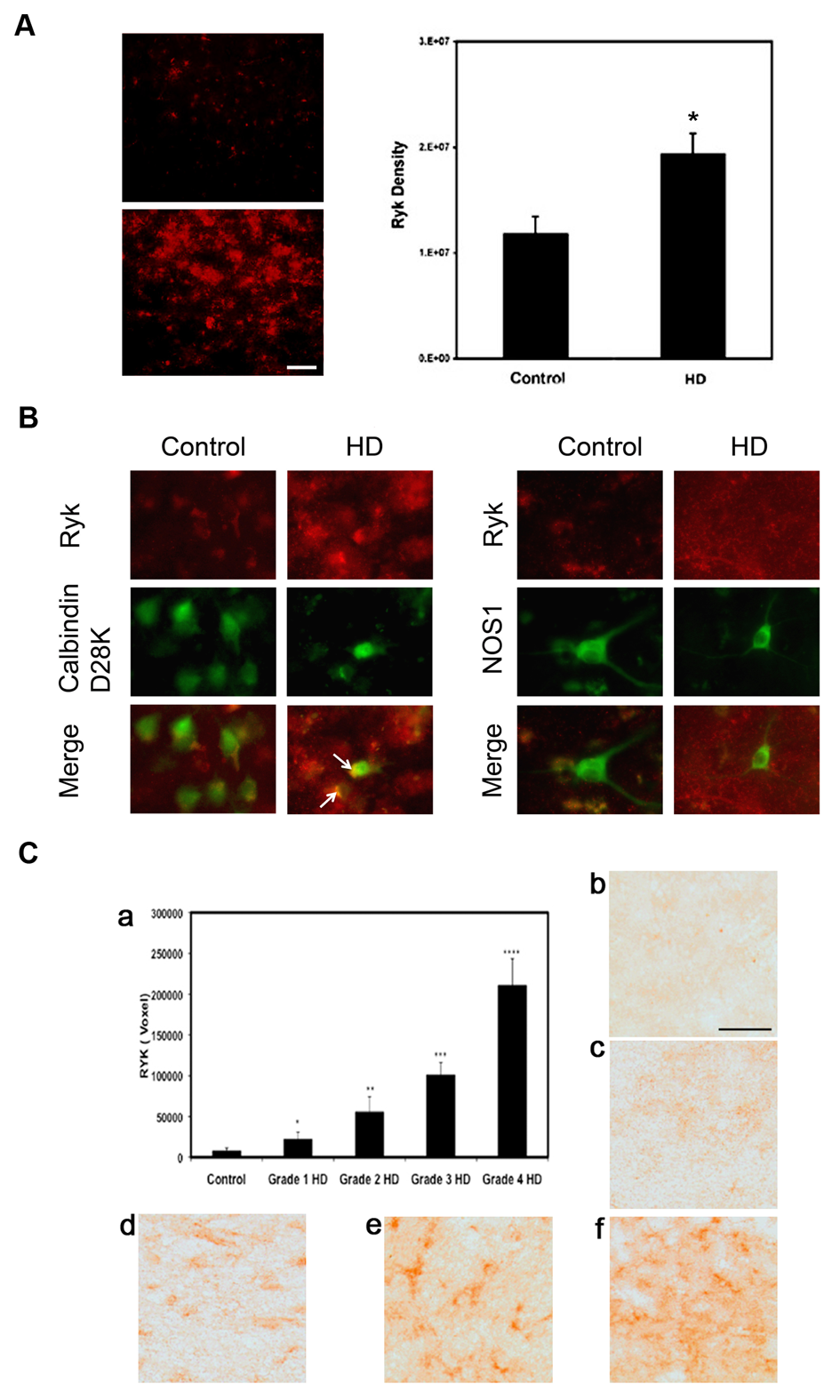

Supplement: Figure S9 — Ryk is increased in human HD caudate nucleus. Ryk immunofluorescence was assessed using the same Ryk antibody as in Figure S7. (A) Compared with age-matched controls (upper panel), Ryk immunofluorescence was increased in human caudate nucleus of grade 1/2 patients (lower panel). Densitometric analysis (right panel) showed increased Ryk protein levels in HD caudate nucleus (right) compared with controls (left) (n = 6 per group), *p<0.02. Scale bar, 50 µm. Significance was tested using one-way ANOVA. (B) Selective Ryk expression in brain striata. Combined immunofluorescence showed that Ryk co-localized (white arrows) with calbindin (degenerating neurons) but not with NOS immunoreactivity (spared neurons) in caudate neurons in brains from HD patients. Some level of co-localization was detected in control brain striata. (C) Densitometric analysis showed a disease grade-dependent increase of RYK immunoreactivity in human HD caudate nucleus. Level of Ryk expression in control (n = 5), Grade 1 HD (n = 4), Grade 2 HD (n = 5), Grade 3 HD (n = 5), and Grade 4 HD (n = 4) samples. *p<0.05 compared to control; **p<0.02 compared to Grade 1 HD; ***p<0.01 compared to Grade 2 HD; ****p<0.01 compared to Grade 3 HD. Also shown are images of Ryk immunostaining. (a) Immunoexpression of Ryk was low in the brain tissues of age-matched controls. (b–e) Increased Ryk immunoreactivity correlated with the degree of neuropathological severity (b, Control; c, Grade 1; d, Grade 2; e, Grade 3; f, Grade 4). Scale bar, 100 µm. Significance was tested using one-way ANOVA, with correction for multiple testing by Tukey's Multiple Comparison Test. (TIF) [file pbio.1001895.s009.tif]

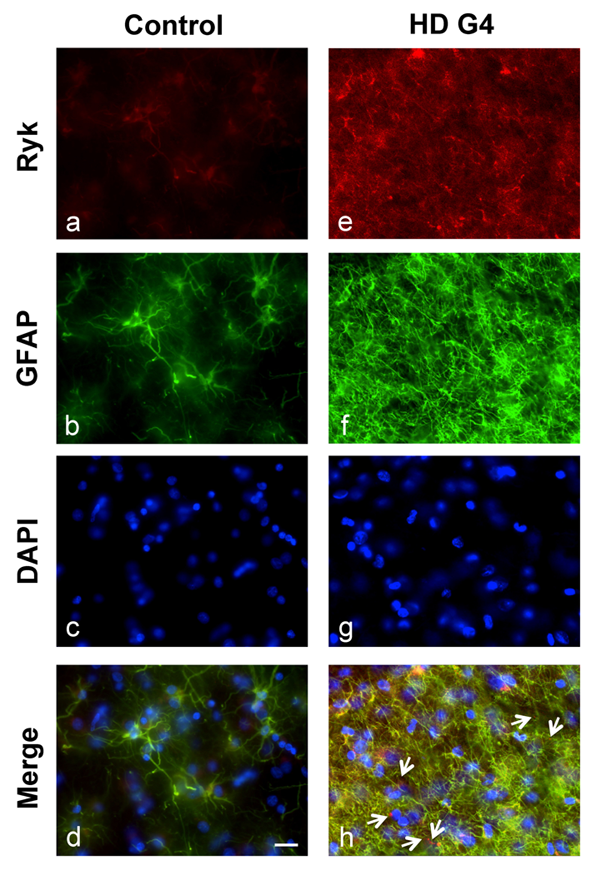

Supplement: Figure S10 — Partial co-localization of Ryk and GFAP immunofluorescence in Grade 4 HD caudate nucleus. Combined Ryk GFAP immunofluorescence shows co-localization with Ryk and GFAP in both Grade 4 HD (HD G4) striatum and, to a much smaller degree, in normal age-matched (Control) specimens. Compared with age-matched control specimens (a–d), Ryk immunofluorescence was increased within astroglia from human caudate nucleus Grade 4 HD patients (e–f), but not on a one-to-one basis. Several Ryk immunofluorescence signals were observed in the GFAP-negative area (white arrows), and there are multiple glial figures without co-localized Ryk. Ryk immunofluorescence was assessed using the same Ryk antibody as in Figure S8. Scale bar, 20 µm. (TIF) [file pbio.1001895.s010.tif]
