# Supplementary material for: The Wnt Receptor Ryk Reduces Neuronal and Cell Survival Capacity by Repressing FOXO Activity During the Early Phases of Mutant Huntingtin Pathogenicity
Source: PLoS Biol. 2014 Jun 24;12(6):e1001895. doi: 10.1371/journal.pbio.1001895 (PMC4068980; doi:10.1371/journal.pbio.1001895)
Supplement: Figure S2 — Stable Fourier analysis modules containing genes up-regulated in 128Q nematode neurons (Pmec-3 targets). The genes up-regulated (FDR <0.01) in nematode neurons are indicated by green nodes. Node borders in purple and blue indicate down- and up-regulation (p<0.01) of the human best ortholog in the caudate nucleus of HD patients, respectively, as previously reported [29]. Square nodes indicate druggable genes [28]. The enrichment in GO annotations was generated using Gostat and enrichments considered significant for p<0.001. Four modules described the extracellular matrix (Nos. 1–3, No. 5), three modules membrane proteins (Nos. 4–6), two modules the cytoskeleton (Nos. 7–8), nine modules the mitochondria (Nos. 9–13, 22–23, 26, 37), one module the golgi apparatus (No. 14), and five modules the nucleus (Nos. 15–20), with other modules corresponding to carbohydrate metabolism (Nos. 21–24), lipid metabolism (Nos. 25–27), chaperones (Nos. 37–39), and general metabolism (Nos. 28–36). Of note, module 40 contained components involved in signaling pathways (Wnt and TGF-β), axonal guidance, and cell cycle. (PDF) [file pbio.1001895.s002.pdf]

Stable modules	Process	Gene Ontology annotation	Genes count / total	P-value
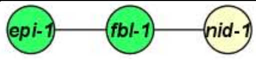 1	Proteins of basement membrane	Proteinaceous extracellular matrix	2/23	0.00050
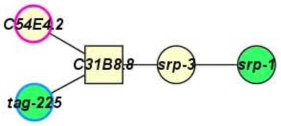 2	Protease inhibitors	Serine-type endopeptidase inhibitor activity<br>Proteinaceous extracellular matrix	2/20<br><br>2/23	0.00066<br><br>0.00028
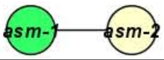 3	Sphingomyelinases	Sphingolipid catabolic process	2/2	2.6e-07
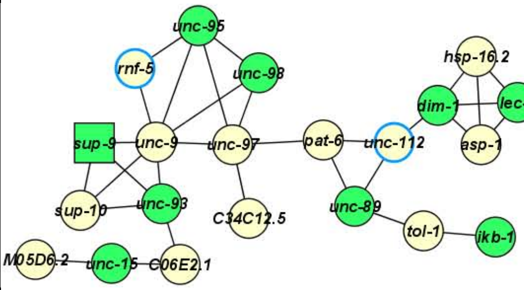 4	Channel complex subunits, lectins, innexins	N/A	N/A	N/A
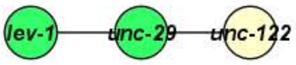 5	Acetylcholine receptor and extracellular matrix	Nicotinic acetylcholine-activated cation-selective channel activity	2/11	0.0004
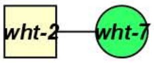 6	Transporters, ABC superfamily	N/A	N/A	N/A
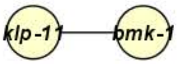 7	Kinesin-like proteins	Cytoskeleton-dependent intracellular transport	2/34	0.00031
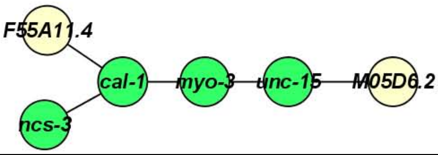 8	Cytoskeleton and Calcium binding proteins	N/A	N/A	N/A
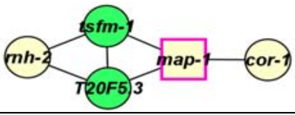 9	Enzymes of mitochondrial translation	N/A	N/A	N/A
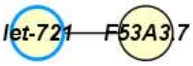 10	Electron transfer flavoprotein ubiquinone oxidoreductase	N/A	N/A	N/A
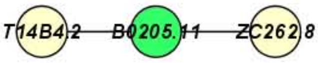 11	Mitochondrial/chloroplast ribosomal proteins S18	Ribonucleoprotein complex<br>structural Constituent of ribosome	3/153<br><br>3/116	0.00021<br><br>1.5e-05
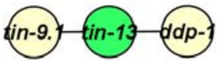 12	Mitochondrial import inner membrane translocases	N/A	N/A	N/A
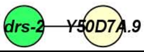 13	Enzymes, mitochondrial	N/A	N/A	N/A

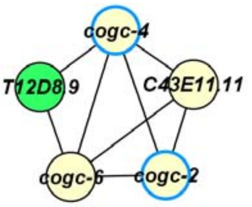 14	Golgi proteins	N/A	N/A	N/A
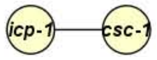 15	Centromere protein, chromosome segregation	N/A	N/A	N/A
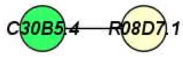 16	Predicted RNA-binding protein	N/A	N/A	N/A
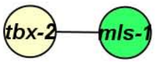 17	T-box transcription factors	N/A	N/A	N/A
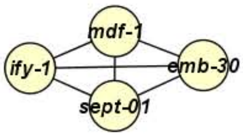 18	Proteins of cell cycle	N/A	N/A	N/A
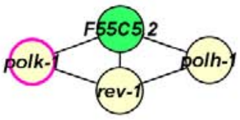 19	DNA polymerases	DNA repair<br>response to DNA<br><br>Damage stimulus<br>Response to stress	3/75<br><br><br>3/84<br>3/140	0.00020<br><br><br>0.00020<br>0.00069
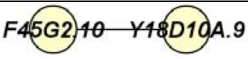 20	Y18D10A.9, WD40 repeat protein	N/A	N/A	N/A
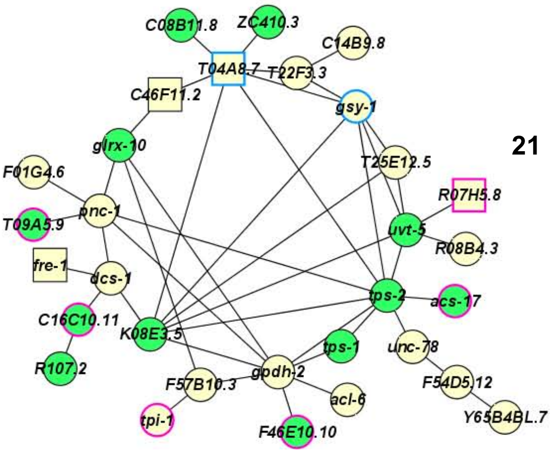 21	Enzymes of carbohydrate metabolism	Energy derivation by oxidation of organic compounds	5/24	5.3e-09
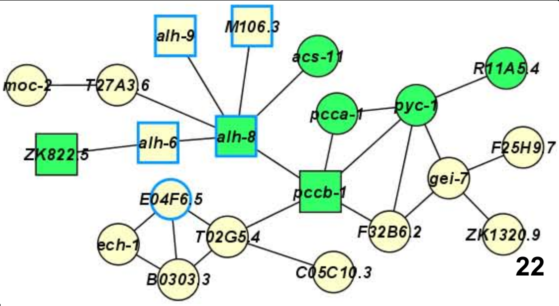 22	Enzymes of glucose metabolism and energy metabolism	Monocarboxylic acid metabolic process<br><br>Ligase activity, forming carbon-carbon bonds	5/48<br><br>2/2	2.3e-05<br><br>0.00068
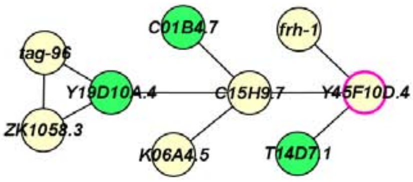 23	Enzymes of galactose metabolism	Galactose metabolic process	2/3	0.00036

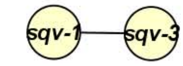 24	Proteins of carbohydrate metabolism	N/A	N/A	N/A
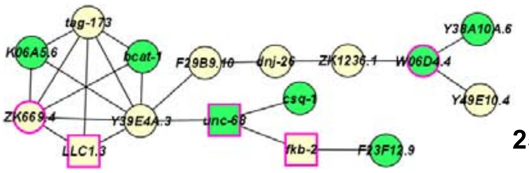 25	Enzymes of lipid metabolism	N/A	N/A	N/A
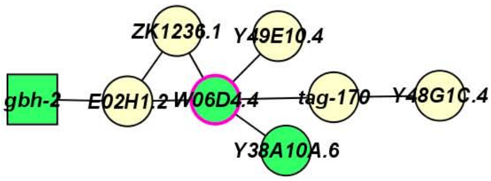 26	Enzymes of lipid metabolism	N/A	N/A	N/A
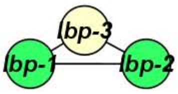 27	Fatty acid-binding proteins	Lipid binding	3/44	7.9e-07
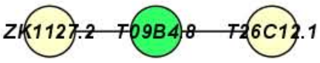 28	Enzymes of metabolism	N/A	N/A	N/A
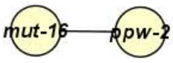 29	Translation initiation factors and related proteins	N/A	N/A	N/A
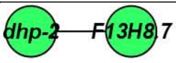 30	Hydrolases	N/A	N/A	N/A
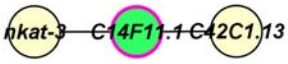 31	Transaminases	Transaminase activity	2/21	0.00044
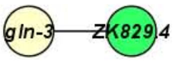 32	Glutamine synthetase, glutamate dehydsrogenase	N/A	N/A	N/A
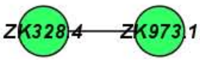 33	Metalloprotease and Transcription accessory protein	N/A	N/A	N/A
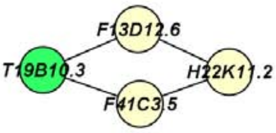 34	Beta-galactosidases and serine carboxy peptidases	Beta-galactosidase activity<br><br>Serine carboxy-peptidase activity	2/2<br><br>2/6	3.6e-06<br><br>3.6e-05
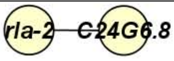 35	rla-2, acidic ribosomal subunit protein P2	N/A	N/A	N/A
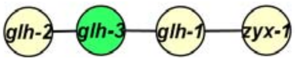 36	ATP-dependent RNA helicases and focal adhesion adaptator protein	JUN kinase binding<br>ATP-dependent Helicase activity<br>Pole plasm	3/4<br>3/62<br>3/21	9.6e-09<br>3.3e-05<br>3.6e-06
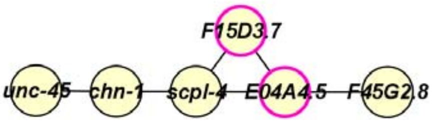 37	Chaperones, cytoskeletal and mitochondrial proteins	Ubiquitin protein ligase binding	2/3	9.2e-05
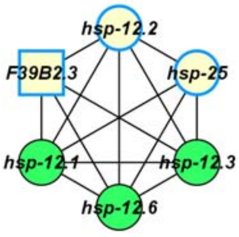 38	Heat shock proteins	N/A	N/A	N/A
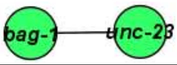 39	Chaperones	N/A	N/A	N/A

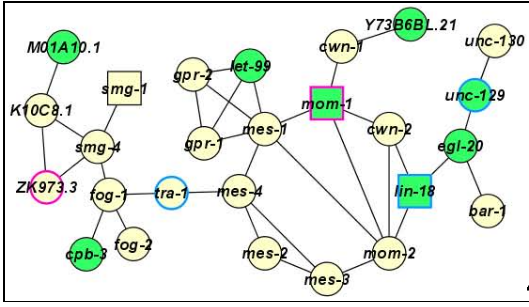 **40**	Wnt signaling pathway, transcription factors and axonal guidance	Microtubule cytoskeleton organization and biogenesis<br><br>Microtubule-based process	4/80<br><br>4/106	0.00047<br><br>0.00096
