# Supplementary material for: The Wnt Receptor Ryk Reduces Neuronal and Cell Survival Capacity by Repressing FOXO Activity During the Early Phases of Mutant Huntingtin Pathogenicity
Source: PLoS Biol. 2014 Jun 24;12(6):e1001895. doi: 10.1371/journal.pbio.1001895 (PMC4068980; doi:10.1371/journal.pbio.1001895)
Supplement: Figure S3 — Stable Fourier analysis modules containing genes down-regulated in 128Q nematode neurons (Pmec-3 targets). The genes down-regulated (FDR<0.01) in nematode neurons are indicated by red nodes. Node borders in purple and blue indicate down- and up-regulation (p<0.01) of the human best ortholog in the caudate nucleus of HD patients, respectively, as previously reported ([29]; see [3]). Square nodes indicate druggable genes [28]. The enrichment in GO annotations was generated using Gostat and enrichments considered significant for p<0.001. The modules obtained described extracellular components (Nos. 1–3), membrane (Nos. 4–10), transport (No. 11), nucleus (Nos. 13–15), calcium sensors (No. 21), metabolism (Nos. 16–19), and signal transducers such as neuropeptides and the Ras and hedgehog pathways (Nos. 23–28). (PDF) [file pbio.1001895.s003.pdf]

| Stable modules | Process | Gene Ontology annotation | Genes count / total | P-value |
|---|---|---|---|---|
| 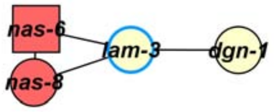 1 | Metalloproteases and basal membrane proteins | N/A | N/A | N/A |
| 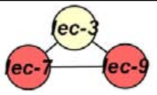 2 | Lectins | Sugar binding | 3/80 | 1.66e-05 |
| 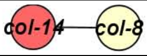 3 | Collagens | N/A | N/A | N/A |
| 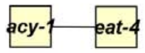 4 | Permease and adenylyl cyclase | N/A | N/A | N/A |
| 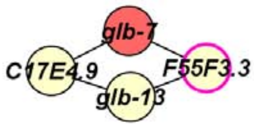 5 | Hemoproteins and Na$^+$/K$^+$ ATPases | Oxygen transport<br><br>Sodium:potassium-exchanging ATPase activity | 2/3<br><br>2/3 | 2.72e-05<br><br>2.72e-05 |
| 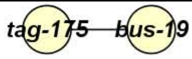 6 | Predicted membrane proteins | N/A | N/A | N/A |
| 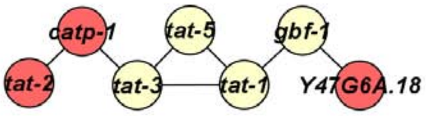 7 | P-type ATPases and Golgi proteins | phospholipid-translocating ATPase activity | 4/6 | 3.37e-10 |
| 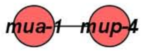 8 | Involved in muscular attachment | N/A | N/A | N/A |
| 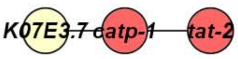 9 | P-type ATPases | ATPase activity, coupled to transmembrane movement of ions, phosphorylative mechanism | 3/23 | 3.52e-06 |
| 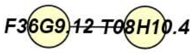 10 | Integral membrane O-acyltransferase | N/A | N/A | N/A |
| 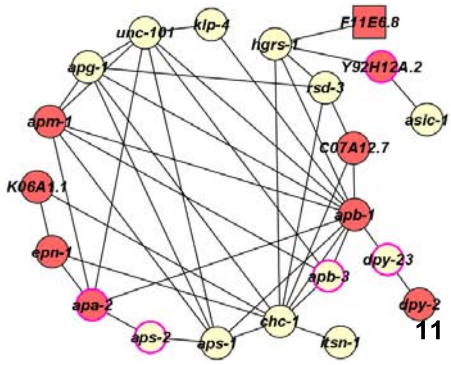 11 | Adaptins and transport proteins | Protein complex assembly<br><br>Intracellular protein transport<br><br>Clathrin coat | 10/40<br><br>12/111<br><br>7/13 | 7.67e-15<br><br>3.67e-14<br><br>2.07e-13 |
| 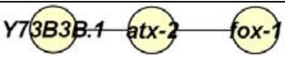 12 | RNA-binding and actin-binding proteins | N/A | N/A | N/A |
| 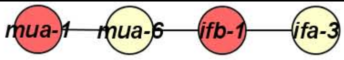 13 | Nuclear envelope protein lamins | | | |
| 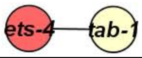 14 | Transcription factors | N/A | N/A | N/A |
| 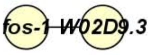 15 | Transcription activators | N/A | N/A | N/A |
| 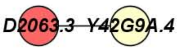 16 | Y42G9A.4, ortholog of mevalonate kinase | N/A | N/A | N/A |
| 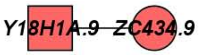 17 | Zinc carboxypeptidases | Carboxypeptidase A activity | 2/8 | 7.04e-05 |

| | | | | |
|---|---|---|---|---|
| 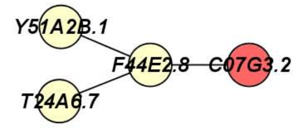 18 | Predicted riboflavin biosynthesis proteins | N/A | N/A | N/A |
| 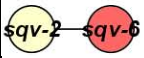 19 | Carbohydrate group transferases | N/A | N/A | N/A |
| 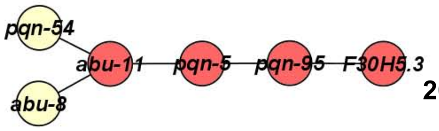 20 | Prion-like-(Q/N-rich)-domain-bearing protein | N/A | N/A | N/A |
| 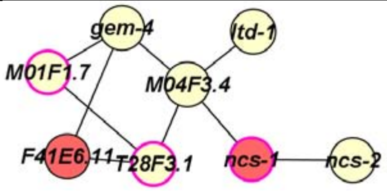 21 | Calcium sensors and calcium binding proteins | Calcium ion binding | 3/105 | 0.000802 |
| 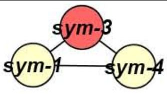 22 | Synthetic lethal with Mec | N/A | N/A | N/A |
| 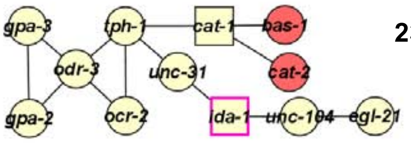 23 | G protein subunits, kinesin, transporter, neurotransmitter catabolism | N/A | N/A | N/A |
| 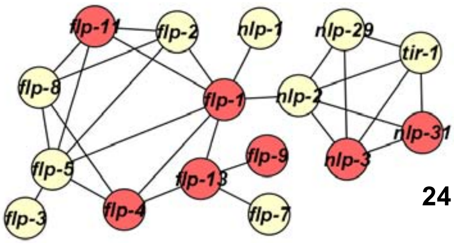 24 | FMRFamide-like peptide and neuropeptide-like protein | Neuropeptide signaling pathway<br><br>G-protein coupled receptor protein signaling pathway | 5/10<br><br>5/83 | 1.25e-11<br><br>7.00E-07 |
| 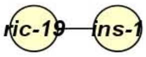 25 | Neuroendocrine pathway | N/A | N/A | N/A |
| 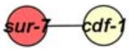 26 | Let-60/ Ras signaling pathway | N/A | N/A | N/A |
| 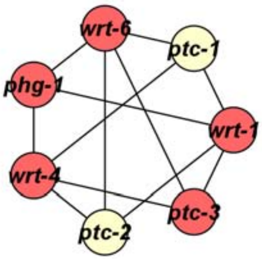 27 | Patched and hedgehog homolog protein | Intein-mediated protein splicing<br><br>Hedgehog receptor activity | 3/8<br><br>3/9 | 6.21e-06<br><br>6.21e-06 |

| | | | | |
|---|---|---|---|---|
| 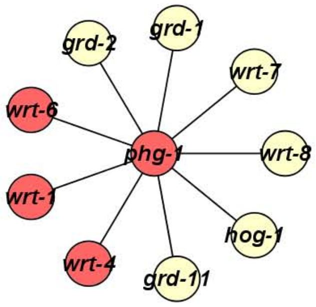<br>**28** | Hedgehog-like proteins | Intein-mediated protein splicing<br>Proteolysis<br>Cell communication | 8/8<br>9/183<br>9/445 | 2.1e-22<br>1.82e-11<br>4.81e-08 |
