# Supplementary material for: The Wnt Receptor Ryk Reduces Neuronal and Cell Survival Capacity by Repressing FOXO Activity During the Early Phases of Mutant Huntingtin Pathogenicity
Source: PLoS Biol. 2014 Jun 24;12(6):e1001895. doi: 10.1371/journal.pbio.1001895 (PMC4068980; doi:10.1371/journal.pbio.1001895)
Supplement: Table S1 — Genes deregulated in 19Q nematode cells versus control (GFP alone) cells. Forty-one worm genes were found to be deregulated. Most of them were down-regulated (34/41) and about a half of them encoded extracellular and catalytic proteins or were associated to membranes. Gene Ontology (GO) enrichment tests indicated that Pepsin A activity (GO:0004194) was enriched (p<0.01). Among these 41 genes, six genes conserved in humans. Also shown are the best homologous genes in humans with their expression levels in the HD brain (caudate and cortex) versus control brains [29]. Human genes deregulated in HD caudate nucleus and cortex are labeled in orange and green, respectively (p<0.01). (PDF) [file pbio.1001895.s011.pdf]

ORF	Name	Log Ratio	FDR	Human protein homolog	Human gene homolog	Human gene title	Human gene symbol	caudate_log2fc	caudate_pValue	cortex_BA4_log2fc	cortex_BA4_pValue
F38A5.9	nspb-5	-4.94588130	3.19E-06	-							
Y62H9A.7		-4.83988130	2.43E-10	-							
K07D4.8	pqn-48	-4.45388130	1.35E-05	ENSP00000262807	ENSG00000105648	interferon, gamma-inducible protein 30	IFI30				
C15C7.4		-4.36788130	1.15E-05	-							
Y35H6.1		-4.22888130	0.00043567	-							
F20A1.1		-3.83388130	2.06E-07	-							
C24H11.4	srd-74	-3.65188130	1.15E-05	-							
Y71G12B.10		-3.42288130	1.93E-05	ENSP00000363614	ENSG00000117305	3-hydroxymethyl-3-methylglutaryl-Coenzyme A lyase	HMGCL	0.18	0.0000083	0.058	0.437
B0416.2		-3.28288130	5.26E-05	-							
Y62E10A.13		-3.03688130	5.26E-05	ENSP00000275605	ENSG00000146733	phosphoserine phosphatase	PSPH	0.267	0.283	0.0366	0.283
Y26D4A.6	clec-108	-3.01788130	0.00457507	-							
R02E12.5		-3.00588130	0.00408746	-							
K05G3.2		-2.99088130	0.00012698	-							
ZK678.6	srg-64	-2.89288130	0.00023584	-							
Y50D4C.4	sqv-6	-2.87388130	0.00023584	-							
M03B6.4		-2.86188130	5.26E-05	-							
W02D7.10	clec-219	-2.82288130	0.00023584	-							
Y116F11A.3		-2.73788130	0.00062767	-							
C05D12.1		-2.63788130	0.00580173	-							
F26D11.10	inx-4	-2.62488130	0.00338901	-							
F38A5.12	nspb-2	-2.59788130	0.0009975	-							
Y82E9BR.15	elc-1	-2.59288130	0.000734	ENSP00000284811	ENSG00000154582	transcription elongation factor B (SIII), polypeptide 1 (15kDa, elongin C)	TCEB1	-0.204	0.0000225	-0.147	0.0246
ZK546.4		-2.59288130	0.00388489	-							
C38C6.6	tag-297	-2.58688130	0.00268828	-							
W08E12.2		-2.57988130	0.00791626	-							

T10C6.9		-2.53988130	0.00268828	-			
C53D5.3		-2.48388130	0.00060121	-			
F49F1.12		-2.47688130	0.00152717	-			
M01B2.7	srsx-37	-2.46388130	0.00047765	-			
Y116F11B.8		-2.30688130	0.00036227	-			
F15G9.2		-2.11388130	0.00984344	-			
R151.4		-1.79588130	0.00689312	-			
T10H9.6	str-180	-1.71088130	0.00239801	ENSP00000352717	ENSG00000197437	olfactory receptor, family 13, subfamily G, member 1	OR13G1
M7.13	str-3	-1.68688130	0.00060121	ENSP00000352717	ENSG00000197437	olfactory receptor, family 13, subfamily G, member 1	OR13G1
T22B3.1	dpy-20	2.652118692	2.06E-07	-			
T08A9.9	spp-5	2.847118692	0.00886884	-			
W08F4.9	fbxb-14	3.012118692	0.00060121	-			
Y39B6A.20	asp-1	3.108118692	0.00388489	-			
F21F8.7	asp-6	3.387118692	0.00348052	-			
ZK792.7		3.533118692	3.19E-06	-			
Y47H10A.5		4.216118692	0.00033837	-			
