# Supplementary material for: The Wnt Receptor Ryk Reduces Neuronal and Cell Survival Capacity by Repressing FOXO Activity During the Early Phases of Mutant Huntingtin Pathogenicity
Source: PLoS Biol. 2014 Jun 24;12(6):e1001895. doi: 10.1371/journal.pbio.1001895 (PMC4068980; doi:10.1371/journal.pbio.1001895)
Supplement: Table S2 — Genes deregulated in 128Q nematode cells versus 19Q nematode cells. We found 2,070 worm genes to be deregulated. Only 18 of the 2,070 genes were also deregulated in 19Q nematode cells versus GFP cells, including pqn-48, a lysosomal thiol reductase, and four nematode-specific genes encoding membrane proteins: srg-64, nspb-2, nspb-5, and str-180. Also found were nematode genes with no human homologs as inferred from Inparanoid [58], including a lectine (clec-219), innexin (inx-4), and xylosyltransferase enzyme (sqv-6). Among the 2,070 dysregulated genes, 516 genes are conserved in humans. Also shown are the best homologous genes in humans with their expression levels in HD brains (caudate and cortex) versus control brains [29]. Human genes deregulated in HD caudate nucleus and cortex are labeled in orange and green, respectively (p<0.01). (PDF) [file pbio.1001895.s012.pdf]

ORF	WBgene	Gene Name	Log Ratio	FDR	Human protein homolog	Human gene homolog	Human gene title	Human gene symbol	caudate: log2fc	caudate: pValue	cortex_BA4: log2fc	cortex_BA4: pValue
F27E5.4	WBGene00004017	phg-1	-4.54532	2.07e-08	ENSP00000298743	ENSG00000180447	growth arrest-specific 1	GAS1	0.221	0.00016	0.223	0.00477
C03A7.4	WBGene00004099	pqn-5	-4.01067	0.00433514	ENSP00000334197	ENSG00000188155	keratin associated protein 10-6	KRTAP10-6				
C03A7.8	WBGene00000030	abu-7	-3.89295	0.000597344	ENSP00000334197	ENSG00000188155	keratin associated protein 10-6	KRTAP10-6				
C03A7.7	WBGene00000029	abu-6	-3.81447	0.00477655	ENSP00000334197	ENSG00000188155	keratin associated protein 10-6	KRTAP10-6				
F40E10.5	WBGene00009573	-	-3.77463	0.0000000543274	-							
C27H5.5	WBGene00000613	col-36	-3.68262520120724	0.0005547682	-							
F57H12.7	WBGene00003178	mec-17	-3.59864	3.05e-06	ENSP00000373079	ENSG00000206488	chromosome 6 open reading frame 134	C6orf134	-0.0567	0.107	0.00896	0.868
ZK1067.7	WBGene00004174	pqn-95	-3.55862520120724	0.0000004490373	-							
D2096.6	WBGene00017073	-	-3.53462520120724	0.000001928172	-							
F38B2.1b	WBGene00002050	ifa-1	-3.49809	0.000422248	ENSP00000327054	ENSG00000176619	lamin B2	LMNB2	0.00659	0.825	0.0167	0.67
Y105C5A.4	WBGene00000028	abu-5	-3.49073	0.000386781	ENSP00000334197	ENSG00000188155	keratin associated protein 10-6	KRTAP10-6				
F20B10.3	WBGene00008967	-	-3.42262520120724	0.000000860125	-							
F09G8.6	WBGene00000666	col-91	-3.37662520120724	0.004440461	-							
F38A6.1	WBGene00004013	pha-4	-3.32028	1.92e-05	ENSP00000250448	ENSG00000129514	forkhead box A1	FOXA1	0.0763	0.0204	-0.0181	0.764
T21B4.2	WBGene00000660	col-85	-3.29162520120724	0.00566169	-							
W02A2.3	WBGene00004156	pqn-74	-3.23762520120724	0.00001035837	-							
BE0003N10.2	WBGene00015267	chin-1	-3.23062520120724	0.004278583	-							
ZK682.5	WBGene00022789	-	-3.15262520120724	0.000004083784	-							
F22E10.3	WBGene00004008	pgp-14	-3.14862520120724	0.0002969553	-							
T08B1.2b	WBGene00006589	tnt-4	-3.14738	0.00812444	ENSP00000356286	ENSG00000118194	troponin T2, cardiac	TNNT2	-0.0381	0.262	0.00515	0.938
F40E3.5	WBGene00018232		-3.12862520120724	0.003612786	-							
W06F12.2a	WBGene00012306	-	-3.11362520120724	0.00005780488	-							

C52D10.13	WBGene00000711	col-138	-3.10262520120724	0.004947873	-								
ZK662.2	WBGene00014039	-	-3.07762520120724	0.000001928172	-								
T06C10.3	WBGene00020289		-3.04662520120724	0.002196161	-								
F59B10.3	WBGene00010319	-	-3.02062520120724	0.0001049904	-								
Y47D3B.6	WBGene00012942	-	-3.01762520120724	0.000008189924	-								
C06G1.2	WBGene00015546	-	-3.01662520120724	0.000001872256	-								
M05B5.2	WBGene00010870	-	-2.99562520120724	0.000001099089	-								
F35D11.2a	WBGene00004122	pqn-35	-2.98062520120724	0.00001249121	-								
Y49C4A.8b	WBGene00021709	ugt-29	-2.96466	0.000719072	ENSP00000282507	ENSG00000168671	UDP glycosyltransferase 3 family, polypeptide A2	UGT3A2					
F11E6.8	WBGene00008711		-2.95362	2.6e-05	ENSP00000296474	ENSG00000164078	macrophage stimulating 1 receptor (c-met-related tyrosine kinase)	MST1R	-0.0103	0.829	0.0662	0.155	
ZK813.1	WBGene00022820	-	-2.94162520120724	0.006947101	-								
C18E9.7	WBGene00007687	-	-2.93462520120724	0.00001668931	-								
F30H5.3	WBGene00017937	-	-2.90962520120724	0.000001013888	-								
ZK829.2	WBGene00001839	hdl-1	-2.89662520120724	0.004339155	-								
C14C11.8	WBGene00004104	pqn-13	-2.88373	5.33e-05	ENSP00000334197	ENSG00000188155							
F32A5.8	WBGene00017971		-2.88005	0.000678418	ENSP00000333406	ENSG00000186153	WW domain containing oxidoreductase	WWOX	0.107	0.0649	-0.107	0.139	
C33B4.3b	WBGene00006444	shn-1	-2.86901	0.000835491	ENSP00000293441	ENSG00000161681	SH3 and multiple ankyrin repeat domains 1	SHANK1	-0.0016	0.95	-0.115	0.0304	
F26G1.6	WBGene00017842	-	-2.85962520120724	0.000005241535	-								
Y54F10AM.2a	WBGene00001410	feh-1	-2.84571	0.00883552	ENSP00000318965	ENSG00000163697	amyloid beta (A4) precursor protein-binding, family B, member 2 (Fe65-like)	APBB2	-0.0727	0.101	-0.228	0.00189	
C46H11.7	WBGene00016731	-	-2.84262520120724	0.000002193184	-								

R13A5.9	WBGene00020051		-2.8359	0.00204386	ENSP00000 281416	ENSG000 00151690						
F35B12.4	WBGene00009384		-2.83345	0.00179351	ENSP00000 342098	ENSG000 00166145	Serine protease inhibitor, Kunitz type 1	SPINT1	-0.00816	0.756	0.0224	0.581
R12E2.15	WBGene00020040	-	-2.82262520120724	0.0004853088	-							
F56C9.8	WBGene00018952	-	-2.81762520120724	0.0002476666	-							
M02G9.1	WBGene00010830	-	-2.80662520120724	0.000004590152	-							
C35E7.3	WBGene00016455		-2.78762520120724	0.004225744	-							
F45E4.8	WBGene00003758	nlp-20	-2.78662520120724	0.00002634696	-							
W06F12.2c	WBGene00012306	-	-2.77962520120724	0.000035534	-							
ZK180.5c	WBGene00022679	-	-2.77662520120724	0.00272499	-							
F56D2.3	WBGene00018965	-	-2.76762520120724	0.000009948764	-							
T04C10.2a	WBGene00001329	epn-1	-2.76478	0.00456848	ENSP00000 268933	ENSG000 00049283	epsin 3	EPN3	-0.03	0.438	-0.172	0.00832
W01C9.3	WBGene00004155	pqn-73	-2.76462520120724	0.000123965	-							
F25G6.6	WBGene00003816	nrs-2	-2.76162520120724	0.0000283242	-							
H36L18.2	WBGene00010424	-	-2.76162520120724	0.0005170232	-							
Y95B8A.12	WBGene00022391		-2.7611	0.00176868	ENSP00000 344961	ENSG000 00187510						
R03C1.1	WBGene00010987	-	-2.76062520120724	0.000005241535	-							
F32H2.5	WBGene00009342	fasn-1	-2.75462520120724	0.008841172	-							
C54E4.4	WBGene00016919	-	-2.75162520120724	0.0000341181	-							
C10H11.4	WBGene00015693	ugt-28	-2.74884	0.00316516	ENSP00000 282507	ENSG000 00168671	UDP glycosyltransferase 3 family, polypeptide A2	UGT3A2				
K02G10.4c	WBGene00001454	flp-11	-2.74162520120724	0.0007326097	-							
T10E10.4	WBGene00020421	-	-2.73562520120724	0.000006740196	-							
Y50D4C.4	WBGene00005024	sqv-6	-2.73062520120724	0.004136799	-							
C18E9.9	WBGene00007689	-	-2.72162520120724	0.0001344149	-							
W01D2.5	WBGene00012182		-2.71818	0.00109447	ENSP00000 296327	ENSG000 00163959						
R02F11.1	WBGene00019839	-	-2.70862520120724	0.00001042946	-							
Y38C1BA.3	WBGene00000683	col-109	-2.70762520120724	0.0009607443	-							
W03F8.1	WBGene00006586	tni-4	-2.70469	0.00406601	ENSP00000 341838	ENSG000 00129991	troponin I, cardiac	TNNI3	-0.0113	0.696	0.00672	0.875

AC8.3	WBGene00007075	-	-2.69262520120724	0.00579306	-							
F22A3.1	WBGene00017687	ets-4	-2.67404	0.000421027	ENSP00000363149	ENSG00000124664	SAM pointed domain containing ets transcription factor	SPDEF	-0.00481	0.829	-0.0102	0.75
Y54E10BL.6	WBGene00003186	mek-2	-2.67404	0.0033321	ENSP00000302486	ENSG00000169032	mitogen-activated protein kinase kinase 1	MAP2K1	-0.883	4.81e-08	-0.255	0.0325
Y74E4A.1b	WBGene00022283	lgc-27	-2.66162520120724	0.00001541319	-							
Y54E5A.1	WBGene00013197		-2.65932	0.0001	ENSP00000307126	ENSG00000168350						
R08F11.4	WBGene00019968	-	-2.65662520120724	0.0005679671	-							
F56C3.4	WBGene00018941		-2.64562520120724	0.007697792	-							
Y57G11C.24b	WBGene00001330	eps-8	-2.64215	0.00116327	ENSP00000201647	ENSG00000131037	EPS8-like 1	EPS8L1	-0.0172	0.644	0.00362	0.963
T05B4.8	WBGene00020239	-	-2.63162520120724	0.00004358117	-							
F33A8.9	WBGene00000658	col-83	-2.62562520120724	0.009205347	-							
T25E4.1	WBGene00020803	-	-2.61162520120724	0.000008127517	-							
F36A4.6	WBGene00000610	col-33	-2.59962520120724	0.0002969553	-							
E01G6.1	WBGene00008449	-	-2.58962520120724	0.00001736177	-							
Y47G6A.18	WBGene00021644		-2.57348	0.0054676	ENSP00000265070	ENSG00000113384	Golgi phosphoprotein 3 (coat-protein)	GOLPH3	-0.0321	0.181	-0.0402	0.294
T17H7.4j	WBGene00001573	gei-16	-2.57262520120724	0.000642002	-							
F14H12.1	WBGene00000738	col-165	-2.56562520120724	0.0001146617	-							
ZC84.1	WBGene00013846	-	-2.56562520120724	0.0007934975	-							
W08E12.2	WBGene00021083	-	-2.56062520120724	0.000008499885	-							
K02G10.4b	WBGene00001454	flp-11	-2.55962520120724	0.000605359	-							
D2005.2	WBGene00003746	nlp-8	-2.55462520120724	0.001363145	-							
F19H8.3	WBGene00000188	arl-3	-2.55141	0.000405324	ENSP00000260746	ENSG00000138175	ADP-ribosylation factor-like 3	ARL3	-0.225	0.00321	-0.18	0.054
T08A11.1	WBGene00011604		-2.54262520120724	0.0007609562	-							
Y74E4A.1a	WBGene00022283	lgc-27	-2.54062520120724	0.00003721851	-							
H27M09.4	WBGene00001075	dpy-14	-2.53462520120724	0.0001745737	-							
W02D7.10	WBGene00020945	-	-2.51462520120724	0.003707096	-							

T23F1.6	WBGene00004153	pqn-71	-2.49868	0.000247721	ENSP00000334197	ENSG00000188155	keratin associated protein 10-6	KRTAP10-6				
Y73F4A.1	WBGene00013514	-	-2.49162520120724	0.002638225	-							
Y73F4A.2	WBGene00013515	-	-2.48362520120724	0.001790244	-							
F38B2.1a	WBGene00002050	ifa-1	-2.48274	0.000288896	ENSP00000327054	ENSG00000176619	lamin B2	LMNB2	0.00659	0.825	0.0167	0.67
F09F3.6	WBGene00008628	-	-2.47862520120724	0.004615718	-							
H06H21.10a	WBGene00019166	tat-2	-2.47783	0.00597867	ENSP00000284509	ENSG00000104043	ATPase, Class I, type 8B, member 4	ATP8B4	-0.0176	0.582	-0.0196	0.611
T10C6.10	WBGene00011691		-2.47362520120724	0.009818352	-							
F13H8.4	WBGene00017437	-	-2.45862520120724	0.00001056295	-							
ZK1151.1e	WBGene00006876	vab-10	-2.44963	0.000468236	ENSP00000359805	ENSG00000151914	dystonin	DST	0.0741	0.222	-0.000465	0.989
T22H2.5a	WBGene00011935	scrm-1	-2.44227	0.00254964	ENSP00000345494	ENSG00000188313	phospholipid scramblase 1	PLSCR1	0.331	0.0526	0.133	0.253
F54B11.5	WBGene00010028		-2.43982	0.00659622	ENSP00000265981	ENSG00000110315	ring finger protein 141	RNF141	-0.0511	0.289	-0.281	0.000964
T04B2.5	WBGene00011423	-	-2.43762520120724	0.005955538	-							
C07G3.2	WBGene00015574	-	-2.43462520120724	0.0007983517	-							
Y6B3B.10	WBGene00006505	lagr-1	-2.42462520120724	0.0001723422	-							
Y73B6BL.34	WBGene00000686	col-112	-2.42462520120724	0.00538805	-							
F54D1.6	WBGene00010047	-	-2.42162520120724	0.004340453	-							
C17G10.5	WBGene00003097	lys-8	-2.40162520120724	0.0001238028	-							
F09F7.8	WBGene00017305	nspb-12	-2.39962520120724	0.00029516	-							
Y17G7B.11	WBGene00012464		-2.3969	0.000358432	ENSP00000222250	ENSG00000105643	arrestin domain containing 2	ARRDC2	0.416	1.11e-08	0.072	0.4
ZC101.2b	WBGene00006787	unc-52	-2.39567	0.000586231	ENSP00000363827	ENSG00000142798	heparan sulfate proteoglycan 2 (perlecan)	HSPG2	0.00454	0.915	-0.0173	0.754
M04G7.1	WBGene00019772	-	-2.39262520120724	0.0001627591	-							
T03D8.6	WBGene00011393	-	-2.38462520120724	0.0002908258	-							
C10H11.6	WBGene00015695	ugt-26	-2.37728	0.000623267	ENSP00000282507	ENSG00000168671	UDP glycosyltransferase 3 family, polypeptide A2	UGT3A2				

T19B10.2	WBGene00011831	-	-2.37662520120724	0.00001976734	-							
R07E3.2	WBGene00011103	-	-2.37662520120724	0.00002510609	-							
C06B8.7	WBGene00007372		-2.37605	0.000463584	ENSP00000315945	ENSG00000177675	scavenger receptor cysteine-rich type 1 protein M160	M160	-0.0681	0.0319	-0.136	0.0206
F26D11.10	WBGene00002126	inx-4	-2.37362520120724	0.005514881	-							
F28E10.2	WBGene00017901	-	-2.37262520120724	0.008532363	-							
F42F12.4	WBGene00009636		-2.35153	0.00190506	ENSP00000371525	ENSG00000182551						
Y54E2A.6	WBGene00013191		-2.34785	0.000414199	ENSP00000320252	ENSG00000112249	activating signal cointegrator 1 complex subunit 3	ASCC3	-0.0563	0.117	0.065	0.0926
R13H4.8	WBGene00011266	-	-2.34662520120724	0.0004427129	-							
W08E12.3	WBGene00021084	-	-2.33862520120724	0.001654431	-							
ZK675.4	WBGene00014067	-	-2.33562520120724	0.00002567776	-							
Y38C9A.2	WBGene00000480	cgp-1	-2.33558	0.00204823	ENSP00000216044	ENSG00000100226	GTP binding protein 1	GTPBP1	0.0461	0.0604	-0.00801	0.885
Y47D7A.13	WBGene00021625	-	-2.32962520120724	0.00007294438	-							
T26E4.10	WBGene00012052	-	-2.32662520120724	0.0000992108	-							
T05B4.12	WBGene00020243	-	-2.31762520120724	0.0004545407	-							
T07F10.1	WBGene00011587		-2.31719	0.00554914	ENSP00000265162	ENSG00000138792	glutamyl aminopeptidase (aminopeptidase A)	ENPEP	0.0267	0.246	0.0362	0.174
T05H4.6b	WBGene00020269		-2.31596	0.0013732	ENSP00000353741	ENSG00000120705	eukaryotic translation termination factor 1	ETF1	0.00443	0.937	-0.0266	0.653
ZK337.5	WBGene00003471	mtd-1	-2.30962520120724	0.0002969553	-							
Y43B11AL.2	WBGene00005941	srx-50	-2.30062520120724	0.006214731	-							
C49A9.8	WBGene00016762	ugt-24	-2.2988	0.00887296	ENSP00000282507	ENSG00000168671	UDP glycosyltransferase 3 family, polypeptide A2	UGT3A2				
F16B4.7	WBGene00017509	-	-2.29062520120724	0.009387839	-							
F35A5.3	WBGene00000033	abu-10	-2.28662520120724	0.0003143407	-							

F35B12.7	WBGene00003762	nlp-24	-2.28462520120724	0.0006945305	-								
Y39E4B.1	WBGene00012714	abce-1	-2.28408	0.00463276	ENSP00000296577	ENSG00000164163	ATP-binding cassette, sub-family E (OABP), member 1	ABCE1	-0.111	0.125	-0.212	0.0339	
T08B1.2a	WBGene00006589	tnt-4	-2.28408	0.00901287	ENSP00000356286	ENSG00000118194	troponin T2, cardiac	TNNT2	-0.0381	0.262	0.00515	0.938	
C39E9.10	WBGene00008033		-2.28408	0.000462391	ENSP00000309945	ENSG00000169682	spinster	SPIN1	0.109	7.84e-05	-0.0369	0.517	
C44B11.3	WBGene00003175	mec-12	-2.2804	0.000452944	ENSP00000301071	ENSG00000167552	tubulin, alpha 3	TUBA3	0.0956	0.13	-0.124	0.0685	
T05A1.2	WBGene00000696	col-122	-2.27662520120724	0.00005679396	-								
F10C1.7c	WBGene00002054	ifb-2	-2.27427	0.000678738	ENSP00000327054	ENSG00000176619	lamin B2	LMNB2	0.00659	0.825	0.0167	0.67	
F25A2.1	WBGene00017764	-	-2.27262520120724	0.001484514	-								
F32G8.6	WBGene00000298	cat-4	-2.26323	0.00557488	ENSP00000254299	ENSG00000131979	GTP cyclohydrolase 1 (dopa-responsive dystonia)	GCH1	-0.42	1.09e-06	-0.021	0.794	
R07B1.2	WBGene00002270	lec-7	-2.25762520120724	0.002536269	-								
C46H11.8	WBGene00016732	phat-1	-2.25662520120724	0.00002046879	-								
T05H10.3	WBGene00011508	-	-2.25662520120724	0.0004310377	-								
K06A5.8d	WBGene00019434		-2.25097	0.00411073	ENSP00000358982	ENSG00000085433	WD repeat domain 47	WDR47	-0.625	2.13e-10	-0.0271	0.648	
Y39G10AR.6b	WBGene00021464	ugt-31	-2.25097	0.0033336	ENSP00000282507	ENSG00000168671	UDP glycosyltransferase 3 family, polypeptide A2	UGT3A2					
F39H12.4	WBGene00018215	igcm-1	-2.25062520120724	0.00005803791	-								
F58B4.3	WBGene00010236	-	-2.25062520120724	0.0002092524	-								
D1053.4	WBGene00008369	-	-2.24762520120724	0.001638946	-								
ZC64.4	WBGene00002987	lim-4	-2.24239	0.00188219	ENSP00000362861	ENSG00000106852	LIM homeobox 6	LHX6	-0.0299	0.444	-0.175	0.00617	
C25A11.4a	WBGene00000100	ajm-1	-2.24162520120724	0.0007068794	-								

C54H2.1	WBGene00006368	sym-3	-2.23135	0.00458472	ENSP00000300434	ENSG00000167106	family with sequence similarity 102, member A	FAM102A	-0.113	0.0653	-0.0913	0.0969
B0250.2	WBGene00007119		-2.22862520120724	0.002040473	-							
C46H11.4a	WBGene00002979	lfe-2	-2.22277	0.000347007	ENSP00000272117	ENSG00000143772	inositol 1,4,5-trisphosphate 3-kinase B	ITPKB	0.123	0.0023	-0.0646	0.184
R02F11.2	WBGene00019840		-2.22032	0.00388015	ENSP00000316228	ENSG00000104938	C-type lectin domain family 4, member M	CLEC4M	-0.0752	0.0401	-0.0972	0.0868
C34E11.2	WBGene00007935	-	-2.21862520120724	0.006255655	-							
ZK622.4	WBGene00022782	-	-2.21662520120724	0.0001699732	-							
T01D1.6	WBGene00000034	abu-11	-2.21541	0.000215421	ENSP00000334197	ENSG00000188155						
T02E9.5	WBGene00011383	-	-2.21362520120724	0.0006012732	-							
T26E4.2	WBGene00012044	-	-2.21362520120724	0.002649782	-							
F10D11.6	WBGene00008652		-2.21051	0.00251613	ENSP00000262865	ENSG00000101425	bactericidal/permeability-increasing protein	BPI	-0.0184	0.56	0.0581	0.135
F45E1.7	WBGene00018467	sdpn-1	-2.21051	0.000909816	ENSP00000263246	ENSG00000100266	Protein kinase C and casein kinase substrate in neurons 2	PACSIN2	-0.0126	0.615	-0.0378	0.295
B0302.1b	WBGene00002207	kin-25	-2.20192	0.00231982	ENSP00000371341	ENSG00000061938	tyrosine kinase, non-receptor, 2	TNK2	-0.281	5.23e-06	-0.183	0.018
R07B1.9	WBGene00011081	-	-2.19562520120724	0.001720154	-							
Y102A11A.7	WBGene00022417	-	-2.19462520120724	0.00005171967	-							
F28D1.9	WBGene00009218		-2.19456	0.00308438	ENSP00000300456	ENSG00000167114	solute carrier family 27 (fatty acid transporter), member 4	SLC27A4	0.00502	0.896	-0.225	4.55e-05
C46A5.3	WBGene00000603	col-14	-2.19162520120724	0.0001107282	-							
F59A7.2	WBGene00019090	-	-2.19062520120724	0.001790284	-							
R09B5.9	WBGene00000558	cnc-4	-2.18762520120724	0.0001492112	-							
C54G4.4	WBGene00008314	-	-2.18462520120724	0.00005005449	-							
F22H10.3	WBGene00017727	-	-2.18462520120724	0.001995508	-							
F59C12.1	WBGene00000401	cdh-9	-2.18162520120724	0.002761928	-							

F54D8.1	WBGene00001076	dpy-17	-2.17562520120724	0.0003401555	-							
F22H10.2	WBGene00017726	-	-2.17562520120724	0.004666535	-							
C53B7.1	WBGene00004370	rig-3	-2.17462520120724	0.003425638	-							
C28C12.4	WBGene00016180	-	-2.17262520120724	0.001711885	-							
K06A5.8c	WBGene00019434		-2.17126	0.0016699	ENSP00000358982	ENSG00000085433	WD repeat domain 47	WDR47	-0.625	2.13e-10	-0.0271	0.648
C46H11.9	WBGene00016733	phat-2	-2.17062520120724	0.00002427495	-							
F21E9.2	WBGene00017666	-	-2.17062520120724	0.00006125187	-							
F20A1.10	WBGene00017621	-	-2.16162520120724	0.0001365689	-							
F26E4.3	WBGene00009158	-	-2.15562520120724	0.002390992	-							
T07E3.6b	WBGene00020317	-	-2.14962520120724	0.002194229	-							
F41E6.11	WBGene00018292		-2.14674	0.000383109	ENSP00000218147	ENSG00000085185	BCL6 co-repressor-like 1	BCORL1	0.0511	0.138	-0.103	0.023
F08A10.1c	WBGene00008570		-2.14551	0.00573835	ENSP00000374097	ENSG00000080709	potassium intermediate/small conductance calcium-activated channel, subfamily N, member 2	KCNN2	0.423	3.43e-05	0.0721	0.508
F10C1.2b	WBGene00002053	ifb-1	-2.14306	0.0016249	ENSP00000327054	ENSG00000176619	lamin B2	LMNB2	0.00659	0.825	0.0167	0.67
C05B5.8	WBGene00007324	-	-2.14262520120724	0.0007641239	-							
F53C3.5	WBGene00018749	-	-2.14262520120724	0.005264304	-							
ZK792.4	WBGene00014077		-2.13816	0.000804635	ENSP00000367059	ENSG00000187017	Espin	ESPN	0.0358	0.195	-0.0476	0.252
F26F12.4	WBGene00017835	-	-2.13762520120724	0.0000467304	-							
C16H3.2	WBGene00002272	lec-9	-2.12462520120724	0.000558412	-							
4R79.1	WBGene00003525	nas-6	-2.12344	0.00037164	ENSP00000269202	ENSG00000141434	meprin A, beta	MEP1B	-0.103	0.0769	0.0772	0.0831
Y50E8A.16	WBGene00001817	haf-7	-2.11562520120724	0.004657633	-							
F53F1.5	WBGene00009983	-	-2.10962520120724	0.0002515138	-							
T05H4.7	WBGene00020270	-	-2.10862520120724	0.00008062297	-							
Y51A2D.11	WBGene00013079	-	-2.10362520120724	0.002917621	-							
C06C6.4	WBGene00003653	nhr-63	-2.10362520120724	0.006328448	-							

F14H12.3	WBGene00017471		-2.10137	0.00744984	ENSP00000260356	ENSG00000137801	thrombospondin 1	THBS1	-0.00853	0.819	0.0674	0.164
EGAP7.1	WBGene00001065	dpy-3	-2.09862520120724	0.001304383	-							
C34B7.1	WBGene00007911	-	-2.09662520120724	0.001363729	-							
F55C10.2	WBGene00000727	col-154	-2.09562520120724	0.0003195368	-							
F08B4.2b	WBGene00000397	cdh-5	-2.09362520120724	0.001138466	-							
T05G5.11	WBGene00004097	pqm-96	-2.09362520120724	0.002493727	-							
H19N07.1	WBGene00010405		-2.09278	0.00878548	ENSP00000219627	ENSG00000103342	G1 to S phase transition 1 /// G1 to S phase transition 1	GSPT1	-0.0146	0.809	-0.237	0.00962
F48A11.1	WBGene00000497	chs-2	-2.08962520120724	0.005763964	-							
Y39A1A.7	WBGene00012646		-2.08862520120724	0.002794088	-							
F41G3.10	WBGene00018303	-	-2.08662520120724	0.0003871668	-							
Y58A7A.5	WBGene00021979	-	-2.07862520120724	0.003037723	-							
T26C5.3a	WBGene00012033		-2.07684	0.0081599	ENSP00000352456	ENSG00000169359	solute carrier family 33 (acetyl-CoA transporter), member 1	SLC33A1	0.161	0.00639	-0.058	0.379
C44C10.9	WBGene00008089	-	-2.07662520120724	0.0005988358	-							
B0416.2	WBGene00015178	-	-2.07662520120724	0.005232336	-							
T10B11.9	-	-	-2.07362520120724	0.0003592767	-							
T05G5.12	WBGene00000932	dao-6	-2.07062520120724	0.001293575	-							
Y110A2AL.2	WBGene00022439	-	-2.06862520120724	0.001145427	-							
F43H9.2a	WBGene00018398	sptl-2	-2.06703	0.000971279	ENSP00000216484	ENSG00000100596	serine palmitoyltransferase, long chain base subunit 2	SPTLC2	0.103	0.147	-0.0503	0.508
T05C1.1	WBGene00020248	-	-2.06662520120724	0.0007934975	-							
K04H4.2b	WBGene00010573	-	-2.05662520120724	0.0001073049	-							
C24A1.1	WBGene00016028	flp-24	-2.05562520120724	0.001017614	-							
M02G9.2	WBGene00010831	-	-2.05362520120724	0.001123195	-							
C33G3.3	WBGene00007903	lgc-21	-2.05062520120724	0.000123965	-							
Y62F5A.9	WBGene00013387	-	-2.05062520120724	0.0005096181	-							
C09D8.1b	WBGene00004215	ptp-3	-2.04986	0.00654241	ENSP00000347106	ENSG00000105426	Protein tyrosine phosphatase, receptor type, S	PTPRS	0.00607	0.904	-0.229	0.00862

T20B5.1	WBGene00000161	apa-2	-2.04251	0.00356714	ENSP00000327694	ENSG00000183020	adaptor-related protein complex 2, alpha 2 subunit	AP2A2	-0.137	0.000745	-0.197	0.0136
F43B10.2	WBGene00018374	tag-343	-2.03762520120724	0.0009891202	-							
C26F1.1a	WBGene00016146	-	-2.03662520120724	0.0001213554	-							
C33A12.3	WBGene00007882		-2.0327	0.0080132	ENSP00000281146	ENSG00000151470						
C25F6.7b	WBGene00016106		-2.02166	0.000653983	ENSP00000322730	ENSG00000135916	brain protein I3	BRI3	0.00915	0.87	-0.0171	0.809
Y92H12A.2	WBGene00022358		-2.01798	0.0075816	ENSP00000256832	ENSG00000049759	neural precursor cell expressed, developmentally down-regulated 4-like	NEDD4L	-0.36	5.26e-10	-0.31	0.00246
T20G5.8	WBGene00011870	-	-2.00762520120724	0.0006230255	-							
DY3.5	WBGene00004115	pqn-26	-2.00662520120724	0.001686449	-							
Y37D8A.6	WBGene00012548		-2.00204	0.00479569	ENSP00000351975	ENSG00000089486	chromosome 16 open reading frame 5	C16orf5	-0.104	0.0515	-0.00662	0.904
T01E8.8	WBGene00011335	-	-2.00062520120724	0.004260303	-							
C52B9.9	WBGene00003179	mec-18	-1.99962520120724	0.001102175	-							
F42G9.9b	WBGene00004212	ptl-1	-1.99713	0.000512114	ENSP00000353375	ENSG00000047849	microtubule-associated protein 4	MAP4	0.0265	0.522	-0.000668	0.991
C18B2.5b	WBGene00015956	-	-1.99062520120724	0.0002699214	-							
C03B1.7	WBGene00015378	-	-1.99062520120724	0.00942084	-							
K11D12.3	WBGene00005655	srr-4	-1.98962520120724	0.0007322577	-							
C04F6.3	WBGene00000503	cht-1	-1.98732	0.00149482	ENSP00000356198	ENSG00000133063	chitinase 1 (chitotriosidase)	CHIT1	0.0251	0.574	0.105	0.0851
B0564.9	WBGene00007208	-	-1.97762520120724	0.004377448	-							
Y71F9AR.1	WBGene00000237	bam-2	-1.97762520120724	0.005048164	-							
ZC168.1	WBGene00003568	ncx-3	-1.97662520120724	0.002196161	-							
F53A9.3	WBGene00018726	-	-1.97362520120724	0.0001146617	-							
K04H4.2a	WBGene00010573	-	-1.97362520120724	0.0003627146	-							
C37C3.13	WBGene00016505	-	-1.97162520120724	0.004665318	-							

F39D8.1b	WBGene00004123	pqn-36	-1.97062520120724	0.0004710579	-								
Y41D4A.1	WBGene00021504	-	-1.96862520120724	0.0002092524	-								
T24C12.4	WBGene00020766	-	-1.96662520120724	0.0001238028	-								
F42C5.7	WBGene00001713	grl-4	-1.96662520120724	0.0001791611	-								
C24A1.2	WBGene00016029		-1.96525	0.000539325	ENSP00000 257831	ENSG000 00135373	Ets homologous factor	EHF	0.0641	0.0523	-0.0016	0.959	
T05F1.9	WBGene00011494	-	-1.96162520120724	0.005114483	-								
T27C4.1	WBGene00020854	-	-1.96062520120724	0.002885553	-								
T25D10.4	WBGene00020801	-	-1.95862520120724	0.0004051728	-								
F49A5.8	WBGene00005770	srw-23	-1.95862520120724	0.002794088	-								
D1009.1a	WBGene00017012		-1.95544	0.00240757	ENSP00000 300456	ENSG000 00167114	solute carrier family 27 (fatty acid transporter), member 4	SLC27A4	0.00502	0.896	-0.225	4.55e-05	
F25G6.7a	WBGene00017798	-	-1.95462520120724	0.000149502	-								
F55A12.7	WBGene00000150	apm-1	-1.94562520120724	0.000616257	-								
C24G6.2b	WBGene00016059	-	-1.94562520120724	0.0008499568	-								
B0334.6	WBGene00007146		-1.9444	0.000516564	ENSP00000 370779	ENSG000 00180269							
F43G6.8	WBGene00009660	-	-1.94162520120724	0.0003887195	-								
F46G11.1	WBGene00018514		-1.93582	0.00242075	ENSP00000 309595	ENSG000 00107815	progressive external ophthalmoplegi a 1	PEO1	0.0314	0.532	0.122	0.00646	
C50B8.4	WBGene00008226	-	-1.93562520120724	0.0001561946	-								
Y73B6BR.1a	WBGene00004169	pqn-89	-1.93562520120724	0.002125453	-								
W02F12.2	WBGene00020947		-1.93337	0.00256464	ENSP00000 342609	ENSG000 00177076	N-acylsphingosine amidohydrolase 3-like	ASAH3L					
F01G10.6	WBGene00008509	-	-1.93162520120724	0.0004006195	-								
T05A1.1b	WBGene00003808	npr-2	-1.92562520120724	0.002540478	-								
Y38H6C.3	WBGene00012616	-	-1.92262520120724	0.0002649852	-								
ZK512.1	WBGene00013982	-	-1.91862520120724	0.0001396519	-								

T08H4.3	WBGene00020368	ast-1	-1.91743	0.000936269	ENSP00000353344	ENSG00000157557	v-ets erythroblastosis virus E26 oncogene homolog 2 (avian)	ETS2	0.156	0.139	-0.396	0.00103
T18D3.4	WBGene00003514	myo-2	-1.91497	0.00547204	ENSP00000348634	ENSG00000197616	Myosin heavy chain, cardiac muscle alpha isoform (MyHC-alpha)	MYH6	0.0246	0.21	-0.0157	0.595
K06A1.1	WBGene00019424		-1.91252	0.00795149	ENSP00000201031	ENSG00000087510	transcription factor AP-2 gamma (activating enhancer binding protein 2 gamma)	TFAP2C	0.012	0.633	0.0368	0.311
F58B4.6	WBGene00010239	-	-1.91162520120724	0.0007296366	-							
K07D8.1	WBGene00003497	mup-4	-1.9113	0.0036599	ENSP00000325527	ENSG00000166147	fibrillin 1 (Marfan syndrome)	FBN1	0.0636	0.143	0.00268	0.957
T06E4.8	WBGene00011535	-	-1.90862520120724	0.0002092524	-							
Y54E10A.16a	WBGene00021834	-	-1.90862520120724	0.000769897	-							
C49G7.5	WBGene00016783	-	-1.90762520120724	0.003522872	-							
W03F9.4	WBGene00021002	-	-1.90562520120724	0.0004603871	-							
W02G9.4	WBGene00020954	-	-1.90462520120724	0.003774393	-							
C42D8.8a	WBGene00000149	apl-1	-1.90271	0.00191442	ENSP00000221891	ENSG00000105290	amyloid beta (A4) precursor-like protein 1	APLP1	-0.258	0.00208	-0.134	0.0344
C39D10.6	WBGene00016533	-	-1.90062520120724	0.0006763265	-							
F52H3.5	WBGene00009947		-1.90026	0.00140831	ENSP00000307640	ENSG00000172425						
F53A9.7	WBGene00018730	-	-1.89862520120724	0.003830853	-							
T23F11.3	WBGene00011955	cdka-1	-1.89658	0.00149346	ENSP00000318486	ENSG00000176749	cyclin-dependent kinase 5, regulatory subunit 1 (p35)	CDK5R1	-0.131	0.00933	-0.194	0.022
F13E9.11	WBGene00008760	-	-1.89562520120724	0.0005951081	-							
R02E12.5	WBGene00019829	-	-1.88962520120724	0.004132809	-							

F52D10.2	WBGene00009930		-1.888	0.00142099	ENSP00000371397	ENSG00000185112	family with sequence similarity 43, member A	FAM43A	0.125	0.00636	-0.145	0.195
T10B10.6	WBGene00011683	phat-6	-1.88762520120724	0.003307538	-							
F53F1.4	WBGene00009982	-	-1.88462520120724	0.0002743055	-							
C54G7.3a	WBGene00002983	lgx-1	-1.88162520120724	0.0003040117	-							
Y73F8A.5	WBGene00013518		-1.87328	0.0044768	ENSP00000234923	ENSG00000116299	maba1	KIAA1324	-0.137	0.00006	-0.111	0.0204
Y70C5C.6b	WBGene00003702	nhr-112	-1.86962520120724	0.0006800552	-							
F26A10.1	WBGene00017813	-	-1.86462520120724	0.002188633	-							
Y39E4B.13	WBGene00012721		-1.86462520120724	0.007060118	-							
T08B2.4	WBGene00020345	-	-1.86262520120724	0.004884213	-							
C49F8.3	WBGene00008215	-	-1.86162520120724	0.0008107688	-							
B0462.1	WBGene00007181		-1.86162520120724	0.002553507	-							
R07H5.11	WBGene00011131	-	-1.85962520120724	0.0076038	-							
W01A11.4	WBGene00002273	lec-10	-1.85862520120724	0.001718901	-							
F26G1.5	WBGene00017841	-	-1.85662520120724	0.0003741234	-							
F53B3.3	WBGene00018742	-	-1.85662520120724	0.0005170232	-							
F15D4.8	WBGene00001459	flp-16	-1.85662520120724	0.006793188	-							
T05G5.10	WBGene00002064	iff-1	-1.85489	0.000751236	ENSP00000336702	ENSG00000132507	eukaryotic translation initiation factor 5A	EIF5A	-0.0503	0.421	0.0544	0.565
F53B3.5	WBGene00018743	-	-1.85462520120724	0.0002092524	-							
F47A4.1b	WBGene00009799	-	-1.84862520120724	0.003053015	-							
C38C6.6	WBGene00007999	tag-297	-1.84862520120724	0.00327412	-							
Y110A7A.2	WBGene00022454		-1.8463	0.00121813	ENSP00000332468	ENSG00000131323	TNF receptor-associated factor 3	TRAF3	-0.161	0.019	-0.0854	0.195
K06H7.9	WBGene00019460	idi-1	-1.8463	0.00832285	ENSP00000370748	ENSG00000067064	isopentenyl-diphosphate delta isomerase	IDI1	0.0567	0.0735	-0.0729	0.118
F58A6.10	WBGene00019027	srb-12	-1.84562520120724	0.00098953	-							
E03D2.1	WBGene00003751	nlp-13	-1.84262520120724	0.005515253	-							
F48C11.3	WBGene00003741	nlp-3	-1.84162520120724	0.009667947	-							
F29C12.1	WBGene00004120	pqn-32	-1.83962520120724	0.001357627	-							
F53F4.13	WBGene00009995	-	-1.83862520120724	0.0004067526	-							
B0280.7	WBGene00015103	-	-1.83662520120724	0.0005496379	-							
T16G1.1	WBGene00004150	pqn-67	-1.83562520120724	0.001021615	-							

B0303.7	WBGene00015128		-1.83527	0.00748734	ENSP00000302913	ENSG00000109686	SH3 domain protein D19	SH3D19	0.139	0.00564	-0.0822	0.268
F08A10.1b	WBGene00008570		-1.83527	0.00576669	ENSP00000374097	ENSG00000080709	potassium intermediate/small conductance calcium-activated channel, subfamily N, member 2	KCNN2	0.423	3.43e-05	0.0721	0.508
F33D4.3	WBGene00001456	flp-13	-1.83362520120724	0.001269682	-							
T13H5.2	WBGene00011756	ctg-2	-1.83362520120724	0.005070931	-							
F53A9.9	WBGene00018732	-	-1.83262520120724	0.006033266	-							
W07E11.4	WBGene00012327	-	-1.82662520120724	0.001181518	-							
W02D9.5	WBGene00012211	-	-1.82362520120724	0.001981896	-							
C25B8.1a	WBGene00002233	kqt-1	-1.823	0.00634552	ENSP00000347853	ENSG00000185760	Potassium voltage-gated channel, KQT-like subfamily, member 5	KCNQ5	-0.104	0.00106	0.0391	0.375
C25A11.4b	WBGene00000100	ajm-1	-1.81962520120724	0.00148536	-							
C10F3.2	WBGene00000979	dhs-16	-1.8181	0.000743521	ENSP00000318631	ENSG00000025423	hydroxysteroid (17-beta) dehydrogenase 6	HSD17B6	-0.0382	0.633	0.305	0.0044
F31F6.5	WBGene00000902	daf-6	-1.81562520120724	0.001978036	-							
F45H7.4	WBGene00004183	prk-2	-1.81442	0.00211487	ENSP00000353824	ENSG00000198355	pim-3 oncogene	PIM3	0.126	0.0602	-0.0209	0.685
ZK1248.2	WBGene00000650	col-74	-1.81362520120724	0.001388461	-							
T12A2.16a	WBGene00001989	hot-4	-1.81362520120724	0.002254155	-							
C13D9.1	WBGene00005657	srr-6	-1.81062520120724	0.001390333	-							
K06A5.8a	WBGene00019434		-1.80461	0.000938087	ENSP00000358982	ENSG00000085433	WD repeat domain 47	WDR47	-0.625	2.13e-10	-0.0271	0.648
F11D5.1b	WBGene00017380	-	-1.80362520120724	0.001430322	-							
C15H9.11	WBGene00015805	-	-1.80262520120724	0.002638225	-							
W04G3.8	WBGene00012261	-	-1.79662520120724	0.003077935	-							
Y75B8A.20	WBGene00001724	grl-15	-1.79562520120724	0.0005170232	-							
F39G3.5a	WBGene00018210	-	-1.78962520120724	0.0003635001	-							
Y66D12A.24	WBGene00013448	-	-1.78762520120724	0.0004897584	-							

ZK970.7	WBGene00014173	-	-1.78762520120724	0.002188633	-								
F26E4.7a	WBGene00009162	-	-1.78062520120724	0.0005350282	-								
ZC334.2	WBGene00002113	ins-30	-1.77562520120724	0.003477871	-								
C25H3.1	WBGene00016111	-	-1.76562520120724	0.008113767	-								
T06E4.9	WBGene00011536	-	-1.76262520120724	0.0004006195	-								
AH9.2	WBGene00000797	crn-4	-1.75762520120724	0.004966369	-								
M195.3	WBGene00000135	amt-3	-1.75662520120724	0.001581956	-								
Y64G10A.7	WBGene00013416		-1.75556	0.00951475	ENSP00000348982	ENSG00000162591	EGF-like-domain, multiple 3	EGFL3	0.139	0.000192	0.0526	0.27	
K10D6.2c	WBGene00010742	-	-1.75362520120724	0.0005411367	-								
F53B1.4	WBGene00018737	-	-1.75362520120724	0.004001721	-								
T28C6.4	WBGene00000691	col-117	-1.75062520120724	0.0004157021	-								
B0412.2	WBGene00000903	daf-7	-1.74943	0.0081599	ENSP00000257868	ENSG00000135414	growth differentiation factor 11	GDF11	-0.0199	0.594	0.0839	0.0681	
F35H10.4	WBGene00006914	vha-5	-1.74662520120724	0.002683675	-								
F58G1.4	WBGene00010266	-	-1.74362520120724	0.0002864788	-								
Y45F10A.5	WBGene00003755	nlp-17	-1.73962520120724	0.0007496986	-								
C45B2.2	WBGene00016659	-	-1.73762520120724	0.001593394	-								
F47B8.1	WBGene00009802	-	-1.73762520120724	0.002390992	-								
F49E12.10	WBGene00009903		-1.73594	0.00583134	ENSP00000320604	ENSG00000170271	chromosome 5 open reading frame 4	C5orf4	0.144	6.57e-06	-0.000859	0.989	
T06E4.10	WBGene00011537	-	-1.73462520120724	0.0007496986	-								
ZK154.1	WBGene00022665	-	-1.73462520120724	0.001520771	-								
C34H4.4a	WBGene00000681	col-107	-1.73262520120724	0.0006611196	-								
F38B6.3	WBGene00018173	-	-1.73162520120724	0.001257172	-								
F53F8.1	WBGene00009998		-1.72981	0.00098086	ENSP00000366330	ENSG00000119138	Kruppel-like factor 9	KLF9	0.0652	0.146	0.00925	0.861	
F12B6.1	WBGene00000020	abt-2	-1.72858	0.00873117	ENSP00000363868	ENSG00000165029	ATP-binding cassette, sub-family A (ABC1), member 1	ABCA1	0.0535	0.208	0.0904	0.0654	
F53A9.8	WBGene00018731	-	-1.72762520120724	0.001654431	-								
Y76B12C.1	WBGene00022295	-	-1.72662520120724	0.00032653	-								
T06E4.11	WBGene00004146	pqn-63	-1.72362520120724	0.0004020331	-								
C10C5.2	WBGene00007506	-	-1.72362520120724	0.008183025	-								
B0213.3	WBGene00003766	nlp-28	-1.72162520120724	0.0008835172	-								
ZK54.1	WBGene00022647	-	-1.71662520120724	0.0007785597	-								

T05B4.3	WBGene00020237	phat-4	-1.71462520120724	0.0005614246	-							
T24C4.4	WBGene00020760	-	-1.71462520120724	0.00139378	-							
C37E2.4	WBGene00000457	ceh-36	-1.71362520120724	0.002613426	-							
T03F1.11	WBGene00020192	-	-1.71162520120724	0.001445365	-							
T19H5.4	WBGene00011849	-	-1.71162520120724	0.002223921	-							
T22H2.5b	WBGene00011935	scrm-1	-1.71019	0.00265205	ENSP00000 345494	ENSG000 00188313	phospholipid scramblase 1	PLSCR1	0.331	0.0526	0.133	0.253
C45B2.7	WBGene00004219	ptr-4	-1.70662520120724	0.002615866	-							
C23H3.9c	WBGene00016022	-	-1.70362520120724	0.0003143407	-							
F48B9.4	WBGene00018590	nlp-37	-1.70062520120724	0.002390992	-							
C09F12.1	WBGene00000522	clc-1	-1.70062520120724	0.002743043	-							
M88.4	WBGene00010907	-	-1.69862520120724	0.001830344	-							
C25B8.6	WBGene00003710	nhr-120	-1.69862520120724	0.005305082	-							
F35H10.10	WBGene00018073	-	-1.69662520120724	0.0003674862	-							
F40E10.2	WBGene00004950	sox-3	-1.69547	0.00145089	ENSP00000 359567	ENSG000 00134595	SRY (sex determining region Y)-box 3	SOX3	0.185	2.78e-07	0.0289	0.557
ZK899.2	WBGene00014141	-	-1.69462520120724	0.0005131538	-							
K11G9.1	WBGene00019652	-	-1.69162520120724	0.002308995	-							
K02E10.6	WBGene00019320	-	-1.69062520120724	0.0006033311	-							
R12E2.7	WBGene00020033	-	-1.68862520120724	0.0007191506	-							
F53C3.4	WBGene00018748	-	-1.68862520120724	0.005349995	-							
K08E5.2b	WBGene00003519	nac-3	-1.68762520120724	0.006576951	-							
W08E12.6	WBGene00021087	-	-1.68462520120724	0.0005614246	-							
Y75B7AR.1	WBGene00022287	-	-1.68162520120724	0.0007191506	-							
F14D7.8	WBGene00008795	-	-1.68062520120724	0.001177434	-							
K01C8.3a	WBGene00006562	tdc-1	-1.6783	0.00593693	ENSP00000 339928	ENSG000 00132437	Dopa decarboxylase (aromatic L-amino acid decarboxylase)	DDC	-0.0553	0.152	-0.0549	0.277
ZK1320.5	WBGene00014255	-	-1.67562520120724	0.000815084	-							
C27A7.8	WBGene00007758	-	-1.67462520120724	0.0007326097	-							
T28B4.4	WBGene00020887	-	-1.67362520120724	0.006563451	-							
F47B8.2	WBGene00009803	-	-1.67162520120724	0.001513591	-							
Y32F6B.2	WBGene00003570	ncx-5	-1.67162520120724	0.008313441	-							
K10D3.4	WBGene00010738	-	-1.67062520120724	0.000501023	-							

H19J13.1	WBGene00010403		-1.66972	0.00608286	ENSP00000296358	ENSG00000163982	otopetrin 1	OTOP1				
M03A1.7	WBGene00000928	dao-2	-1.66862520120724	0.0009204087	-							
F54C1.9	WBGene00006058	sst-20	-1.66862520120724	0.0009249432	-							
ZK180.5a	WBGene00022679	-	-1.66862520120724	0.004001721	-							
T07D1.3	WBGene00020302	-	-1.66562520120724	0.0004585217	-							
C32F10.4	WBGene00016332	-	-1.66562520120724	0.001520771	-							
Y38F2AL.1	WBGene00021415	nsy-4	-1.66562520120724	0.001678689	-							
F02C12.1	WBGene00008515	-	-1.66462520120724	0.006186415	-							
W02A2.5	WBGene00012190	-	-1.66362520120724	0.004543798	-							
DC2.3	WBGene00017080	-	-1.66162520120724	0.001520771	-							
C05E11.8	WBGene00001455	flp-12	-1.66162520120724	0.00554411	-							
C49C3.12	WBGene00008202	-	-1.65762520120724	0.004178784	-							
T25F10.3	WBGene00020806	-	-1.65562520120724	0.002515984	-							
Y55F3C.7b	WBGene00021950	-	-1.65462520120724	0.000605359	-							
T06D8.10	WBGene00011530	-	-1.65062520120724	0.005223131	-							
T04G9.2	WBGene00003534	nas-15	-1.64762520120724	0.0007191506	-							
Y46H3A.4	WBGene00021600	-	-1.64562520120724	0.0008773175	-							
T23G5.5	WBGene00000934	dat-1	-1.64519	0.0039286	ENSP00000219833	ENSG00000103546	solute carrier family 6 (neurotransmitter transporter, noradrenalin), member 2	SLC6A2	0.0503	0.113	0.0195	0.608
Y37D8A.16	WBGene00012554	-	-1.64162520120724	0.0008315449	-							
F59A3.1	WBGene00019074		-1.64152	0.00904096	ENSP00000225719	ENSG00000108582	carboxypeptidase D	CPD	0.06	0.323	-0.0522	0.328
Y54G2A.4	WBGene00021870		-1.63906	0.00199746	ENSP00000332624	ENSG00000182544						
T01D1.4	WBGene00020149	-	-1.63862520120724	0.005597345	-							
K07D4.7b	WBGene00019487	tag-218	-1.63784	0.00487687	ENSP00000264051	ENSG00000066248	neuronal guanine nucleotide exchange factor	NGEF	-0.997	9.72e-09	-0.831	0.000244
F41H10.8	WBGene00001244	elo-6	-1.63262520120724	0.0005258397	-							
ZK896.6	WBGene00014137	-	-1.63262520120724	0.001235662	-							
Y79H2A.1	WBGene00000273	brp-1	-1.63262520120724	0.00671184	-							
Y43F8B.3	WBGene00012814	-	-1.63162520120724	0.000497302	-							
C40H5.4	WBGene00008047	lact-7	-1.63162520120724	0.001116129	-							

C06C6.5b	WBGene00003640	nhr-50	-1.63162520120724	0.001201967	-								
W07B8.3	WBGene00021071	-	-1.62962520120724	0.0008773175	-								
K08H2.1	WBGene00004827	skr-21	-1.62562520120724	0.005430889	-								
C33A12.4	WBGene00007883	-	-1.62462520120724	0.00113621	-								
F46F6.4	WBGene00001122	dyf-6	-1.62435	0.00702525	ENSP00000264020	ENSG00000118096	chromosome 11 open reading frame2	C11orf2	-0.255	0.000619	-0.0896	0.152
K07E3.4a	WBGene00019492		-1.62435	0.00453537	ENSP00000216605	ENSG00000100714	methylenetetrahydrofolate dehydrogenase (NADP+ dependent) 1, methenyltetrahydrofolate cyclohydrolase, formyltetrahydrofolate synthetase	MTHFD1	-0.453	1.28e-09	-0.0196	0.772
W04G3.1	WBGene00012255	-	-1.62262520120724	0.002861478	-								
F57G12.2	WBGene00010222	-	-1.62262520120724	0.003093815	-								
C04C3.5	WBGene00001119	dyf-3	-1.6219	0.00422508	ENSP00000344392	ENSG00000103351	clusterin associated protein 1	CLUAP1	-0.0256	0.533	-0.0895	0.0916
Y11D7A.4	WBGene00004281	rab-28	-1.62067	0.00147487	ENSP00000340079	ENSG00000157869	RAB28, member RAS oncogene family	RAB28	0.0562	0.0429	-0.046	0.379
T23F4.2	WBGene00020740	-	-1.61962520120724	0.002536269	-								
VH15N14R.1	WBGene00012151	-	-1.61862520120724	0.0008574562	-								
D1054.9b	WBGene00008376	-	-1.61762520120724	0.0005011512	-								
F57B1.5	WBGene00010188	-	-1.61462520120724	0.006845192	-								
C50D2.2	WBGene00016806		-1.60963	0.00152992	ENSP00000372390	ENSG00000099960	solute carrier family 7 (cationic amino acid transporter, y+ system), member 4	SLC7A4	-0.0134	0.77	0.0229	0.712
K01D12.5	WBGene00010464	-	-1.60662520120724	0.0007649613	-								
Y37E11AL.5	WBGene00021361	-	-1.60662520120724	0.001138466	-								

ZC247.3	WBGene00003000	lin-11	-1.60595	0.00131882	ENSP00000254457	ENSG00000132130	LIM homeobox 1	LHX1	-0.0566	0.211	-0.0272	0.537
T06C12.10	WBGene00011517	cgt-1	-1.60473	0.00360876	ENSP00000363397	ENSG00000148154	UDP-glucose ceramide glucosyltransferase	UGCG	-0.307	0.00439	-0.161	0.0603
C54G7.3b	WBGene00002983	lgx-1	-1.60462520120724	0.0007326097	-							
T12A2.16b	WBGene00001989	hot-4	-1.60462520120724	0.001102175	-							
C50F2.7	WBGene00016840	-	-1.60262520120724	0.0006116005	-							
K08A8.2a	WBGene00004949	sox-2	-1.60228	0.00686073	ENSP00000265007	ENSG00000039600	SRY (sex determining region Y)-box 30	SOX30	0.00622	0.727	0.00836	0.748
M195.2	WBGene00010948	-	-1.60162520120724	0.0005951081	-							
C44C1.3	WBGene00003563	ncs-1	-1.60105	0.0018771	ENSP00000361475	ENSG00000107130	Frequenin homolog (Drosophila)	FREQ	-0.0768	0.033	0.0152	0.736
C50D2.1	WBGene00016805	-	-1.59962520120724	0.002314883	-							
Y67A10A.8	WBGene00013457		-1.59737	0.0040857	ENSP00000344203	ENSG00000163291	progestin and adipoQ receptor family member III	PAQR3	-0.0385	0.561	0.0887	0.255
F20C5.4	WBGene00008969	-	-1.59662520120724	0.0007326097	-							
C01G12.10	WBGene00007250	nspb-8	-1.59662520120724	0.003529543	-							
F48F7.1	WBGene00000105	alg-1	-1.59614	0.00479998	ENSP00000220592	ENSG00000123908	Eukaryotic translation initiation factor 2C 2 (eIF2C 2) (eIF-2C 2) (Argonaute-2) (Slicer protein) (PAZ Piwi domain protein) (PPD)	EIF2C2	0.194	0.00337	0.0679	0.123
F09E10.6	WBGene00017296	-	-1.59562520120724	0.0004100157	-							
F39H2.1	WBGene00009562	flp-22	-1.59562520120724	0.009683108	-							

VF36H2L.1	WBGene00000147	aph-1	-1.59369	0.00177258	ENSP00000261879	ENSG00000138613	anterior pharynx defective 1 homolog B (C. elegans) /// anterior pharynx defective 1 homolog B (C. elegans)	APH1B	-0.00867	0.829	-0.00845	0.86
T05H4.4	WBGene00020267		-1.59369	0.00444812	ENSP00000338461	ENSG00000100243	diaphorase (NADH) (cytochrome b-5 reductase)	DIA1	0.211	2.09e-05	0.0501	0.468
T23H4.1	WBGene00002142	inx-20	-1.59162520120724	0.0006491561	-							
Y53F4B.27	WBGene00013173	-	-1.58562520120724	0.000558412	-							
C36H8.2	WBGene00002128	inx-6	-1.58162520120724	0.0005614246	-							
F18G5.3	WBGene00001674	gpa-12	-1.58143	0.00675292	ENSP00000275364	ENSG00000146535	guanine nucleotide binding protein (G protein) alpha 12	GNA12	0.247	3.7e-05	0.246	0.000745
T14D7.2	WBGene00011768	-	-1.57662520120724	0.006055877	-							
R08F11.5	WBGene00005754	srw-7	-1.57562520120724	0.002067467	-							
F14B8.5b	WBGene00017447	-	-1.57362520120724	0.001467469	-							
F25E5.3	WBGene00017784	-	-1.57362520120724	0.005767802	-							
F10D7.2	WBGene00017339		-1.57284	0.00159147	ENSP00000332646	ENSG00000109736	Tetracycline transporter-like protein	TETRAN	0.0717	0.0764	0.133	0.077
C18A11.2	WBGene00015948		-1.57162520120724	0.004692993	-							
R102.2	WBGene00011289	-	-1.56962520120724	0.0005614246	-							
R74.8a	WBGene00011282	-	-1.56962520120724	0.003628649	-							
C31H2.1a	WBGene00016292		-1.56917	0.00250095	ENSP00000293970	ENSG00000162065	KIAA1171 protein	KIAA1171	-0.171	0.00913	-0.0385	0.636
ZK1128.5	WBGene00044072	tag-246	-1.56549	0.00650846	ENSP00000262188	ENSG00000082014	SWI/SNF related, matrix associated, actin dependent regulator of chromatin, subfamily d, member 3	SMARCD3	-0.43	8.79e-08	0.0345	0.652

F30B5.4	WBGene00017934		-1.56426	0.00477655	ENSP00000 297438	ENSG000 00164823	Chromosome 8 open reading frame 1	C8orf1	0.019	0.502	0.0531	0.173
Y57G11C.24d	WBGene00001330	eps-8	-1.56181	0.00280665	ENSP00000 201647	ENSG000 00131037	EPS8-like 1	EPS8L1	-0.0172	0.644	0.00362	0.963
T23B3.2	WBGene00020710	-	-1.55862520120724	0.006113657	-							
Y73F8A.19	WBGene00006441	tag-64	-1.55662520120724	0.009078428	-							
K04B12.1	WBGene00004048	plx-2	-1.55562520120724	0.001611863	-							
F22E12.3	WBGene00009060		-1.55362520120724	0.004178192	-							
C29H12.6	WBGene00016237	-	-1.55262520120724	0.001896837	-							
Y77E11A.2	WBGene00022307	-	-1.55062520120724	0.0006791063	-							
C27H2.3	-	-	-1.54962520120724	0.003728648	-							
R05D7.5	WBGene00011030	-	-1.54862520120724	0.0006624761	-							
W09C5.5	WBGene00012352	-	-1.54862520120724	0.0008786058	-							
T28F4.2	WBGene00012137	asic-2	-1.54662520120724	0.002593202	-							
ZC434.9b	WBGene00013895		-1.54587	0.00232879	ENSP00000 222481	ENSG000 00158516	carboxypeptidase A2 (pancreatic)	CPA2	0.0847	0.0564	-0.00519	0.928
C23H5.2b	WBGene00016024	-	-1.54562520120724	0.0006279703	-							
F22B5.3	WBGene00009041	cut-3	-1.54362520120724	0.000710121	-							
C47D2.1	WBGene00016738	-	-1.54362520120724	0.001001762	-							
C49G7.10	WBGene00016788	-	-1.54362520120724	0.006102265	-							
D1005.5	WBGene00016999	-	-1.54262520120724	0.0009704376	-							
F53A9.2	WBGene00018725	-	-1.54262520120724	0.001399601	-							
C07A12.7a	WBGene00015561		-1.54096	0.00318634	ENSP00000 368818	ENSG000 00175662	target of myb1-like 2 (chicken)	TOM1L2	-0.0351	0.299	-0.148	0.0174
T26C12.4	WBGene00001548	gcy-23	-1.54062520120724	0.0007496986	-							
Y39B6A.5	WBGene00012668	-	-1.54062520120724	0.009658444	-							
Y18H1A.9	WBGene00021213		-1.53606	0.00310133	ENSP00000 222481	ENSG000 00158516	carboxypeptidase A2 (pancreatic)	CPA2	0.0847	0.0564	-0.00519	0.928
C28A5.4	WBGene00000463	ceh-43	-1.53238	0.0048755	ENSP00000 222598	ENSG000 00105880	distal-less homeo box 5	DLX5	-0.186	7.65e-05	0.0644	0.154
W02D7.3	WBGene00020939	-	-1.53062520120724	0.002390992	-							
T28F4.1	WBGene00012136	-	-1.52962520120724	0.001371633	-							
Y40H7A.4	WBGene00012744	-	-1.52362520120724	0.0009000061	-							
T19A5.3a	WBGene00020554	-	-1.52362520120724	0.004692993	-							
C53C9.2	WBGene00016898	-	-1.52262520120724	0.00848387	-							

C48D5.1	WBGene00003605	nhr-6	-1.52134	0.00256409	ENSP00000344479	ENSG00000153234	Nuclear receptor subfamily 4, group A, member 2	NR4A2	0.177	0.0486	-0.0141	0.855
ZK678.5	WBGene00006950	wrt-4	-1.52062520120724	0.001197031	-							
C44H4.1	WBGene00008093	-	-1.51962520120724	0.001520771	-							
Y113G7B.11	WBGene00013761	-	-1.50762520120724	0.003539389	-							
T13H5.3	WBGene00011757	-	-1.50662520120724	0.0007035365	-							
C02F5.8	WBGene00006627	tsp-1	-1.50662520120724	0.003728648	-							
Y37D8A.23c	WBGene00006762	unc-25	-1.50417	0.00930084	ENSP00000350928	ENSG00000128683	glutamate decarboxylase 1 (brain, 67kDa)	GAD1	-0.673	2.85e-07	-0.796	0.00244
C42C1.2	WBGene00016580		-1.50172	0.00541057	ENSP00000228705	ENSG00000111110	protein phosphatase 1H (PP2C domain containing)	PPM1H	-0.0239	0.472	-0.172	0.00342
K02A4.2	WBGene00001681	gpc-1	-1.50172	0.0026157	ENSP00000360021	ENSG00000172380	guanine nucleotide binding protein (G protein), gamma 12	GNG12	0.316	3.18e-07	0.139	0.0061
Y47D3A.2	WBGene00012923	fbxa-128	-1.50062520120724	0.001568062	-							
Y57G11C.45	WBGene00013334	-	-1.49962520120724	0.001304383	-							
C14F5.2	WBGene00006980	zig-3	-1.49962520120724	0.00766301	-							
W08F4.6	WBGene00021095	mlt-8	-1.49662520120724	0.00467905	-							
Y110A2AL.8b	WBGene00004210	ptc-3	-1.49559	0.00489958	ENSP00000332353	ENSG00000185920	patched homolog (Drosophila)	PTCH1	0.00986	0.784	0.122	0.021
Y54G2A.25c	WBGene00002243	lad-2	-1.49559	0.00213879	ENSP00000359077	ENSG00000198910	L1 cell adhesion molecule	L1CAM	-0.236	5.26e-06	-0.102	0.094
ZK1010.4	WBGene00014178	-	-1.49362520120724	0.00531628	-							
T05E11.3	WBGene00011480		-1.49314	0.00304733	ENSP00000299767	ENSG00000166598	tumor rejection antigen (gp96) 1	TRA1	-0.0347	0.709	-0.0623	0.501
Y37A1B.7	WBGene00012540	-	-1.49262520120724	0.001269617	-							
Y71H2B.10a	WBGene00000160	apb-1	-1.49191	0.00963479	ENSP00000350199	ENSG00000100280	adaptor-related protein complex 1, beta 1 subunit	AP1B1	0.0133	0.756	-0.064	0.301

C32D5.7	WBGene00016315	-	-1.48962520120724	0.001720154	-							
ZK180.5b	WBGene00022679	-	-1.48762520120724	0.00977749	-							
K01C8.3b	WBGene00006562	tdc-1	-1.48701	0.00541071	ENSP00000339928	ENSG00000132437	Dopa decarboxylase (aromatic L-amino acid decarboxylase)	DDC	-0.0553	0.152	-0.0549	0.277
Y22D7AL.16	WBGene00021254		-1.48462520120724	0.0009700651	-							
C46E1.1	WBGene00008115	-	-1.48462520120724	0.002574447	-							
K03H1.4	WBGene00010539	-	-1.48362520120724	0.004219066	-							
F01G12.2a	WBGene00006353	sur-7	-1.48362520120724	0.007808274	-							
F54B8.4	WBGene00010019	-	-1.48262520120724	0.000893048	-							
ZK154.3	WBGene00003171	mec-7	-1.48262520120724	0.001790284	-							
F41E7.1	WBGene00009617		-1.48087	0.00755379	ENSP00000296422	ENSG00000164037	Na+/H+ exchanger domain containing 1	NHEDC1				
C49G7.3	WBGene00016781	-	-1.48062520120724	0.001357627	-							
F53H4.5	WBGene00010010	-	-1.48062520120724	0.002216801	-							
M03E7.2	WBGene00019754	-	-1.47762520120724	0.001257172	-							
Y105E8A.5	WBGene00000241	bbs-1	-1.47762520120724	0.001552172	-							
C07D10.4	WBGene00003526	nas-7	-1.47662520120724	0.001480923	-							
F22D6.3a	WBGene00003815	nrs-1	-1.47597	0.00800776	ENSP00000256854	ENSG00000134440	asparaginyl-tRNA synthetase /// asparaginyl-tRNA synthetase	NARS	-0.12	0.0107	-0.0771	0.0481
C37H5.1	WBGene00003591	nex-4	-1.47462520120724	0.008703459	-							
F41E6.2	WBGene00001694	grd-5	-1.47162520120724	0.0007899062	-							
K06A4.7	WBGene00010597		-1.47162520120724	0.005688866	-							
T05B4.10	WBGene00020241	-	-1.46962520120724	0.004464422	-							
F36H5.8	WBGene00018106	-	-1.46962520120724	0.007098263	-							
F37A8.4	WBGene00003748	nlp-10	-1.46762520120724	0.00300476	-							
Y46B2A.1	WBGene00021576	-	-1.46662520120724	0.0008835172	-							
F52E4.5	WBGene00018702	-	-1.46462520120724	0.004665318	-							
F27C1.4	WBGene00017854	-	-1.46362520120724	0.005963218	-							
F31D4.8	WBGene00009293	-	-1.46362520120724	0.006055877	-							
F40F4.6	WBGene00018237	-	-1.46162520120724	0.0008679104	-							
C02E7.6	WBGene00015339	-	-1.46062520120724	0.0008390602	-							
Y57G11C.31	WBGene00013321	-	-1.46062520120724	0.0009431409	-							

D2063.3b	WBGene00017062	-	-1.46062520120724	0.00497475	-								
T19D7.2	WBGene00020570	-	-1.45962520120724	0.001678689	-								
C04G6.10	WBGene00015458	-	-1.45762520120724	0.0007008933	-								
C08G5.4	WBGene00004926	snt-6	-1.45662520120724	0.002768855	-								
B0222.3	WBGene00015055		-1.45635	0.00255843	ENSP00000272542	ENSG00000144136	solute carrier family 20 (phosphate transporter), member 1	SLC20A1	0.0644	0.401	-0.25	0.00233
B0348.4a	WBGene00001177	egl-8	-1.45512	0.00659609	ENSP00000334105	ENSG00000101333	phospholipase C, beta 4	PLCB4	0.0395	0.246	-0.203	0.00999
ZK1193.2	WBGene00022858	-	-1.45462520120724	0.001230807	-								
F56A11.3	WBGene00001165	efn-4	-1.45267	0.00357211	ENSP00000357392	ENSG00000169242	ephrin-A1	EFNA1	0.446	0.000337	0.18	0.0713
ZC155.5a	WBGene00022533	-	-1.45162520120724	0.008595552	-								
M04G12.1b	WBGene00010867	tag-260	-1.45144	0.00624327	ENSP00000355568	ENSG00000168264	interferon regulatory factor 2 binding protein 2	IRF2BP2	0.264	0.000409	-0.0873	0.324
F42G8.11	WBGene00004979	sph-1	-1.45144	0.00712932	ENSP00000263233	ENSG00000102003	synaptophysin	SYP	-0.44	2.04e-08	-0.247	0.0057
C48E7.1	WBGene00016749	-	-1.45062520120724	0.001441939	-								
F33H12.4	WBGene00005586	sri-74	-1.44862520120724	0.001269682	-								
T02E9.2	WBGene00001716	grl-7	-1.44862520120724	0.001391041	-								
C18D11.1	WBGene00007679	-	-1.44862520120724	0.003941517	-								
T01D1.3	WBGene00020148	-	-1.44862520120724	0.004459625	-								
T01B7.8	WBGene00011313	-	-1.44762520120724	0.0007899062	-								
F15B9.1	WBGene00001387	far-3	-1.44762520120724	0.002951596	-								
C08F11.13	WBGene00007460	-	-1.44662520120724	0.004220779	-								
F59B1.9	WBGene00000062	acr-23	-1.44662520120724	0.00744196	-								
C23H3.1	WBGene00001193	egl-26	-1.44462520120724	0.001109819	-								
F23B2.5a	WBGene00001444	flp-1	-1.44462520120724	0.001822802	-								
C42D4.3	WBGene00016596	-	-1.44362520120724	0.0009302254	-								
ZK54.3	WBGene00022648	-	-1.44162520120724	0.002883467	-								
C54D2.1	WBGene00016915	-	-1.43862520120724	0.001181518	-								
F09C3.2	WBGene00008610	-	-1.43862520120724	0.002568256	-								
F13B12.4	WBGene00008732	-	-1.43762520120724	0.002174939	-								
F36H1.11	WBGene00009496	-	-1.43762520120724	0.004988244	-								
F44E7.5a	WBGene00018427	-	-1.43462520120724	0.001674829	-								

K11D12.4	WBGene00019644	cpt-4	-1.43162520120724	0.0008801946	-							
F58E6.1a	WBGene00010251	-	-1.43162520120724	0.001218242	-							
F14D7.7	WBGene00008794	-	-1.43162520120724	0.001304383	-							
E04A4.2	WBGene00017117	fbxb-80	-1.43162520120724	0.004023996	-							
F46F3.1	WBGene00000449	ceh-27	-1.4306	0.00499451	ENSP00000 304331	ENSG000 00169840	GS homeobox 1	GSH1	-0.0902	0.0141	-0.0199	0.689
F48F7.2	WBGene00006372	syn-2	-1.42862520120724	0.009425957	-							
F09C3.1	WBGene00003980	pes-7	-1.42814	0.00738211	ENSP00000 268182	ENSG000 00140575	IQ motif containing GTPase activating protein 1	IQGAP1	0.354	2.16e-06	0.143	0.252
F26G1.2	WBGene00017838	-	-1.42762520120724	0.002912682	-							
C43C3.3	WBGene00001124	dyf-8	-1.42762520120724	0.006105483	-							
F41H10.5	WBGene00018318	-	-1.42162520120724	0.0009704376	-							
Y64G10A.6	WBGene00013415	-	-1.42162520120724	0.005364271	-							
R07B1.10	WBGene00002271	lec-8	-1.42062520120724	0.002095435	-							
ZK1320.4	WBGene00014254	cyp-13A10	-1.41956	0.00813044	ENSP00000 222982	ENSG000 00106258	cytochrome P450, family 3, subfamily A, polypeptide 5	CYP3A5	-0.019	0.47	0.0539	0.211
ZC328.1	WBGene00022591	-	-1.41862520120724	0.0010786	-							
W06B3.2b	WBGene00004859	sma-5	-1.41862520120724	0.00307325	-							
F35C11.4	WBGene00009403		-1.41588	0.00670756	ENSP00000 363984	ENSG000 00165152	chromosome 9 open reading frame 125 /// chromosome 9 open reading frame 125	C9orf125	-0.388	7.67e-05	-0.274	0.0241
F23B2.11	WBGene00003958	pcp-3	-1.41466	0.00875005	ENSP00000 366676	ENSG000 00112812	protease, serine, 16 (thymus)	PRSS16	0.0215	0.538	-0.0745	0.221
F35E8.1	WBGene00009417	-	-1.41462520120724	0.004339155	-							
F18E9.2	WBGene00003745	nlp-7	-1.41462520120724	0.008677599	-							
M02H5.6	WBGene00003688	nhr-98	-1.41362520120724	0.0009704376	-							
H19M22.2b	WBGene00002915	let-805	-1.41362520120724	0.003347877	-							
Y71H2AL.1	WBGene00022164	-	-1.41362520120724	0.003541487	-							
F14B4.1	WBGene00008779	-	-1.41362520120724	0.004048031	-							
T07C5.3	WBGene00011566	nhr-214	-1.41162520120724	0.001743451	-							
F47D12.1a	WBGene00001518	gar-2	-1.41162520120724	0.004582421	-							
C26B2.8	WBGene00016130	-	-1.41062520120724	0.001102175	-							

F54H5.4	WBGene00003480	mua-1	-1.41062520120724	0.003843775	-								
T27F2.4	WBGene00012101	-	-1.40762520120724	0.001257213	-								
Y48G10A.6	WBGene00013023	-	-1.40462520120724	0.0009740664	-								
T10B9.8	WBGene00011677	cyp-13A1	-1.40362	0.00726553	ENSP00000222982	ENSG00000106258	cytochrome P450, family 3, subfamily A, polypeptide 5	CYP3A5	-0.019	0.47	0.0539	0.211	
T25E12.4a	WBGene00012019	dkf-2	-1.40239	0.00524473	ENSP00000368356	ENSG00000115825	protein kinase D3	PRKD3	0.223	1.43e-05	0.0933	0.0893	
W04E12.9	WBGene00012254	-	-1.40062520120724	0.001138466	-								
F42E11.2c	WBGene00009632		-1.39871	0.00675292	ENSP00000269346	ENSG00000141540	tweety homolog 2 (Drosophila)	TTYH2	-0.0378	0.5	-0.246	0.000546	
F23H11.7	WBGene00017762	-	-1.39862520120724	0.001503824	-								
R05A10.2	WBGene00011019	-	-1.39862520120724	0.003027058	-								
T07C4.5	WBGene00011561	-	-1.39862520120724	0.00669748	-								
C30A5.6	WBGene00016240	-	-1.39762520120724	0.007098263	-								
F21F3.1	WBGene00017671	-	-1.39762520120724	0.008100861	-								
C34H3.2	WBGene00003846	odd-2	-1.39626	0.00285176	ENSP00000297565	ENSG00000164920	odd-skipped related 2 (Drosophila)	OSR2	0.00975	0.702	0.00359	0.924	
Y110A2AL.4b	WBGene00022441	-	-1.39562520120724	0.001678689	-								
T23E7.4	WBGene00003744	nlp-6	-1.39562520120724	0.003728648	-								
F40F8.8	WBGene00009579		-1.39504	0.00821526	ENSP00000265138	ENSG00000113369	arrestin domain containing 3	ARRDC3	0.653	9.31e-05	0.0788	0.636	
T02E1.8	WBGene00011380	-	-1.39262520120724	0.001021615	-								
C49G7.4	WBGene00016782	phat-3	-1.39262520120724	0.001358436	-								
F27E11.3a	WBGene00000478	cfz-2	-1.39258	0.00422876	ENSP00000363826	ENSG00000177283	frizzled homolog 8 (Drosophila) /// frizzled homolog 8 (Drosophila)	FZD8	0.0629	0.164	0.162	0.0256	
F45E10.2b	WBGene00009729	-	-1.39162520120724	0.003398825	-								
K07C11.10	WBGene00019482	-	-1.39162520120724	0.005070931	-								
K08F9.3	WBGene00010686	-	-1.39162520120724	0.007206327	-								
F20B6.4	WBGene00017628	-	-1.38862520120724	0.003920001	-								
C26F1.10	WBGene00001464	flp-21	-1.38762520120724	0.001173715	-								
C01G12.11	WBGene00007251	nspb-9	-1.38562520120724	0.005193423	-								
F53G12.7	WBGene00000622	col-45	-1.38462520120724	0.001037695	-								
R05C11.1	WBGene00019873	-	-1.38462520120724	0.001157252	-								

Y48C3A.5b	WBGene00012989	-	-1.38462520120724	0.006677159	-							
W01C9.5	WBGene00012178	-	-1.37762520120724	0.001480923	-							
F52F12.6	WBGene00009939	ztf-11	-1.37664	0.00513209	ENSP00000327465	ENSG00000196132	metal-regulatory transcription factor 1	MTF1	0.188	5.09e-07	0.0221	0.595
ZK265.7	WBGene00013959	-	-1.37562520120724	0.005430889	-							
F15B9.8	WBGene00008851	-	-1.37462520120724	0.001790244	-							
K02G10.4a	WBGene00001454	flp-11	-1.37462520120724	0.003099354	-							
Y82E9BL.12	WBGene00022328	-	-1.37462520120724	0.004947873	-							
C35C5.10	WBGene00007959		-1.37419	0.00772173	ENSP00000361143	ENSG00000157600	hypothetical protein FLJ22679	RP13-360B22.2	-0.00509	0.909	0.121	0.0195
Y38C1AA.9	WBGene00021400	-	-1.37262520120724	0.004470968	-							
K01C8.2	WBGene00010457	-	-1.37162520120724	0.001138466	-							
Y54G2A.14	WBGene00021879	clec-83	-1.36762520120724	0.001654431	-							
T05B4.11	WBGene00020242	phat-5	-1.36762520120724	0.002510214	-							
K11H12.4	WBGene00019660	-	-1.36562520120724	0.002079953	-							
F59C6.11	WBGene00010328	-	-1.36562520120724	0.003307628	-							
F14E5.5	WBGene00008803	-	-1.36462520120724	0.005791128	-							
Y47H9C.5	WBGene00001045	dnj-27	-1.36438	0.00534328	ENSP00000264065	ENSG00000077232	DnaJ (Hsp40) homolog, subfamily C, member 10	DNAJC10	0.0674	0.344	-0.198	0.0354
F42G9.9d	WBGene00004212	ptl-1	-1.36438	0.00368251	ENSP00000353375	ENSG00000047849	microtubule-associated protein 4	MAP4	0.0265	0.522	-0.000668	0.991
W07G4.6	WBGene00005652	srr-1	-1.35962520120724	0.001102175	-							
F45H11.1	WBGene00009743	sptf-1	-1.35962520120724	0.001334132	-							
C06A8.3	WBGene00015514	-	-1.35962520120724	0.00144849	-							
C34D1.5	WBGene00007932	zip-5	-1.35962520120724	0.003867536	-							
F54D5.15	WBGene00010057	-	-1.35962520120724	0.004868479	-							
R12E2.9	WBGene00002137	inx-15	-1.35562520120724	0.001245573	-							
C03F11.2	WBGene00015388	-	-1.35462520120724	0.002608564	-							
Y74C9A.2	WBGene00022276	nlp-40	-1.34862520120724	0.006214731	-							
K02B12.4	WBGene00010497	axl-1	-1.34762520120724	0.007017	-							
W06F12.1a	WBGene00003048	lit-1	-1.34721	0.00479569	ENSP00000262393	ENSG00000087095	Nemo like kinase	NLK	-0.113	0.0112	-0.0806	0.228
C09D1.2	WBGene00015631	-	-1.34162520120724	0.004099688	-							
F35C8.7a	WBGene00018037	chtl-1	-1.33985	0.00331389	ENSP00000336888	ENSG00000129353	CTL2 protein	CTL2	0.29	7.59e-07	0.063	0.471

R06A4.8	WBGene00011050		-1.33962520120724	0.005193423	-								
C05B5.6	WBGene00007323	fbxa-155	-1.33862520120724	0.001434035	-								
T28C12.6	WBGene00020893	-	-1.33862520120724	0.001503824	-								
C05B5.3	WBGene00004100	pqn-8	-1.33562520120724	0.003628649	-								
F01D5.8	WBGene00008498	-	-1.33562520120724	0.007462534	-								
T10B9.10	WBGene00000372	cyp-13A7	-1.33372	0.00983081	ENSP00000222982	ENSG00000106258	cytochrome P450, family 3, subfamily A, polypeptide 5	CYP3A5	-0.019	0.47	0.0539	0.211	
C42D8.5	WBGene00000039	acn-1	-1.33372	0.00678432	ENSP00000290866	ENSG00000159640	angiotensin I converting enzyme (peptidyl-dipeptidase A) 1	ACE	-0.209	3.48e-07	-0.0103	0.771	
C08B6.10	WBGene00007430	-	-1.33262520120724	0.00538921	-								
C12D5.7	WBGene00015709	cyp-33A1	-1.33127	0.00742504	ENSP00000360317	ENSG00000138115	cytochrome P450, family 2, subfamily C, polypeptide 8 /// cytochrome P450, family 2, subfamily C, polypeptide 8	CYP2C8	-0.151	6.66e-05	-0.0017	0.976	
W02D3.7	WBGene00002257	lbp-5	-1.33004	0.00410703	ENSP00000362817	ENSG00000121769	fatty acid binding protein 3, muscle and heart (mammary-derived growth inhibitor)	FABP3	-0.0737	0.0485	-0.229	0.00379	
Y50E8A.3	WBGene00003861	oig-3	-1.32962520120724	0.001565339	-								
F47G6.4	WBGene00004969	spe-15	-1.32759	0.00470043	ENSP00000358994	ENSG00000196586	myosin VI	MYO6	0.27	0.000396	0.204	0.00659	
F52A8.3a	WBGene00009916	-	-1.32662520120724	0.001438048	-								
C03B1.12	WBGene00003053	lmp-1	-1.32636	0.00334639	ENSP00000333298	ENSG00000185896	lysosomal-associated membrane protein 1	LAMP1	0.121	9.74e-06	0.0266	0.634	
F18E9.1	WBGene00017568	-	-1.32562520120724	0.006653498	-								
F28A12.2	WBGene00001721	grl-12	-1.32462520120724	0.001735694	-								
W07E6.3	WBGene00021075	-	-1.32462520120724	0.002125453	-								

C55A6.5	WBGene00008334	sdz-8	-1.32462520120724	0.004011026	-								
D2005.6	WBGene00008401	-	-1.32262520120724	0.005688866	-								
Y82E9BL.16	WBGene00022332	fbxa-20	-1.31962520120724	0.003598695	-								
Y95B8A.1	WBGene00003548	nas-30	-1.31962520120724	0.004009942	-								
D1069.2	WBGene00000778	cpn-2	-1.31901	0.0032969	ENSP00000273368	ENSG00000144834	transgelin 3	TAGLN3	-0.869	2.93e-09	-0.129	0.0111	
F08A8.1b	WBGene00008564		-1.31778	0.00346971	ENSP00000293217	ENSG00000161533	Acyl-Coenzyme A oxidase 1, palmitoyl	ACOX1	0.113	0.0307	0.162	0.00506	
F09E8.8	WBGene00008626	-	-1.31662520120724	0.001916614	-								
M6.1b	WBGene00002056	ifc-2	-1.31662520120724	0.005331616	-								
C26D10.6	WBGene00007746	-	-1.31562520120724	0.003781848	-								
F54F11.1	WBGene00010069	-	-1.31362520120724	0.001431072	-								
T24F1.7	WBGene00011999	-	-1.31362520120724	0.00157252	-								
R05C11.3	WBGene00019875	-	-1.31262520120724	0.00410237	-								
C43C3.1	WBGene00002067	ifp-1	-1.30962520120724	0.002884112	-								
ZK512.9	WBGene00001720	grl-11	-1.30562520120724	0.001438048	-								
R08A2.7	WBGene00011137	-	-1.30562520120724	0.003023224	-								
F38B6.1	WBGene00018171	-	-1.30362520120724	0.001847145	-								
C25H3.12	WBGene00016121	-	-1.30262520120724	0.004393545	-								
C55A6.6	WBGene00008335	-	-1.30262520120724	0.005223131	-								
C25G6.4	WBGene00016109	-	-1.30262520120724	0.005253037	-								
F09B12.1b	WBGene00008605	mlt-9	-1.29362520120724	0.001484514	-								
K09E4.4	WBGene00010722		-1.2908	0.00670756	ENSP00000225927	ENSG00000108784	N-acetylglucosaminidase, alpha- (Sanfilippo disease IIIB)	NAGLU	-0.0151	0.598	-0.0515	0.22	
F54H5.5	WBGene00018841	-	-1.28962520120724	0.002012824	-								
K06A4.2	WBGene00010592	-	-1.28962520120724	0.0059741	-								
C31H5.5	WBGene00007856	-	-1.28962520120724	0.006653498	-								
K09D9.1	WBGene00019564	-	-1.28862520120724	0.008101524	-								
K03E5.1	WBGene00019360		-1.28712	0.00741318	ENSP00000306726	ENSG00000171208	neuropilin (NRP) and tolloid (TLL)-like 2	NETO2	-1.09	4.94e-11	-0.596	0.000124	
ZK945.1	WBGene00014164	lact-2	-1.28662520120724	0.001460394	-								
T16G1.5	WBGene00011799	-	-1.28562520120724	0.002732818	-								
T19C4.7	WBGene00003771	nlp-33	-1.28562520120724	0.003633165	-								

C01G12.7	WBGene00007247	-	-1.28362520120724	0.001521507	-							
Y66A7A.6	WBGene00001633	gly-8	-1.28362520120724	0.001593394	-							
Y27F2A.5	WBGene00021297	-	-1.28362520120724	0.003707096	-							
T10B9.9	WBGene00011678	-	-1.28362520120724	0.004375276	-							
M79.4	WBGene00001462	flp-19	-1.28262520120724	0.003822098	-							
C34B7.3	WBGene00007913	cyp-36A1	-1.28262520120724	0.007114898	-							
R11D1.11	WBGene00000984	dhs-21	-1.27977	0.00341339	ENSP00000303356	ENSG00000169738	dicarbonyl/L-xylulose reductase	DCXR	0.177	0.00303	0.279	0.00223
F41E6.1	WBGene00018284	-	-1.27962520120724	0.004251935	-							
H19M22.2d	WBGene00002915	let-805	-1.27862520120724	0.00157252	-							
C05C9.2	WBGene00007326	-	-1.27862520120724	0.00671665	-							
R09A8.4	WBGene00000755	col-182	-1.27662520120724	0.006214731	-							
F20G2.6	WBGene00008989	-	-1.27562520120724	0.00433951	-							
K11C4.4	WBGene00003844	odc-1	-1.27486	0.00368591	ENSP00000234111	ENSG00000115758	Ornithine decarboxylase	ODC1	0.403	0.00000368	-0.123	0.149
C07E3.3	WBGene00007414	-	-1.27362520120724	0.001631984	-							
K08C9.4	WBGene00000641	col-65	-1.27362520120724	0.002733235	-							
F29G6.3a	WBGene00009259	-	-1.27362520120724	0.003501954	-							
Y47H10A.5	WBGene00012961	-	-1.27262520120724	0.002888572	-							
F36H12.1	WBGene00018117	-	-1.27262520120724	0.004593803	-							
T28D6.4	WBGene00012124		-1.27118	0.00433315	ENSP00000281131	ENSG00000151458	KIAA1223 protein	KIAA1223	-0.157	0.00141	-0.0211	0.769
R173.3	WBGene00020125	-	-1.27062520120724	0.001612306	-							
ZK757.1	WBGene00014073	-	-1.27062520120724	0.00665834	-							
C29G2.3	WBGene00016231	-	-1.26962520120724	0.003517128	-							
D1044.7	WBGene00017032	-	-1.26662520120724	0.001583879	-							
C52G5.2	WBGene00008260	-	-1.26262520120724	0.001627835	-							
T19B10.1	WBGene00011830	cyp-29A2	-1.26262520120724	0.00595856	-							
F52G3.5	WBGene00018713	-	-1.26062520120724	0.002350222	-							
T28F3.4b	WBGene00012130	-	-1.26062520120724	0.009941195	-							
T10H9.6	WBGene00006224	str-180	-1.25762520120724	0.009043437	-							
C30F2.2	WBGene00007816	-	-1.25662520120724	0.001890268	-							
T03G11.3	WBGene00020196		-1.25524	0.00399183	ENSP00000237275	ENSG00000118491	family with sequence similarity 164, member B	FAM164B				
R07E3.3	WBGene00011104	cut-5	-1.25262520120724	0.003707096	-							

Y57A10A.26	WBGene00013266	-	-1.25062520120724	0.001841036	-							
ZK673.7	WBGene00006583	tnc-2	-1.25062520120724	0.002424534	-							
T19H12.6	WBGene00020590	-	-1.24962520120724	0.004202926	-							
C34D4.9	WBGene00003527	nas-8	-1.24762520120724	0.001520771	-							
T04F8.4	WBGene00011443	-	-1.24762520120724	0.007460861	-							
Y73B3A.15	WBGene00022217	fbxa-221	-1.24662520120724	0.008492983	-							
Y50E8A.5	WBGene00013048	-	-1.24562520120724	0.002155567	-							
EEED8.2	WBGene00017133	-	-1.24362520120724	0.001732354	-							
C12D12.5	WBGene00015716	-	-1.24062520120724	0.00452899	-							
R09A1.5	WBGene00019975	-	-1.23962520120724	0.002429988	-							
Y57G11B.6	WBGene00013298	-	-1.23962520120724	0.007159413	-							
C05D10.4b	WBGene00015480	-	-1.23762520120724	0.001831184	-							
T27F6.8	WBGene00012107	-	-1.23762520120724	0.004657856	-							
R07A4.1	WBGene00001202	egl-36	-1.23439	0.00479086	ENSP00000265969	ENSG00000129159	potassium voltage-gated channel, Shaw-related subfamily, member 1	KCNC1	-0.258	0.000159	-0.0804	0.319
ZK218.5	WBGene00013939	-	-1.23262520120724	0.003865367	-							
F38B2.2	WBGene00009529	-	-1.23262520120724	0.008706986	-							
M01A8.1	WBGene00010795	-	-1.22662520120724	0.003289076	-							
Y45G5AM.6	WBGene00021558	-	-1.22662520120724	0.003305886	-							
F35D2.3	WBGene00018041	-	-1.22662520120724	0.004412563	-							
R09H10.3	WBGene00011181		-1.22581	0.00482513	ENSP00000237014	ENSG00000118271	transthyretin (prealbumin, amyloidosis type I)	TTR	0.217	0.0645	-0.0737	0.0763
F30B5.1	WBGene00001074	dpy-13	-1.22362520120724	0.008299768	-							
B0478.1a	WBGene00002178	jnk-1	-1.21845	0.00489958	ENSP00000352157	ENSG00000109339	mitogen-activated protein kinase 10	MAPK10	-0.0242	0.552	-0.18	0.0676
B0361.5	WBGene00015159	psd-1	-1.21845	0.00671402	ENSP00000338695	ENSG00000100141	phosphatidylserine decarboxylase	PISD	-0.285	3.28e-07	-0.0219	0.697
Y105C5B.5	WBGene00013647	-	-1.21762520120724	0.006397387	-							
ZC204.2	WBGene00022554	-	-1.21762520120724	0.00778583	-							
ZC334.7	WBGene00013865	-	-1.21462520120724	0.005082815	-							
T14B4.6	WBGene00001064	dpy-2	-1.21062520120724	0.002748523	-							
C25G6.2	WBGene00006635	tsp-9	-1.21062520120724	0.008183025	-							
C08B6.4	WBGene00007425	-	-1.20762520120724	0.002390992	-							

C49G7.2	WBGene00005385	srh-169	-1.20762520120724	0.009835524	-								
R03H10.2	WBGene00019855	-	-1.20662520120724	0.003289076	-								
F58H1.2	WBGene00010285	-	-1.20662520120724	0.006332147	-								
W06D4.1	WBGene00001843	hgo-1	-1.20496	0.0040076	ENSP00000283871	ENSG00000113924	homogentisate 1,2-dioxygenase	HGD					
Y61A9LA.1	WBGene00022014	-	-1.20462520120724	0.002390992	-								
ZC13.3	WBGene00022504	-	-1.20462520120724	0.003707096	-								
K10C2.5	WBGene00001715	grl-6	-1.20362520120724	0.001959461	-								
C18E3.6	WBGene00015975	-	-1.20362520120724	0.004627741	-								
T19C3.3	WBGene00020561	-	-1.20362520120724	0.00977749	-								
F15E6.5	WBGene00017486	-	-1.20062520120724	0.003947947	-								
F38B6.7	WBGene00018176	-	-1.20062520120724	0.004182291	-								
F38H4.3	WBGene00009547	-	-1.19762520120724	0.002593202	-								
K10H10.3a	WBGene00000972	dhs-8	-1.19761	0.00471554	ENSP00000333406	ENSG00000186153	WW domain containing oxidoreductase	WWOX	0.107	0.0649	-0.107	0.139	
Y15E3A.1	WBGene00003681	nhr-91	-1.19761	0.00406601	ENSP00000362686	ENSG00000148200	nuclear receptor subfamily 6, group A, member 1	NR6A1	-0.0336	0.412	0.0504	0.216	
F34H10.5	WBGene00009382	-	-1.19462520120724	0.006372875	-								
F53C11.8	WBGene00009977	swan-1	-1.19462520120724	0.00980338	-								
K06B4.10	WBGene00010604	nhr-199	-1.19362520120724	0.006088313	-								
F26A3.6	WBGene00009144	del-3	-1.19362520120724	0.007098263	-								
F32B5.2	WBGene00017976	-	-1.19162520120724	0.002064447	-								
T27A8.1	WBGene00012073		-1.19162520120724	0.003077935	-								
T25C12.3	WBGene00012018	-	-1.18962520120724	0.002236159	-								
F10F2.3	WBGene00008655	-	-1.18962520120724	0.002731181	-								
T25C8.2	WBGene00000067	act-5	-1.18362520120724	0.003398825	-								
Y43F4A.1a	WBGene00012796		-1.18289	0.00559801	ENSP00000328829	ENSG00000185621	Leishmanolysin-like (metallopeptidase M8 family)	LMLN	-0.00772	0.845	0.0115	0.835	
C16D9.5	WBGene00015860	-	-1.18162520120724	0.002638225	-								
Y39B6A.6	WBGene00006639	tsp-13	-1.17862520120724	0.002079232	-								
R05A10.4	WBGene00011021	-	-1.17862520120724	0.006157172	-								
T26H10.1	WBGene00000955	des-2	-1.17862520120724	0.006569753	-								

C01B12.4	WBGene00015287		-1.17799	0.00707051	ENSP00000296327	ENSG00000163959	organic solute transporter alpha	OST alpha				
K08D12.4	WBGene00019538	-	-1.17762520120724	0.002465431	-							
H22D07.1	WBGene00019224	gnrr-5	-1.17462520120724	0.007705702	-							
F12F3.1	WBGene00001374	exp-2	-1.17431	0.00435722	ENSP00000360806	ENSG00000158445	potassium voltage-gated channel, Shab-related subfamily, member 1	KCNB1	-0.614	9.08e-08	-0.14	0.137
C05E7.1	WBGene00007343	-	-1.17162520120724	0.007098263	-							
T05D4.3	WBGene00011476	-	-1.17062520120724	0.002125453	-							
T06G6.12	WBGene00011551	-	-1.17062520120724	0.002235157	-							
F58F6.1	WBGene00000678	col-104	-1.17062520120724	0.00726526	-							
B0213.5	WBGene00003768	nlp-30	-1.17062520120724	0.008101524	-							
F42G8.7	WBGene00018358	-	-1.16862520120724	0.002638225	-							
F36H1.4c	WBGene00002992	lin-3	-1.16862520120724	0.005009502	-							
C27H5.1	WBGene00003966	pdl-1	-1.16818	0.00641502	ENSP00000287600	ENSG00000156973	phosphodiesterase 6D, cGMP-specific, rod, delta	PDE6D	-0.12	0.00125	-0.184	0.00107
E04F6.3	WBGene00017123	maoc-1	-1.16762520120724	0.002129956	-							
ZC455.1	WBGene00013902	-	-1.16762520120724	0.003193769	-							
Y113G7A.15	WBGene00013752	-	-1.16762520120724	0.00538921	-							
T27C4.2	WBGene00020855	-	-1.16762520120724	0.006496216	-							
R13.1	-	-	-1.16762520120724	0.006809472	-							
T01D3.3a	WBGene00011328	-	-1.16762520120724	0.006900026	-							
C31B8.12	WBGene00016285	-	-1.16762520120724	0.006918602	-							
K08E7.9	WBGene00003995	pgp-1	-1.16572	0.00440086	ENSP00000265724	ENSG00000085563	ATP-binding cassette, sub-family B (MDR/TAP), member 1	ABCB1	0.0752	0.0186	-0.0626	0.0926
BE10.2	WBGene00007210		-1.16572	0.00500637	ENSP00000341662	ENSG00000187546						
F02G3.1b	WBGene00017184	ncam-1	-1.16462520120724	0.002891465	-							
C10B5.3	WBGene00015671	-	-1.16462520120724	0.00494805	-							
C33D3.1	WBGene00001250	elt-2	-1.1645	0.00617262	ENSP00000359576	ENSG00000130700	GATA binding protein 5	GATA5	-0.0501	0.0887	-0.0326	0.446

C25A1.5	WBGene00007707		-1.1645	0.00600731	ENSP00000219368	ENSG00000103089	fatty acid 2-hydroxylase	FA2H	0.0249	0.798	-0.403	0.00614
F41A4.1	WBGene00018256	-	-1.16362520120724	0.005349995	-							
W03D8.6	WBGene00002174	itx-1	-1.16162520120724	0.004042073	-							
C01G6.3	WBGene00007225	-	-1.16062520120724	0.002196161	-							
ZC410.5b	WBGene00013882	-	-1.16062520120724	0.004558601	-							
F28H7.7	WBGene00009240	-	-1.15862520120724	0.009043437	-							
Y55B1AL.1	WBGene00021903	-	-1.15762520120724	0.002194229	-							
K05G3.2	WBGene00019423	-	-1.15562520120724	0.00611682	-							
F12D9.1b	WBGene00017400	-	-1.15562520120724	0.008595797	-							
T23F11.6	WBGene00011957	-	-1.15462520120724	0.002390992	-							
M110.9	WBGene00010917	-	-1.15362520120724	0.002311723	-							
W02D9.10	WBGene00012216	-	-1.15362520120724	0.003728648	-							
K02A2.3	WBGene00019289	kcc-2	-1.15362520120724	0.00810519	-							
F56F3.6	WBGene00002100	ins-17	-1.15162520120724	0.00382214	-							
C30B5.7	WBGene00016248	-	-1.15162520120724	0.004470968	-							
T07D10.1	WBGene00011581	-	-1.15062520120724	0.007220558	-							
F38C2.1	WBGene00009535	-	-1.15062520120724	0.008706986	-							
C49A1.4	WBGene00001377	eya-1	-1.14978	0.00448886	ENSP00000362978	ENSG00000158161	Eyes absent homolog 3 (Drosophila)	EYA3	-0.0155	0.527	0.0805	0.0392
C36H8.3	WBGene00001452	flp-9	-1.14762520120724	0.003527011	-							
W01C8.5	WBGene00020921	-	-1.14562520120724	0.002373262	-							
Y44A6D.2	WBGene00012853	-	-1.14562520120724	0.003343343	-							
C50F4.1	WBGene00008229	-	-1.14562520120724	0.004871252	-							
F48D6.3	WBGene00001957	hlh-13	-1.1412	0.00489958	ENSP00000345599	ENSG00000188686	Basic helix-loop-helix transcription factor scleraxis	SCXA				
K07C11.4	WBGene00019477	-	-1.14062520120724	0.002390992	-							
F54B8.3	WBGene00010018	fbxa-69	-1.14062520120724	0.00523186	-							
F48E3.8a	WBGene00018607	-	-1.14062520120724	0.009013902	-							
F23H12.3	WBGene00009093	-	-1.14062520120724	0.009088159	-							
R13A5.10	WBGene00020052	-	-1.13862520120724	0.002636893	-							
ZK262.2	WBGene00013949	-	-1.13862520120724	0.003275385	-							
Y64H9A.2	WBGene00022024	-	-1.13862520120724	0.007134214	-							
T01C8.2	WBGene00020143	-	-1.13762520120724	0.00243729	-							
F58B3.9	WBGene00010234	-	-1.13462520120724	0.0009783293	-							
R08C7.13	WBGene00019956	fbxb-75	-1.13462520120724	0.002854653	-							
Y65B4BR.6a	WBGene00001725	grl-16	-1.13262520120724	0.00272499	-							

C14A4.9	WBGene00007560	-	-1.13162520120724	0.002917621	-							
Y1H11.2	WBGene00001783	gst-35	-1.13162520120724	0.004226364	-							
C36F7.4a	WBGene00004372	rig-5	-1.13062520120724	0.0067358	-							
K12B6.4	WBGene00019669	-	-1.12862520120724	0.003883056	-							
Y32G9B.1	WBGene00021309	-	-1.12762520120724	0.005226282	-							
R01B10.3	WBGene00019805	-	-1.12762520120724	0.008758518	-							
F54E2.2	WBGene00018824	-	-1.12562520120724	0.002350222	-							
M04D8.6	WBGene00010864	xbx-3	-1.12562520120724	0.0045792	-							
T25F10.5	WBGene00000244	bbs-8	-1.12403	0.0075342	ENSP00000337653	ENSG00000165533	tetratricopeptide repeat domain 8	TTC8	0.0215	0.81	-0.0304	0.727
C05D12.1	WBGene00007339	-	-1.12262520120724	0.002390992	-							
B0302.5	WBGene00015122	-	-1.12262520120724	0.003338725	-							
C06B3.3	WBGene00007362	cyp-35C1	-1.12162520120724	0.003252696	-							
T04B8.5a	WBGene00020207		-1.12158	0.00489958	ENSP00000275730	ENSG00000146828	solute carrier family 12 (potassium/chloride transporters), member 9	SLC12A9	-0.0128	0.764	-0.0914	0.106
C10C5.3	WBGene00007507		-1.12035	0.00590152	ENSP00000232907	ENSG00000114786	aminoacylase 1	ACY1	0.103	0.0076	-0.0421	0.513
D1009.4	WBGene00003752	nlp-14	-1.11862520120724	0.009709793	-							
B0478.1b	WBGene00002178	jnk-1	-1.1179	0.00550331	ENSP00000352157	ENSG00000109339	mitogen-activated protein kinase 10	MAPK10	-0.0242	0.552	-0.18	0.0676
ZK6.10	WBGene00022644	dod-19	-1.11762520120724	0.002837449	-							
T09B9.3	WBGene00011644		-1.11762520120724	0.005595326	-							
F22E12.2	WBGene00009059	chw-1	-1.11762520120724	0.006487793	-							
C24A3.2a	WBGene00016033	-	-1.11462520120724	0.003822098	-							
C30F12.6	WBGene00016265		-1.11299	0.00968323	ENSP00000309818	ENSG00000174417	thyrotropin-releasing hormone receptor	TRHR	0.0478	0.285	0.0417	0.36
C26D10.5a	WBGene00001159	eff-1	-1.11062520120724	0.004375386	-							
R07E5.17	WBGene00011121	-	-1.10862520120724	0.003885035	-							
C25B8.4b	WBGene00016088	-	-1.10862520120724	0.004377448	-							
Y57A10A.11	WBGene00004394	rol-1	-1.10862520120724	0.005091369	-							
R07B7.13	WBGene00011097	nhr-206	-1.10862520120724	0.00531628	-							

B0024.4	WBGene00007097	-	-1.10562520120724	0.008703459	-								
ZK377.1	WBGene00006952	wrt-6	-1.10462520120724	0.007355692	-								
W06F12.3	WBGene00012307	-	-1.10262520120724	0.003037723	-								
C38C3.5a	WBGene00003591	unc-60	-1.10162520120724	0.009600638	-								
C34F11.9c	WBGene00001101	dsh-1	-1.10073	0.00526579	ENSP00000316054	ENSG00000161202	dishevelled, dsh homolog 3 (Drosophila)	DVL3	0.0161	0.634	-0.0264	0.614	
K05C4.11	WBGene00010587	-	-1.09962520120724	0.009096263	-								
R08E3.1a	WBGene00019957	-	-1.09962520120724	0.009325613	-								
F25B3.2	WBGene00009099	-	-1.09862520120724	0.005886656	-								
H10E21.4	WBGene00019184	-	-1.09762520120724	0.005259417	-								
T24B8.6	WBGene00001950	hlh-3	-1.09705	0.00612713	ENSP00000266744	ENSG00000139352	achaete-scute complex-like 1 (Drosophila)	ASCL1	0.178	0.00468	0.0907	0.229	
C45B11.3	WBGene00000981	dhs-18	-1.09583	0.00763328	ENSP00000262542	ENSG00000119471	hydroxysteroid dehydrogenase like 2	HSDL2	0.232	0.0261	0.199	0.0477	
D1037.3	WBGene00001501	ftn-2	-1.09583	0.009719	ENSP00000273550	ENSG00000167996	Ferritin, heavy polypeptide 1	FTH1	0.0393	0.21	-0.0244	0.607	
Y38H6C.20	WBGene00012632	-	-1.09562520120724	0.004136799	-								
C25D7.15	WBGene00007722	-	-1.09462520120724	0.003889607	-								
C05E11.3	WBGene00015494	-	-1.09162520120724	0.002868523	-								
F36H5.2c	WBGene00018101	math-27	-1.09162520120724	0.009908386	-								
C16C8.2	WBGene00015841	-	-1.08862520120724	0.002894117	-								
C18D1.3	WBGene00001447	flp-4	-1.08862520120724	0.003255848	-								
F09E5.14	WBGene00017291	-	-1.08862520120724	0.005091369	-								
C30G12.1	WBGene00016273	-	-1.08862520120724	0.008008335	-								
K07G5.4	WBGene00010643	-	-1.08862520120724	0.008415044	-								
F08B12.4	WBGene00008572	-	-1.08762520120724	0.002761928	-								
F53F4.6	WBGene00004328	rdy-2	-1.08762520120724	0.004187241	-								
F22F4.2	WBGene00002125	inx-3	-1.08762520120724	0.004692993	-								
B0395.2	WBGene00007174		-1.08724	0.00786187	ENSP00000356591	ENSG00000057252	Sterol O-acyltransferase (acyl-Coenzyme A: cholesterol acyltransferase) 1	SOAT1	0.0104	0.63	-0.00864	0.802	
Y43F8C.4	WBGene00012826		-1.08602	0.00970824	ENSP00000374221	ENSG00000188878							

ZK836.3	WBGene00014099	-	-1.08562520120724	0.00744196	-							
F16H6.10	WBGene00008900	-	-1.08462520120724	0.00306765	-							
C02F5.11	WBGene00006628	tsp-2	-1.08262520120724	0.008431438	-							
K02D7.1	WBGene00019298		-1.07988	0.00552664	ENSP00000354532	ENSG00000198805	nucleoside phosphorylase	NP	0.148	0.0265	0.263	0.0266
F53H8.2	WBGene00000195	arr-1	-1.07988	0.00738722	ENSP00000316694	ENSG00000137486	arrestin, beta 1	ARRB1	0.44	3.76e-11	0.0911	0.114
F37B1.2	WBGene00001760	gst-12	-1.07962520120724	0.002673894	-							
ZK270.1	WBGene00004237	ptr-23	-1.07962520120724	0.009749771	-							
D2092.8	WBGene00017068	-	-1.07862520120724	0.004002	-							
F53B1.3	WBGene00018736	-	-1.07862520120724	0.005611082	-							
D2089.4b	WBGene00004207	ptb-1	-1.07743	0.00799411	ENSP00000349428	ENSG00000011304	Polypyrimidine tract binding protein 1	PTBP1	0.045	0.265	0.0129	0.804
Y57G11C.49	WBGene00013338	lgc-8	-1.07462520120724	0.00875914	-							
F22F4.1	WBGene00017716	-	-1.07462520120724	0.009872366	-							
B0019.1	WBGene00000138	amx-2	-1.07262520120724	0.003307628	-							
C18A3.8	WBGene00001958	hlh-14	-1.07262520120724	0.00410237	-							
K10H10.6	WBGene00010762		-1.0713	0.00703918	ENSP00000333406	ENSG00000186153	WW domain containing oxidoreductase	WWOX	0.107	0.0649	-0.107	0.139
Y41D4B.16	WBGene00021518	-	-1.06962520120724	0.003027058	-							
F35E12.9	WBGene00009433	-	-1.06962520120724	0.006157172	-							
F52A8.4	WBGene00009917	-	-1.06862520120724	0.009503425	-							
C14C10.7	WBGene00007590	-	-1.06662520120724	0.002897135	-							
Y40C7B.4	WBGene00021500	-	-1.06262520120724	0.003247051	-							
W05E10.2	WBGene00012281		-1.06162520120724	0.00903513	-							
F56C9.7	WBGene00018951	-	-1.05862520120724	0.002978518	-							
W04G3.2	WBGene00012256	-	-1.05662520120724	0.002697598	-							
C37E2.5	WBGene00000458	ceh-37	-1.05562520120724	0.003529543	-							
C16D2.1	WBGene00007633	-	-1.05562520120724	0.007121811	-							
F38B6.5a	WBGene00000745	col-172	-1.05462520120724	0.00307325	-							
T04H1.6	WBGene00003075	lrx-1	-1.05162520120724	0.002832142	-							
W09G12.9	WBGene00021123	-	-1.05162520120724	0.006251398	-							
C08H9.15	WBGene00007474	-	-1.04662520120724	0.003704516	-							
H16D19.4	WBGene00010402	-	-1.04662520120724	0.00407742	-							
C26D10.5b	WBGene00001159	eff-1	-1.04662520120724	0.008910973	-							
F47G4.4	WBGene00009825	-	-1.04562520120724	0.003538719	-							
ZK84.1	WBGene00022649	-	-1.04562520120724	0.005055081	-							

F55G11.6	WBGene00010126	-	-1.04362520120724	0.003189747	-							
C35D10.12	WBGene00016448	-	-1.04262520120724	0.00528428	-							
T20F5.4	WBGene00020626	-	-1.04162520120724	0.003539389	-							
B0563.6b	WBGene00015263	-	-1.03662520120724	0.0082323	-							
C09G1.5	WBGene00007487	-	-1.03562520120724	0.008703459	-							
Y119C1B.6	WBGene00022472	-	-1.03362520120724	0.005799494	-							
C08F1.10	WBGene00015613	-	-1.03262520120724	0.004679956	-							
Y71D11A.5	WBGene00022106	-	-1.03262520120724	0.004742812	-							
C50B8.5	WBGene00008227	-	-1.03162520120724	0.003973433	-							
D1009.5	WBGene00017014	dylt-2	-1.03162520120724	0.004048312	-							
F13A7.12	WBGene00008727	-	-1.02962520120724	0.003391735	-							
R06C1.6	WBGene00011060	-	-1.02962520120724	0.003732448	-							
R11G11.2b	WBGene00003648	nhr-58	-1.02862520120724	0.003955698	-							
F33E2.3	WBGene00009359	-	-1.02662520120724	0.003958363	-							
F36H2.3b	WBGene00009500	-	-1.02562520120724	0.006192842	-							
F55A12.4c	WBGene00000966	dhs-2	-1.02348	0.00835146	ENSP00000318631	ENSG00000025423	hydroxysteroid (17-beta) dehydrogenase 6	HSD17B6	-0.0382	0.633	0.305	0.0044
T01B10.2	WBGene00001703	grd-14	-1.02262520120724	0.005948064	-							
F56B6.5	WBGene00006864	uvt-6	-1.02262520120724	0.006112049	-							
C25B8.3a	WBGene00000786	cpr-6	-1.02062520120724	0.003668366	-							
T13G4.6	WBGene00020493	-	-1.02062520120724	0.006298739	-							
C30H6.2	WBGene00006487	tag-141	-1.0198	0.0080208	ENSP00000269187	ENSG00000141424	solute carrier family 39 (zinc transporter), member 6	SLC39A6	-0.0982	0.396	-0.552	0.000862
T19B10.9	WBGene00011836	-	-1.01962520120724	0.008833807	-							
ZC482.5			-1.01962520120724	0.01005339	-							
F40H3.4	WBGene00001440	fkh-8	-1.01862520120724	0.003421816	-							
C24H10.5	WBGene00006861	uvt-2	-1.01662520120724	0.003457384	-							
T27B7.2	WBGene00020849	nhr-225	-1.01362520120724	0.004298294	-							
Y38H6C.1	WBGene00012615	-	-1.01362520120724	0.00721076	-							
Y57A10C.6	WBGene00013284		-1.01121	0.00775283	ENSP00000360569	ENSG00000116171	Sterol carrier protein 2	SCP2	0.117	0.0442	0.34	0.00002
Y43C5A.2	WBGene00012782	-	-1.01062520120724	0.00744196	-							
T20D3.2	WBGene00011856	-	-1.00962520120724	0.004657633	-							
F07C3.9	WBGene00017197	-	-1.00562520120724	0.00676735	-							
Y71H2AM.13	WBGene00022178	-	-1.00262520120724	0.003483707	-							
Y51H4A.9	WBGene00000710	col-137	-1.00262520120724	0.003542738	-							

F59B1.8	WBGene00019100	-	-1.00062520120724	0.003612786	-							
T05A7.7	WBGene00020224	-	-0.999625201207244	0.003920001	-							
C31H2.3	WBGene00016293	-	-0.999625201207244	0.006918602	-							
T23H2.4	WBGene00020743	-	-0.997625201207244	0.00483409	-							
W05H9.1	WBGene00021048	-	-0.993625201207244	0.003628649	-							
Y105E8A.12	WBGene00013672	catp-1	-0.993625201207244	0.003889607	-							
M28.8	WBGene00010899	-	-0.993625201207244	0.004331953	-							
C02A12.1	WBGene00001781	gst-33	-0.992625201207244	0.003704516	-							
C06H5.1	WBGene00007392	fbxa-156	-0.992625201207244	0.004340453	-							
F32D8.1	WBGene00009326	-	-0.992625201207244	0.0059741	-							
F46G10.1	WBGene00009796	-	-0.990625201207244	0.004209526	-							
F27E5.1	WBGene00009192	-	-0.987625201207244	0.003786734	-							
ZK938.2	WBGene00014159	-	-0.987625201207244	0.00607998	-							
B0213.15	WBGene00015044	cyp-34A9	-0.987625201207244	0.008258073	-							
ZK971.1	WBGene00014175	-	-0.986625201207244	0.009234635	-							
F15E6.2	WBGene00017483	lgc-22	-0.985625201207244	0.003728648	-							
Y50D7A.7	WBGene00000081	ads-1	-0.983009	0.00744984	ENSP00000264167	ENSG00000018510	Alkylglycerone phosphate synthase	AGPS	0.0311	0.561	-0.281	0.00807
C35B1.7	WBGene00016436	-	-0.980625201207244	0.003947947	-							
K03B8.6	WBGene00010524	-	-0.980625201207244	0.004671203	-							
F01D4.6b	WBGene00003167	mec-3	-0.980625201207244	0.00614338	-							
C25H3.5	WBGene00016114	flp-27	-0.977625201207243	0.008738143	-							
Y34B4A.6	WBGene00021322	-	-0.973625201207243	0.003569868	-							
C06A6.5	WBGene00015510	-	-0.973625201207243	0.004137496	-							
F58A3.5	WBGene00010225	-	-0.973625201207243	0.005442136	-							
F58E6.10	WBGene00006778	unc-42	-0.972625201207243	0.003973433	-							
C36F7.4b	WBGene00004372	rig-5	-0.972625201207243	0.006653039	-							
ZK370.6	WBGene00022720	-	-0.970625201207243	0.006793188	-							
F08B4.4	WBGene00017236	-	-0.967625201207243	0.004042073	-							
ZK520.2	WBGene00004796	sid-2	-0.967625201207243	0.004234281	-							
F31E3.2a	WBGene00017950	-	-0.967625201207243	0.006144164	-							
C17F4.7	WBGene00015913	-	-0.966625201207243	0.003786734	-							
T24C4.3	WBGene00020759	-	-0.966625201207243	0.007886972	-							
ZK1290.12	WBGene00006947	wrt-1	-0.960936	0.00796426	ENSP00000266991	ENSG00000139549	desert hedgehog homolog (Drosophila)	DHH				
C15C8.1	WBGene00007604	-	-0.959625201207243	0.005009502	-							
R07C12.2	WBGene00019932	-	-0.957625201207243	0.006841351	-							

Y46G5A.8	WBGene00012900	-	-0.956625201207243	0.004815084	-								
T27E9.9	WBGene00012099	acc-4	-0.954625201207243	0.008469742	-								
Y42H9AR.5	WBGene00021540		-0.954625201207243	0.009756621	-								
C08F11.8	WBGene00007455	ugt-22	-0.951126	0.00885429	ENSP00000 282507	ENSG000 00168671	UDP glycosyltransfer ase 3 family, polypeptide A2	UGT3A2					
Y82E9BL.10	WBGene00022326	fbxa-14	-0.950625201207243	0.004339155	-								
C27A12.5	WBGene00000429	ceh-2	-0.950625201207243	0.005538124	-								
Y57G11C.39	WBGene00013328	-	-0.949625201207243	0.004276542	-								
T22F7.1	WBGene00020701	-	-0.946625201207243	0.005381082	-								
C06A12.5	WBGene00007358	lact-6	-0.944625201207243	0.004375276	-								
R07A4.2	WBGene00011073	-	-0.943625201207243	0.004538463	-								
W06B11.3	WBGene00021060	-	-0.943625201207243	0.009134224	-								
C05D2.4	WBGene00000239	bas-1	-0.941625201207243	0.004339155	-								
Y37B11A.1	WBGene00021343	-	-0.941625201207243	0.004343696	-								
K06B4.11	WBGene00003643	nhr-53	-0.941625201207243	0.009374041	-								
C09B8.3	WBGene00015622	-	-0.940625201207243	0.00438174	-								
Y82E9BL.11	WBGene00022327	fbxa-15	-0.940625201207243	0.005081072	-								
C49G7.7	WBGene00016785	-	-0.939625201207243	0.004371558	-								
C24F3.3	WBGene00003531	nas-12	-0.939625201207243	0.004409821	-								
C49A1.10	WBGene00008191	-	-0.937625201207243	0.004339155	-								
Y71G12B.26	WBGene00022161	-	-0.936625201207243	0.005783595	-								
R12E2.4a	WBGene00002139	inx-17	-0.936625201207243	0.00724686	-								
R12E2.4b	WBGene00002139	inx-17	-0.934625201207243	0.00437505	-								
Y11D7A.5	WBGene00012429	-	-0.934625201207243	0.004495388	-								
C24B5.3	WBGene00004216	ptr-1	-0.926625201207243	0.00561291	-								
H05L03.3	WBGene00019160	-	-0.924625201207243	0.005758233	-								
C09E8.2b	WBGene00015645	-	-0.918625201207243	0.007529441	-								
F21E9.3	WBGene00017667	-	-0.911625201207243	0.005149113	-								
F10G7.7	WBGene00017371	sre-39	-0.911625201207243	0.006139553	-								
Y67A6A.2	WBGene00003652	nhr-62	-0.910625201207243	0.00474843	-								
T01D3.3b	WBGene00011328	-	-0.908625201207243	0.00790154	-								
F17C8.2	WBGene00000664	col-89	-0.907625201207243	0.009091782	-								
K08B12.1	WBGene00019520	-	-0.904625201207243	0.0049566	-								
C02F4.4	WBGene00007264	-	-0.903625201207243	0.005914989	-								
F14F3.1a	WBGene00006870	vab-3	-0.902075	0.00918887	ENSP00000 368424	ENSG000 00007372	Paired box gene 6 (aniridia, keratitis)	PAX6	0.692	2.54e-08	0.763		3.87e-07
C49F8.1	WBGene00008213	-	-0.901625201207243	0.006332147	-								
C53D6.8	WBGene00008285	-	-0.900625201207243	0.009016155	-								

Y38H6A.3	WBGene00012614	-	-0.898625201207243	0.004627741	-								
W04B5.3a	WBGene00021020	-	-0.898625201207243	0.008986931	-								
F21G4.1	WBGene00009023		-0.895944	0.00990921	ENSP00000311291	ENSG00000174640	solute carrier organic anion transporter family, member 2A1	SLCO2A1	-0.00276	0.951	0.0577	0.401	
F02D8.5	WBGene00008522	-	-0.893625201207243	0.00673075	-								
C52D10.1	WBGene00016875	-	-0.893625201207243	0.007707666	-								
K02G10.1	WBGene00019344	-	-0.893625201207243	0.007800971	-								
Y55F3AM.5	WBGene00021923	-	-0.891625201207243	0.005327441	-								
F54F3.4	WBGene00010063	-	-0.890625201207243	0.005177616	-								
Y69E1A.8	WBGene00013478	-	-0.890625201207243	0.007429724	-								
M01H9.2	WBGene00019718	-	-0.890625201207243	0.00873001	-								
F52B11.3	WBGene00009926	noah-2	-0.888625201207243	0.004657633	-								
F35B3.4	WBGene00018031	-	-0.888625201207243	0.005149113	-								
F37B1.3	WBGene00001762	gst-14	-0.883625201207243	0.005530379	-								
C08F11.4	WBGene00005684	sru-21	-0.883625201207243	0.006653039	-								
ZK250.8	WBGene00022691	math-50	-0.881625201207243	0.005724044	-								
F44F4.1	WBGene00009700	-	-0.880625201207243	0.008695512	-								
F19C7.1	WBGene00017591	-	-0.878625201207243	0.005126736	-								
C27A2.5	WBGene00016153	-	-0.877625201207244	0.005070931	-								
R02C2.4	WBGene00019816	nhr-204	-0.875625201207244	0.005724044	-								
K08F8.7	WBGene00010683	-	-0.875625201207244	0.005986932	-								
K02F6.5	WBGene00019339	-	-0.875625201207244	0.007229612	-								
C39D10.5	WBGene00016532	-	-0.874625201207244	0.007064796	-								
Y53F4B.35	WBGene00001779	gst-31	-0.873625201207244	0.008424118	-								
C06C3.5	WBGene00007376	-	-0.873625201207244	0.009392801	-								
ZC487.1	WBGene00022623	-	-0.871625201207244	0.007577467	-								
Y23H5A.5c	WBGene00000832	ctn-1	-0.86774	0.009719	ENSP00000320434	ENSG00000119326	catenin (cadherin-associated protein), alpha-like 1	CTNNAL1	0.149	0.0217	0.162	0.0926	
F36H5.10	WBGene00018108	-	-0.867625201207244	0.005724044	-								
T07F10.6	WBGene00011592	-	-0.867625201207244	0.00690017	-								
C01G10.16	WBGene00007241	-	-0.865625201207244	0.007392143	-								
F41D9.2	WBGene00018282	-	-0.858625201207244	0.005763964	-								
B0432.5a	WBGene00000296	cat-2	-0.857625201207244	0.00828115	-								

F44G3.8	WBGene00009709	fbxa-144	-0.855625201207244	0.009354811	-
B0272.4	WBGene00007130	-	-0.844625201207243	0.006667505	-
E03G2.2	WBGene00003409	mrp-3	-0.844625201207243	0.01007944	-
C06E1.1	WBGene00015518	-	-0.842625201207243	0.007569064	-
Y50C1A.2	WBGene00021734	-	-0.841625201207243	0.009618557	-
C30G4.3	WBGene00001537	gcy-11	-0.839625201207243	0.00669748	-
ZK1290.8	WBGene00006956	wrt-10	-0.837625201207243	0.004146482	-
F55G11.7	WBGene00010127	-	-0.834625201207243	0.006146711	-
F58B4.1b	WBGene00003549	nas-31	-0.831625201207243	0.00719871	-
F53E10.4	WBGene00018760	-	-0.828625201207243	0.006900026	-
Y57A10A.14	WBGene00013257	-	-0.828625201207243	0.007698842	-
W09G10.3	WBGene00021116	-	-0.828625201207243	0.008021364	-
Y37E3.5	WBGene00021349	-	-0.827625201207243	0.006584802	-
T16H5.1b	WBGene00002141	inx-19	-0.825625201207243	0.00724686	-
C06E4.2	WBGene00015531	-	-0.825625201207243	0.007906905	-
Y34B4A.9	WBGene00021325	-	-0.824625201207243	0.005999791	-
Y45F3A.8	WBGene00012863	-	-0.824625201207243	0.006144164	-
C44C3.6	WBGene00005864	srw-117	-0.821625201207243	0.009659695	-
C42D8.1	WBGene00016599	-	-0.819625201207243	0.006809472	-
F01D5.10	WBGene00008500	-	-0.816625201207243	0.008530822	-
Y47D7A.5	WBGene00001714	grl-5	-0.815625201207243	0.006460281	-
T12A2.11	WBGene00005163	srg-5	-0.812625201207243	0.007295689	-
F41E7.4	WBGene00009620	fip-5	-0.811625201207243	0.00878557	-
K02E7.5	WBGene00019310	-	-0.808625201207243	0.006033838	-
F41D9.5	WBGene00018283	sulp-3	-0.806625201207243	0.007098263	-
F47B10.7	WBGene00009818	acbp-3	-0.804625201207243	0.007076687	-
F10F2.9	WBGene00004118	pqn-29	-0.804625201207243	0.007100107	-
F14B8.6	WBGene00017448		-0.801625201207243	0.00957275	-
C29F7.2	WBGene00007811	-	-0.795625201207243	0.006620091	-
C52A10.1	WBGene00016862	-	-0.794625201207243	0.007808274	-
C06E7.4	WBGene00015541	-	-0.789625201207243	0.007355692	-
ZK770.3	WBGene00002134	inx-12	-0.785625201207243	0.00720738	-
F56G4.1	WBGene00010157	-	-0.783625201207243	0.008441392	-
K09G1.1b	WBGene00010727	-	-0.781625201207243	0.00724686	-
F15E6.7	WBGene00005715	srv-4	-0.781625201207243	0.008618473	-
Y59E9AL.5	WBGene00021995	-	-0.779625201207243	0.007348463	-
Y77E11A.14	WBGene00022314	-	-0.775625201207243	0.008677599	-
C26F1.5	WBGene00001719	grl-10	-0.773625201207243	0.01003284	-
F55A4.3	WBGene00018855	-	-0.772625201207243	0.00771431	-

F01D4.6a	WBGene00003167	mec-3	-0.763625201207243	0.008453117	-							
Y22D7AL.14	WBGene00021252	-	-0.745625201207244	0.00744196	-							
C05C8.7	WBGene00015464	-	-0.745625201207244	0.008119108	-							
ZK218.11	WBGene00013943		-0.736625201207244	0.009872366	-							
W01D2.2	WBGene00003651	nhr-61	-0.733625201207244	0.009964752	-							
Y48B6A.4	WBGene00001133	eat-2	-0.730625201207244	0.009259474	-							
K06B4.2	WBGene00003642	nhr-52	-0.722625201207243	0.009994071	-							
C06H5.2	WBGene00007393	fbxa-157	-0.706625201207243	0.009872366	-							
C02E7.7	WBGene00015340	-	-0.703625201207243	0.008469742	-							
K04G2.9	WBGene00010567	-	-0.693625201207243	0.009077459	-							
B0213.6	WBGene00003769	nlp-31	-0.683625201207243	0.009151287	-							
C29H12.3a	WBGene00004346	rgs-3	-0.674625201207243	0.009393675	-							
F46C8.6	WBGene00001069	dpy-7	-0.660625201207243	0.007134214	-							
F46E10.2	WBGene00018489	-	-0.505625201207244	0.004340992	-							
F45F2.11	WBGene00018483	-	0.232374798792757	0.006900026	-							
ZK6.7b	WBGene00022642		0.504457	0.000606466	ENSP00000238983	ENSG00000182333	lipase, gastric	LIPF	0.0401	0.146	0.0248	0.535
W08E12.8	WBGene00021089	-	0.579374798792757	0.0001953086	-							
F53B1.2	WBGene00018735		0.649374798792757	0.01009056	-							
C09B9.4	WBGene00015629	-	0.718374798792757	0.0076038	-							
C01F1.1	WBGene00015296		0.719374798792757	0.00983942	-							
F43G9.10	WBGene00009671	-	0.732374798792757	0.009136765	-							
F54B3.1	WBGene00010013	-	0.741374798792757	0.008157576	-							
K04D7.5	WBGene00001653	gon-4	0.744374798792757	0.009234635	-							
Y39G8B.3	WBGene00012724	sre-48	0.748374798792756	0.009941195	-							
K08H10.7	WBGene00004323	rde-1	0.761374798792756	0.007764877	-							
M28.1	WBGene00010893	-	0.768374798792756	0.00002366637	-							
F45D3.4	WBGene00009724	-	0.785374798792757	0.00965754	-							
B0554.7	WBGene00015260	-	0.787374798792757	0.006235202	-							
ZK1321.3	WBGene00000178	aqp-10	0.793374798792757	0.007678161	-							
C33C12.1	WBGene00016334	-	0.793374798792757	0.00830888	-							
C14E2.6	WBGene00015775	-	0.796374798792757	0.005884285	-							
K01C8.5	WBGene00001571	gei-14	0.823374798792757	0.008992466	-							
C51E3.1	WBGene00008247	srsx-26	0.829374798792757	0.007617	-							
T03F7.3	WBGene00005269	srh-46	0.833374798792757	0.007816425	-							
F46B6.12	WBGene00009776		0.833374798792757	0.009941195	-							
C32D5.1	WBGene00016310	-	0.836374798792757	0.006088313	-							
C09E7.9	WBGene00015643	-	0.840374798792757	0.006192842	-							
R09F10.1	WBGene00019986	-	0.846374798792757	0.003092405	-							
C35C5.1	WBGene00004746	sdc-2	0.846374798792757	0.00589303	-							

ZK328.4	WBGene00022694	-	0.847374798792757	0.008432308	-								
T05G5.1	WBGene00011498	-	0.857374798792757	0.003920001	-								
Y38E10A.22	WBGene00012600	-	0.857374798792757	0.006443052	-								
W02D3.8			0.859374798792757	0.01002007	-								
C23H4.6	WBGene00007694		0.863374798792757	0.009709793	-								
F35B3.7	WBGene00018033	-	0.864374798792757	0.009576075	-								
F09G2.4	WBGene00017313		0.864374798792757	0.00977749	-								
F20D12.2	WBGene00017642	-	0.872374798792756	0.006199184	-								
C05C10.2b	WBGene00007329	-	0.873374798792756	0.005654933	-								
R02D3.7	WBGene00019824	-	0.875374798792756	0.005688866	-								
F02E11.5	WBGene00017183	scl-15	0.876016	0.00255567	ENSP00000362778	ENSG00000164530	protease inhibitor 16	PI16	0.0436	0.16	-0.00847	0.85	
F57C9.4a	WBGene00019011	-	0.876374798792756	0.009077459	-								
K05C4.4	WBGene00010581	-	0.877374798792756	0.005611082	-								
T21B10.5	WBGene00011887	set-17	0.877374798792756	0.008531606	-								
C03B1.3	WBGene00015374		0.892374798792756	0.00942084	-								
ZK1251.8	WBGene00006664	twk-9	0.895374798792756	0.005232609	-								
ZC328.4	WBGene00004721	san-1	0.895374798792756	0.009863866	-								
Y50D7A.2	WBGene00021752		0.900542	0.0070544	ENSP00000221481	ENSG00000104884	excision repair cross-complementing rodent repair deficiency, complementation group 2 (xeroderma pigmentosum D)	ERCC2	-0.051	0.229	-0.0966	0.0722	
T16G1.7	WBGene00011801	-	0.909374798792757	0.007906167	-								
C39B10.3	WBGene00008023	-	0.915374798792757	0.004814446	-								
F59H6.9	WBGene00019139	bath-1	0.915374798792757	0.009941195	-								
F25B5.1	WBGene00017773	-	0.920374798792757	0.008158383	-								
Y4C6B.3	WBGene00021157	-	0.925374798792757	0.006139553	-								
D1081.9	WBGene00008387	-	0.932374798792757	0.008552694	-								
Y57G11A.3	WBGene00013291		0.932425	0.00890536	ENSP00000259006	ENSG00000136490	LIM domain containing 2	LIMD2					
R07B7.10	WBGene00011094	-	0.938374798792757	0.00346809	-								
R05D3.1	WBGene00019876	-	0.938374798792757	0.006174748	-								
ZK112.3	WBGene00022659	-	0.942374798792757	0.006582745	-								
M79.3	WBGene00010903	-	0.946374798792757	0.004260303	-								

ZC487.3	WBGene00022625	-	0.951374798792756	0.00785476	-								
F13A7.9	WBGene00004817	skr-11	0.954374798792757	0.00447951	-								
K10B3.9	WBGene00003124	mai-1	0.956374798792757	0.005193423	-								
M02F4.8	WBGene00000175	aqp-7	0.966374798792757	0.006872929	-								
C08F8.2	WBGene00007444		0.96676	0.00890536	ENSP00000352678	ENSG00000156502	suppressor of var1, 3-like 1 (S. cerevisiae)	SUPV3L1	-0.061	0.145	-0.00419	0.938	
C17A2.2	WBGene00015870	-	0.976374798792757	0.002578936	-								
R107.4c	WBGene00011299	ikke-1	0.976374798792757	0.003930252	-								
T02C12.4	WBGene00011369	-	0.976374798792757	0.007569064	-								
F18C5.3	WBGene00007050	tag-184	0.979023	0.00659622	ENSP00000261637	ENSG00000120800	down-regulated in metastasis	DRIM	0.0354	0.403	0.15	0.0197	
F35C8.4	WBGene00006371	syn-1	0.979374798792756	0.003707137	-								
F59B10.4a	WBGene00010320	-	0.981374798792756	0.003859597	-								
T22E7.1a	WBGene00020693	-	0.994374798792757	0.005688866	-								
F52C6.8	WBGene00018665	bath-4	0.997374798792756	0.009658444	-								
T05G5.9b	WBGene00011503	-	0.998374798792757	0.004240749	-								
C43E11.2b	WBGene00016602	mus-81	0.998643	0.00962708	ENSP00000307853	ENSG00000172732	MUS81 endonuclease homolog (yeast)	MUS81	0.0145	0.641	-0.0253	0.608	
C14A4.4a	WBGene00000796	crn-3	1.0011	0.00601155	ENSP00000366135	ENSG00000171824	exosome component 10	EXOSC10	0.218	0.000342	0.000351	0.997	
C55B7.5	WBGene00016944	uri-1	1.0011	0.00690381	ENSP00000312530	ENSG00000105176	chromosome 19 open reading frame 2	C19orf2	0.108	0.00214	-0.0662	0.159	
T07C12.12	WBGene00011576	-	1.00137479879276	0.007355692	-								
C56E6.6	WBGene00016974	-	1.00437479879276	0.006247592	-								
Y32G9A.10	WBGene00021307	-	1.00437479879276	0.009214745	-								
Y55D5A.4	WBGene00021918	-	1.00537479879276	0.005149113	-								
F15A8.4	WBGene00017477	-	1.00537479879276	0.008279879	-								
R144.10	WBGene00020098	-	1.00737479879276	0.005257767	-								
F22E5.9	WBGene00017708	-	1.00737479879276	0.009697943	-								
F54G8.5	WBGene00004223	ptr-9	1.00837479879276	0.003421828	-								
C26C6.7	WBGene00007741	-	1.00837479879276	0.00443009	-								
C17B7.11	WBGene00015885	fbxa-65	1.00837479879276	0.009600638	-								
M01E11.3	WBGene00019712	-	1.01037479879276	0.006372875	-								
T20D4.19	WBGene00020624	-	1.01237479879276	0.004412563	-								
K08E4.3	WBGene00010667	-	1.01237479879276	0.00709945	-								

M01G12.12	WBGene00004509	rrf-2	1.01437479879276	0.006332147	-							
W02A2.2	WBGene00001390	far-6	1.01437479879276	0.007529916	-							
F59C6.3	WBGene00010324	-	1.01437479879276	0.007575467	-							
R03E9.2	WBGene00019843	-	1.01537479879276	0.003732448	-							
W02B12.6	WBGene00000196	ars-1	1.01537479879276	0.009393675	-							
F33C8.4	WBGene00009358	-	1.01737479879276	0.00327412	-							
M04B2.2	WBGene00010846	-	1.01737479879276	0.005414028	-							
F28F5.6	WBGene00017905	-	1.01737479879276	0.008943946	-							
F20A1.1	WBGene00017613	-	1.01837479879276	0.005305082	-							
ZC155.7	WBGene00022534	syn-16	1.02137479879276	0.008649213	-							
T20B12.8	WBGene00001974	hmg-4	1.02194	0.00744931	ENSP00000278412	ENSG00000149136	structure specific recognition protein 1	SSRP1	0.116	0.00931	-0.0929	0.228
ZK945.4	WBGene00014166	-	1.02537479879276	0.004966369	-							
Y38A10A.6	WBGene00021391	-	1.02737479879276	0.003733993	-							
R05D3.4	WBGene00007008	rfp-1	1.02737479879276	0.009658444	-							
F15G9.6	WBGene00008869	-	1.02837479879276	0.001743451	-							
W10C6.1	WBGene00003133	mat-2	1.0293	0.0086869	ENSP00000339109	ENSG00000153107	anaphase promoting complex subunit 1	ANAPC1	-0.0668	0.0827	-0.0532	0.318
K07C5.2	WBGene00010625	-	1.03037479879276	0.003389665	-							
R04A9.1	WBGene00019861	-	1.03437479879276	0.003605114	-							
T04A8.11	WBGene00011412		1.03543	0.00912754	ENSP00000300151	ENSG00000166902	mitochondrial ribosomal protein L16	MRPL16	0.0331	0.417	-0.0524	0.376
C09H10.6	WBGene00007500	nasp-1	1.03543	0.00759537	ENSP00000345532	ENSG00000132780	nuclear autoantigenic sperm protein (histone-binding)	NASP	0.241	3.45e-05	0.0264	0.627
F29B9.8	WBGene00017923	-	1.04037479879276	0.003391735	-							
Y39A3CR.8	WBGene00021447	-	1.04137479879276	0.01008327	-							
T16G12.8	WBGene00011809	-	1.04737479879276	0.004339155	-							
ZK546.5	WBGene00022762	-	1.05537479879276	0.005109592	-							
Y51A2D.9	WBGene00013077	-	1.06037479879276	0.005986932	-							
M05D6.1	WBGene00010874	-	1.06337479879276	0.006084458	-							
T13A10.7	WBGene00005741	srv-30	1.06637479879276	0.006157172	-							
R05G6.8	WBGene00004039	plc-4	1.06731	0.0060279	ENSP00000313731	ENSG00000161714	phospholipase C, delta 3	PLCD3	0.0192	0.62	-0.0716	0.127

T23D8.3	WBGene00011944		1.06731	0.00591166	ENSP00000356548	ENSG00000135521							
T27E7.6	WBGene00012091	-	1.06737479879276	0.004221737	-								
F18C5.2	WBGene00006944	wrn-1	1.06977	0.00590021	ENSP00000298139	ENSG00000165392	Werner syndrome	WRN	0.0333	0.293	0.0182	0.662	
R10H1.1	WBGene00019998	-	1.07137479879276	0.004492112	-								
F47G9.4	WBGene00009831	-	1.07137479879276	0.004884213	-								
K07A12.7	WBGene00010624		1.07467	0.00984332	ENSP00000362208	ENSG00000116898	mitochondrial ribosomal protein S15 /// mitochondrial ribosomal protein S15	MRPS15	-0.168	8.31e-06	-0.12	0.00231	
K11G12.4	WBGene00004876	smf-1	1.07637479879276	0.002633325	-								
B0212.3	WBGene00015037	-	1.07737479879276	0.005056911	-								
F27D9.5	WBGene00017864	pcca-1	1.07958	0.00832285	ENSP00000365463	ENSG00000175198	propionyl Coenzyme A carboxylase, alpha polypeptide	PCCA	-0.0127	0.703	-0.0403	0.437	
R12C12.7	WBGene00020027	-	1.08037479879276	0.004048031	-								
B0252.3b	WBGene00015088	-	1.08137479879276	0.004470968	-								
R13A1.7	WBGene00020044	-	1.08137479879276	0.005033545	-								
ZK177.8b	WBGene00022673		1.08203	0.0086469	ENSP00000262878	ENSG00000101347	SAM domain and HD domain 1	SAMHD1	0.563	8.24e-07	0.218	0.0295	
F55F8.2b	WBGene00018890		1.08326	0.00972838	ENSP00000328690	ENSG00000089737	DEAD (Asp-Glu-Ala-Asp) box polypeptide 24	DDX24	-0.219	0.000424	-0.0016	0.979	
C47E8.8	WBGene00008145	set-5	1.08326	0.0055598	ENSP00000354874	ENSG00000181013							
F52E10.2	WBGene00009933	-	1.08337479879276	0.007808274	-								
T06G6.9	WBGene00006889	pfd-3	1.08693	0.00850442	ENSP00000286428	ENSG00000155959	von Hippel-Lindau binding protein 1	VBP1	-0.107	0.17	-0.244	0.000648	
C06E8.3a	WBGene00004182	prk-1	1.08937479879276	0.006809472	-								
F46E10.10a	WBGene00018491		1.09184	0.0091978	ENSP00000233114	ENSG00000014641	Malate dehydrogenase 1, NAD (soluble)	MDH1	-0.295	2.43e-07	-0.0163	0.895	

F41C3.3	WBGene00018269	acs-11	1.09184	0.00970824	ENSP00000 320646	ENSG000 00176715						
F28C6.5	WBGene00009205	-	1.09437479879276	0.002976113	-							
F38E11.2	WBGene00002013	hsp-12.6	1.09552	0.00624327	ENSP00000 227251	ENSG000 00109846	crystallin, alpha B	CRYAB	0.478	2.2e-05	0.0902	0.596
F43H9.3	WBGene00018399	-	1.09937479879276	0.006569753	-							
C26B2.7	WBGene00016129	-	1.09937479879276	0.008573972	-							
F28B3.10	WBGene00017889	-	1.10637479879276	0.006900026	-							
T04A8.15	WBGene00011415	-	1.10937479879276	0.002636893	-							
K08E7.3	WBGene00002368	let-99	1.11337479879276	0.004050871	-							
C27A12.2	WBGene00016154	-	1.11337479879276	0.00944893	-							
F29G9.5	WBGene00004502	rpt-2	1.11391	0.00795149	ENSP00000 261303	ENSG000 00100764	proteasome (prosome, macropain) 26S subunit, ATPase, 1	PSMC1	-0.0199	0.619	-0.123	0.0368
B0414.5	WBGene00000772	cpb-3	1.11437479879276	0.002540478	-							
ZK546.14b	WBGene00022765		1.11514	0.00380517	ENSP00000 361092	ENSG000 00148296	surfeit 6	SURF6	0.0889	0.0143	-0.0976	0.0809
C46G7.1	WBGene00016721	-	1.11637479879276	0.004706151	-							
C05D11.2	WBGene00006516	vps-16	1.11637479879276	0.006105483	-							
F26H9.8	WBGene00009178	-	1.11737479879276	0.002733235	-							
M05D6.7	WBGene00001523	gbh-2	1.11882	0.00601155	ENSP00000 335261	ENSG000 00185973	trimethyllysine hydroxylase, epsilon	TMLHE	-0.0396	0.299	0.0404	0.464
D1022.7a	WBGene00000101	aka-1	1.1225	0.00496182	ENSP00000 287727	ENSG000 00157077	zinc finger, FYVE domain containing 9	ZFYVE9	-0.0618	0.0385	-0.206	0.0179
ZK643.1	WBGene00014033	-	1.12437479879276	0.0076038	-							
F55C5.3a	WBGene00006677	twk-24	1.12637479879276	0.009328746	-							
R10H10.7	WBGene00011225	-	1.12737479879276	0.004111019	-							
T24B1.1	WBGene00011975		1.13476	0.00690381	ENSP00000 163416	ENSG000 00066455	golgi autoantigen, golgin subfamily a, 5	GOLGA5	0.113	0.0393	0.0298	0.622
W06D4.4	WBGene00012298		1.13476	0.00489958	ENSP00000 343103	ENSG000 00132600	protein arginine N-methyltransferase 7	PRMT7	-0.209	4.47e-05	-0.171	0.0298
K10D6.1	WBGene00010741	-	1.13937479879276	0.005688866	-							

F45E1.3	WBGene00018464	-	1.13937479879276	0.007698842	-							
W05B10.2	WBGene00012277	-	1.14037479879276	0.005092069	-							
K10D11.4	WBGene00010748	-	1.14637479879276	0.003947947	-							
F22B7.7	WBGene00006662	twk-7	1.14825	0.0086469	ENSP00000334650	ENSG00000186795	potassium channel, subfamily K, member 18	KCNK18				
ZC190.5	WBGene00022539	-	1.14937479879276	0.008351892	-							
F21H12.6	WBGene00017686		1.1507	0.00687546	ENSP00000365220	ENSG00000134900	tripeptidyl peptidase II	TPP2	0.013	0.807	-0.0335	0.645
C06B8.9	WBGene00005768	srw-21	1.15237479879276	0.002951596	-							
T14D7.1	WBGene00011767		1.15683	0.00634095	ENSP00000302620	ENSG00000172482						
C32H11.7	WBGene00007870	-	1.15737479879276	0.005986932	-							
F08F1.4a	WBGene00017258	-	1.15737479879276	0.009062055	-							
K11C4.5	WBGene00006801	unc-68	1.15928	0.00809902	ENSP00000355533	ENSG00000198626	ryanodine receptor 2 (cardiac)	RYR2	-0.73	1.03e-06	0.14	0.227
T11F9.2b	WBGene00006486	tag-140	1.15937479879276	0.003707137	-							
C38D4.4	WBGene00008005	-	1.16037479879276	0.003092405	-							
C08A9.9	WBGene00015589	-	1.16337479879276	0.002162307	-							
Y17G9B.4	WBGene00021201	-	1.16337479879276	0.002493727	-							
M57.1	WBGene00019777	-	1.16937479879276	0.004340453	-							
T12G3.8	WBGene00000270	bre-5	1.17032	0.00987439	ENSP00000323479	ENSG00000169255	UDP-Gal:betaGlcNAc beta 1,3-galactosyltransferase, polypeptide 3	B3GALT3	-0.538	7.17e-06	-0.208	0.165
T10G3.5	WBGene00011696	eea-1	1.17155	0.00612713	ENSP00000317955	ENSG00000102189	Early endosome antigen 1, 162kD	EEA1	0.14	0.0441	0.0108	0.921
D2013.9	WBGene00008405	ttll-12	1.17155	0.00487666	ENSP00000216129	ENSG00000100304	KIAA0153 protein	KIAA0153	-0.0492	0.192	0.00429	0.912
C01G5.2	WBGene00015304	tag-292	1.18337	0.002975666	ENSP00000245255	ENSG00000125207	piwi-like 1 (Drosophila)	PIWIL1	-0.0346	0.107	0.0162	0.534
ZK822.1	WBGene00014089	-	1.18337479879276	0.002025854	-							
C14A11.5	WBGene00015750	-	1.18337479879276	0.00524152	-							

F32D8.10	WBGene00009333	-	1.18537479879276	0.004966369	-								
F56A3.4	WBGene00004955	spd-5	1.18837479879276	0.0049566	-								
F21C3.4	WBGene00004324	rde-2	1.19137479879276	0.001844176	-								
C28G1.5	WBGene00016190	-	1.19137479879276	0.005020497	-								
K07D4.8	WBGene00004135	pqn-48	1.19137479879276	0.007013752	-								
C06G4.1	WBGene00015557	-	1.19237479879276	0.001934293	-								
F30F8.1	WBGene00009270	-	1.19337479879276	0.005884285	-								
Y37E3.4	WBGene00021348	-	1.19537479879276	0.004470968	-								
Y54E10A.12	WBGene00021832	-	1.19637479879276	0.008826258	-								
B0523.3	WBGene00003993	pgl-2	1.19937479879276	0.002613426	-								
F42G10.1	WBGene00009645	-	1.20337479879276	0.003947947	-								
W04C9.4	WBGene00021026	-	1.20637479879276	0.002975666	-								
T14B4.1	WBGene00020498		1.20711	0.00440086	ENSP00000040877	ENSG00000059588	TAR (HIV) RNA binding protein 1	TARBP1	-0.204	0.00266	-0.11	0.27	
F41G4.1	WBGene00018307		1.20834	0.00983081	ENSP00000296218	ENSG00000163879	dynein, axonemal, light intermediate polypeptide 1	DNALI1	0.375	0.000573	0.0409	0.379	
F58B3.4	WBGene00010231		1.20834	0.00382856	ENSP00000202816	ENSG00000089048	chromosome 20 open reading frame 6	C20orf6	0.187	0.00217	0.0486	0.386	
C16A3.3	WBGene00015808	-	1.20937479879276	0.001503824	-								
Y82E9BR.20	WBGene00022352	-	1.20937479879276	0.003339028	-								
C33D9.1b	WBGene00001366	exc-5	1.21324	0.00433514	ENSP00000364277	ENSG00000102302	FYVE, RhoGEF and PH domain containing 1 (faciogenital dysplasia)	FGD1	0.0945	0.000768	0.0587	0.203	
B0205.11	WBGene00015025		1.21692	0.00747599	ENSP00000357823	ENSG00000143436	mitochondrial ribosomal protein L9	MRPL9	-0.0381	0.49	0.00183	0.97	
F16H6.5	WBGene00008895	-	1.21837479879276	0.00410237	-								
Y17G9B.5	WBGene00021202		1.21937	0.00652678	ENSP00000270517	ENSG00000130159	ECSIT homolog (Drosophila)	ECSIT					
Y97E10AR.2a	WBGene00022397	-	1.22137479879276	0.005149113	-								
F53F1.3	WBGene00009981	-	1.22437479879276	0.001788907	-								
K02B12.5	WBGene00010498	-	1.22437479879276	0.004298294	-								
K07C5.3	WBGene00010626	-	1.22837479879276	0.001615897	-								

Y102A5C.2	WBGene00013611	-	1.23137479879276	0.002723606	-								
D1081.7	WBGene00008385	-	1.23137479879276	0.003904741	-								
T24D8.1	WBGene00020767	-	1.23537479879276	0.00347649	-								
W02H5.6	WBGene00005477	srh-271	1.23537479879276	0.00404848	-								
ZK1127.11	WBGene00001872	him-14	1.23537479879276	0.006214731	-								
Y45F10C.4	WBGene00012880	-	1.24137479879276	0.001345628	-								
C17H12.10	WBGene00015935	-	1.24237479879276	0.002556719	-								
Y51A2D.12	WBGene00005191	srg-34	1.24837479879276	0.003423899	-								
Y57G11C.7	WBGene00013305	-	1.25137479879276	0.004470968	-								
C34B2.6	WBGene00016391		1.25371	0.0094291	ENSP00000 353826	ENSG000 00196365	protease, serine, 15	PRSS15	-0.000377	0.994	0.00495	0.925	
F13A7.3	WBGene00005745	srv-34	1.25437479879276	0.002170114	-								
Y97E10B.6	WBGene00005902	srx-11	1.25437479879276	0.004340453	-								
C17G10.2	WBGene00015916	-	1.25737479879276	0.009259474	-								
D2023.5	WBGene00008409	-	1.25837479879276	0.006146586	-								
F46E10.10b	WBGene00018491		1.25984	0.00349789	ENSP00000 233114	ENSG000 00014641	Malate dehydrogenase 1, NAD (soluble)	MDH1	-0.295	2.43e-07	-0.0163	0.895	
F39C12.1	WBGene00018193	-	1.26437479879276	0.003859597	-								
M03E7.5	WBGene00019757	-	1.26537479879276	0.005048164	-								
C35D10.6	WBGene00016443	-	1.26737479879276	0.003973433	-								
K07B1.4b	WBGene00019464		1.26842	0.00812444	ENSP00000 198801	ENSG000 00166391	monoacylglycer ol O-acyltransferase 2	MOGAT2	-0.0223	0.544	0.0856	0.149	
M70.5	WBGene00019787	-	1.27237479879276	0.003945016	-								
C09E7.7	WBGene00015641	-	1.27437479879276	0.001534794	-								
C06E4.8	WBGene00015537	-	1.27537479879276	0.002801685	-								
T23F6.4	WBGene00004315	rbd-1	1.27701	0.00404273	ENSP00000 261741	ENSG000 00122965	RNA binding motif protein 19	RBM19	0.0162	0.641	-0.0361	0.458	
T05H10.2	WBGene00000151	apn-1	1.27737479879276	0.001520771	-								
T06E4.3b	WBGene00000226	atl-1	1.27737479879276	0.00232069	-								
C16A11.5	WBGene00015818	-	1.27737479879276	0.00848387	-								
F41H10.4	WBGene00018317	-	1.27837479879276	0.001790244	-								
B0416.7	WBGene00015182	-	1.28037479879276	0.00327412	-								
F07C6.4b	WBGene00008555	-	1.28137479879276	0.00443009	-								
C16C10.10	WBGene00006448	glod-4	1.28436	0.00434768	ENSP00000 301328	ENSG000 00167699	Chromosome 17 open reading frame 25	C17orf25	-0.0775	0.0255	0.0547	0.174	

F08H9.1	WBGene00000592	coh-3	1.28437479879276	0.004276542	-							
T04F8.1	WBGene00011440	sfxn-1.5	1.28804	0.00743101	ENSP00000316905	ENSG00000164466	sideroflexin 1	SFXN1	-0.0645	0.115	0.0208	0.802
F52F12.7	WBGene00009940		1.29785	0.00367581	ENSP00000276449	ENSG00000147465	steroidogenic acute regulator	STAR	-0.241	9.34e-06	-0.0551	0.447
F21H7.2	WBGene00009028	-	1.30037479879276	0.001618805	-							
F28F8.8	WBGene00009225	fbxa-94	1.30037479879276	0.004202926	-							
F44G4.1	WBGene00009711		1.30398	0.00359988	ENSP00000359688	ENSG00000117133	RNA processing factor 1	RPF1	-0.0814	0.0379	-4.82e-05	0.999
T23B12.7	WBGene00001040	dnj-22	1.30398	0.0072356	ENSP00000220496	ENSG00000104129						
F23B2.6	WBGene00000121	aly-2	1.30437479879276	0.002331588	-							
D2030.6	WBGene00008416	-	1.30437479879276	0.003497553	-							
K01C8.9	WBGene00003821	nst-1	1.30521	0.00659622	ENSP00000338573	ENSG00000130119	Guanine nucleotide binding protein-like 3 (nucleolar)-like	GNL3L	0.0513	0.0805	0.00454	0.92
F16C3.1	WBGene00008885	-	1.30537479879276	0.001957205	-							
T10E9.2	WBGene00020412	-	1.30537479879276	0.0076038	-							
Y116F11A.5	-	-	1.30737479879276	0.001430322	-							
Y39H10A.2	WBGene00021479	-	1.30837479879276	0.006807328	-							
C34F11.3b	WBGene00016415		1.31134	0.00628724	ENSP00000358858	ENSG00000116337	adenosine monophosphate deaminase 2 (isoform L)	AMPD2	-0.554	7.81e-09	-0.0245	0.682
F32E10.2	WBGene00017990	-	1.31437479879276	0.001831184	-							
Y41D4B.7	WBGene00003677	nhr-87	1.31737479879276	0.007819323	-							
F18C5.10	WBGene00017563	-	1.31837479879276	0.002585252	-							
F16H6.7	WBGene00008897	-	1.31837479879276	0.003065188	-							
ZK177.4b	WBGene00022672	-	1.32137479879276	0.002299128	-							
C15A11.2	WBGene00007597	-	1.32137479879276	0.002582442	-							
F55G1.4	WBGene00018900	rod-1	1.32437479879276	0.00201794	-							
C10G8.8b	WBGene00015684		1.32606	0.00847862	ENSP00000221922	ENSG00000105321	coiled-coil domain containing 9	CCDC9	0.027	0.465	0.0231	0.546
C25E10.5	WBGene00016095	-	1.32837479879276	0.001823923	-							
DH11.2	WBGene00008436	-	1.32837479879276	0.00307325	-							

C33H5.17	WBGene00016383		1.32851	0.00350316	ENSP00000348669	ENSG00000197114	zinc finger, CCCH-type with G patch domain	ZGPAT	-0.0484	0.129	0.0321	0.611
C15H11.7	WBGene00003922	pas-1	1.32851	0.00975359	ENSP00000261479	ENSG00000100902	proteasome (prosome, macropain) subunit, alpha type, 6	PSMA6	-0.11	0.177	-0.11	0.148
R186.1	WBGene00011305		1.32974	0.00627074	ENSP00000332721	ENSG00000183597						
M05D6.5	WBGene00010878	-	1.33437479879276	0.003707137	-							
F47H4.9	WBGene00009840	fbxa-189	1.33637479879276	0.002465431	-							
F28A10.5	WBGene00017873	-	1.33737479879276	0.001441939	-							
T28A11.21	WBGene00020883	fbxa-64	1.34037479879276	0.001430322	-							
ZK829.1	WBGene00014093		1.34077	0.00695075	ENSP00000263278	ENSG00000087076	dehydrogenase/reductase (SDR family) member 10	DHRS10	0.0509	0.367	0.152	0.047
K01C8.7	WBGene00010459		1.342	0.00315661	ENSP00000297578	ENSG00000164933	Mitochondrial folate transporter/carrier	MFTC	0.0196	0.746	-0.0553	0.501
C04C3.2	WBGene00015412	lgc-9	1.34337479879276	0.00148144	-							
F56A3.1	WBGene00018908	-	1.34337479879276	0.009845798	-							
Y57A10A.29	WBGene00013269		1.34568	0.00332122	ENSP00000301761	ENSG00000167985						
T23E7.2d	WBGene00020732	-	1.34637479879276	0.005259417	-							
F42A10.4b	WBGene00001160	efk-1	1.35058	0.0052167	ENSP00000263026	ENSG00000103319	eukaryotic elongation factor-2 kinase	EEF2K	0.1	0.034	0.211	0.00168
C32E8.9	WBGene00016325		1.35426	0.00687964	ENSP00000357278	ENSG00000093144	enoyl Coenzyme A hydratase domain containing 1	ECHDC1	-0.09	0.0795	-0.252	0.00178
K07E12.2	WBGene00019501	-	1.35437479879276	0.005872065	-							
C34E11.3	WBGene00044079	tag-241	1.35937479879276	0.0008030707	-							
F42H10.3	WBGene00018367	-	1.35937479879276	0.003115608	-							
F27B3.5	WBGene00017847	-	1.36037479879276	0.001218242	-							
F52C6.10	WBGene00018667	bath-7	1.36037479879276	0.007462534	-							

C13G3.3a	WBGene00007554	pptr-2	1.36039	0.00578274	ENSP00000230402	ENSG00000112640	protein phosphatase 2, regulatory subunit B (B56), delta isoform	PPP2R5D	-0.292	1.52e-06	-0.279	0.00214
ZK973.1	WBGene00022830	-	1.36137479879276	0.003627126	-							
F44F4.11	WBGene00006530	tba-4	1.36137479879276	0.006496216	-							
C16C10.11	WBGene00007630		1.36407	0.00442124	ENSP00000266157	ENSG00000138869	chromosome 22 open reading frame 16	C22orf16	-0.333	2.44e-07	-0.0558	0.444
K12F2.2b	WBGene00006874	vab-8	1.36652	0.00697713	ENSP00000355475	ENSG00000162849						
C16C4.7	WBGene00015831	-	1.36937479879276	0.007753407	-							
C30B5.4	WBGene00016245		1.37388	0.00316432	ENSP00000257020	ENSG00000134597	RNA binding motif protein, X-linked 2	RBMX2	0.0681	0.0579	0.114	0.0539
ZC513.4	WBGene00006935	vrs-1	1.37637479879276	0.001302771	-							
W09G12.7	WBGene00021121	-	1.37737479879276	0.002291771	-							
F55C5.5	WBGene00010094	tsfm-1	1.37756	0.00683177	ENSP00000313877	ENSG00000123297	Ts translation elongation factor, mitochondrial	TSFM	-0.0652	0.21	-0.0196	0.75
B0310.1	WBGene00015137	-	1.38337479879276	0.002311723	-							
F35D2.1	WBGene00018039	-	1.38437479879276	0.001138466	-							
F14D2.11	WBGene00017459	-	1.39037479879276	0.008231348	-							
F43H9.4	WBGene00018400	-	1.39237479879276	0.001021615	-							
F08E10.2	WBGene00008574	srbc-61	1.39237479879276	0.002951596	-							
F08F8.4	WBGene00017269		1.3935	0.0037802	ENSP00000306999	ENSG00000171320	establishment of cohesion 1 homolog 2 (S. cerevisiae)	ESCO2	-0.0244	0.231	0.0555	0.193
F25H8.5d	WBGene00001113	dur-1	1.39437479879276	0.001145427	-							
ZC395.8	WBGene00022598	ztf-8	1.39937479879276	0.005688866	-							
B0495.2	WBGene00015203		1.40209	0.00513209	ENSP00000367900	ENSG00000008128		CDC2L2				
F15C11.2c	WBGene00008852		1.40454	0.00524948	ENSP00000365576	ENSG00000135018	ubiquilin 1	UBQLN1	-0.0764	0.119	-0.234	0.0143
Y43F8C.12	WBGene00003413	mrp-7	1.40537479879276	0.001840081	-							

C04H4.1	WBGene00007311	-	1.40537479879276	0.004188338	-							
DY3.1	WBGene00006574	tin-13	1.41435	0.00904698	ENSP00000215570	ENSG00000099800	translocase of inner mitochondrial membrane 13 homolog (yeast)	TIMM13	0.0133	0.822	-0.157	0.0416
C28A5.1	WBGene00007788	-	1.41737479879276	0.008589705	-							
F56H11.4	WBGene00001239	elo-1	1.42237479879276	0.003232546	-							
Y32H12A.2	WBGene00021311		1.42293	0.00456848	ENSP00000350787	ENSG00000100296	chromosome 22 open reading frame 19	C22orf19	0.0681	0.025	-0.159	0.00947
C14C10.4	WBGene00007587		1.42293	0.00456848	ENSP00000260665	ENSG00000138095	Leucine-rich PPR-motif containing	LRPPRC	-0.226	1.25e-05	-0.233	0.00964
F07H5.10	WBGene00008561	-	1.42337479879276	0.001981358	-							
T02G5.13a	WBGene00020169	mmaa-1	1.42539	0.00255567	ENSP00000368769	ENSG00000151611	Methylmalonic aciduria (cobalamin deficiency) type A	MMAA	-0.0261	0.5	0.0125	0.826
C35D10.7b	WBGene00016444	-	1.42637479879276	0.001593394	-							
F59H5.3	WBGene00019131	bath-12	1.42637479879276	0.00744196	-							
M60.5	WBGene00002234	kqt-2	1.42837479879276	0.006397387	-							
T09D3.7	WBGene00005184	srg-27	1.42937479879276	0.0002034908	-							
C01H6.9	WBGene00007258		1.43397	0.00422508	ENSP00000325290	ENSG00000177602	germ cell associated 2 (haspin)	GSG2	-0.00541	0.889	0.0631	0.314
C09E8.3	WBGene00015646	mlt-10	1.43837479879276	0.000884378	-							
T07C12.10	WBGene00011574	-	1.43837479879276	0.005711076	-							
C53D6.2	WBGene00006852	unc-129	1.44133	0.00496182	ENSP00000224605	ENSG00000107623	growth differentiation factor 10	GDF10	0.259	0.000316	0.0201	0.753
F44G4.3	WBGene00009713	-	1.44237479879276	0.003889554	-							
F47G6.3	WBGene00018580	-	1.44237479879276	0.005074201	-							
C23G10.6	WBGene00016013	-	1.44337479879276	0.001450566	-							
ZK1248.7	WBGene00022877	-	1.44337479879276	0.005496738	-							
K08H2.5	WBGene00010692	-	1.45037479879276	0.003671425	-							
C44B7.5	WBGene00016627	-	1.45237479879276	0.002942433	-							
C15H11.11	WBGene00007619	-	1.45537479879276	0.000828933	-							

F33H2.6	WBGene00009369		1.45727	0.0080208	ENSP00000318024	ENSG00000176623						
Y63D3A.2	WBGene00013403	fbxb-100	1.45837479879276	0.0003335718	-							
C45G3.3	WBGene00001590	gip-2	1.45849	0.00927878	ENSP00000342071	ENSG00000144182	lipoyltransferase 1	LIPT1	0.19	0.00313	0.102	0.286
Y48G1A.2	WBGene00021658	-	1.45937479879276	0.007867052	-							
K07B1.3	WBGene00006729	ucp-4	1.46217	0.00131882	ENSP00000360398	ENSG00000153291	solute carrier family 25, member 27	SLC25A27	-0.255	0.000183	-0.226	0.0209
ZK945.3	WBGene00014165	puf-12	1.46585	0.00290888	ENSP00000371463	ENSG00000080608	KIAA0020	KIAA0020	0.00863	0.742	-0.0632	0.143
C41C4.2	WBGene00005150	sre-2	1.46937479879276	0.001138365	-							
VC5.2	WBGene00020908	-	1.47237479879276	0.001841036	-							
B0414.6	WBGene00001600	glh-3	1.47237479879276	0.006376499	-							
Y54F10BM.10	WBGene00021864	-	1.47237479879276	0.00823392	-							
W05F2.4	WBGene00021036	-	1.47337479879276	0.005162016	-							
D1081.8	WBGene00008386		1.47566	0.0099777	ENSP00000360532	ENSG00000096401	CDC5 cell division cycle 5-like (S. pombe)	CDC5L	-0.0781	0.0633	-0.0556	0.279
F54F2.1	WBGene00018832	-	1.47637479879276	0.001115741	-							
C54E4.1	WBGene00016917	-	1.47637479879276	0.001438048	-							
Y38F2AL.5	WBGene00021417	nhr-236	1.47637479879276	0.001678939	-							
W07G1.2	WBGene00012329	sre-44	1.47837479879276	0.002067467	-							
Y4C6B.7	WBGene00021161	-	1.47837479879276	0.003501954	-							
F56C11.6b	WBGene00018958	-	1.47937479879276	0.005387191	-							
AH10.2	WBGene00007083	-	1.48237479879276	0.003037723	-							
Y102A5C.33	WBGene00013629	srz-95	1.48837479879276	0.001593394	-							
C02B4.3	WBGene00007259	-	1.48937479879276	0.0002385246	-							
C12D12.3	WBGene00015714	-	1.49137479879276	0.001678939	-							
C50E3.5	WBGene00016816	-	1.49137479879276	0.008749284	-							
Y40B10A.2	WBGene00021487		1.4916	0.00687189	ENSP00000361616	ENSG00000165644	catechol-O-methyltransferase domain containing 1	COMTD1	-0.039	0.306	0.0336	0.57
C02D5.3	WBGene00015337	gsto-2	1.49406	0.00583191	ENSP00000358727	ENSG00000148834	glutathione S-transferase omega 1	GSTO1	-0.178	0.0129	-0.00949	0.885

T28A8.6	WBGene00012112	-	1.49437479879276	0.005724044	-						
R12E2.3	WBGene00004464	rpn-8	1.50141	0.00366942	ENSP00000219313	ENSG00000103035	Proteasome (prosome, macropain) 26S subunit, non-ATPase, 7 (Mov34 homolog)	PSMD7	0.0201	0.458	0.0996	0.031
C09E7.8b	WBGene00015642	-	1.50237479879276	0.001636327	-						
F42C5.4	WBGene00018346	-	1.50537479879276	0.0008968756	-						
B0523.5	WBGene00001443	fli-1	1.50755	0.00572083	ENSP00000324573	ENSG00000177731	flightless I homolog (Drosophila)	FLII	0.118	0.00114	-0.141	0.0301
T04C9.4a	WBGene00003375	mlp-1	1.50755	0.00317934	ENSP00000310901	ENSG00000175183	cysteine and glycine-rich protein 2	CSRP2	1.33	1.52e-10	0.281	0.0224
F27D9.8	WBGene00017866	stn-2	1.51122	0.00380517	ENSP00000311837	ENSG00000172554	syntrophin, gamma 2	SNTG2	0.0417	0.194	0.0343	0.379
B0513.5	WBGene00007197		1.51122	0.00486097	ENSP00000334726	ENSG00000100033	proline dehydrogenase (oxidase) 1	PRODH	0.511	5.35e-05	0.537	0.000215
Y38F2AL.6	WBGene00021418	-	1.51137479879276	0.005349995	-						
F39H11.1	WBGene00009565		1.51368	0.00331655	ENSP00000283882	ENSG00000153774	craniofacial development protein 1	CFDP1	0.116	0.000537	0.0378	0.461
R04A9.5b	WBGene00019864	-	1.51437479879276	0.0006838345	-						
B0001.2	WBGene00007087	-	1.51437479879276	0.0007191506	-						
R107.2	WBGene00011298		1.51613	0.00732961	ENSP00000311984	ENSG00000213995	hypothetical protein FLJ10769	FLJ10769	0.0247	0.721	-0.169	0.0159
JC8.7	WBGene00010439		1.52226	0.000216641	ENSP00000261511	ENSG00000056050					
Y47H10A.4	WBGene00012960	-	1.52237479879276	0.0006941726	-						
F52C6.7	WBGene00018664	bath-11	1.52237479879276	0.007098263	-						
D2005.5	WBGene00008400	drh-3	1.52437479879276	0.0009704376	-						
Y43F8B.10	WBGene00012820	-	1.52537479879276	0.001514882	-						
K11D9.3	WBGene00010772	-	1.52937479879276	0.004582421	-						
ZK809.8	WBGene00014088	-	1.53237479879276	0.0003751174	-						

K06A5.6	WBGene00019433		1.5382	0.00440836	ENSP00000357873	ENSG00000196177	acyl-Coenzyme A dehydrogenase, short/branched chain	ACADSB	0.00949	0.688	-0.0222	0.459
C40C9.5a	WBGene00006412	nlg-1	1.5382	0.00183	ENSP00000370483	ENSG00000146938	neuroligin 4, X-linked	NLGN4X	-0.556	2.37e-07	0.0593	0.514
Y6D1A.1	WBGene00012395	-	1.53837479879276	0.005263134	-							
D1054.3	WBGene00008371		1.53943	0.00331472	ENSP00000367211	ENSG00000165416	SGT1, suppressor of G2 allele of SKP1 (S. cerevisiae)	SUGT1	0.136	0.00805	0.188	0.0206
C49H3.11	WBGene00004471	rps-2	1.53943	0.0091351	ENSP00000341885	ENSG00000140988	ribosomal protein S2	RPS2	0.0247	0.656	-0.0234	0.693
Y7A5A.3	WBGene00012409	-	1.54137479879276	0.008113579	-							
ZK792.2	WBGene00002130	inx-8	1.54237479879276	0.0009302254	-							
C06A8.4	WBGene00004823	skr-17	1.54237479879276	0.006113657	-							
F10G8.9a	WBGene00008667		1.54433	0.00897917	ENSP00000280701	ENSG00000151093						
T25G3.1	WBGene00012029		1.55046	0.00873775	ENSP00000296121	ENSG00000163807	KIAA1143	KIAA1143	0.26	2.24e-06	0.0696	0.376
F38E9.2	WBGene00003555	nas-39	1.55169	0.0042309	ENSP00000061240	ENSG00000038295	tolloid-like 1	TLL1	-0.0169	0.411	0.0167	0.53
F40G12.5	WBGene00009600	-	1.55437479879276	0.0006116005	-							
C35D10.7a	WBGene00016444	-	1.55537479879276	0.003628649	-							
T06C10.4	WBGene00001453	flp-10	1.55837479879276	0.0006827269	-							
Y57A10B.3	WBGene00013275	-	1.56337479879276	0.001123195	-							
W05F2.5	WBGene00021037	fbxa-203	1.56337479879276	0.00184638	-							
D2023.6	WBGene00008410		1.56763	0.00240158	ENSP00000238561	ENSG00000063761	aarF domain containing kinase 1	ADCK1	0.0425	0.172	-0.016	0.75
F11C1.5a	WBGene00008694		1.57009	0.0047416	ENSP00000368612	ENSG00000102763	KIAA0564 protein	KIAA0564	-0.0236	0.354	-0.00777	0.796

C23G10.7b	WBGene00016014		1.57009	0.00990872	ENSP00000374387	ENSG00000155275						
C35C5.5	WBGene00002975	lev-8	1.57037479879276	0.002361975	-							
F34D6.3	WBGene00006318	sup-9	1.57622	0.00350316	ENSP00000302166	ENSG00000169427	potassium channel, subfamily K, member 9	KCNK9	-0.0681	0.0639	-0.0883	0.0748
Y42G9A.6	WBGene00021535	wht-7	1.57867	0.00613614	ENSP00000343820	ENSG00000160179	ATP-binding cassette, sub-family G (WHITE), member 1	ABCG1	0.0149	0.879	-0.233	0.0223
W06H8.8f	WBGene00006436	ttn-1	1.58137479879276	0.007425734	-							
F54F7.6	WBGene00010066	-	1.58337479879276	0.002311723	-							
B0285.10	WBGene00000513	ckb-3	1.58437479879276	0.001431072	-							
K08E3.5e	WBGene00010665		1.59216	0.00366942	ENSP00000338703	ENSG00000169764	UDP-glucose pyrophosphorylase 2	UGP2	-0.0623	0.056	-0.123	0.0133
W03A3.2	WBGene00020964	polq-1	1.59216	0.00165612	ENSP00000264233	ENSG00000051341	polymerase (DNA directed), theta	POLQ	-0.0511	0.381	0.133	0.1
R11A5.3	WBGene00011231	-	1.59437479879276	0.0005563053	-							
T13H10.1	WBGene00002193	kin-5	1.59837479879276	0.001102175	-							
C17G10.4b	WBGene00000383	cdc-14	1.59952	0.00289144	ENSP00000364389	ENSG00000081377	CDC14 cell division cycle 14 homolog B (S. cerevisiae)	CDC14B	0.102	0.00331	0.0127	0.842
C47E8.4	WBGene00008143		1.59952	0.0033316	ENSP00000158526	ENSG00000071859	family with sequence similarity 50, member A	FAM50A	0.345	1.16e-08	-0.000189	0.998
C08B11.8	WBGene00007435		1.60687	0.00698875	ENSP00000263440	ENSG00000088035	asparagine-linked glycosylation 6 homolog (yeast, alpha-1,3-glucosyltransferase)	ALG6	-0.0488	0.284	-0.048	0.506
K08F4.3	WBGene00010678	-	1.60737479879276	0.007586633	-							
F28F8.4	WBGene00009222	fbxa-95	1.61037479879276	0.006900026	-							
C09D1.1a	WBGene00006820	unc-89	1.61437479879276	0.00443009	-							
Y57G11B.1	WBGene00013294	-	1.61937479879276	0.0004897584	-							

K11D12.11	WBGene00019650	-	1.62137479879276	0.001783467	-							
C05E4.13	WBGene00006288	str-262	1.62337479879276	0.001312293	-							
F55G1.4	WBGene00018900	rod-1	1.62404	0.00196435	ENSP00000 328236	ENSG000 00184445	kinetochore associated 1	KNTC1	0.0457	0.0777	0.00587	0.866
C40C9.5b	WBGene00006412	nlg-1	1.62404	0.00150003	ENSP00000 370483	ENSG000 00146938	neuroligin 4, X-linked	NLGN4X	-0.556	2.37e-07	0.0593	0.514
C16B8.1	WBGene00003007	lin-18	1.62637	0.00900118	ENSP00000 296084	ENSG000 00163785	Tyrosine-protein kinase RYK precursor	RYK	0.169	0.0000071	0.0913	0.043
D1086.5	WBGene00008392	-	1.62637479879276	0.0005738413	-							
F57B10.4	WBGene00019002	-	1.62737479879276	0.001752599	-							
C44B7.10	WBGene00016630	-	1.62737479879276	0.009576075	-							
C34C6.4	WBGene00007917		1.62895	0.00555324	ENSP00000 319851	ENSG000 00016391	choline dehydrogenase	CHDH	0.0888	0.0425	0.0506	0.36
T05E7.4	WBGene00020260	-	1.62937479879276	0.002025819	-							
C34B2.5	WBGene00016390		1.6314	0.00812637	ENSP00000 231238	ENSG000 00113312	tetratricopeptide repeat domain 1	TTC1	-0.0051	0.869	-0.111	0.0183
C44B7.4	WBGene00016626		1.6314	0.00141939	ENSP00000 357585	ENSG000 00164451						
B0414.7b	WBGene00003472	mtk-1	1.63508	0.0015874	ENSP00000 265125	ENSG000 00085511	Mitogen-activated protein kinase kinase kinase 4	MAP3K4	0.0222	0.299	0.041	0.199
T23G7.3	WBGene00011966		1.63508	0.00313104	ENSP00000 318966	ENSG000 00104637	PIN2-interacting protein 1	PINX1	0.0235	0.515	-0.0396	0.328
F53C11.1	WBGene00009971	-	1.63637479879276	0.002636016	-							
K06A5.1	WBGene00019429	-	1.63937479879276	0.001773054	-							
F11E6.1c	WBGene00008706		1.63998	0.00132233	ENSP00000 314508	ENSG000 00177628	group-specific component (vitamin D binding protein)	GC	0.00555	0.89	0.0344	0.487
C54D1.3	WBGene00002163	ist-1	1.64037479879276	0.0005197241	-							
Y95B8A.11	WBGene00022390	-	1.64337479879276	0.008172892	-							
T21B10.4	WBGene00011886	-	1.64637479879276	0.005611082	-							

D2023.2	WBGene00004258	pyc-1	1.64857	0.00496182	ENSP00000347900	ENSG00000173599	pyruvate carboxylase	PC	0.077	0.0655	0.099	0.0998
F08G5.3a	WBGene00008582	-	1.64937479879276	0.0007983517	-							
T13B5.3	WBGene00020471	-	1.65037479879276	0.001802702	-							
F22E12.1	WBGene00009058	-	1.65337479879276	0.0007706149	-							
C14C6.12	WBGene00015763	-	1.65637479879276	0.0007764237	-							
E03A3.4	WBGene00001944	his-70	1.65737479879276	0.0002105056	-							
F54C9.9	WBGene00010044		1.6596	0.000971292	ENSP00000320917	ENSG00000129347	KRI1 homolog (S. cerevisiae)	KRI1				
C27H5.2a	WBGene00016172	-	1.66037479879276	0.001122871	-							
C49C8.2	WBGene00016766	-	1.66037479879276	0.002338111	-							
K06G5.1	WBGene00010605	-	1.66237479879276	0.0004310377	-							
T22C1.1	WBGene00011912		1.66328	0.00124657	ENSP00000013070	ENSG00000012963	chromosome 14 open reading frame 130	C14orf130	-0.333	2.38e-05	-0.00531	0.915
C47A4.3	WBGene00008124	-	1.66337479879276	0.002894117	-							
M03C11.7	WBGene00010844		1.66696	0.00246802	ENSP00000315379	ENSG00000117360	PRP3 pre-mRNA processing factor 3 homolog (yeast)	PRPF3	0.301	1.89e-06	0.0991	0.109
B0304.4	WBGene00015136	-	1.67337479879276	0.005658323	-							
C28C12.12	WBGene00016182	-	1.67337479879276	0.007375039	-							
F19H8.1	WBGene00006603	tps-2	1.67937479879276	0.002154151	-							
T05E11.2	WBGene00011479	-	1.68637479879276	0.002853657	-							
F38A5.9	WBGene00018166	nspb-5	1.68837479879276	0.0003499355	-							
C09D4.2	WBGene00015633	-	1.69037479879276	0.001201104	-							
C02F12.1	WBGene00006643	tsp-17	1.69237479879276	0.004022692	-							
C01B4.10	WBGene00006136	str-76	1.69337479879276	0.001868702	-							
C10G8.7	WBGene00000454	ceh-33	1.69516	0.00777261	ENSP00000247182	ENSG00000126778	sine oculis homeobox homolog 1 (Drosophila)	SIX1	0.0102	0.835	0.0862	0.113

F57B10.6	WBGene00019004	xpg-1	1.69639	0.00146016	ENSP00000347978	ENSG00000134899	excision repair cross-complementing rodent repair deficiency, complementation group 5 (xeroderma pigmentosum, complementation group G (Cockayne syndrome))	ERCC5	0.177	0.000161	0.142	0.0502
C16C8.11	WBGene00015849	-	1.70037479879276	0.008299555	-							
F49D11.5	-	-	1.70237479879276	0.0006624761	-							
K05F1.9	WBGene00019410	-	1.70237479879276	0.001138466	-							
F55C5.2	WBGene00010092	-	1.70337479879276	0.005717529	-							
K08F4.7	WBGene00001752	gst-4	1.70537479879276	0.004666535	-							
F44F4.2	WBGene00009701	-	1.70537479879276	0.008158383	-							
C16C8.4	WBGene00015842	-	1.70637479879276	0.004538463	-							
Y59A8B.11	WBGene00013348	fbxa-106	1.70837479879276	0.003502757	-							
C33F10.5b	WBGene00016354	rig-6	1.70837479879276	0.003920001	-							
W01A11.2	WBGene00020910		1.70988	0.00686768	ENSP00000198801	ENSG00000166391	monoacylglycerol O-acyltransferase 2	MOGAT2	-0.0223	0.544	0.0856	0.149
D2024.7	WBGene00017054	-	1.71237479879276	0.0004194459	-							
C04F12.3	WBGene00002069	ikb-1	1.71237479879276	0.006113657	-							
W06H8.8c	WBGene00006436	ttn-1	1.71337479879276	0.0003662245	-							
C34E10.10	WBGene00016411		1.71356	0.00316836	ENSP00000308332	ENSG00000147679						
F10C2.6	WBGene00001095	drs-2	1.71601	0.00961737	ENSP00000355086	ENSG00000117593						
T09A5.9	WBGene00011637		1.71724	0.00479086	ENSP00000234038	ENSG00000115685	protein phosphatase 1, regulatory subunit 7	PPP1R7	-0.216	4.11e-07	-0.0322	0.381
C01C7.1	WBGene00000186	ark-1	1.71937479879276	0.006718562	-							
K10F12.3a	WBGene00004045	pll-1	1.7246	0.00468866	ENSP00000341052	ENSG00000115896	phospholipase C-like 1	PLCL1	0.136	0.163	-0.162	0.176

F38A5.5	WBGene00018162	nspb-3	1.73337479879276	0.0005680517	-							
F23D12.5	WBGene00009089	-	1.73337479879276	0.0009910165	-							
T23E1.2	WBGene00020729	-	1.74137479879276	0.002067467	-							
C14H10.4	WBGene00006135	str-74	1.74237479879276	0.002897135	-							
Y45F10D.13	WBGene00012891	tag-208	1.74544	0.00137296	ENSP00000360293	ENSG00000095637	sorbin and SH3 domain containing 1	SORBS1	0.165	0.0233	0.0935	0.092
T20F5.3	WBGene00020625		1.74789	0.00146193	ENSP00000343867	ENSG00000148187	mitochondrial ribosome recycling factor	MRRF	0.0815	0.00322	-0.0416	0.214
T04H1.4	WBGene00004296	rad-50	1.75157	0.00200327	ENSP00000368100	ENSG00000113522	RAD50 homolog (S. cerevisiae)	RAD50	0.0937	0.0497	-0.114	0.225
Y57G11C.6	WBGene00013304	-	1.75437479879276	0.000001099089	-							
C50F2.4	WBGene00016838	-	1.75437479879276	0.0008574562	-							
C03B1.4	WBGene00015375	-	1.75437479879276	0.004606642	-							
K04H4.5	WBGene00010574	-	1.75437479879276	0.00497475	-							
F46G10.5	WBGene00004238	ptr-24	1.76137479879276	0.001269682	-							
Y40H7A.8	WBGene00005208	srg-51	1.76137479879276	0.007355692	-							
E01G4.5	WBGene00008447	-	1.76237479879276	0.002821526	-							
F43G6.7	WBGene00009659	-	1.76437479879276	0.0010786	-							
C01B4.8	WBGene00015272	-	1.76537479879276	0.0006033311	-							
K03E6.3	WBGene00003565	ncs-3	1.76837479879276	0.0002854226	-							
C30C11.2	WBGene00004460	rpn-3	1.76874	0.00309039	ENSP00000264639	ENSG00000108344	proteasome (prosome, macropain) 26S subunit, non-ATPase, 3	PSMD3	-0.2	0.000104	-0.2	0.00133
C32E8.10c	WBGene00006751	unc-11	1.76874	0.000596108	ENSP00000342095	ENSG00000073921	Phosphatidylinositol binding clathrin assembly protein	PICALM	0.094	0.0637	-0.0339	0.674
T07C5.1c	WBGene00011564	ugt-50	1.77237479879276	0.006993496	-							
ZC168.3	WBGene00013857		1.77242	0.00726553	ENSP00000297431	ENSG00000164815	origin recognition complex, subunit 5-like (yeast)	ORC5L	-0.109	9.96e-05	-0.167	0.00487
Y11D7A.10	WBGene00012434	-	1.77437479879276	0.00382214	-							
ZC247.1	WBGene00013859	-	1.77737479879276	0.002035984	-							
Y57G7A.11	WBGene00021974	-	1.78837479879276	0.00745894	-							

ZK1236.7	WBGene00022865		1.78959	0.00102421	ENSP00000369561	ENSG00000198171	chromosome 20 open reading frame 116	C20orf116	0.0107	0.77	-0.116	0.113
R12H7.2	WBGene00000217	asp-4	1.79137479879276	0.009932945	-							
T07H6.5	WBGene00020332		1.79327	0.00100264	ENSP00000297405	ENSG00000164796	CUB and Sushi multiple domains 3	CSMD3	-0.107	0.0403	0.138	0.0307
C05G5.5	WBGene00007351		1.79817	0.0018707	ENSP00000335004	ENSG00000186854						
ZC410.3	WBGene00013881	-	1.80137479879276	0.0003335718	-							
T24H7.5b	WBGene00020784	tat-4	1.80237479879276	0.002976113	-							
F54B11.9	WBGene00010032	-	1.80537479879276	0.0003002532	-							
ZK1058.5	WBGene00014205		1.80553	0.00901287	ENSP00000373300	ENSG00000206562						
F35G12.1a	WBGene00009439		1.80676	0.00548286	ENSP00000262430	ENSG00000103150	malonyl-CoA decarboxylase	MLYCD	0.162	0.000126	-0.049	0.357
Y32F6A.5	WBGene00012530		1.80921	0.00610103	ENSP00000262288	ENSG00000121064	serine carboxypeptidase 1	SCPEP1	0.362	4.39e-07	-0.0054	0.945
ZC8.3	WBGene00022499	set-30	1.81237479879276	0.0002221317	-							
B0334.10	WBGene00007148	-	1.81537479879276	0.001257213	-							
F52B5.3	WBGene00009922		1.82515	0.000781077	ENSP00000161863	ENSG00000047188	YTH domain containing 2	YTHDC2	-0.0931	0.0978	-0.174	0.0849
T03G11.1	WBGene00004145	pqn-62	1.82837479879276	0.00007294438	-							
F44G4.6	WBGene00009715	-	1.83037479879276	0.0002772778	-							
Y18D10A.8	WBGene00012478	-	1.83537479879276	0.001173715	-							
D1081.2	WBGene00006844	unc-120	1.83837479879276	0.003114095	-							
ZC155.4	WBGene00022532	-	1.84037479879276	0.008668793	-							
C23G10.5	WBGene00016012	-	1.84337479879276	0.0003040117	-							
F55A12.10	WBGene00018867	-	1.84337479879276	0.007819323	-							
C30C11.1	WBGene00016249		1.846	0.00245745	ENSP00000223324	ENSG00000106591	mitochondrial ribosomal protein L32	MRPL32	-0.286	1.07e-05	-0.00546	0.939
F23B12.9	WBGene00001170	egl-1	1.84737479879276	0.0003143407	-							
F20D12.1	WBGene00017641	csr-1	1.84737479879276	0.001857386	-							
R31.2	WBGene00011270	-	1.84837479879276	0.00234931	-							

D2013.8	WBGene00004744	scp-1	1.8509	0.000678418	ENSP00000265565	ENSG00000114650	SREBP cleavage-activating protein	SCAP	-0.0257	0.528	-0.113	0.072
K08F4.1	WBGene00010676		1.8509	0.00254772	ENSP00000313029	ENSG00000127586	CTF18, chromosome transmission fidelity factor 18 homolog (S. cerevisiae)	CHTF18	-0.0112	0.746	0.00184	0.965
T07H6.2	WBGene00003394	mom-1	1.85826	0.00718384	ENSP00000322304	ENSG00000102312	porcupine homolog (Drosophila)	PORCN	-0.124	0.000353	-0.15	0.0101
F21C10.4	WBGene00017654	-	1.85837479879276	0.0007476318	-							
C06A5.6	WBGene00015504	-	1.86037479879276	0.003316807	-							
C29A12.3a	WBGene00002985	lig-1	1.86194	0.00593462	ENSP00000263274	ENSG00000105486	ligase I, DNA, ATP-dependent	LIG1	0.0919	0.00929	0.0467	0.296
C14H10.2	WBGene00007592	-	1.86337479879276	0.0005170232	-							
Y102A5C.1	WBGene00013610	fbxa-206	1.86337479879276	0.003040185	-							
H14A12.4	WBGene00003376	mls-1	1.86684	0.00448523	ENSP00000331791	ENSG00000184058	T-box 1	TBX1	-0.0226	0.437	-0.0182	0.667
C15H11.5	WBGene00007615	set-31	1.87437479879276	0.002256334	-							
F26H11.1	WBGene00009179	kbp-3	1.87437479879276	0.004586793	-							
R74.2	WBGene00011278	-	1.87937479879276	0.002470601	-							
M01A10.1	WBGene00019692	-	1.88337479879276	0.004779416	-							
F15D4.5	WBGene00008862	-	1.88737479879276	0.0001056284	-							
W03F8.6	WBGene00020995	-	1.89137479879276	0.0002045415	-							
T24B8.2	WBGene00011976		1.89505	0.00349083	ENSP00000324491	ENSG00000147457	CHMP family, member 7	CHMP7	0.0192	0.6	-0.0142	0.823
F52G2.3	WBGene00009943	-	1.89637479879276	0.0002115202	-							
T09B4.9	WBGene00020383		1.90363	0.000886374	ENSP00000270538	ENSG00000104980	translocase of inner mitochondrial membrane 44 homolog (yeast)	TIMM44	0.0383	0.289	-0.109	0.0237
C02F12.4	WBGene00006431	tag-52	1.90437479879276	0.00004730358	-							
C53D6.3	WBGene00008280	acc-2	1.90437479879276	0.000488793	-							

F43E2.5	WBGene00018393		1.90731	0.00761159	ENSP00000371930	ENSG00000175806	Methionine sulfoxide reductase A	MSRA	-0.0433	0.0279	-0.0128	0.681
Y113G7B.7	WBGene00013758	fbxa-114	1.90737479879276	0.004871252	-							
C38D9.2	WBGene00008010	-	1.91237479879276	0.0001213554	-							
Y55F3AM.15	WBGene00000816	csn-4	1.91344	0.00346971	ENSP00000264389	ENSG00000138663	COP9 constitutive photomorphogenic homolog subunit 4 (Arabidopsis)	COPS4	-0.0234	0.252	-0.0241	0.421
Y57G11A.1b	WBGene00013289	tag-273	1.91437479879276	0.0001107282	-							
F46H5.7a	WBGene00018522	-	1.92037479879276	0.009473069	-							
F55A11.8	WBGene00010081	-	1.92237479879276	0.007442966	-							
R05D3.6	WBGene00019880	-	1.92337479879276	0.001132156	-							
C07D8.6	WBGene00015565	-	1.92737479879276	0.007023256	-							
F29C12.5	WBGene00009247	bath-45	1.93237479879276	0.003774393	-							
T28B4.3	WBGene00020886	-	1.93637479879276	0.000133633	-							
T28D9.3	WBGene00020895	-	1.93637479879276	0.003399057	-							
B0047.3	WBGene00015014	bath-24	1.94237479879276	0.009941195	-							
F29F11.4	WBGene00006667	twk-12	1.94537479879276	0.0006147888	-							
T10G3.1	WBGene00011692	-	1.94937479879276	0.0001319176	-							
C32F10.2	WBGene00003020	lin-35	1.95146	0.000936269	ENSP00000262133	ENSG00000103479	retinoblastoma-like 2 (p130)	RBL2	0.0771	0.0727	0.0783	0.0639
C23H3.5	WBGene00016021	-	1.95637479879276	0.0001256696	-							
C07H4.1	WBGene00007421	-	1.95837479879276	0.002256334	-							
ZK54.2	WBGene00006602	tps-1	1.95837479879276	0.002493727	-							
F08C6.7	WBGene00006827	unc-98	1.96237479879276	0.0005371493	-							
B0496.3a	WBGene00006814	unc-82	1.96537479879276	0.0001018239	-							
F47A4.3	WBGene00009800	rrc-1	1.96617	0.00157211	ENSP00000007510	ENSG00000004777	sorting nexin 26	SNX26	-0.193	0.000703	-0.022	0.733
F26A1.6	WBGene00017804	-	1.96837479879276	0.001123195	-							
F09E8.7	WBGene00002974	lev-1	1.97037479879276	0.003163613	-							
T23H4.2	WBGene00003659	nhr-69	1.97937479879276	0.005681665	-							
W05E10.4	WBGene00006609	tre-3	1.98237479879276	0.0004483314	-							
Y57A10A.10	WBGene00013255		1.98334	0.00111757	ENSP00000363824	ENSG00000095209	transmembrane protein 38B	TMEM38B	0.127	0.00517	0.104	0.24
F31D5.3d	WBGene00006495	tag-149	1.98937479879276	0.000501023	-							
W06H8.2	WBGene00021066	-	1.99237479879276	0.0003887195	-							

ZC247.2	WBGene00013860	-	1.99237479879276	0.001138466	-								
T16A9.4	WBGene00011794	-	1.99537479879276	0.000710121	-								
F59A1.9	WBGene00010295	fbxa-193	1.99537479879276	0.001052057	-								
Y39B6A.1	WBGene00012664		1.99805	0.00308673	ENSP00000357791	ENSG00000197915							
F54C8.2	WBGene00010036	cpar-1	2.00137479879276	0.001686449	-								
H12C20.6	WBGene00003691	nhr-101	2.00337479879276	0.001490761	-								
Y66H1B.3	WBGene00022049	-	2.01237479879276	0.0001568459	-								
C14C10.3	WBGene00007586	ril-2	2.01437479879276	0.00528428	-								
K09C4.8	WBGene00006308	sul-1	2.01522	0.00262086	ENSP00000260128	ENSG00000137573	sulfatase 1	SULF1	0.172	0.0127	-0.0144	0.796	
C01G10.7	WBGene00007234		2.01645	0.0029892	ENSP00000365538	ENSG00000125246	citrate lyase beta like	CLYBL	-0.025	0.653	-0.118	0.0549	
C16C8.5	WBGene00015843	-	2.01837479879276	0.007206327	-								
ZK829.4	WBGene00014095		2.02871	0.0081599	ENSP00000277865	ENSG00000148672	glutamate dehydrogenase 1	GLUD1	0.00609	0.818	-0.0388	0.329	
C13C12.1	WBGene00000285	cal-1	2.03137479879276	0.00004358117	-								
T22B2.4	WBGene00006321	sup-12	2.03607	0.00562376	ENSP00000368341	ENSG00000112183	RNA binding motif protein 24	RBM24	-0.34	4.48e-07	0.0677	0.483	
M02D8.6	WBGene00019732	-	2.03837479879276	0.001238606	-								
Y7A5A.5	WBGene00012411	-	2.04137479879276	0.002067043	-								
Y110A7A.13	WBGene00000502	chp-1	2.0422	0.00153816	ENSP00000319255	ENSG00000110172	cysteine and histidine-rich domain (CHORD)-containing, zinc binding protein 1	CHORDC1	0.183	0.181	0.254	0.189	
C18H7.2b	WBGene00002140	inx-18	2.04237479879276	0.0009302254	-								
W02B8.1	WBGene00012197	-	2.04537479879276	0.003728648	-								
F45H10.2	WBGene00009739	-	2.04537479879276	0.009253549	-								
D1086.8	WBGene00008395	-	2.04737479879276	0.001311196	-								
C16C8.13	WBGene00015851	-	2.04737479879276	0.004805353	-								

C56C10.9	WBGene00016965		2.05078	0.00309039	ENSP00000353094	ENSG00000078808	calcium binding protein Cab45 precursor /// calcium binding protein Cab45 precursor	Cab45	0.0848	0.0374	0.0474	0.327
M02D8.1	WBGene00019727	-	2.05137479879276	0.0001213554	-							
C18A11.7b	WBGene00001000	dim-1	2.05137479879276	0.001324475	-							
Y110A2AL.12a	WBGene00022447		2.05446	0.00022409	ENSP00000332313	ENSG00000184886						
F31D5.3a	WBGene00006495	tag-149	2.05837479879276	0.0005234086	-							
W09C3.6	WBGene00021113	gsp-3	2.06237479879276	0.0005197241	-							
Y51A2D.10	WBGene00013078	-	2.06537479879276	0.0006095968	-							
Y38F2AR.11a	-	-	2.06537479879276	0.007098263	-							
C32A3.1a	WBGene00004765	sel-8	2.06837479879276	0.002419029	-							
M05B5.4	WBGene00010872		2.0704	0.00127056	ENSP00000219345	ENSG00000103066	lysophospholipase 3 (lysosomal phospholipase A2)	LYPLA3	0.0256	0.519	0.0449	0.496
Y39A3B.5	WBGene00021439	ckr-2	2.07531	0.00145089	ENSP00000295589	ENSG00000163394	cholecystokinin A receptor	CCKAR	-0.0167	0.717	0.0359	0.559
F57B10.12	WBGene00003184	mei-2	2.07537479879276	0.00632539	-							
F59A1.8	WBGene00010294	fbxa-129	2.07737479879276	0.001431072	-							
B0563.2	WBGene00006637	tsp-11	2.08237479879276	0.002256334	-							
R107.7	WBGene00001749	gst-1	2.08267	0.00483838	ENSP00000196968	ENSG00000084207	glutathione S-transferase pi	GSTP1	0.22	5.03e-05	0.096	0.186
ZK546.17	WBGene00022766	cblc-1	2.0888	0.00130994	ENSP00000361154	ENSG00000132763						
W06D12.2	WBGene00006692	twk-42	2.09437479879276	0.0001283356	-							
ZC64.2	WBGene00022515	-	2.09537479879276	0.001752599	-							
F37B1.8	WBGene00001767	gst-19	2.10037479879276	0.003522872	-							
M162.8	WBGene00010934	fbxa-118	2.10337479879276	0.003182772	-							
F42D1.2	WBGene00009628		2.10474	0.00329612	ENSP00000348234	ENSG00000198650	tyrosine aminotransferase	TAT	0.0162	0.513	-0.0265	0.534

T22C1.6	WBGene00011917		2.10474	0.00141939	ENSP00000 362712	ENSG000 00084652						
C54F6.5	WBGene00016923	-	2.11037479879276	0.002423504	-							
Y57A10A.18	WBGene00004167	pqn-87	2.11437479879276	0.001735694	-							
R05F9.6	WBGene00019890		2.11945	0.000906595	ENSP00000 342316	ENSG000 00079739	phosphoglucom utase 1	PGM1	0.283	0.000139	0.176	0.0123
F52H3.1	WBGene00002497	let-268	2.11945	0.000354782	ENSP00000 223127	ENSG000 00106397	procollagen-lysine, 2-oxoglutarate 5-dioxygenase 3	PLOD3	0.259	3.31e-05	0.0384	0.707
C52E4.4	WBGene00004501	rpt-1	2.12559	0.00489246	ENSP00000 292644	ENSG000 00161057	proteasome (prosome, macropain) 26S subunit, ATPase, 2	PSMC2	-0.112	0.0353	-0.0917	0.278
F58G11.3	WBGene00010281	-	2.12737479879276	0.001520771	-							
C16A11.6	WBGene00015819	-	2.13337479879276	0.002571161	-							
B0024.9	WBGene00007099	trx-2	2.13907	0.00628724	ENSP00000 216185	ENSG000 00100348	thioredoxin 2	TXN2	0.0314	0.299	-0.0783	0.144
M03B6.5	WBGene00010837	-	2.14437479879276	0.001611863	-							
F14D2.15	WBGene00023427	-	2.15937479879276	0.004706151	-							
K07A3.1	WBGene00001404	fbp-1	2.15992	0.00296901	ENSP00000 364475	ENSG000 00165140	fructose-1,6-bisphosphatase 1	FBP1	0.0115	0.786	-0.0206	0.701
F42C5.5	WBGene00018347	-	2.16437479879276	0.001953025	-							
F59G1.8	WBGene00019129	-	2.17337479879276	0.001404923	-							
R07E3.5	WBGene00011106	acl-5	2.17341	0.0037704	ENSP00000 289657	ENSG000 00158669	putative lysophosphatidi c acid acyltransferase	DKFZp586M1 819	0.214	4.56e-06	-0.028	0.618
F35G2.4	WBGene00004025	phy-2	2.17709	0.000602468	ENSP00000 307318	ENSG000 00122884	procollagen-proline, 2-oxoglutarate 4-dioxygenase (proline 4-hydroxylase), alpha polypeptide I	P4HA1	0.285	0.00145	0.196	0.101
F21A10.1	WBGene00008998	-	2.17937479879276	0.0004261326	-							

D2005.3	WBGene00008398		2.17954	0.00426689	ENSP00000221784	ENSG00000105185	Programmed cell death 5	PDCD5	0.024	0.683	0.0474	0.535
T07H6.1b	WBGene00020330	-	2.18437479879276	0.002897135	-							
K07C11.1	WBGene00003937	pax-1	2.18445	0.00861122	ENSP00000355245	ENSG00000198807	paired box gene 9	PAX9	0.0197	0.538	-0.0459	0.277
F57B10.11	WBGene00000236	bag-1	2.18935	0.000385772	ENSP00000224112	ENSG00000107262	BCL2-associated athanogene /// BCL2-associated athanogene	BAG1	0.0175	0.698	-0.0818	0.144
F47G6.2	WBGene00018579	-	2.18937479879276	0.00005803791	-							
M05D6.6	WBGene00010879	-	2.19037479879276	0.0007191506	-							
F14D2.1	WBGene00017450	bath-27	2.19637479879276	0.004702763	-							
T04H1.9	WBGene00006539	tbb-6	2.19737479879276	0.0006970733	-							
C27B7.4	WBGene00007761	rad-26	2.2102	0.000261655	ENSP00000296477	ENSG00000164080						
F08F3.10	WBGene00017266	-	2.21037479879276	0.001449004	-							
VC27A7L.1	WBGene00012147	-	2.21937479879276	0.0006865604	-							
T03G6.3	WBGene00020195	-	2.22337479879276	0.0002484798	-							
T03F1.5	WBGene00020187	gsp-4	2.22837479879276	0.0004300189	-							
B0035.4	WBGene00007107	pfd-4	2.23227	0.00917052	ENSP00000360473	ENSG00000101132	prefoldin 4	PFDN4	-0.0972	0.00213	-0.0369	0.393
M01G5.3	WBGene00019716		2.23227	0.00498756	ENSP00000345152	ENSG00000188735						
T04B8.1	WBGene00020203	-	2.23337479879276	0.002853913	-							
T22H2.4	WBGene00011934	-	2.24237479879276	0.0003867291	-							
T09B4.8	WBGene00020382	-	2.24837479879276	0.0000454845	-							
C13C4.7	WBGene00007551	-	2.24837479879276	0.001438048	-							
Y35H6.1	WBGene00021341	-	2.25137479879276	0.0001399958	-							
F36F2.2	WBGene00009476	-	2.25137479879276	0.0007609562	-							
T07C12.9	WBGene00011573	-	2.25537479879276	0.0003592767	-							
F22A3.6a	WBGene00017691	ilys-5	2.26637479879276	0.00003195483	-							
K08F8.4	WBGene00000240	pah-1	2.27029	0.000835491	ENSP00000303500	ENSG00000171759	phenylalanine hydroxylase	PAH	-0.00828	0.759	0.025	0.517
ZK892.5	WBGene00014129	-	2.27137479879276	0.001073401	-							

R04B5.6	WBGene00011004		2.27396	0.00953743	ENSP00000267814	ENSG00000140263	sorbitol dehydrogenase	SORD	0.112	0.00109	-0.113	0.0139
T28B4.1a	WBGene00020884	-	2.27537479879276	0.001936615	-							
C37E2.1	WBGene00007993	-	2.27637479879276	0.006804982	-							
B0513.4	WBGene00007196	-	2.28137479879276	0.00003235633	-							
C15C7.4	WBGene00015790	-	2.29437479879276	0.0001337124	-							
F42A10.8	WBGene00003546	nas-28	2.29737479879276	0.001484514	-							
F53G2.4b	WBGene00004129	pqn-42	2.29737479879276	0.00596408	-							
T03F7.1	WBGene00004910	snf-11	2.29849	0.000515092	ENSP00000287766	ENSG00000157103	solute carrier family 6 (neurotransmitter transporter, GABA), member 1	SLC6A1	-0.13	0.0485	-0.0028	0.977
ZK945.9	WBGene00003058	lov-1	2.30237479879276	0.0006116005	-							
C04E6.7	WBGene00015423	-	2.30237479879276	0.0008773175	-							
T08G11.5	WBGene00006765	unc-29	2.31237479879276	0.0007500735	-							
F20D6.1	WBGene00017633	-	2.31437479879276	0.0002290809	-							
C16C8.14	WBGene00015852	-	2.31537479879276	0.002328445	-							
Y82E9BR.6	WBGene00022339	-	2.31837479879276	0.00004519628	-							
H12D21.9	WBGene00010385	-	2.31837479879276	0.0006033311	-							
C01B4.7	WBGene00015271	-	2.32437479879276	0.0003040117	-							
K03E6.6	WBGene00003991	pfn-3	2.32537479879276	0.00005803791	-							
F19C6.4	WBGene00008953	-	2.32537479879276	0.000166224	-							
W05B10.1	WBGene00012276	his-74	2.33037479879276	0.009077459	-							
C28G1.6	WBGene00016191	-	2.33537479879276	0.00002011985	-							
F36D3.3	WBGene00005654	srr-3	2.33737479879276	0.001438048	-							
F02H6.5	WBGene00008538		2.33773	0.00123627	ENSP00000260324	ENSG00000137767	sulfide quinone reductase-like (yeast)	SQRDL	0.364	2.97e-06	0.135	0.0668
Y69F12A.2a	WBGene00000118	alh-12	2.34037479879276	0.00007276364	-							
F13D11.3	WBGene00017428	-	2.34237479879276	0.00008062163	-							
H14N18.1b	WBGene00006760	unc-23	2.34509	8.52e-05	ENSP00000359727	ENSG00000112208	BCL2-associated athanogene 2	BAG2	0.0927	0.0227	-0.105	0.102
F40F4.2	WBGene00002254	lbp-2	2.35137479879276	0.0001223028	-							
ZK593.2	WBGene00014002	-	2.35337479879276	0.0004693717	-							
ZC410.4a	WBGene00006663	twk-8	2.35437479879276	0.000008406864	-							
ZK1248.16	WBGene00002268	lec-5	2.35537479879276	0.003118251	-							
T27A1.4	WBGene00020836	-	2.36337479879276	0.0005170232	-							

W02B3.4	WBGene00020924		2.36593	0.000487857	ENSP00000223528	ENSG00000106692	Fukuyama type congenital muscular dystrophy (fukutin)	FCMD	-0.0711	0.0385	0.0602	0.39
C40H1.5	WBGene00008040	-	2.37337479879276	0.0001699732	-							
K07C11.5	WBGene00019478	tag-225	2.38188	0.00400512	ENSP00000262768	ENSG00000035862	tissue inhibitor of metalloproteinase 2	TIMP2	0.35	0.00146	-0.238	0.137
B0273.1	WBGene00015097	-	2.38737479879276	0.001663075	-							
F37C12.12	WBGene00003176	mec-14	2.38837479879276	0.000804177	-							
B0285.7	WBGene00007139	-	2.39037479879276	0.0000238777	-							
F48F5.6	WBGene00009848	-	2.39737479879276	0.0009431409	-							
C52D10.6	WBGene00004818	skr-12	2.40737479879276	0.00008558027	-							
C28G1.3	WBGene00016188	sec-15	2.41131	0.000508218	ENSP00000260762	ENSG00000138190	SEC15-like 1 (S. cerevisiae)	SEC15L1	-0.0173	0.334	0.00658	0.837
F23F12.9a	WBGene00017755	-	2.41437479879276	0.0003758724	-							
K08C7.7	WBGene00010649	-	2.41937479879276	0.0006116005	-							
T14G12.3	WBGene00006408	tag-18	2.42737479879276	0.003171606	-							
ZC317.2	WBGene00022588	-	2.42937479879276	0.0003662245	-							
F57A8.4	WBGene00010179	-	2.42937479879276	0.000616257	-							
F42A10.4a	WBGene00001160	efk-1	2.43093	0.00194774	ENSP00000263026	ENSG00000103319	eukaryotic elongation factor-2 kinase	EEF2K	0.1	0.034	0.211	0.00168
C14F11.1a	WBGene00015778		2.43583	0.00153816	ENSP00000245206	ENSG00000125166	glutamic-oxaloacetic transaminase 2, mitochondrial (aspartate aminotransferase 2)	GOT2	-0.16	0.00076	-0.171	0.0124
T05B9.1	WBGene00011464	-	2.43737479879276	0.003278953	-							
C14F5.3c	WBGene00006588	tnt-3	2.44687	0.00120243	ENSP00000356286	ENSG00000118194	troponin T2, cardiac	TNNT2	-0.0381	0.262	0.00515	0.938
ZK228.3	WBGene00013946	-	2.45537479879276	0.001142535	-							

F46C3.1	WBGene00003970	pek-1	2.46649	0.000605648	ENSP00000307235	ENSG00000172071	Eukaryotic translation initiation factor 2-alpha kinase 3	EIF2AK3	0.036	0.145	0.0367	0.392
C10F3.5	WBGene00003954	pcm-1	2.47017	0.00242666	ENSP00000356354	ENSG00000120265	protein-L-isoaspartate (D-aspartate) O-methyltransferase	PCMT1	-0.588	8.65e-06	-0.661	0.000831
F53F10.1	WBGene00018763		2.47262	8.13e-05	ENSP00000353377	ENSG00000150636	chromosome 18 open reading frame 14	C18orf14	0.0228	0.218	0.0415	0.144
D2092.4	WBGene00017065	-	2.47337479879276	0.0000316547	-							
T27D1.1	WBGene00000885	cyn-9	2.47537479879276	0.0008692252	-							
F15G9.4b	WBGene00001863	him-4	2.4763	0.000279684	ENSP00000356462	ENSG00000143341						
M03A8.4	WBGene00001572	gei-15	2.47937479879276	0.0002115202	-							
R03G5.5	WBGene00019846		2.48488	0.00141229	ENSP00000354677	ENSG00000116157	glutathione peroxidase 7	GPX7	0.0235	0.49	0.0199	0.695
T13C2.3a	WBGene00020479	-	2.48537479879276	0.0006925339	-							
Y37A1B.5	WBGene00012538		2.49469	0.0033336	ENSP00000357860	ENSG00000143416	Selenium binding protein 1	SELENBP1	0.0725	0.153	0.00548	0.922
Y73F8A.6	WBGene00000370	ccg-1	2.49537479879276	0.00001728567	-							
C39D10.3	WBGene00016531	-	2.49937479879276	0.00003537513	-							
F54G8.4	WBGene00003597	nhl-1	2.50082	0.000147755	ENSP00000339659	ENSG00000109654	tripartite motif-containing 2	TRIM2	0.085	0.196	-0.243	0.000794
B0252.2	WBGene00000211	asm-1	2.50337479879276	0.000035534	-							
Y5F2A.1	WBGene00012382	-	2.51337479879276	0.0005811352	-							
F11E6.1a	WBGene00008706		2.51554	0.000478231	ENSP00000314508	ENSG00000177628	group-specific component (vitamin D binding protein)	GC	0.00555	0.89	0.0344	0.487
Y17D7B.4	WBGene00012452	-	2.51637479879276	0.0006346431	-							
F13G3.12	WBGene00008770	-	2.51637479879276	0.0007785597	-							
R11A8.1	WBGene00011236	-	2.51637479879276	0.002571096	-							

F38A6.2	WBGene00001248	elp-1	2.5278	0.00135388	ENSP00000320663	ENSG00000143924	echinoderm microtubule associated protein like 4	EML4	-0.89	1.47e-11	-0.0312	0.683
F28H1.4	WBGene00017909		2.52903	0.000389158	ENSP00000219207	ENSG00000102934	plasma membrane proteolipid (plasmolipin)	PLLP	0.244	0.0496	-0.326	0.0171
Y39A3CL.6	WBGene00004249	pvf-1	2.53137479879276	0.000009511236	-							
C46G7.4a	WBGene00004112	pqn-22	2.53537479879276	0.00005026189	-							
F11F1.6	WBGene00008718	-	2.53937479879276	0.00009906586	-							
F46H5.3a	WBGene00018519		2.54006	0.000878851	ENSP00000299198	ENSG00000166165	creatine kinase, brain	CKB	-0.115	0.0322	0.0256	0.643
C05D9.3	WBGene00015472	-	2.54137479879276	0.0002940035	-							
T19B10.3	WBGene00011832		2.54374	0.00101518	ENSP00000306920	ENSG00000170266	Galactosidase, beta 1	GLB1	0.00193	0.969	-0.12	0.191
F52H3.7b	WBGene00002265	lec-2	2.55723	0.002692	ENSP00000318214	ENSG00000168961	lectin, galactoside-binding, soluble, 9 (galectin 9)	LGALS9	0.171	0.00398	-0.00962	0.85
Y71G12B.11b	WBGene00022151		2.55723	0.00242884	ENSP00000303476	ENSG00000171914	talin 2	TLN2	-0.0393	0.205	-0.049	0.35
M01F1.9	WBGene00010815		2.56091	7.2e-05	ENSP00000367318	ENSG00000107185	KIAA0258	KIAA0258	0.0648	0.0507	-0.0643	0.338
F55B12.5	WBGene00003812	nrf-5	2.56737479879276	0.00005997496	-							
Y48C3A.1	WBGene00012985	-	2.56837479879276	0.005743274	-							
F16B4.5b	WBGene00017507	-	2.57937479879276	0.0002212795	-							
Y75B12B.4	WBGene00013569	bath-36	2.59137479879276	0.003491992	-							
B0416.1	WBGene00015177	-	2.59737479879276	0.0005170232	-							
Y73B6BL.21	WBGene00022242		2.5977	0.00175978	ENSP00000220772	ENSG00000104332	secreted frizzled-related protein 1	SFRP1	0.122	0.191	0.0386	0.542
C29F5.2	WBGene00016211	sdz-3	2.60137479879276	0.0002691015	-							
B0213.17	WBGene00015046	nlp-34	2.61437479879276	0.00004483253	-							
W08D2.1	WBGene00001188	egl-20	2.61977	0.00179965	ENSP00000222462	ENSG00000002745	wingless-type MMTV integration site family, member 16	WNT16	0.0955	0.0227	0.0531	0.37

K03C7.1	WBGene00019358	-	2.62137479879276	0.000004955231	-								
F52E4.1b	WBGene00018701	pccb-1	2.62345	0.00256464	ENSP00000 251654	ENSG000 00114054	propionyl Coenzyme A carboxylase, beta polypeptide	PCCB	-0.0136	0.816	-0.228	0.0031
K10C9.3	WBGene00019624		2.62468	0.000941303	ENSP00000 028008	ENSG000 00026297	ribonuclease T2	RNASET2	0.161	0.131	-0.0269	0.783
Y47G6A.15	WBGene00021641	-	2.63037479879276	0.000893048	-							
B0244.8	WBGene00015083	egg-1	2.63337479879276	0.000009948764	-							
C24A3.6	WBGene00006672	twk-18	2.64337479879276	0.000008555732	-							
T09A12.2b	WBGene00020373		2.65043	0.00110949	ENSP00000 354677	ENSG000 00116157	glutathione peroxidase 7	GPX7	0.0235	0.49	0.0199	0.695
T06A4.2	WBGene00020282	mps-3	2.65737479879276	0.0000000001109	-							
F40A3.6	WBGene00018221	-	2.66337479879276	0.0007500735	-							
F21D9.4	WBGene00009018	-	2.66637479879276	0.000642002	-							
H12C20.2a	WBGene00004064	pms-2	2.68844	0.00034196	ENSP00000 265849	ENSG000 00122512						
T04A6.1b	WBGene00020200	-	2.69337479879276	0.0001683932	-							
F02A9.3	WBGene00001386	far-2	2.69637479879276	0.0009831883	-							
F10E7.4	WBGene00006893	spon-1	2.69703	5.18e-05	ENSP00000 309297	ENSG000 00152268	spondin 1, extracellular matrix protein	SPON1	0.0843	0.245	0.498	0.000132
Y113G7A.5	WBGene00013746	-	2.69937479879276	0.0003040117	-							
T21B6.2	WBGene00011879	-	2.70437479879276	0.000008262135	-							
F42E11.4	WBGene00006584	tni-1	2.71297	4.2e-05	ENSP00000 341838	ENSG000 00129991	troponin I, cardiac	TNNI3	-0.0113	0.696	0.00672	0.875
C34C6.7	WBGene00007919	-	2.71437479879276	0.00003869035	-							
T28A11.2	WBGene00020869	-	2.71637479879276	0.00004862974	-							
T19D2.3	WBGene00020568	-	2.71737479879276	0.000006740196	-							
K02A4.1	WBGene00001149	bcat-1	2.71787	0.00044546	ENSP00000 261192	ENSG000 00060982	branched chain aminotransferase 1, cytosolic	BCAT1	-0.0965	0.00291	-0.35	0.00038
F25E2.3	WBGene00017781		2.71787	0.00233283	ENSP00000 217455	ENSG000 00101473	Peroxisomal acyl-CoA thioesterase	PTE1	-0.0639	0.0453	0.0273	0.578
C03H5.5	WBGene00015407	-	2.72037479879276	0.00001597311	-							

Y55F3C.3	WBGene00021948		2.72646	0.000742442	ENSP00000360806	ENSG00000158445	potassium voltage-gated channel, Shab-related subfamily, member 1	KCNB1	-0.614	9.08e-08	-0.14	0.137
K08F4.9	WBGene00000975	dhs-12	2.73537479879276	0.0007899062	-							
T22H6.2b	WBGene00011938		2.73872	4.89e-05	ENSP00000360268	ENSG00000059573	aldehyde dehydrogenase 18 family, member A1	ALDH18A1	-0.0386	0.396	-0.201	0.00567
C07E3.9	WBGene00007419		2.75098	0.000377703	ENSP00000312286	ENSG00000170890	phospholipase A2, group IB (pancreas)	PLA2G1B	-0.0389	0.145	-0.0303	0.469
B0414.2	WBGene00004393	rnt-1	2.75344	0.000261655	ENSP00000352514	ENSG00000124813	runt-related transcription factor 2	RUNX2	0.0122	0.764	0.0917	0.0369
W06H8.8g	WBGene00006436	ttn-1	2.75537479879276	0.00002366637	-							
B0350.2a	WBGene00006780	unc-44	2.75834	0.000295866	ENSP00000349588	ENSG00000145362	Ankyrin 2, neuronal	ANK2	0.145	0.00138	0.152	0.119
F55A12.1	WBGene00018862	-	2.76037479879276	0.00008606812	-							
C18A11.7b	WBGene00001000	dim-1	2.76037479879276	0.000815084	-							
C29F5.1	WBGene00016210	-	2.76637479879276	0.0000106375	-							
Y37D8A.22	WBGene00012559		2.76692	2.49e-05	ENSP00000262291	ENSG00000062716	Likely ortholog of rat vacuole membrane protein 1	VMP1	0.000874	0.972	-0.00374	0.921
C47E12.12	WBGene00008155	-	2.76937479879276	0.001138466	-							
C46G7.2	WBGene00016722	-	2.77837479879276	0.00003518165	-							
Y19D10A.4	WBGene00021219	-	2.78137479879276	0.0002092524	-							
Y44A6D.3	WBGene00012854	-	2.78337479879276	0.00006453293	-							
T09A5.1b	WBGene00023408	cex-2	2.78537479879276	0.000000595114	-							
T07H6.4	WBGene00020331	-	2.83137479879276	0.0003002532	-							
F08B6.4b	WBGene00006819	unc-87	2.83237479879276	0.00002011985	-							
F38E11.1	WBGene00002012	hsp-12.3	2.83437	0.00172401	ENSP00000227251	ENSG00000109846	crystallin, alpha B	CRYAB	0.478	2.2e-05	0.0902	0.596
Y69E1A.5	WBGene00013477	-	2.83537479879276	0.0001492112	-							
T28D9.7	WBGene00020897	-	2.84737479879276	0.000004299733	-							
ZK721.2	WBGene00006764	unc-27	2.85522	0.000855479	ENSP00000341838	ENSG00000129991	troponin I, cardiac	TNNI3	-0.0113	0.696	0.00672	0.875

F53A3.1	WBGene00018720	-	2.85737479879276	0.00005250294	-								
C27B7.7	WBGene00007764	-	2.86437479879276	0.000008157557	-								
Y43F8B.11	WBGene00012821	-	2.87437479879276	0.00001457533	-								
T11F9.3	WBGene00003539	nas-20	2.89437479879276	0.0000078732	-								
K06A4.3	WBGene00010593	-	2.89737479879276	0.000001718981	-								
F22F7.1b	WBGene00017719		2.89936	0.000177433	ENSP00000355467	ENSG00000143653							
C07H6.7	WBGene00003024	lin-39	2.91408	0.00138343	ENSP00000222726	ENSG00000106004	homeo box A5	HOXA5	0.0225	0.603	0.0898	0.156	
Y75B8A.26	WBGene00003414	mrp-8	2.92337479879276	0.000007443263	-								
C04F12.9	WBGene00007303	rnh-1.3	2.92637479879276	0.0004006195	-								
C46F4.2	WBGene00016716	acs-17	2.93124	0.000273914	ENSP00000339787	ENSG00000068366	acyl-CoA synthetase long-chain family member 4	ACSL4	-0.204	0.000238	-0.0351	0.668	
F36F2.1	WBGene00009475		2.93247	1.33e-05	ENSP00000311436	ENSG00000174429							
T12D8.9	WBGene00011736	-	2.94037479879276	0.00008558027	-								
F40E10.3	WBGene00000822	csq-1	2.94106	0.000328623	ENSP00000357058	ENSG00000143318	calsequestrin 1 (fast-twitch, skeletal muscle)	CASQ1	0.00652	0.857	-0.06	0.405	
Y49F6C.8	WBGene00021730	-	2.94137479879276	0.00002011985	-								
W06A7.2	WBGene00012291	-	2.94337479879276	0.00003433974	-								
Y38F1A.9	WBGene00003860	oig-2	2.95437479879276	0.0001113101	-								
Y66H1B.2	WBGene00022048		2.95945	0.000719214	ENSP00000358879	ENSG00000196924	filamin A, alpha (actin binding protein 280)	FLNA	0.335	5.42e-07	0.0949	0.316	
F13D12.4b	WBGene00000114	alh-8	2.96068	0.0001872	ENSP00000342564	ENSG00000119711	Aldehyde dehydrogenase 6 family, member A1	ALDH6A1	0.21	0.0146	0.434	0.00156	
C38C3.5c	WBGene00006794	unc-60	2.96068	0.000377703	ENSP00000298159	ENSG00000165410	cofilin 2 (muscle)	CFL2	0.174	0.105	-0.313	0.00457	
W09B12.1	WBGene00000035	ace-1	2.96068	0.000297884	ENSP00000350037	ENSG00000087085	acetylcholinesterase (YT blood group)	ACHE	-0.468	4.9e-06	-0.165	0.01	
K06A9.3	WBGene00019437	-	2.97437479879276	0.00003140214	-								

C08D8.2b	WBGene00006582	tmd-2	2.98037479879276	0.000000937522	-								
Y41E3.2	WBGene00001066	dpy-4	2.98037479879276	0.000006740196	-								
F10G2.1	WBGene00017362	-	2.98037479879276	0.00002557041	-								
C37F5.1	WBGene00002990	lin-1	2.98275	0.00121813	ENSP00000350681	ENSG00000158711	ELK4, ETS-domain protein (SRF accessory protein 1)	ELK4	-0.0337	0.255	0.0229	0.537	
C44F1.5	WBGene00000070	acy-3	2.99637479879276	0.00005997496	-								
Y105C5A.8	WBGene00013633	-	2.99737479879276	0.00002986326	-								
ZK688.6b	WBGene00022801	pcp-5	3.00114	1.33e-05	ENSP00000317362	ENSG00000137509	Prolylcarboxypeptidase (angiotensinase C)	PRCP	0.0879	0.0123	-0.0877	0.14	
F37H8.5	WBGene00009514	-	3.01337479879276	0.00009813392	-								
EGAP4.1	WBGene00017149	-	3.01837479879276	0.0000238856	-								
Y22D7AL.10	WBGene00021248		3.04774	0.00230088	ENSP00000233893	ENSG00000115541	heat shock 10kDa protein 1 (chaperonin 10)	HSPE1	0.162	0.109	-0.0423	0.705	
F22A3.2	WBGene00017688	-	3.05137479879276	0.00001457533	-								
F45D11.2	WBGene00018449	-	3.06437479879276	0.000005259257	-								
F54D7.4	WBGene00006984	zig-7	3.06837479879276	0.00005173412	-								
R05D7.1	WBGene00011026	-	3.07237479879276	0.0004636422	-								
R11A5.4a	WBGene00011232		3.07717	0.000633094	ENSP00000216780	ENSG00000100889	Phosphoenolpyruvate carboxykinase 2 (mitochondrial)	PCK2	0.0167	0.507	-0.0222	0.548	
T22A3.4b	WBGene00044070	set-18	3.07717	3.13e-05	ENSP00000331557	ENSG00000185420	SET and MYND domain containing 3	SMYD3	-0.405	5.45e-06	-0.149	0.191	
Y43F8C.9	WBGene00012831	-	3.07937479879276	0.000009368688	-								
F21C10.7	WBGene00017657	-	3.08437479879276	0.000001237314	-								
C06A1.2	WBGene00007353	-	3.08637479879276	0.00004154819	-								
F53A9.10a	WBGene00006587	tnt-2	3.09189	9.66e-05	ENSP00000356286	ENSG00000118194	troponin T2, cardiac	TNNT2	-0.0381	0.262	0.00515	0.938	
F46C8.3	WBGene00018485	-	3.10737479879276	0.0002221317	-								
F20D6.10	WBGene00017639	-	3.10937479879276	0.0000668689	-								
Y54E10A.5	WBGene00021827		3.11764	7.37e-06	ENSP00000221114	ENSG00000104671	dynactin 6	DCTN6	-0.172	0.0401	-0.201	0.013	

C37C3.6a	WBGene00016498	ppn-1	3.11764	0.000319154	ENSP00000345395	ENSG00000100767	papilin, proteoglycan-like sulfated glycoprotein	PAPLN	0.0436	0.249	-0.113	0.0415
C50F7.6	WBGene00016846		3.12622	0.000158002	ENSP00000263666	ENSG00000121440	PDZ domain containing RING finger 3	PDZRN3	0.0374	0.392	0.0234	0.693
F26B1.7	WBGene00002601	let-381	3.14837479879276	0.0001146617	-							
C18B2.3	WBGene00015954	-	3.15737479879276	0.00001000268	-							
R148.6	WBGene00006452	heh-1	3.17437479879276	0.0000886648	-							
F15B10.2	WBGene00001090	drh-1	3.18631	0.000106383	ENSP00000369213	ENSG00000107201	DEAD (Asp-Glu-Ala-Asp) box polypeptide 58	DDX58	0.109	0.0382	0.0458	0.34
ZK666.5	WBGene00014045	clec-59	3.19637479879276	0.000004121831	-							
K02F3.9	WBGene00019332	-	3.23237479879276	0.000001566302	-							
T21B6.3	WBGene00011880	-	3.23237479879276	0.0001492112	-							
F09B9.4	WBGene00008603	-	3.23737479879276	0.000008127517	-							
Y5F2A.2	WBGene00012383	-	3.24037479879276	0.0001845487	-							
H14N18.3	WBGene00019203	-	3.24937479879276	0.000009067646	-							
F10A3.1	WBGene00008631	-	3.25337479879276	0.0000371559	-							
C47E12.8	WBGene00000964	dhp-2	3.25375	0.000100797	ENSP00000276651	ENSG00000147647	dihydropyrimidinase	DPYS	-0.0647	0.0978	-0.00139	0.98
Y39H10A.7a	WBGene00000498	chk-1	3.25375	0.00674464	ENSP00000278916	ENSG00000149554	CHK1 checkpoint homolog (S. pombe)	CHEK1	-0.0216	0.265	0.0368	0.151
T20B3.2	WBGene00006585	tni-3	3.25866	3.5e-05	ENSP00000341838	ENSG00000129991	troponin I, cardiac	TNNI3	-0.0113	0.696	0.00672	0.875
R02F2.2	WBGene00019832		3.26602	0.00391812	ENSP00000355060	ENSG00000074964						
ZK1058.9	WBGene00014207	-	3.27237479879276	0.0000080764	-							
T28H10.2	WBGene00012143		3.28196	6.9e-06	ENSP00000315682	ENSG00000176946						
D1086.2	WBGene00008389	-	3.28837479879276	0.0000005808792	-							
E04F6.8	WBGene00017127	-	3.28937479879276	0.000001718981	-							
F25H8.5b	WBGene00001113	dur-1	3.29037479879276	0.00001387518	-							
Y59C2A.1	WBGene00021984	-	3.29037479879276	0.0000413939	-							

C28C12.7a	WBGene00004995	spp-10	3.29054	0.00010351	ENSP00000362212	ENSG00000197746	prosaposin (variant Gaucher disease and variant metachromatic leukodystrophy)	PSAP	0.114	0.0314	-0.0695	0.246
F32E10.8	WBGene00017995	-	3.32837479879276	0.00008377214	-							
C32E12.4	WBGene00016330	-	3.33237479879276	0.0000001135195	-							
Y45F10B.13	WBGene00012875	-	3.33637479879276	0.000000860125	-							
Y17G9B.1	WBGene00021198	-	3.33837479879276	0.000001928172	-							
Y34D9A.6	WBGene00021331	glrx-10	3.34818	0.00209675	ENSP00000369314	ENSG00000173221	glutaredoxin (thioltransferase)	GLRX	0.0693	0.622	-0.0255	0.683
F44B9.2	WBGene00018407	-	3.36537479879276	0.00003049281	-							
E04F6.9	WBGene00017128	-	3.38137479879276	0.000005514039	-							
C25F9.5	WBGene00007725	-	3.38437479879276	0.000006740196	-							
C16B8.2	WBGene00015822	-	3.41137479879276	0.000001237314	-							
Y73B3A.3	WBGene00022205		3.41317	0.000687148	ENSP00000351632	ENSG00000198643	family with sequence similarity 3, member D	FAM3D	-0.0293	0.335	-0.00824	0.863
C02C2.5	WBGene00015334		3.4193	1.58e-05	ENSP00000229447	ENSG00000009765	chromosome 6 open reading frame 71	C6orf71	-0.0103	0.526	-0.0166	0.57
ZK1058.6	WBGene00014206	nit-1	3.42037479879276	0.000002468541	-							
Y43F8B.1a	WBGene00012812	-	3.42037479879276	0.00006690391	-							
ZK809.7	WBGene00004192	prx-2	3.42298	7.13e-07	ENSP00000349543	ENSG00000164751	Peroxisomal membrane protein 3, 35kDa (Zellweger syndrome)	PXMP3	0.0752	0.0373	0.0547	0.205
Y105E8A.6	WBGene00006824	unc-95	3.42337479879276	0.0001137647	-							
ZK678.6	WBGene00005221	srg-64	3.43337479879276	0.0006311375	-							
M03B6.4	WBGene00010836	-	3.44737479879276	0.0000001135195	-							
Y66D12A.11	WBGene00013437	-	3.45337479879276	0.00001999819	-							
E03G2.3	WBGene00003169	mec-5	3.46037479879276	0.0001586644	-							
F08D12.7	WBGene00017250	-	3.46737479879276	0.00002191732	-							
ZC412.3	WBGene00013884	-	3.47637479879276	0.0000006011814	-							

M70.4a	WBGene00019786		3.47939	0.000315743	ENSP00000351632	ENSG00000198643	family with sequence similarity 3, member D	FAM3D	-0.0293	0.335	-0.00824	0.863
C52D10.8	WBGene00004819	skr-13	3.48337479879276	0.00000537967	-							
C54G4.7	WBGene00008317		3.48429	0.00118908	ENSP00000373447	ENSG00000164074						
Y37D8A.2	WBGene00012544		3.48552	5.79e-05	ENSP00000280800	ENSG00000151176						
K12F2.1	WBGene00003515	myo-3	3.48797	4.17e-06	ENSP00000348634	ENSG00000197616	myosin, heavy chain 6, cardiac muscle, alpha (cardiomyopathy, hypertrophic 1)	MYH6	0.0246	0.21	-0.0157	0.595
Y54G2A.29	WBGene00021893		3.49533	2.34e-06	ENSP00000369014	ENSG00000173376						
T04B2.3	WBGene00011422	-	3.50637479879276	0.000003855723	-							
C48E7.10	WBGene00005000	spp-15	3.51737479879276	0.0000007056771	-							
K08C7.6	WBGene00010648	-	3.52237479879276	0.00001049961	-							
F21D12.5	WBGene00017664	-	3.52337479879276	0.00001126868	-							
R57.1a	WBGene00020082		3.53334	8.39e-05	ENSP00000256999	ENSG00000086205	folate hydrolase (prostate-specific membrane antigen) 1	FOLH1	0.172	0.273	-0.392	0.0336
F07A5.7	WBGene00006754	unc-15	3.54837479879276	0.00002191732	-							
ZK822.5	WBGene00014092		3.56645	5.35e-05	ENSP00000299233	ENSG00000139357	solute carrier family 5 (iodide transporter), member 8	SLC5A8	-0.0101	0.714	-0.0703	0.143
F33C8.3	WBGene00006634	tsp-8	3.58117	0.000152943	ENSP00000227155	ENSG00000085117	CD82 antigen	CD82	0.0684	0.122	0.021	0.755
Y71H2B.4	WBGene00022194	-	3.58537479879276	0.00001114327	-							

F01G12.5a	WBGene00002280	let-2	3.60201	0.000187299	ENSP00000 331902	ENSG000 00188153	collagen, type IV, alpha 5 (Alport syndrome)	COL4A5	-0.0398	0.297	0.0789	0.15
C12C8.1	WBGene00002026	hsp-70	3.61237479879276	0.0000000157963	-							
Y67D8C.8	WBGene00022072	cpg-9	3.62937479879276	0.000001928172	-							
Y22F5A.4	WBGene00003090	lys-1	3.63837479879276	0.0000371559	-							
D1007.14	WBGene00004113	pqn-24	3.64437479879276	0.0000001723639	-							
Y73C8C.9	WBGene00022266	srt-56	3.66037479879276	0.000005259257	-							
B0379.1	WBGene00007155	-	3.68137479879276	0.000004296551	-							
M04B2.5	WBGene00006678	twk-25	3.68837479879276	0.000002787109	-							
F01F1.3	WBGene00017160	-	3.71837479879276	0.000002355463	-							
F10G7.11	WBGene00017374	-	3.71837479879276	0.00002191732	-							
ZK899.5	WBGene00014143	-	3.72437479879276	0.00001157831	-							
F38A5.12	WBGene00018169	nspb-2	3.72637479879276	0.000001840312	-							
Y10G11A.3	WBGene00012425	-	3.74437479879276	0.0000007056771	-							
B0304.1c	WBGene00001948	hlh-1	3.74794	8.39e-05	ENSP00000 228641	ENSG000 00111046	myogenic factor 6 (herculin)	MYF6	-0.0332	0.37	0.0166	0.721
K09A9.6	WBGene00010703		3.75898	1.24e-05	ENSP00000 368767	ENSG000 00198363	aspartate beta-hydroxylase	ASPH	0.0439	0.228	0.049	0.296
C49C3.15	WBGene00008204	-	3.79837479879276	0.000008127517	-							
C46F11.1	WBGene00006822	unc-93	3.8068	5.09e-05	ENSP00000 230256	ENSG000 00112494	unc-93 homolog A (C. elegans)	UNC93A	0.0105	0.592	-0.0146	0.596
F13D12.10	WBGene00008744	-	3.82237479879276	0.000001158303	-							
C51E3.9	WBGene00008254	-	3.82537479879276	0.000006740196	-							
F54D12.4	WBGene00018817	-	3.82837479879276	0.00005161744	-							
Y47D7A.2	WBGene00021616	-	3.85637479879276	0.000001653945	-							
B0304.1b	WBGene00001948	hlh-1	3.87179	9.27e-05	ENSP00000 228641	ENSG000 00111046	myogenic factor 6 (herculin)	MYF6	-0.0332	0.37	0.0166	0.721
F41G4.8	WBGene00018311	-	3.87437479879276	0.0000005808792	-							
K08C7.3b	WBGene00001328	epi-1	3.89755	3.22e-07	ENSP00000 252999	ENSG000 00130702	laminin, alpha 5	LAMA5	0.184	1.46e-06	0.0172	0.8
F57B7.4	WBGene00003248	mig-17	3.95137479879276	0.000001718981	-							
E01G4.3	WBGene00008446	-	3.95737479879276	0.000035534	-							
Y10G11A.2	WBGene00012424	dlc-3	3.96037479879276	0.000000364122	-							
R08B4.2	WBGene00044330	alr-1	4.01772	7.18e-06	ENSP00000 368332	ENSG000 00004848	Aristaless related homeobox	ARX	0.0452	0.315	0.000287	0.996

H22K11.4	WBGene00019227	sgca-1	4.0214	7.18e-06	ENSP00000262018	ENSG00000108823	sarcoglycan, alpha (50kDa dystrophin-associated glycoprotein)	SGCA	0.0182	0.728	0.0406	0.477
Y11D7A.14	WBGene00012437	-	4.05637479879276	0.0000000001657	-							
C35A5.6	WBGene00007950	-	4.05637479879276	0.0000005808792	-							
F46G10.6	WBGene00003511	mxl-3	4.05819	4.87e-05	ENSP00000284165	ENSG00000125952	MYC associated factor X	MAX	0.171	0.00177	-0.109	0.0612
F56H11.1b	WBGene00001403	fbl-1	4.06677	2.57e-07	ENSP00000331544	ENSG00000077942	fibulin 1	FBLN1	0.23	0.0142	0.11	0.0377
F46C5.6	WBGene00009781		4.07536	4.71e-06	ENSP00000305924	ENSG00000119698	KIAA1622	KIAA1622	-0.109	0.00106	-0.0246	0.743
Y53G8B.2	WBGene00021818		4.0913	2.15e-05	ENSP00000198801	ENSG00000166391	monoacylglycerol O-acyltransferase 2	MOGAT2	-0.0556	0.0613	0.00217	0.963
ZK863.2	WBGene00000614	col-37	4.11537479879276	0.00001630102	-							
T10D4.9	WBGene00005554	sri-42	4.12137479879276	0.000001928172	-							
Y51A2D.14	WBGene00013081	-	4.12537479879276	0.0000007056771	-							
W08A12.2	WBGene00021079	-	4.15537479879276	0.0000002844905	-							
F02C9.1	WBGene00017172	-	4.15737479879276	0.000000860125	-							
F13H8.7	WBGene00017440		4.18817	8.21e-08	ENSP00000324343	ENSG00000100024	ureidopropionase, beta	UPB1	-0.0617	0.215	0.0486	0.533
F28H1.2	WBGene00000779	cpn-3	4.20137479879276	0.000001928172	-							
ZK1321.4	WBGene00014262	-	4.20537479879276	0.000000863392	-							
F40F4.3	WBGene00002253	lbp-1	4.21137479879276	0.000001928172	-							
F07H5.2	WBGene00004790	sgn-1	4.236	2.74e-05	ENSP00000371512	ENSG00000185053						
F41B4.1	WBGene00018257	-	4.28037479879276	0.0000001733434	-							
F22D6.11	WBGene00001643	gly-18	4.28259	0.000714453	ENSP00000365920	ENSG00000187210	Glucosaminyl (N-acetyl) transferase 1, core 2 (beta-1,6-N-acetylglucosaminyltransferase)	GCNT1	-0.0545	0.197	0.0197	0.73

F31A3.1	WBGene00000026	abu-3	4.51436	1.12e-05	ENSP00000334197	ENSG00000188155						
F56B6.4c	WBGene00006863	uvt-5	4.53275	3.11e-06	ENSP00000340736	ENSG00000163754	glycogenin	GYG	0.0223	0.761	-0.254	0.00969
C30F12.7	WBGene00016266		4.56341	4.89e-07	ENSP00000217901	ENSG00000067829	isocitrate dehydrogenase 3 (NAD+) gamma	IDH3G	-0.154	0.000117	-0.0152	0.713
K03H1.6	WBGene00010541	ttr-1	4.61537479879276	0.0000000157963	-							
F53A9.10b	WBGene00006587	tnt-2	4.69217	1.68e-06	ENSP00000356286	ENSG00000118194	troponin T2, cardiac	TNNT2	-0.0381	0.262	0.00515	0.938
C05E4.3	WBGene00005642	srp-1	4.80376	1.04e-06	ENSP00000283752	ENSG00000057149	serine (or cysteine) proteinase inhibitor, clade B (ovalbumin), member 4	SERPINB4	-0.0942	0.0771	0.108	0.0833
C05D11.4	WBGene00002881	let-756	4.84423	1.2e-05	ENSP00000371790	ENSG00000102678	fibroblast growth factor 9 (glia-activating factor)	FGF9	-0.545	1.18e-09	-0.33	0.00448
R119.2	WBGene00020088		4.88592	9.1e-06	ENSP00000349658	ENSG00000196236						
T22A3.2	WBGene00011906	hsp-12.1	4.97912	0.000148695	ENSP00000227251	ENSG00000109846	crystallin, alpha B	CRYAB	0.478	2.2e-05	0.0902	0.596
C02F12.7	WBGene00015356	tag-278	5.08825	2.66e-11	ENSP00000357460	ENSG00000111879	chromosome 6 open reading frame 60	C6orf60	0.0735	0.085	0.13	0.0185
F23D12.7	WBGene00009091	-	5.14837479879276	0.0000001733434	-							
C48E7.6	WBGene00016751		5.51132	4.41e-08	ENSP00000312506	ENSG00000173546	chondroitin sulfate proteoglycan 4 (melanoma-associated)	CSPG4	0.0576	0.0546	0.0233	0.627
