# Supplementary material for: The Wnt Receptor Ryk Reduces Neuronal and Cell Survival Capacity by Repressing FOXO Activity During the Early Phases of Mutant Huntingtin Pathogenicity
Source: PLoS Biol. 2014 Jun 24;12(6):e1001895. doi: 10.1371/journal.pbio.1001895 (PMC4068980; doi:10.1371/journal.pbio.1001895)
Supplement: Table S4 — Pathways and processes enriched in genes specifically deregulated by 128Q expression as shown by GSEA. n is the count of genes found in the gene set enriched in deregulation versus the total Gene Set. *Underlined are the genes deregulated in 128Q cells (FDR<0.01) or human HD caudate nucleus as previously reported [29] (p<0.01) or both. **%H is the percent of core genes with human homolog(s) as indicated by Inparanoid clusters. Gene sets are from Wormbook***, KEGG annotations$, and Gene Ontology annotations with¥ or without# Inferred from Electronic Annotation. (DOCX) [file pbio.1001895.s014.docx]

| **Gene set** | **NES** | **Nom p-val** | **FDR q-val** | ***n*** | **enriched genes/Human homologs*** | **% H**** |
| --- | --- | --- | --- | --- | --- | --- |
| Neuron Differentiation^#^ | -1.6 | 0.005 | 0.019 | 22/46 | unc-86, unc-37/TLE4, cnd-1/NEUROD1, ceh-17/ALX4, unc-4, zag-1/ZFHX1B, hda-4/HDAC4, unc-6/NTN1, unc-104, sad-1/BRSK2, unc-119/UNC119, unc-42, mec-3, mec-8/RBPMS, cdh-4/FAT, vab-1, ham-2, hlh-14, unc-73/C6orf134, lin-11/LHX1, unc-53, mec-17 | 55 |
| TGFβ Signaling*** | -1.43 | 0.052 | 0.207 | 14/31 | plx-2, scd-1, daf-7/GDF11, scd-2/ALK, bra-1/ZMYND11, sma-9, efn-4/EFNA1, daf-3, egl-4, ttx-1, sma-6/BMPR1B, daf-4, mab-20, lon-1 | 36 |
| G-Prot. Signaling Pathway^#^ | -1.39 | 0.078 | 0.079 | 16/29 | kqt-1, flp-3, gar-2, gpc-1/GNG12, egl-8, lat-1, gpa-11, flp-7, kqt-3/KCNQ1, dop-1, odr-10/OR13G1, acy-2, ser-2, egl-30, eat-16, C13B9.4/CALCR | 25 |
| Inositol Phosphate Metabolism^$^ | -1.3 | 0.118 | 0.115 | 12/31 | pqn-25, egl-8, Y75B8A.24/PIK4CA, nekl-4/NEK10, cal-4, dgk-5/DGKZ, plc-3/PLCG1, pkc-2, VZC374L.1/MAP2K4, ppk-1/PIP5K1A, dgk-4, cmd-1/CALM1 | 58 |
| Axon Guidance^¥^ | -1.28 | 0.138 | 0.067 | 15/36 | ajm-1, unc-53/NAV2, lin-11/LHX1, unc-73, itx-1, vab-1, F25F2.2/FAT, unc-119/UNC119, sad-1/BRSK2, ina-1/ITGA6, unc-104, unc-6/NTN1, zag-1/ZFHX1B, unc-4, ceh-17/ALX4 | 60 |
| Wnt Signaling*** | 1.32 | 0.087 | 0.1 | 11/51 | Lin-39/HOXA1, egl-20/WNT16, mom-2, lin-18/RYK,  lin-35/RBL2, mom-1/PORCN, mab-5/HOXB7, lin-9, bar-1, tlp-1/ZNF503, mes-1 | 64 |
| Glycolysis/ Glucogenesis^$^ | 1.72 | 0.008 | 0.011 | 14/30 | fbp-1/FBP1, R05F9.6/PGM1, LLC1.3/DLD, tpi-1/TPI1, gpd-1/GAPD, alh-9/ALDH7A1, ZK669.4/DBT, C30H6.7, gpd-4/GAPD, Y38F1A.6/ADHFE1, F57B10.3, C04C3.3/PDHB, pgk-1/PGK1, sur-5/AACS | 86 |
| Cell Cycle^¥^ | 1.73 | 0 | 0.002 | 80/  234 | skr-13, skr-12, mei-2, sel-8, skr-2/SKP1A, tbb-2,  bmk-1/KIF11, fog-1, mom-2, gpr-1, mom-1/PORCN,  air-2/AURKA, F17C11.10/WDHD1, gpr-2, apc-10, tba-1, rsa-1/PPP2R3C, kbp-1, atl-1, puf-8, him-14/MSH4, htp-2/HORMAD1, rec-8, rde-2, sep-1, spd-5, F33H2.6/FAM82B, sas-6, klp-15, lin-5, brd-1/BARD1, htp-3, mes-1, him-3/HORMAD1, C28C12.2, dpl-1/TFDP1, hsp-3, mnat-1/MNAT1, zim-3, htp-1/HORMAD1, spo-11/SPO11, M116.5, F23C8.9/TIPIN, san-1, klp-18/KIF15, czw-1/ZW10, mat-2/ANAPC1, C05C10.5, cki-1, uri-1/C19orf2,  apc-2/ANAPC2, hpr-17/RAD17, ima-2, sgo-1, zhp-3, mig-5, hcp-2/GCC2, rfc-4/RFC4, plk-1, fbf-2, snf-8, hcp-4, aspm-1, K06A5.2, sas-4, msh-5/MSH5, him-10, pry-1, cdk-1/CDC2, dpy-26, K06B9.4, him-6/BLM, mis-12, cks-1/CKS1B, his-73, ima-3/KPNA4, drsh-1/RNASEN, syp-2, D2096.11, fog-3/TOB2 | 39 |

**Table S4.** Cont.

| Mitochondria^#^ | 2.27 | 0 | 0 | 62/94 | C30F12.7/IDH3G, alh-8, alh-12, C37E2.1/IDH3B, R05D3.6, F45H10.3, Y71H2AM.5/COX6B1, Y39A3CR.4/TIMM8A, T09B4.9/TIMM44, alh-9/ALDH7A1, tomm-7/TOM7, F32D1.2, ucp-4/UCP4, asg-2/ATP5L, tin-13/TIMM13, tin-9.1, F40G9.2/COX17, K01C8.7/SLC25A32, Y71H2AM.4, R05G6.7/VDAC2, F15D3.7/TIMM23, exos-4.1/EXOSC4, C18E9.4, dif-1/SLC25A20, wah-1/PDCD8, asb-1/ATP5F1, cps-6/ENDOG, mai-1/ATPIF1, R07B7.10, F33A8.5/SDHD, C18E9.6/TOMM40, F23H12.2/TOMM20, alh-6,  gbh-2/TMLHE, Y119D3B.16/MRPL45, clk-1/COQ7, C42C1.10/SLC25A19, C33A12.1, cco-1/COX5B,  alh-7/ALDH5A1, R07E3.4, tin-10/TIMM10, cchl-1/HCCS, mai-2/ATPIF1, K02F3.2/SLC25A12, F59A2.3/C1QBP, F33D4.5/MRPL1, frh-1/FXN, F54C8.1/HADHSC, B0432.4/SLC25A11, F35G12.2/IDH3G, D2030.4/NDUFB7, F55G1.5/SLC25A22, D2096.1, tag-316/SLC25A4,  fzo-1/MFN2, drp-1/TOMM22, T12E12.4, K04F1.15/ALDH2, E04A4.5/TIMM17A, B0261.4/MRPL47, B0272.3/HADHSC | 77 |
| --- | --- | --- | --- | --- | --- | --- |
| Oxidative phosphorylation^$^ | 2.37 | 0 | 0 | 45/85 | F45H10.2, R05D3.6, F45H10.3, F57B10.14, Y71H2AM.5/COX6B1, cyc-1/CYC, F44G4.2, F29C4.2, T02H6.11/UQCRB, R04F11.2, isp-1/UQCRFS1, R07E4.3, T20H4.5/NDUFS8, atp-5, F26E4.6, gas-1/NDUSF2, tag-99/NDUSF2, F32D1.2, ucr-2.2/UQCRC2, F58F12.1/ATP5D, Y56A3A.19/NDUFAB1, asg-2/ATP5L, cco-2/COX5A, F40G9.2/COX17, ucr-2.3/UQCRC2, Y69A2AR.18, Y71H2AM.4, Y94H6A.8/NDUFA12, F16B4.6, C18E9.4, asg-1/ATP5L, Y54F10AM.5/NDUFA8, sdhb-1/SDHB, asb-1/ATP5F1, C25H3.9, sdha-2/SDHA, sdhd-1/SDHD, F37C12.3, Y57G11C.12/NDUFA6, C33A12.1, vha-14/ATP6V1D, cco-1/COX5B, ZK809.3, asb-2/ATP5F1, C25A1.13/MRPL43 | 60 |
