# Supplementary material for: The Wnt Receptor Ryk Reduces Neuronal and Cell Survival Capacity by Repressing FOXO Activity During the Early Phases of Mutant Huntingtin Pathogenicity
Source: PLoS Biol. 2014 Jun 24;12(6):e1001895. doi: 10.1371/journal.pbio.1001895 (PMC4068980; doi:10.1371/journal.pbio.1001895)
Supplement: Table S5 — RT-PCR analysis for hits of interest as emphasized by microarray data analysis. (DOCX) [file pbio.1001895.s015.docx]

| Gene name | Microarray data  (log ratio 128Q/19Q) | Type of deregulation | Module number | RT-PCR data  (2^-ΔΔCt 128Q/19Q) | Deregulation trend confirmed |
| --- | --- | --- | --- | --- | --- |
| *phg-1* | -4.54 | down | 27 (Fig. S3) | 0.18 | Yes |
| *abu-11* | -2.21 | down | 20 (Fig. S3) | 4.38 | No |
| *flp-1/*FRMF-like | -1.77 | down | 24 (Fig. S3) | 0.25 | Yes |
| *lit-1/*NLK | -1.34 | down | n.a. | 0.109 | Yes |
| *wrt-1*/DHH | -0.96 | down | 27 (Fig. S3) | 2.02 | No |
| *tsfm-1/*EF-Ts | 1.37 | up | 9 (Fig. S2) | 7.57 | Yes |
| *unc-129/*TGF-ß | 1.44 | up | 40 (Fig. S2) | 14.23 | Yes |
| *lin-18*/Ryk | 1.63 | up | 40 (Fig. S2) | 14.74 | Yes |
| *Glh-3* | 1.80 | up | 36 (Fig. S2) | 30.03 | Yes |
| *mom-1/*PORCN | 1.85 | up | 40 (Fig. S2) | 9.06 | Yes |
| *Cal-1* | 2.49 | up | 8 (Fig. S2) | 8.93 | Yes |
| *alh-8/*ALDH6A1 | 2.96 | up | 22 (Fig. S2) | 21.57 | Yes |

n.a., not applicable.
