# Supplementary material for: The Wnt Receptor Ryk Reduces Neuronal and Cell Survival Capacity by Repressing FOXO Activity During the Early Phases of Mutant Huntingtin Pathogenicity
Source: PLoS Biol. 2014 Jun 24;12(6):e1001895. doi: 10.1371/journal.pbio.1001895 (PMC4068980; doi:10.1371/journal.pbio.1001895)
Supplement: Table S6 — Pathways/processes highlighted by Fourier analysis and GSEA. (DOCX) [file pbio.1001895.s016.docx]

| **Fourier module number** | **deregulation** | **Functional content**  **(see Table S4, Gene set)** |
| --- | --- | --- |
| 24 (Fig. S3) | Down-regulated | G-protein signaling |
| 25 (Fig. S3) | Down-regulated | axon guidance |
| 7 (Fig. S3) | Up-regulated | cell cycle |
| 12 (Fig. S3) | Up-regulated | mitochondria |
| 18 (Fig. S3) | Up-regulated | cell cycle |
| 21 (Fig. S2) | Up-regulated | mitochondria, glycolysis |
| 22 (Fig. S2) | Up-regulated | mitochondria, glycolysis |
| 23 (Fig. S2) | Up-regulated | mitochondria |
| 25 (Fig. S2) | Up-regulated | glycolysis |
| 26 (Fig. S2) | Up-regulated | mitochondria |
| 37 (Fig. S2) | Up-regulated | mitochondria |
| 40 (Fig. S2) | Up-regulated | Wnt signaling, cell cycle |
