# Supplementary material for: The Wnt Receptor Ryk Reduces Neuronal and Cell Survival Capacity by Repressing FOXO Activity During the Early Phases of Mutant Huntingtin Pathogenicity
Source: PLoS Biol. 2014 Jun 24;12(6):e1001895. doi: 10.1371/journal.pbio.1001895 (PMC4068980; doi:10.1371/journal.pbio.1001895)
Supplement: Table S7 — Putative daf-16/FOXO transcriptional targets that are also found in the transcriptomic signature of expanded-polyQ nematode touch cells. Data on putative daf-16/FOXO targets are from the studies of Murphy et al. [60], MacElwee et al. [61], and Oh et al. [62]. *Microarray data. **Chromatin immunoprecipitation data. ***Gene expression entropy was assessed using entropy-based feature selection as previously described [5] across 14 HD-associated conditions including the striatum of N-terminal htt transgenic mice R6/2 (at 6 wk and 12 wk) and D9-N171-98Q (a.k.a. DE5; at 14 mo) [63],[64], full-length htt transgenic mice YAC128 (at 12 mo and 24 mo), knock-in mice CHL2 (at 22 mo) and HdH(Q92/Q92) (at 18 mo), caudate nucleus and BA4/BA9 cortex from post-mortem HD brains [29], blood samples from presymptomatic and symptomatic HD carriers [65], and HD-induced pluripotent stem (iPS) cells that were differentiated into neural stem cell (NSC) lines and that expressed 60 or 180 CAG repeats [66]. Most putative daf-16 targets that are highly conserved in the mouse have moderate to high entropy values, suggesting that their behavior is significantly dependent on the HD-associated context (cell type, time requirement) in which they operate. NA, not applicable. (DOCX) [file pbio.1001895.s017.docx]

| **Deregulation in 128Q nematode touch cells** | | | | **Putative *daf-16*/FOXO targets** | | | **Best mouse ortholog** | |
| --- | --- | --- | --- | --- | --- | --- | --- | --- |
| Gene Symbol | ORF | Up (U) or down (D) regulated | Log ratio (round to 3 decimals) | Murphy et al. 2003* [58] | MacElwee et al. 2003*  [59] | Oh et al. 2006** [60] | Gene Symbol | Entropy across HD-associated conditions  *** |
| ttr-1 | K03H1.6 | U | 4.004 | no | yes | no | NA |  |
| lbp-1 | F40F4.3 | U | 3.550 | no | yes | no | NA |  |
| O02237_CAEEL | E01G4.3 | U | 2.943 | yes | no | no | NA |  |
| O62234_CAEEL | F36F2.1 | U | 2.551 | yes | no | no | RNU4-5P | NA |
| O61980_CAEEL | H14N18.1 | U | 2.538 | yes | no | no | 1700003M02Rik | High |
| B7WN88_CAEEL | C34C6.7 | U | 2.486 | yes | no | no | NA |  |
| O45227_CAEEL | B0513.4 | U | 2.454 | no | no | yes | NA |  |
| ttr-5 | C40H1.5 | U | 2.382 | yes | no | no | NA |  |
| D0FY28_CAEEL | ZC247.1 | U | 2.374 | yes | no | no | Cog1 | Moderate |
| Q18660_CAEEL | C46F4.2 | U | 2.134 | yes | no | no | Fasn | High |
| F19C6.4 | F19C6.4 | U | 2.122 | no | no | yes | NA |  |
| O02286_CAEEL | R11A5.4 | U | 2.062 | yes | no | no | Rhot1 | High |
| hsp-12.3 | F38E11.1 | U | 1.979 | yes | no | no | Rps10-ps1 | NA |
| tre-3 | W05E10.4 | U | 1.930 | yes | no | no | Ensa | Low |
| O62233_CAEEL | F36F2.2 | U | 1.898 | yes | no | no | NA |  |
| tps-2 | F19H8.1 | U | 1.797 | yes | no | no | NA |  |
| Q5WRM0_CAEEL | K06G5.1 | U | 1.781 | yes | no | no | NA |  |
| lec-2 | F52H3.7 | U | 1.768 | yes | no | no | RNU7-10P | NA |
| Q22705_CAEEL | T23G7.3 | U | 1.718 | yes | no | no | Smim8 | NA |
| C6KRP5_CAEEL | F02H6.5 | U | 1.686 | yes | no | no | Sf3a3 | High |
| msra-1 | F43E2.5 | U | 1.668 | no | yes | no | Sh3glb2 | High |
| gst-4 | K08F4.7 | U | 1.611 | yes | no | no | Itih3 | Moderate |
| skr-11 | F13A7.9 | U | 1.595 | yes | no | no | NA |  |
| TPS1_CAEEL | ZK54.2 | U | 1.567 | yes | no | no | NA |  |
| YSX3_CAEEL | T28D9.3 | U | 1.553 | yes | no | no | AL121932.1 | NA |
| MMAC_CAEEL | ZK546.17 | U | 1.552 | no | no | yes | Mob3b | NA |
| O17373_CAEEL | T13B5.3 | U | 1.505 | yes | no | no | Ifnk | Moderate |
| Q95ZT6_CAEEL | F43H9.4 | U | 1.499 | yes | no | no | NA |  |
| Q93765_CAEEL | F53C11.1 | U | 1.461 | yes | no | no | NA |  |
| PROD_CAEEL | B0513.5 | U | 1.392 | no | no | yes | 3110043O21Rik | High |
| F42G10.1 | F42G10.1 | U | 1.284 | no | no | yes | NA |  |
| hsp-12.6 | F38E11.2 | U | 1.274 | yes | no | no | Rhbdl3 | Low |
| gei-14 | K01C8.5 | U | 1.232 | yes | no | no | NA |  |
| Q9XV94_CAEEL | F16H6.7 | U | 1.201 | yes | no | no | NA |  |
| Q20420_CAEEL | F45D3.4 | U | 1.199 | yes | no | no | NA |  |
| Q965W3_CAEEL | Y40B10A.2 | U | 1.034 | yes | no | no | 5730455P16Rik | Low |
| Q22860_CAEEL | Y71G12B.11 | U | 0.829 | no | no | yes | Inpp5b | 0 |
| Q86FS9_CAEEL | K07B1.4 | U | 0.781 | yes | yes | no | Cwc27 | Moderate |
| mec-17 | F57H12.7 | D | -2.892 | yes | no | no | Lingo2 | Moderate |
| Q22562_CAEEL | T19B10.2 | D | -2.710 | yes | no | no | NA |  |

| Q93809_CAEEL | F58B3.9 | D | -2.381 | yes | no | no | NA |  |
| --- | --- | --- | --- | --- | --- | --- | --- | --- |
| lys-8 | C17G10.5 | D | -2.308 | yes | no | no | NA |  |
| O62277_CAEEL | F58G1.4 | D | -2.181 | yes | no | no | NA |  |
| Q20724_CAEEL | F53F4.13 | D | -1.944 | yes | no | no | NA |  |
| clc-1 | C09F12.1 | D | -1.883 | yes | no | no | NA |  |
| Q19478_CAEEL | F15B9.1 | D | -1.877 | yes | no | no | NA |  |
| Q22614_CAEEL | T20G5.8 | D | -1.876 | yes | no | no | NA |  |
| Q9XX55_CAEEL | Y38H6C.3 | D | -1.870 | yes | no | no | NA |  |
| O61874_CAEEL | ZK6.10 | D | -1.786 | yes | no | no | NA |  |
| Q9XXR5_CAEEL | Y51A2D.11 | D | -1.744 | yes | no | no | NA |  |
| lec-10 | W01A11.4 | D | -1.700 | yes | no | no | NA |  |
| ttr-2 | K03H1.4 | D | -1.688 | no | yes | no | NA |  |
| cyp-35C1 | C06B3.3 | D | -1.662 | yes | no | no | Gm10764 | NA |
| O01920_CAEEL | F23H11.7 | D | -1.617 | yes | no | no | NA |  |
| Q23179_CAEEL | W05H9.1 | D | -1.569 | yes | no | no | NA |  |
| TTC36_CAEEL | F52H3.5 | D | -1.52 | yes | no | no | Tars2 | Low |
| Q19591_CAEEL | F19C7.1 | D | -1.499 | no | no | yes | NA |  |
| myo-2 | T18D3.4 | D | -1.489 | yes | no | no | Rgs7bp | Moderate |
| Q23190_CAEEL | W06B11.3 | D | -1.486 | yes | no | no | NA |  |
| Q9TXT4_CAEEL | F53C3.5 | D | -1.481 | yes | no | no | NA |  |
| Q22774_CAEEL | T25C12.3 | D | -1.47 | yes | no | no | NA |  |
| Q95Y17_CAEEL | Y41D4B.16 | D | -1.434 | yes | no | no | NA |  |
| flp-1 | F23B2.5 | D | -1.410 | yes | no | no | NA |  |
| Q65ZB0_CAEEL | C08B6.4 | D | -1.391 | yes | no | no | NA |  |
| O02361_CAEEL | F35E12.9 | D | -1.329 | yes | no | no | NA |  |
| F36H5.8 | F36H5.8 | D | -1.328 | yes | no | no | NA |  |
| NHR62_CAEEL | Y67A6A.2 | D | -1.296 | yes | no | no | NA |  |
| YZ10_CAEEL | F08B12.4 | D | -1.290 | yes | no | no | NA |  |
| O45622_CAEEL | H19N07.1 | D | -1.250 | yes | no | no | Ipo11 | Moderate |
| O61834_CAEEL | F41A4.1 | D | -1.240 | yes | no | no | NA |  |
| hgo-1 | W06D4.1 | D | -1.227 | yes | no | no | Ddx58 | High |
| P91491_CAEEL | T23B3.2 | D | -1.195 | yes | no | no | NA |  |
| A3QM90_CAEEL | C52D10.1 | D | -1.177 | yes | no | no | NA |  |
| Q20675_CAEEL | F52E4.5 | D | -1.174 | yes | no | no | NA |  |
| Q9XUD5_CAEEL | F55G11.7 | D | -1.165 | yes | no | no | NA |  |
| Q19128_CAEEL | F02G3.1 | D | -1.133 | yes | no | no | Ccdc6 | Moderate |
| Q17821_CAEEL | C08B6.10 | D | -1.087 | yes | no | no | NA |  |
| Q9XX57_CAEEL | Y38H6C.1 | D | -1.050 | yes | no | no | NA |  |
| P91844_CAEEL | ZC410.5 | D | -1.041 | yes | no | no | NA |  |
| jnk-1 | B0478.1 | D | -1.039 | yes | no | no | Ndufb6 | High |
| ZK757.1 | ZK757.1 | D | -1.037 | yes | no | no | NA |  |
| Q9U3Q6_CAEEL | C08F11.8 | D | -0.949 | yes | no | no | Aptx | High |
| Q18639_CAEEL | C45B11.3 | D | -0.937 | yes | no | no | Sdk2 | Low |
| O44659_CAEEL | B0213.15 | D | -0.893 | yes | no | no | Tmem98 | Low |
