# Supplementary material for: The Wnt Receptor Ryk Reduces Neuronal and Cell Survival Capacity by Repressing FOXO Activity During the Early Phases of Mutant Huntingtin Pathogenicity
Source: PLoS Biol. 2014 Jun 24;12(6):e1001895. doi: 10.1371/journal.pbio.1001895 (PMC4068980; doi:10.1371/journal.pbio.1001895)
Supplement: Table S8 — Names and genotypes of the C. elegans strains used in this study. (DOCX) [file pbio.1001895.s018.docx]

| **Name** | **Genotype^a^** | **Origin** |
| --- | --- | --- |
| Bristol N2 | Standard wild type | CGC^b^ |
| CB620 | *lin-18(e620)X* | CGC |
| CF1038 | *daf-16(mu86)I* | CGC |
| EW15 | *bar-1(ga80)X* | CGC |
| ID245^c^ | *igIs245*[*mec-3p::htt57-19Q::CFP; lin-15(+); mec-7p::YFP*] | See Parker et al., 2005 |
| ID1^c^ | *igIs1*[*mec-3p::htt57-128Q::CFP; lin-15(+); mec-7p::YFP*] | See Parker et al., 2005 |
| ID1318^c^ | *igIs245*[*mec-3p::htt57-19Q::CFP; lin-15(+); mec-7p::YFP*]; *lin-18(e620)X* | This study |
| ID1319^c^ | *igIs1*[*mec-3p::htt57-128Q::CFP; lin-15(+); mec-7p::YFP*]; *lin-18(e620)X* | This study |
| ID1281^c^ | *igIs1*[*mec-3p::htt57-128Q::CFP; lin-15(+); mec-7p::YFP*]; *bar-1(ga80)X* | See Parker et al., 2012 |
| ID1320^c^ | *igIs1*[*mec-3p::htt57-128Q::CFP; lin-15(+); mec-7p::YFP*]; *daf-16(mu86)I* | This study |
| ID1321^c^ | *igIs1*[*mec-3p::htt57-128Q::CFP; lin-15(+); mec-7p::YFP*]; *lin-18(e620)X ; bar-1(ga80)X* | This study |
| ID1322^c^ | *igIs1*[*mec-3p::htt57-128Q::CFP; lin-15(+); mec-7p::YFP*]; *lin-18(e620)X ; daf-16(mu86)I* | This study |
| ID1323^c,d^ | *igIs245*[*mec-3p::htt57-19Q::CFP; lin-15(+); mec-7p::YFP*]; *lin-18(e620)X; igEx520[mec-3p::lin-18; myo-2p::GFP]* | This study |
| ID1324^c,d^ | *igIs245*[*mec-3p::htt57-19Q::CFP; lin-15(+); mec-7p::YFP*]; *lin-18(e620)X; igEx521[mec-3p::lin-18; myo-2p::GFP]* | This study |
| ID1325^c,d^ | *igIs1*[*mec-3p::htt57-128Q::CFP; lin-15(+); mec-7p::YFP*]; *lin-18(e620)X; igEx522[mec-3p::lin-18; myo-2p::GFP]* | This study |
| ID1326^c,d^ | *igIs1*[*mec-3p::htt57-128Q::CFP; lin-15(+); mec-7p::YFP*]; *lin-18(e620)X; igEx523[mec-3p::lin-18; myo-2p::GFP]* | This study |
| ID1327^c,e^ | *igIs245*[*mec-3p::htt57-19Q::CFP; lin-15(+); mec-7p::YFP*]; *lin-18(e620)X; igEx524[mec-3p::lin-18; myo-2p::GFP]* | This study |
| ID1328^c,e^ | *igIs245*[*mec-3p::htt57-19Q::CFP; lin-15(+); mec-7p::YFP*]; *lin-18(e620)X; igEx525[mec-3p::lin-18; myo-2p::GFP]* | This study |
| ID1329^c,e^ | *igIs1*[*mec-3p::htt57-128Q::CFP; lin-15(+); mec-7p::YFP*]; *lin-18(e620)X; igEx526[mec-3p::lin-18; myo-2p::GFP]* | This study |
| ID1330^c,e^ | *igIs1*[*mec-3p::htt57-128Q::CFP; lin-15(+); mec-7p::YFP*]; *lin-18(e620)X; igEx527[mec-3p::lin-18; myo-2p::GFP]* | This study |

**Table S8.** Cont.

| ID1331^c,d^ | *igIs245*[*mec-3p::htt57-19Q::CFP; lin-15(+); mec-7p::YFP*]; *lin-18(e620)X; igEx528[mec-3p::lin-18ICD; myo-2p::GFP]* | This study |
| --- | --- | --- |
| ID1332^c,d^ | *igIs245*[*mec-3p::htt57-19Q::CFP; lin-15(+); mec-7p::YFP*]; *lin-18(e620)X; igEx529[mec-3p::lin-18ICD; myo-2p::GFP]* | This study |
| ID1333^c,d^ | *igIs1*[*mec-3p::htt57-128Q::CFP; lin-15(+); mec-7p::YFP*]; *lin-18(e620)X; igEx530[mec-3p::lin-18ICD; myo-2p::GFP]* | This study |
| ID1334^c,d^ | *igIs1*[*mec-3p::htt57-128Q::CFP; lin-15(+); mec-7p::YFP*]; *lin-18(e620)X; igEx531[mec-3p::lin-18ICD; myo-2p::GFP]* | This study |
| ID1335^c,e^ | *igIs245*[*mec-3p::htt57-19Q::CFP; lin-15(+); mec-7p::YFP*]; *lin-18(e620)X; igEx532[mec-3p::lin-18ICD; myo-2p::GFP]* | This study |
| ID1336^c,e^ | *igIs245*[*mec-3p::htt57-19Q::CFP; lin-15(+); mec-7p::YFP*]; *lin-18(e620)X; igEx533[mec-3p::lin-18ICD; myo-2p::GFP]* | This study |
| ID1337^c,e^ | *igIs1*[*mec-3p::htt57-128Q::CFP; lin-15(+); mec-7p::YFP*]; *lin-18(e620)X; igEx534[mec-3p::lin-18ICD; myo-2p::GFP]* | This study |
| ID1338^c,e^ | *igIs1*[*mec-3p::htt57-128Q::CFP; lin-15(+); mec-7p::YFP*]; *lin-18(e620)X; igEx535[mec-3p::lin-18ICD; myo-2p::GFP]* | This study |
| ID1339^c,d^ | *igIs1*[*mec-3p::htt57-128Q::CFP; lin-15(+); mec-7p::YFP*]; *lin-18(e620)X; igEx536[mec-3p-empty-vector; myo-2p::GFP]* | This study |
| ID1340^c,d^ | *igIs1*[*mec-3p::htt57-128Q::CFP; lin-15(+); mec-7p::YFP*]; *lin-18(e620)X; igEx536[mec-3p-empty-vector; myo-2p::GFP]* | This study |
