# Supplementary material for: The Wnt Receptor Ryk Reduces Neuronal and Cell Survival Capacity by Repressing FOXO Activity During the Early Phases of Mutant Huntingtin Pathogenicity
Source: PLoS Biol. 2014 Jun 24;12(6):e1001895. doi: 10.1371/journal.pbio.1001895 (PMC4068980; doi:10.1371/journal.pbio.1001895)
Supplement: Text S1 — Supplementary materials and methods. (DOC) [file pbio.1001895.s019.doc]

**Text S1. Supplementary results.**

First-pass analysis of microarray data

Nearly 50% of the genes deregulated by 128Q expression and annotated in GO encoded membrane or extracellular matrix proteins (Figure S1), consistent with the notion that cell communication may be altered in expanded polyQ cells [1]. Nearly 1/5 of 128Q-dysregulated genes were associated to the nucleus, supporting the notion that mutant HTT fragments alter gene transcription [2]. Metabolic disturbance is a feature of HD [3], and, consistently, greater than 25% of 128Q-dysregulated genes were associated to catabolic activity. Of the 2070 128Q-deregulated genes, 516 genes have human homologs and 139/516 genes were previously found to be dysregulated in the striatum of HD patients [4], suggesting that expression data in nematode cells are relevant to HD pathogenesis. Furthermore, GO analysis of these 139 genes suggested that mitochondrial function, intracellular trafficking, catabolism, and, interestingly, neuron differentiation pathways are altered in 128Q nematode neurons. GO analysis indeed indicated that the 139 genes found to be deregulated by 128Q expression in nematodes and known to be deregulated in the striatum of HD patients are enriched for the ‘regulation of neuron differentiation’ (GO:0045664, *P*< 0.0001), ‘central nervous system development’ (GO:0007417, *P* < 0.001), ‘amine catabolic process’ (GO:0009310, *P* < 0.0001), ‘cellular component organization and biogenesis’ (GO:0016043, *P* < 0.001), ‘mitochondrion’ (GO:0005739, *P* < 0.0001), ‘nuclear envelope-endoplasmic reticulum network’ (GO:0042175, *P*< 0.01), ‘clathrin-coated endocytic vesicle’ ([GO:0045334](http://amigo.geneontology.org/cgi-bin/amigo/go.cgi?view=details&search_constraint=terms&query=GO:0045334), *P* < 0.01) and ‘extracellular matrix part’ (GO:0044420, *P* < 0.01).

Network-based analysis of microarray data highlights axon guidance pathways

The GO analysis of 128Q-dysregulated genes was poorly instructive as the enrichments detected were limited to ‘protein modification’ and ‘phosphate transport’ (*P* < 0.001), ‘structural constituent of cuticle’ (*P* < 0.001) and ‘extracellular matrix’ (*P* < 0.001). In contrast, the use of two complementary methods, Gene Set Enrichment Analysis (GSEA) and a network-based method that is based on Fourier analysis (see the supplemental Material and Methods section), was highly intructive.

Regarding down-regulation, GSEA highlighted ‘Neuron differentiation’, ‘TGF-ß signaling’ and ‘Axon guidance’as new components that may be altered in the early phases of expanded polyQ neurotoxicity (Tables S3, S4). Regarding up-regulation, mitochondrial components (especially oxidative phosphorylation), cell cycle genes and nuclear genes were highlighted. Interestingly, a ‘Neuron differentiation’ component was also identified, namely the Wnt pathway (Tables S3, S4). Thus, GSEA highlighted (*i*) several biological processes previously suspected to be altered in HD [2], which further validated the relevance of nematode data to HD pathogenesis, and (*ii*) cell differentiation pathways such as Wnt signaling as new components involved in the early phases of expanded polyQ neurotoxicity.

In biological networks, highly inter-connected genes (modules) participate into the same pathway(s) or biological process(es). The modules that are enriched in deregulated genes may thus correspond to a pathway or process that is altered in disease, which makes network-based analysis a sensitive approach for the functional deconvolution of gene expression data. Using a stringent network-based method (Fourier analysis) and the reference network Wormnet (see Supplemental Material and Methods), we identified 68 high-confidence modules enriched for genes deregulated by expanded polyQs (Figure S3, up-regulation; Figure S4, down-regulation). Down-regulated modules 27 and 28 contained patched proteins that in mammals are part of Hedgehog, a pathway that has a role in axon guidance [5]. This suggested that Hedgehog signaling might be down-regulated in HD neurons, which is consistent with previously-reported data that indicate that the stimulation of Hedgehog may promote functional recovery in models of neurodegenerative disease [6]. Upregulated module 40 (Wnt/TGF-ß signaling) was of particular interest as it suggested that *lin-18*/Ryk, a Wnt receptor important during neurogenesis and axon guidance [7,8], is up-regulated in HD neurons.

We next used qRT-PCR on the mRNAs extracted from primary cultures of touch receptor cells to assess the specificity of microarray data. Network-based methods strongly reduce the biases associated with false positives and false negatives, thus having an enhanced ability to predict deregulated pathways/processes [9]. We selected a group of 12 key hits that belong to relatively-large (at least 4 nodes) modules of interest such as the up-regulated ‘canonical Wnt’ module 40 (Table S5). While not contained in these modules, *lit-1*/NLK was included in the group test since this gene regulates TCF activity in canonical Wnt. Deregulation for 10/12 genes was validated by qRT-PCR (Table S5), with 3/3 genes confirmed to be deregulated in up-regulated module 40. Importantly, *lin-18*/Ryk was observed to be strongly up-regulated in 128Q nematodes (Table S5).

**References**

1. Luthi-Carter R, Strand A, Peters NL, Solano SM, Hollingsworth ZR, et al. (2000) Decreased expression of striatal signaling genes in a mouse model of Huntington's disease. Hum Mol Genet 9: 1259-1271.

2. Gil JM, Rego AC (2008) Mechanisms of neurodegeneration in Huntington's disease. Eur J Neurosci 27: 2803-2820.

3. Walker FO (2007) Huntington's disease. Lancet 369: 218-228.

4. Hodges A, Strand AD, Aragaki AK, Kuhn A, Sengstag T, et al. (2006) Regional and cellular gene expression changes in human Huntington's disease brain. Hum Mol Genet 15: 965-977.

5. Bourikas D, Pekarik V, Baeriswyl T, Grunditz A, Sadhu R, et al. (2005) Sonic hedgehog guides commissural axons along the longitudinal axis of the spinal cord. Nat Neurosci 8: 297-304.

6. Dellovade T, Romer JT, Curran T, Rubin LL (2006) The hedgehog pathway and neurological disorders. Annu Rev Neurosci 29: 539-563.

7. Lu W, Yamamoto V, Ortega B, Baltimore D (2004) Mammalian Ryk is a Wnt coreceptor required for stimulation of neurite outgrowth. Cell 119: 97-108.

8. Colavita A, Krishna S, Zheng H, Padgett RW, Culotti JG (1998) Pioneer axon guidance by UNC-129, a C. elegans TGF-beta. Science 281: 706-709.

9. Wu J, Lenchik NI, Gerling IC (2008) Approaches to reduce false positives and false negatives in the analysis of microarray data: applications in type 1 diabetes research. BMC Genomics 9 Suppl 2: S12.
