# Supplementary material for: The Wnt Receptor Ryk Reduces Neuronal and Cell Survival Capacity by Repressing FOXO Activity During the Early Phases of Mutant Huntingtin Pathogenicity
Source: PLoS Biol. 2014 Jun 24;12(6):e1001895. doi: 10.1371/journal.pbio.1001895 (PMC4068980; doi:10.1371/journal.pbio.1001895)
Supplement: Text S2 — Supplementary results. (DOC) [file pbio.1001895.s020.doc]

**Text S2. Supplementary materials and methods.**

#### FACS sorting and RNA extraction

#### Cell sorting experiments were performed on a Moflo flow cytometer (Dako). GFP-positive cells were directly collected in trizol LS (Invitrogen) after sorting and total RNAs extracted according to the manufacturer's protocol. The quality of RNAs was controlled on a Bioanalyzer (Agilent).

#### Microarray analysis

#### RNAs were processed with a one-round linear amplification with MessageAmp kit (Ambion) and labelled with the CyScribe Post-Labelling kit (Amersham), and the quality of cDNAs was controlled on a Bioanalyzer (Agilent) at each step. Probes were hybridized on dual color Agilent 22k microarrays according to the manufacturer protocol. Three independent experiments were performed, each of them with dye-swap and as it follows: 128Q::GFP *vs* 19Q::GFP and 19Q::GFP *vs* GFP alone, for a total of 12 microarrays. Microarrays were scanned using a Genepix 4000B scanner (Axon) and analyzed using the Genepix Pro software. Statistical analysis was performed using the Varmixt (VM) tool [1] of the R package, using VM parameters and FDR correction of P-values.

#### Microarray data analysis

#### Deregulated genes were initially classified using Gene Ontology annotations for Biological Process, Molecular Function and Cellular Component ([**http://www.geneontology.org/**](http://www.geneontology.org/)) [2]. Gene Ontology enrichment tests were performed using GOstat (<http://gostat.wehi.edu.au/>) [3]. Gene Set Enrichment Analysis (GSEA) [4,5] was performed using the ‘Pre-ranked gene list’ tool. The gene sets used for GSEA were established using core components of (i) canonical pathways based on information from Wormbook ([**http://www.wormbook.org**](http://www.wormbook.org/)) and including the Wnt, Notch, RTK/Ras/MAPK and TGF-ß pathways [6,7,8,9], and (ii) biological processes based on information from KEGG ([**http://www.genome.ad.jp/kegg/**](http://www.genome.ad.jp/kegg/)) [10] and GO omitting Inferred from Electronic Annotation (IEA) ([**http://www.geneontology.org/**](http://www.geneontology.org/)) [2]. To perform network-based data analysis, we used the integrated *C. elegans* network Wormnet [11]. This large-coverage network contains gene interaction data that were established according to several types of evidences derived from multiple organisms, including several types of gene interactions based on gene co-expression (microarray measurements of the expression of *C. elegans* mRNAs), protein-protein interactions including genome-wide yeast two-hybrid interactions between *C. elegans* proteins and protein-protein interactions from the Worm Interactome database [12], genetic interactions from WormBase [13], human and fly ‘interologs’ *i.e.* human and fly protein interactions [14,15], Yeast ‘associalogs’ *i.e.* functional interactions transferred from the yeast functional gene network [16], phylogenic profiles (comparative genomics linkages derived from the analysis of bacterial and archaeal genomes), gene neighbors and co-citation (literature-mined *C. elegans* gene associations). We elected to use the high confidence core network [11], and, to avoid association artifacts, we omitted to use the interactions based on gene co-expression, which resulted in a network of 5,289 genes and 51,748 interactions. We then applied a Fourier analysis as previously described [17]. This approach is based on the spectral decomposition of gene expression profiles with respect to the connectivity of the reference network. Expression data are considered as a ‘signal’ and the Fourier transformation decomposes the signal as a superposition of signals at different frequencies, with 20 successive attenuations performed [17]. The aim of these transformations is to keep the low-frequency signals and discard the high frequency signals, considered as noise, in the context of gene connectivity. The logic behind this procedure is that highly inter-connected genes may be implicated in the same pathway and may fluctuate in the same direction [17]. Starting from the raw expression data, we iteratively removed the high-frequency components 5% at a time, resulting in 20 increasingly smoothed versions of the expression data. Given a smoothed expression data, we considered modules as groups of connected genes with a signal > 1 or < -1. Additionally, and to generate an even more relevant signal and robust information, we selected the modules that were present after the 6^th^ attenuation and that showed a stable gene content (≥ 50% of the genes with a value over the threshold) for at least 4 successive attenuations. This resulted in 68 highly-stable modules out of a total of 242 modules initially generated by the Fourier analysis. The functional contents of these 68 stables modules were analyzed using Gostat [3], complemented by the analysis of the literature.

#### Reverse transcription-PCR

#### Quantitative RT-PCR was performed on htt-expressing transgenes in different worm mutants and on a group of deregulated genes (as indicated by microarray data analysis) in 128Q *vs* 19Q nematodes. RNA was isolated from young adults from stage-synchronized populations by extraction with a Qiagen RNeasy kit and Dnase I treatment (as per the manufacturer’s protocol). Single strand cDNA synthesis was done using oligoDT and random hexamer priming and 100 ng of total RNA with Absolute Blue Verso 2-Step kit (Thermofisher). Quantitative PCR was performed using SYBR® Green with the ABI PRISM® 7700 Sequence Detection System (Applied Biosystems) and the oligonucleotides (5’–3’): htt-f (CACTTGTCACTACTTTCTCAT), htt-r (GTAGTTCCCGTCATCTTTG), col-1f (catctttgctgaggtcaacta), col-1r (catccctcgcattggagatt). The oligonucleotides used for assessment of specificity of microarray data were (5’–3’): pgh-1f (GATGGCTGCTTGCTCCACTAAC), pgh-1r (ACGTCCTGGGATGCAAGTG), abu-11f (GAATGTGTTCCTCCGCGCTC), abu-11r (AGCAAGATTGTTGGGCGGTG), lit-1f (AGTTGTGCCACGATCTGAC), lit-1r (ATGATGATGGTGAGCCTGC), wrt-1f (AGTTTTATGGCAGGTGCTGACG), wrt-1r (CGTGGACAGTTTGCCATTTGAG), tsfm-1f (TCGAGTCCAGGGTCAATCAGTAG), tsfm-1r (TCAGCATTTGGATCATGCCCTTC), unc-129f (CTGCGGAACCAGTTTGTTG), unc-129r (CAAGAGCATGAGCCAATGAG), lin-18f (CCTTTTCTTCTGAGCACACC), lin-18r (CGAATTTCCCGACGCAGAC), glh-3f (TGGAGCCGCCAACAAATGTG), glh-3r (TTTTCTCGGTGGTGTAGGTGTC), mom-1f (TTCTGCCCGATACTCAATGTGTG), mom-1r (TTTGTTTGAGTGTCTGTGCTTGC), cal-1f (ACTGACACCTGAAGAAATCGACG), cal-1r (ATCTCTTGTTCCGTCGGGTTC), alh-8f (TGGAAGAACACATCCCCACTG) and alh-8r (GTGCTCGACGACCTGAAGAC). Assays and data analysis were performed according to the manufacturer's protocol (User Bulletin #2, ABI PRISM® 7700 Sequence Detection System, Perkin Elmer). All samples were run at least in triplicate using *col-1* as the calibrator gene with a dilution of 1/100 of cDNA. The amount of target, normalized to an endogenous reference (128Q) and relative to the calibrator (*col-1*) was calculated using the 2^–∆∆CT^ method and statistical significance determined using paired *t* tests [18].

#### Western blot analysis and fluorescent immunocytochemistry

#### A rabbit polyclonal antibody specific to Ryk has been characterized [19]. The specificity of this antibody was assessed by Western blot analysis using protein extracts from the striatum of 140CAG HD mice. Proteins were extracted using standard methods, separated by SDS-PAGE and analyzed by Western Blotting using the following primary antibodies: Rabbit anti-RYK (Abgent Ab7775a, 1:100), Mouse anti-actin (MP Biomedicals, 1:5000). Secondary antibodies used were Goat-Anti-mouse IgG HRP-conjugated (Biorad, 1:10,000), Goat-Anti-rabbit IgG HRP-conjugated (Biorad, 1:10,000). Proteins were detected by using ECL+ (ECL for actin) and evaluated by densitometry. This examination was accomplished by using a synthetic Ryk antibody blocking peptide (Abgent BP7775a) as indicated by the manufacturer.

#### Immunofluorescence for Ryk was performed on tissue samples from the striatum of 140CAG HD mice and from human striatal HD and normal control tissue specimens. Immunofluorescence was performed as previously described (Holbert et al., 2003) by incubating striatal tissue sections in the Ryk polyclonal antisera (1:500). Combined immunofluorescence for Ryk and either calbindin or NOS immunoactivities using anti-calbindin (1:500; Swant, Bellinzona, Switzerland) and anti-NOS (1:300; Santa Cruz Inc, Santa Cruz, CA) antibodies in Tris-HCl buffer containing 0.3% Triton X-100 for 24–72 h at 4°C. Sections were then rinsed (three times) in PBS, incubated in the dark with goat anti-rabbit FITC conjugate for 2 h at 20°C (Boehringer Mannheim; 1:200), rinsed (three times) in PBS, and incubated with goat anti-mouse tetramethylrhodamine B isothiocyanate (TRITC) conjugate (Boehringer Mannheim; 1:10) for 2 h at 20°C. Detection of Ryk antisera resulted in the presence of red fluorescence. Detection of calbindin and NOS antisera resulted in the presence of green fluorescence. Sections were wet-mounted and coverslipped with 50% glycerol on completion of the technique. Identical microscopic fields were immediately photographed with a Nikon Eclipse E800 fluorescent microscope, delineating the location of Ryk and either calbindin or NOS immunoreactivities within the same striatal section. The fields were merged and co-localization was analyzed. Densitometric analyses of Ryk immunofluorescence were performed on rendered images from both human and mouse striata using Image J.

Combined immunofluorescence for Ryk and GFAP immunoreactivities was performed by incubating striatal tissue sections with anti-Ryk polyclonal antisera (ABGENT; San Diego CA; 1:100) and GFAP (GA5; Chemicon; Billerica MA;1:1000) antibodies in Tris-HCl buffer containing 0.3% Triton X-100 for 24–72 h at 4°C. Sections were then rinsed (three times) in PBS, incubated in the dark with donkey anti-rabbit Cy3 conjugate for 2 h at 20°C (Jackson ImmunoResearch; West Grove PA; 1:500), rinsed (three times) in PBS, and incubated with donkey anti--mouse FITC conjugate (Jackson ImmunoResearch; West Grove PA; 1:500) for 2 h at 20°C. Detection of Ryk antisera resulted in the presence of red fluorescence, while detection of GFAP antisera resulted in the presence of green fluorescence. Sections were wet-mounted and cover slipped with Vectashield mounting medium with DAPI (Vector Laboratories; Burlingame CA). Identical microscopic fields were immediately photographed with a Nikon Eclipse E800 fluorescent microscope, delineating the location of Ryk and GFAP immunoreactivities within the same striatal section. The fields were merged and co-localization was analyzed.

#### Immunocytochemistry

#### Immunohistochemical localization of antibodies to Ryk (Ab7775a, dilution, 1:1000; Abgent, San Diego, CA) was performed by using a conjugated second antibody method. Tissue sections from human striatum were preincubated in an absolute methanol and 0.3% hydrogen peroxide solution for 30 min, washed (three times) in PBS (pH 7.4) for 10 min each, placed in 10% normal goat serum (GIBCO) for 1 h, incubated free-floating in primary antiserum at room temperature for 12–18 h (all dilutions of primary antisera above included 0.08% Triton X-100 and 2% normal goat serum), washed (three times) in PBS for 10 min each, placed in periodate-conjugated goat anti-rabbit IgG (1:300 in PBS, Boehringer Mannheim, Indianapolis) or goat anti-mouse IgG (1:300 in PBS, Boehringer Mannheim), washed (three times) in PBS for 10 min each, and reacted with 3,39 diaminobenzidine HCl (1 mg/ml) in TrisHCl buffer with 0.005% hydrogen peroxide. Specificity for the antisera used in this study was examined in each immunochemical experiment to assist with interpretation of the results. This examination was accomplished by using a synthetic Ryk antibody blocking peptide (Abgent BP7775a, San Diego, CA) and by omission of the primary antibody to determine the amount of background generated from the detection assay.

**References**

1. Delmar P, Robin S, Daudin JJ (2005) VarMixt: efficient variance modelling for the differential analysis of replicated gene expression data. Bioinformatics 21: 502-508.

2. Ashburner M, Ball CA, Blake JA, Botstein D, Butler H, et al. (2000) Gene ontology: tool for the unification of biology. The Gene Ontology Consortium. Nat Genet 25: 25-29.

3. Beissbarth T, Speed TP (2004) GOstat: find statistically overrepresented Gene Ontologies within a group of genes. Bioinformatics 20: 1464-1465.

4. Mootha VK, Lindgren CM, Eriksson KF, Subramanian A, Sihag S, et al. (2003) PGC-1alpha-responsive genes involved in oxidative phosphorylation are coordinately downregulated in human diabetes. Nat Genet 34: 267-273.

5. Subramanian A, Tamayo P, Mootha VK, Mukherjee S, Ebert BL, et al. (2005) Gene set enrichment analysis: a knowledge-based approach for interpreting genome-wide expression profiles. Proc Natl Acad Sci U S A 102: 15545-15550.

6. Eisenmann DM (2005) Wnt signaling. WormBook: 1-17.

7. Greenwald I (2005) LIN-12/Notch signaling in C. elegans. WormBook: 1-16.

8. Sundaram MV (2006) RTK/Ras/MAPK signaling. WormBook: 1-19.

9. Savage-Dunn C (2005) TGF-beta signaling. WormBook: 1-12.

10. Kanehisa M, Goto S (2000) KEGG: kyoto encyclopedia of genes and genomes. Nucleic Acids Res 28: 27-30.

11. Lee I, Lehner B, Crombie C, Wong W, Fraser AG, et al. (2008) A single gene network accurately predicts phenotypic effects of gene perturbation in Caenorhabditis elegans. Nat Genet 40: 181-188.

12. Li S, Armstrong CM, Bertin N, Ge H, Milstein S, et al. (2004) A map of the interactome network of the metazoan C. elegans. Science 303: 540-543.

13. Chen N, Harris TW, Antoshechkin I, Bastiani C, Bieri T, et al. (2005) WormBase: a comprehensive data resource for Caenorhabditis biology and genomics. Nucleic Acids Res 33: D383-389.

14. Rual JF, Venkatesan K, Hao T, Hirozane-Kishikawa T, Dricot A, et al. (2005) Towards a proteome-scale map of the human protein-protein interaction network. Nature 437: 1173-1178.

15. Giot L, Bader JS, Brouwer C, Chaudhuri A, Kuang B, et al. (2003) A protein interaction map of Drosophila melanogaster. Science 302: 1727-1736.

16. Lee I, Li Z, Marcotte EM (2007) An improved, bias-reduced probabilistic functional gene network of baker's yeast, Saccharomyces cerevisiae. PLoS ONE 2: e988.

17. Rapaport F, Zinovyev A, Dutreix M, Barillot E, Vert JP (2007) Classification of microarray data using gene networks. BMC Bioinformatics 8: 35.

18. Livak KJ, Schmittgen TD (2001) Analysis of relative gene expression data using real-time quantitative PCR and the 2(-Delta Delta C(T)) Method. Methods 25: 402-408.

19. Keeble TR, Halford MM, Seaman C, Kee N, Macheda M, et al. (2006) The Wnt receptor Ryk is required for Wnt5a-mediated axon guidance on the contralateral side of the corpus callosum. J Neurosci 26: 5840-5848.
