# Supplementary material for: The Wnt Receptor Ryk Reduces Neuronal and Cell Survival Capacity by Repressing FOXO Activity During the Early Phases of Mutant Huntingtin Pathogenicity
Source: PLoS Biol. 2014 Jun 24;12(6):e1001895. doi: 10.1371/journal.pbio.1001895 (PMC4068980; doi:10.1371/journal.pbio.1001895)
Supplement: Supplementary file 13 [file pbio.1001895.s013.docx]

Gene set	Genes specifically dysregulated in 128Q cells			Genes specifically dysregulated in 19Q cells			Size
	NES	Nom p-Val	FDR q-Val	NES	Nom p-Val	FDR q-Val	
**Enrichment (FDR q-Val < 0.25) in 128Q and not 19Q cells**							
NEURON DIFFERENTIATION¥	-1.60	0.006	0.019	-1.19	0.17	0.346	46
TGF-ß SIGNALING*	-1.44	0.053	0.208	-0.80	0.77	1.0	31
G-PROTEIN SIGNALING PATHWAY¥	-1.39	0.079	0.079	-1.05	0.38	0.362	29
INOSITOL PHOSPHATE METABOLISM$	-1.30	0.118	0.115	1.19	0.19	0.345	31
AXON GUIDANCE #	-1.28	0.138	0.067	-0.95	0.53	1.0	36
RESPONSE TO OXIDATIVE STRESS¥	-1.12	0.270	0.226	-0.87	0.68	0.766	38
NUCLEUS¥	1.28	0.032	0.123	-0.94	0.65	0.687	237
WNT SIGNALING*	1.32	0.087	0.100	-1.07	0.33	0.725	51
GLYCOLYSIS/GLUOGENESIS$	1.72	0.009	0.012	-0.62	0.96	0.969	30
CELL CYCLE#	1.73	0.0	0.002	-0.88	0.81	0.759	234
MITOCHONDRIA¥	2.27	0.0	0.0	0.63	0.99	0.979	94
OXIDATIVE PHOSPHORYLATION$	2.36	0.0	0.0	-0.91	0.66	1.0	85
**Enrichment (FDR q-Val < 0.25) in both 128Q and 19Q cells**							
PLASMA MEMBRANE¥	-1.69	0.0	0.008	-1.20	0.11	0.249	124
SYNAPSE¥	-1.61	0.004	0.009	-1.44	0.05	0.168	35
EXTRACELLULAR PART¥	1.67	0.010	0.012	-1.29	0.14	0.199	33
DNA REPAIR¥	1.69	0.0	0.001	1.69	0.0	0.003	70
RIBOSOME$	2.21	0.0	0.0	2.12	0.0	0.0	25
PROTEASOME$	2.35	0.0	0.0	2.38	0.0	0.0	29
**No enrichment**							
RTK/RAS/MAPKinase SIGNALING*	-1.19	0.172	0.414	-1.33	0.07	0.387	55
OSMOREGULATION¥	-1.03	0.367	0.389	0.88	0.81	0.690	187
INSULINE SIGNALING*	-0.93	0.555	0.760	-0.79	0.78	0.814	22
NOTCH SIGNALING*	-0.65	0.938	0.958	0.76	0.79	0.845	26
BASAL TRANSCRIPTION FACTORS$	0.65	0.915	0.949	0.96	0.5	0.724	21
SNARE VESICLES/PROTEIN EXPORT$	0.75	0.852	0.981	1.21	0.16	0.425	31
UBIQUITIN/AUTOPHAGY$	0.76	0.819	1.0	0.65	0.93	1.0	24
ENDOPLASMIC RETICULUM/GOLGI¥	0.80	0.751	0.847	-0.73	0.82	0.930	17
APOPTOSIS¥	0.89	0.663	0.724	0.79	0.82	0.891	43
GLUTAMATE METABOLISM$	1.23	0.186	0.277	0.52	0.99	0.996	29
